# Supplementary material for: Stakeholder integration predicts better outcomes from groundwater sustainability policy
Source: Nat Commun. 2023 Jun 27;14:3793. doi: 10.1038/s41467-023-39363-y (PMC10300030; doi:10.1038/s41467-023-39363-y)
Supplement: Supplementary file 1 — Supplementary Information [file 41467_2023_39363_MOESM1_ESM.pdf]

Supplementary Information for

Stakeholder integration predicts better outcomes from groundwater sustainability policy

Debra Perrone*, Melissa M. Rohde*, Courtney Hammond Wagner*, Rebecca Anderson, Samantha Arthur, Ngodoo Atume, Meagan Brown, Lauren Esaki-Kua, Martha Gonzalez Fernandez, Kelly A. Garvey, Katherine Heidel, William D. Jones, Sara Khosrowshahi Asl, Carrie Munill, Rebecca Nelson, J. Pablo Ortiz-Partida, E.J. Remson

*These authors contributed equally

Table of Contents

Supplementary Section 1. Sustainable Groundwater Management Act overview 2
Description of the Sustainable Groundwater Management Act 2
Supplementary Section 2. Groundwater Sustainability Plan Review 8
Operationalizing stakeholder integration 8
Identifying stakeholder presence for Sustainability Plan review 10
Supplementary Section 3. Sensitivity Analysis for Agriculture Engage Component 53
Supplementary Section 4. Cover and Protection Analysis 63
Cover and Protection Sensitivity Analysis 63
Analysis of Undesirable Results and Minimum Threshold Criteria in Sustainability Plans 64
Supplementary Section 5. Coverage and Protection Results by Groundwater Sustainability Plan 82
Supplementary Section 6. Integration Statistics 164
Supplementary Section 7. Integration/Protection Sensitivity Analysis: Ag Engage 179
Supplementary Section 8. Post-hoc Exploratory Analysis 186
Supplementary Section 9. References 195

Supplementary Section 1. Sustainable Groundwater Management Act overview

Description of the Sustainable Groundwater Management Act

In 2014, California passed the Sustainable Groundwater Management Act to manage groundwater resources sustainably. Under the Sustainable Groundwater Management Act, newly formed local groundwater sustainability agencies (Agencies) are required to bring groundwater basins into balance and achieve sustainability within a 20-year planning horizon. If local Agencies fail to meet the state mandate, the State (i.e., State Water Resources Control Board) will intervene and manage the basin on behalf of the local Agency. Agencies are required to prepare and submit Groundwater Sustainability Plans (Sustainability Plans) every five years along with annual reports. The Sustainability Plans and reports are evaluated by the California Department of Water Resources, and Sustainability Plans that are deemed "insufficient" are taken over by the State Water Resources Control Board after a lengthy statutory process.

The Sustainable Groundwater Management Act defines sustainability by the avoidance of six 'undesirable results': (1) chronic lowering of groundwater levels, (2) groundwater storage loss, (3) water quality degradation, (4) land subsidence, (5) seawater intrusion, and (6) surface water depletion. Undesirable results occur when "significant and unreasonable effects [...] are caused by groundwater conditions occurring throughout the basin" [23 CCR 356.26(a)]. Within Sustainability Plans, Agencies are tasked with establishing sustainable management criteria to measure whether groundwater sustainability is being achieved for the six sustainability indicators that correspond to each undesirable result: (1) groundwater levels, (2) groundwater storage, (3) water quality, (4) land subsidence, (5) seawater intrusion, and (6) interconnected surface water. For each sustainability indicator, Agencies must establish minimum thresholds and measurable objectives to quantify groundwater conditions at each monitoring site and track overall progress towards the sustainability goal. Minimum thresholds are numeric values that, if exceeded, may cause undesirable results in the basin, and deem the basin unsustainable. Sustainability Plans have the responsibility to define minimum thresholds and significant and unreasonable effects to the basin based on minimum thresholds with local discretion in how to do so using widely varied methods (Supplementary Tables 4.5-4.6) Alternatively, measurable objectives are numeric metrics to measure whether the sustainability goal is achieved over the planning horizon. The Sustainable Groundwater Management Act requires that potential effects on the beneficial uses and users of groundwater be described when defining undesirable results and minimum thresholds.

On its surface, the Sustainable Groundwater Management Act appears to promise comprehensive groundwater management for all groundwater, but it only applies to state-designated alluvial groundwater basins[1]. Across California, there are 515 groundwater basins. Each basin has been ranked by the California Department of Water Resources into four categories - critically overdrafted, high priority, medium priority, low priority, and very low priority (https://water.ca.gov/Programs/Groundwater-Management/Basin-Prioritization). Only critically overdrafted, high priority and medium priority basins are required to form Agencies and submit Sustainability Plans, whereas low and very low priority basins can opt to voluntarily do so (Supplementary Fig. 1.1). The planning horizon to achieve sustainability is different for critically overdrafted basins and high and medium priority basins. Critically overdrafted basins are required to achieve sustainability by 2040 and submit their first Plan in 2020. High and medium priority basins are required to achieve sustainability by 2042 and submit their first Plan in 2022.

On paper, California's groundwater law mandates sustainable outcomes and engagement, and requires monitoring to prove outcomes occur by checking progress along the way. The Sustainable Groundwater Management Act legislation and regulations express stakeholder engagement and consideration requirements in several ways, at different stages of the process, as well as overarching stakeholder involvement and consideration requirements (Supplementary Table 1.1). Additionally, the Department of

Water Resources encouraged Agencies to include beneficial users in more collaborative style processes through dissemination of a non-binding best practices document for stakeholder engagement which follows the International Association for Public Policy's spectrum of stakeholder engagement[2].

The Sustainable Groundwater Management Act legislation also facilitates locally tailored solutions, which was crucial to its successful passage. At the same time, local control of common-pool natural resources can entrench powerful local vested interests through landowner-based and acreage-based voting[3] on Boards; this, in turn, can threaten how access is facilitated long-term or how stakeholders are involved in future and more long-term decision-making processes that determine access. Local control was central to California's pre-Sustainable Groundwater Management Act voluntary groundwater management and planning efforts (AB3030 and Special Management Acts Districts), which ultimately did not correct or halt overdraft[4]. Although some local jurisdictions in California have self-organized successfully[5,6], the majority of areas with depletion have not adopted legal or other mechanisms to address depletion successfully. The Sustainable Groundwater Management Act represents the first time that basins across California have had to assess supply and demand quantitatively – whether the approach can be implemented to successfully reach sustainability will likely take years to see given the long-time frames.

Agencies have full and express legal powers to adopt rules and regulations and other measures to implement Sustainability Plans (California Water Code Div. 6, Pt. 2.74, Ch. 5), including express legal enforcement powers (California Water Code § 10726.8), with accompanying powers to investigate and penalize non-compliance with associated rules (California Water Code §§ 10725.4(a)(4), 10732). However, many factors are likely to pose a practical challenge to implementation and ultimate enforcement. Implementation efforts may encounter litigation brought by groundwater pumpers, whose common law rights survive the Sustainable Groundwater Management Act legislation[7]. The Sustainable Groundwater Management Act expressly foresees, and provides for, court actions to establish the validity of Sustainability Plans (California Water Code § 10726.6). Agencies responding to local pressure from groundwater pumpers may seek to avoid regulatory approaches to implementation, like allocation regimes, in favor of infrastructure measures like heavy reliance on managed aquifer recharge that face their own noted challenges to successful implementation[8,9]. Local Agencies may face the same cultural and political constraints and resources limitations to enforcing their rules that have affected groundwater governance in other jurisdictions[10]. Whether the State Water Resources Control Board will use its legal powers to step-in in response to local implementation gaps remains to be seen. Ultimately, the performance of the Sustainable Groundwater Management Act will only be as good as its implementation and enforcement in practice.

Supplementary Table 1.1. Key legislative and regulatory requirements to involve and consider stakeholders (non-exhaustive) in California's 2014 Sustainable Groundwater Management Act by phase in the policy process.

**Stage of Sustainable Groundwater Management Act Planning Process**	**Legislative or regulatory requirement and reference**<br>**CWC = California Water Code; CCR = California Code of Regulations**
Overarching (not specific to any phase)	An Agency must 'encourage the active involvement of diverse social, cultural, and economic elements of the population within the groundwater basin prior to and during the development and implementation of the groundwater sustainability plan' (CWC § 10727.8(a))
	An Agency must 'consider the interests of all beneficial uses and users of groundwater', including agricultural users, domestic well owners, municipal well operators, public water systems, local land use planning agencies, environmental users of groundwater, users of hydrologically connected surface waters, the federal government, tribes, and disadvantaged communities (CWC § 10723.2)
Prior to Plan development	An Agency must provide a statement about how 'interested parties may participate in the development and implementation' of the Plan and must post this information on its website (CWC § 10727.8(a); 23 CCR § 353.6)
	An Agency may 'appoint and consult with an advisory committee consisting of interested parties' (CWC § 10727.8(a))<br>'Interested parties' is a defined term (CWC § 10723.4; 23 CCR § 351(p)): 'The groundwater sustainability agency shall establish and maintain a list of persons interested in receiving notices regarding plan preparation, meeting announcements, and availability of draft Sustainability Plans, maps, and other relevant documents. Any person may request, in writing, to be placed on the list of interested persons.'
Proposed Plan, not yet adopted	'Any person may provide comments' about a proposed Plan, including a decision to develop a Plan, to the Department of Water Resources with a copy to the Agency (23 CCR § 353.8(a), (c))
	An Agency must hold a public hearing; curiously, the Agency is only expressly required to 'review and consider' comments received from a city or county notified about the Plan (CWC § 10728.4)
Contents of Plan	The Plan must include descriptive information about consultation and beneficial uses and users. It must:<br>● Describe beneficial uses and users of groundwater in the Basin, 'the types of parties representing those interests, and the nature of consultation with those parties' (23 CCR§ 354.10(a))<br>● List the public meetings at which the Plan was discussed or considered (23 CCR § 354.10(b))<br>● Summarize comments made about the Plan and the Agency's responses (23 CCR § 354.10(c))

Stage of Sustainable Groundwater Management Act Planning Process	Legislative or regulatory requirement and reference<br>CWC = California Water Code; CCR = California Code of Regulations
	● Include a communication Section of the Plan that explains the Agency's decision-making process, identify opportunities for engagement and discuss how input will be used, describe how the Agency fulfills its overarching Sustainable Groundwater Management Act involvement requirement, and how the Agency will inform the public about implementation (23 CCR § 354.10(d))
	The Plan must include descriptive information about the physical setting and characteristics of the Basin, which is used as the basis for 'defining and assessing reasonable sustainable management criteria and projects and management actions' (23 CCR § 354.12), namely information about:<br>● the primary uses of each aquifer (e.g., domestic, irrigation, or municipal supply) (23 CCR § 354.14(b)(4)(E));<br>● groundwater conditions, including groundwater-dependent ecosystems in the basin (23 CCR § 354.16(g));<br>● outflows from the basin by water use sector (23 CCR § 354.18(b)(3))
	The Plan's description of undesirable results must include a description of the 'potential effects on the beneficial uses and users of groundwater, on land uses and property interests, and other potential effects that may occur or are occurring from undesirable results' (23 CCR § 354.26(b)(3))
	The Plan's description of minimum thresholds must include a description of 'how minimum thresholds may affect the interests of beneficial uses and users of groundwater or land uses and property interests' (23 CCR § 354.28(b)(4))
	The Plan must include a description of the monitoring network objectives for the basin, which 'shall be implemented to … [m]onitor impacts to the beneficial uses or users of groundwater' (23 CCR § 354.34(b)(2)).<br>The 'density of monitoring sites and frequency of measurements' must be based on, among other things, '[i]mpacts to beneficial uses and users of groundwater and land uses and property interests affected by groundwater production' (23 CCR § 354.34(f)(3)).
	The Plan must describe projects and management actions to achieve the sustainability goal for the basin, including those to be used where undesirable results 'have occurred or are imminent', and a description of the criteria for implementing the projects and actions (23 CCR § 354.44(a), (b), (1)(A)).<br>NB: this links with stakeholders given that stakeholders may be affected by undesirable results: see above 'Contents of Plan - undesirable results'
Following Plan submission to the California Department of Water Resources	A 60-day public comment period commences after the Department of Water Resources posts a Plan submitted by an Agency on its website (California Water Code § 10733.4; 23 CCR §§ 353.8, 355.2(c))

**Stage of Sustainable Groundwater Management Act Planning Process**	**Legislative or regulatory requirement and reference**<br>**CWC = California Water Code; CCR = California Code of Regulations**
Plan Evaluation by the California Department of Water Resources	In evaluating the Plan and undertaking periodic review of the Plan, the Department of Water Resources expressly must consider '[w]hether the interests of the beneficial uses and users of groundwater in the basin, and the land uses and property interests potentially affected by the use of groundwater in the basin, have been considered.' (23 CCR §§ 355.4(b)(4), 355.6(c)(4))
Following Plan Evaluation by Department of Water Resources	[If the State Water Resources Control Board initiates the process of using its step-in power] Further public comment is provided for in legislation (California Water Code § 10735.2)
Ongoing Requirement	'Each Agency shall adjust the monitoring frequency and density of monitoring sites' to detail water conditions and 'assess the effectiveness of management actions under circumstances that include … [a]dverse impacts to beneficial uses and users' (23 CCR § 354.38(e)(3))

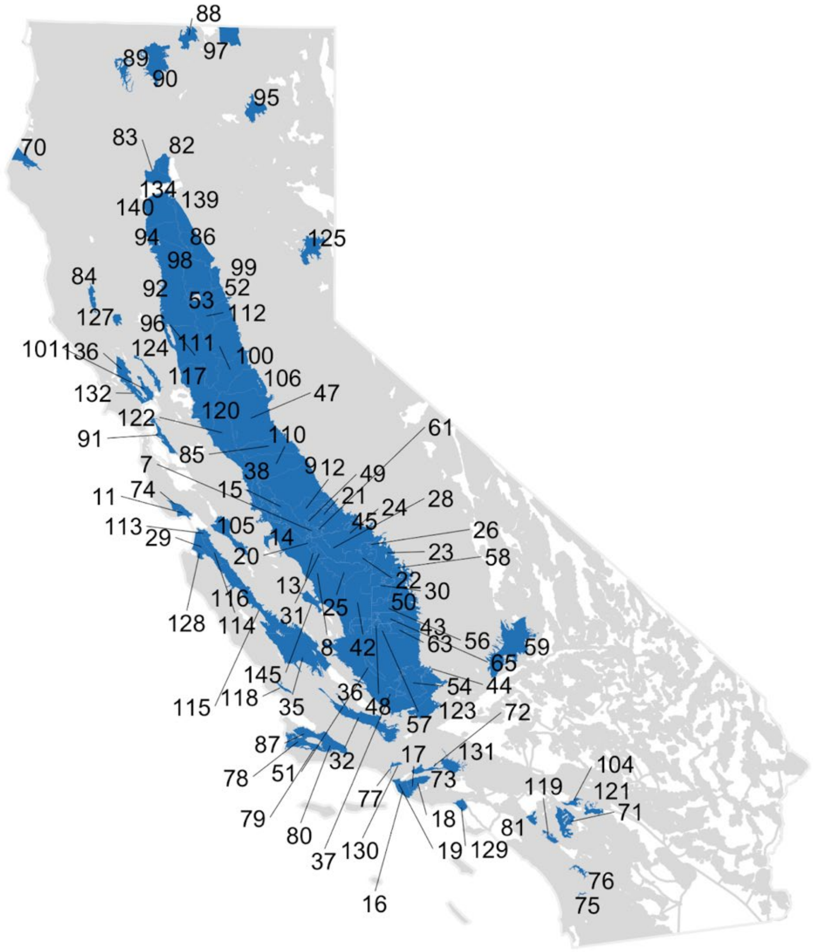

Supplementary Fig. 1.1. Groundwater Sustainability Plans in California. Groundwater basins that are designated as being critical, high priority, or medium priority (blue) are required to develop and implement groundwater sustainability plans to achieve groundwater sustainability within a 20-year planning horizon. Groundwater basins in low and very low priority basins (white) are not required to submit plans. Non-basin areas (grey) are not subject to the Sustainable Groundwater Management Act.

Supplementary Section 2. Groundwater Sustainability Plan Review

Operationalizing stakeholder integration

A diverse body of literature at the intersection of stakeholder engagement, collaborative governance, environmental justice and community-based natural resource management posits that the integration of stakeholders into environmental policy processes leads to more equitable policy and better outcomes for stakeholders[11–15]. Alongside this literature, natural resource management regimes are increasingly turning towards more participatory processes, including water law regimes around the world[16]. When local users are integrated into decision making the resulting management regimes are more likely to contain locally appropriate rules, gain community trust and support, and achieve resource management goals[17–19].

Numerous case study approaches suggest evidence both for and against stakeholder engagement leading to improved environmental outcomes[14,15]. However, very few studies have statistically demonstrated a link between stakeholder participation and improved outcomes[12], though a notable exception is a study by Scott[20] which demonstrates improved water quality conditions amongst collaborative vs. non-collaborative watershed groups in a sample of 357 watersheds. The literature has demonstrated that stakeholder participation can lead to improved environmental outcomes, but that the outcomes are not automatic, and the conditions under which the benefits emerge are not clear[14,21,22]. Evidence for the link between stakeholder engagement and environmental outcomes has remained elusive[12,23]and many of the core tenets underlying the theory of stakeholder participation remain untested[14,15]. Our work builds on these bodies of literature by examining the integration of stakeholders into the Sustainable Groundwater Management Act process.

We use Sustainability Plans as the unit of analysis in this study, and thus we evaluate stakeholder integration as described within the text of the Sustainability Plans. We focus on the phenomenon of integration, rather than stakeholder participation or collaboration, because we are analyzing how well stakeholders are integrated into the planning process and the ensuing outcomes from that process. We are thus able to examine both stated levels of stakeholder engagement and the evidence of stakeholder-specific description, analysis, and proposed benefits within the plan. Drawing from Newig et al.'s[14] hypothesized causal pathways linking stakeholder participation and environmental outcomes, we hypothesized that stakeholder participation in the Sustainability Plan would increase representation of respective stakeholder concerns and increase relevant local knowledge needed in the decision-making process to improve the stakeholder outcomes from the Sustainability Plan.

To analyze stakeholder integration into Sustainability Plans, we classified and evaluated four generalizable components ("integration components") that we used to assess and compare how different stakeholders were incorporated into Sustainability Plans and were coherent with Sustainable Groundwater Management Act regulations (Supplementary Table 2.1). The four components of integration are: 1) *engage* - the level of active stakeholder engagement in the planning process (e.g., advisory committee member, invited stakeholder governing board seat), 2) *describe* - the identification of stakeholder's physical relationship to groundwater via maps and geographical description, 3) *analyze* - the qualitative or quantitative analysis of impacts to a stakeholder group in setting management criteria, and 4) *act* - actions for implementation to achieve sustainability include explicit benefits to the stakeholder. We evaluated each component through a rubric we developed containing a series of questions that we applied across stakeholder groups and Sustainability Plans. This enabled us to calculate scores for each component, ranging from zero to two, where higher scores indicated greater engagement, description, analysis, and planned actions for implementation respectively. We then calculated an aggregate score (*aggregate)* to assess how well each Plan incorporated a stakeholder across the four components. This aggregate score is a simple sum of the component scores.

In line with Refs 14, 15, and 22, we define *engage* as evidence for stakeholder participation in deliberation or decision-making. A designated stakeholder seat on an advisory committee and/or working group or a designated stakeholder seat on the Agency board both represent invited stakeholder engagement in deliberation and/or decision-making. In our application of *engage*, and the other components, we evaluated only explicit mentions of invited seats for stakeholders as evidence (e.g., a designated seat for domestic well owners on the Agency board) and not general statements that allude to potential representation (e.g., stakeholders were represented in advisory committees).

The remaining three integration components, *describe*, *analyze*, and *act*, represent elements of a policy and/or planning process that stakeholder engagement can affect and/or stakeholder groups will be affected by. Drawing from the literature on collaborative governance and planning theory, the planning process can be seen as consisting of stages and/or a cycle through which a problem is defined, approaches to addressing the problem are identified and then a decision is made to select an approach(es)[13,24]. The planning document itself represents this process through elements of the plan.

Identifying stakeholder presence for Sustainability Plan review

For each Sustainability Plan area, we evaluated Sustainability Plan text, and spatial information on well and groundwater-dependent ecosystems to determine whether a Sustainability Plan area should be considered "Not Applicable" (NA) for a given stakeholder group. Supplementary Table 2.2 summarizes our determination for presence or absence of each stakeholder group by Sustainability Plan area.

To evaluate the presence or absence of a stakeholder group in each Sustainability Plan area, we followed a three-step process, see Supplementary Table 2.3. First, from the rubric analysis, Sustainability Plans were flagged as a potential NA for a stakeholder group if the Sustainability Plan stated that the stakeholder group was not present in the Sustainability Plan area, or if the Sustainability Plan received zeros across all components. Second, in the well and groundwater-dependent ecosystem analysis, a Sustainability Plan was flagged as a potential NA if there were no wells or groundwater-dependent ecosystems in the Sustainability Plan area according to the Online System of Well Completion Reports database (OSWCR; https://water.ca.gov/Programs/Groundwater-Management/Wells/Well-Completion-Reports), the Natural Communities Commonly Associated with Groundwater database[25], and the Disadvantaged Community mapping tool (https://gis.water.ca.gov/app/dacs/). Finally, our third step was to reconcile Sustainability Plan text with the mapping analysis to determine whether a stakeholder group should be considered an NA for the analysis. We note that our disadvantaged community analysis focused on domestic wells found in disadvantaged communities, not municipal supplies or small community water systems. Therefore, Sustainability Plans that have disadvantaged communities with municipal supply or small community water systems may be excluded from our sub-group analysis and marked as an NA if they do not have domestic wells in the disadvantaged community. As small farms are not geographically identifiable via spatial or well data, we rely on the main agriculture stakeholder category status to determine presence or absence of small farms in a Sustainability Plan area.

Supplementary Table 2.1. Integration evaluation rubric questions for defining the four integration components: *engage*, *describe*, *analyze*, *act*. Each question was evaluated for each stakeholder group (i.e., agriculture, domestic, environment, disadvantaged communities, and small and medium farms) within a groundwater sustainability plan, except where noted. Related legal or administrative references come from the California Water Code (CWC) and California Code of Regulations (CCR) or noted additional administrative references.

Component	Definition	Question	Response Levels	Related Legal or Administrative Reference
Engage	Stakeholder participation in Plan deliberation or decision-making	Does the Sustainability Plan document opportunities for 1) explicit stakeholder involved in consistent, broad-ranging discussion and two-way engagement on Sustainability Plan content (e.g., citizen advisory committees and/or working groups), and/or 2) explicit stakeholder involved in voting on Plan related process and outputs (e.g., stakeholder seat on the Sustainability Plan board?	NA<br>0. No - no advisory committee member or invited stakeholder board seat for the stakeholder group<br>1. Somewhat - either an advisory committee member or invited stakeholder board seat for the stakeholder group<br>2. Yes - both an advisory committee member and invited stakeholder board seat for the stakeholder group	"Prior to initiating the development of a groundwater sustainability plan, the groundwater sustainability agency shall make available to the public and the department a written statement describing the manner in which interested parties may participate in the development and implementation of the groundwater sustainability plan. The groundwater sustainability agency shall provide the written statement to the legislative body of any city, county, or city and county located within the geographic area to be covered by the plan. The groundwater sustainability agency may appoint and consult with an advisory committee consisting of interested parties for the purposes of developing and implementing a groundwater sustainability plan. The groundwater sustainability agency shall encourage the active involvement of diverse social, cultural, and economic elements of the population within the groundwater basin prior to and during the development and implementation of the groundwater sustainability plan [...]" [CWC § 10727.8(a)]<br><br>"The groundwater sustainability agency shall establish and maintain a list of persons interested in receiving notices regarding plan preparation, meeting announcements, and availability of draft plans, maps, and other relevant documents. Any person may request, in writing, to be placed on the list of interested persons." [CWC §10723.4]<br><br>"'Interested parties' refers to persons and entities on the list of interested persons established by the Agency [...]" [23 CCR § 351(p)]<br><br>"When submitting an adopted Plan to the Department, the Agency shall include… the following information: ... The organization and management structure of the Agency, identifying persons with management authority for implementation of the Plan". [23 CCR § 354.6(b)]

Component	Definition	Question	Response Levels	Related Legal or Administrative Reference
				"Each Plan shall include a summary of information relating to notification and communication by the Agency with other agencies and interested parties including the following: … A communication Section of the Plan that includes the following: (1) An explanation of the Agency's decision-making process. (2) Identification of opportunities for public engagement and a discussion of how public input and response will be used. (3) A description of how the Agency encourages the active involvement of diverse social, cultural, and economic elements of the population within the basin. (4) The method the Agency shall follow to inform the public about progress implementing the Plan, including the status of projects and actions. [23 CCR § 354.10(d)]<br><br>California Department of Water Resources. 2018. Guidance Document for Groundwater Sustainability Plan: Stakeholder Communication and Engagement. Available at: https://water.ca.gov/-/media/DWR-Website/Web-Pages/Programs/Groundwater-Management/Assistance-and-Engagement/Files/Guidance-Doc-for-GSP---Stakeholder-Communication-and-Engagement.pdf
Describe	Description of stakeholder's physical relationship to groundwater via maps and geography	Are [stakeholder] groundwater users identified and mapped in the basin? (i.e., For well-based users: does the Sustainability Plan map minimum well depth, or depth range, of wells? For environment: were groundwater-dependent ecosystems in the basin identified	NA<br>0. No - latitude and longitude not represented (e.g., density map, point map) and depth not described in Sustainability Plan text<br>1. Somewhat - latitude and longitude represented (e.g., density map, point map) and depth not described in Sustainability Plan text<br>2. Yes - latitude and longitude represented (e.g., density map, point map) and depth described in Sustainability Plan text or mapped	"Each Plan shall provide a description of current and historical groundwater conditions in the basin, including data from January 1, 2015, to current conditions, based on the best available information that includes the following: … Identification of groundwater dependent ecosystems within the basin, utilizing data available from the Department, as specified in Section 253.2, or the best available information." [23 CCR §354.16(g)]<br><br>"The hydrogeologic conceptual model shall be summarized in a written description that includes the following: … (4) Principal aquifers and aquitards, including the following information: …. (E) Identification of the primary use or uses of each aquifer, such

Component	Definition	Question	Response Levels	Related Legal or Administrative Reference
		(mapped) and described in the Sustainability Plan using best available data?)		as domestic, irrigation, or municipal water supply." [23 CCR § 354.14(b)(4)(E)]
Analyze	The qualitative or quantitative analysis of impacts to a stakeholder group in setting management criteria	Does the groundwater elevation minimum threshold evaluate the potential impacts on [stakeholder]?	NA (e.g., explicitly no stakeholder group use in basin)<br>0. No - No mention of stakeholder group when analyzing impacts of minimum thresholds<br>1. Somewhat - qualitative description of impacts to stakeholder group when analyzing minimum thresholds<br>2. Yes - quantitative analysis of impacts to stakeholder group when analyzing minimum thresholds	"The description of minimum thresholds shall include the following: ... How minimum thresholds may affect the interests of beneficial uses and users of groundwater or land uses and property interests." [23 CCR §354.28(b)(4)]<br><br>"In addition to the requirements of Section 10727.2, a groundwater sustainability plan shall include, where appropriate and in collaboration with the appropriate local agencies … Impacts on groundwater dependent ecosystems" [CWC § 10727.4(l)]
Act	Actions for implementation to achieve sustainability include explicit benefits to the stakeholder	Does the Sustainability Plan include any Project and Management Actions with explicit benefits to [stakeholder]?	NA<br>0. No - No mention of explicit benefits to stakeholder group from any projects or management actions<br>1. Somewhat - Benefits to stakeholder group explicitly identified from at least one project or management action identified, but benefits not well described<br>2. Yes - Benefits to stakeholder group explicitly identified from at least one project or management action identified and benefits are well described	"Each Plan shall include a description of the projects and management actions that include the following: … An explanation of the benefits that are expected to be realized from the project or management action, and how those benefits will be evaluated." [23 CCR §354.44(b)(5)]

Supplementary Table 2.2. Groundwater Sustainability Plans included in study indicating the presence/absence of each stakeholder group in the basin.

Basin Name	GSP ID	Basin Number	Year Submitted	Agriculture	Domestic	Environment	Disadvantaged Communities
180-400 Foot Aquifer	29	3-004.01	2020	yes	yes	yes	yes
Anderson	83	5-006.03	2022	yes	yes	yes	yes
Antelope	134	5-021.54	2022	yes	yes	yes	yes
Big Valley	95	5-004	2022	yes	yes	yes	yes
Big Valley	127	5-015	2022	yes	yes	yes	yes
Butte	98	5-021.70	2022	yes	yes	yes	yes
Butte Valley	88	1-003	2022	yes	yes	yes	yes
Chowchilla	12	5-022.05	2020	yes	yes	yes	yes
Colusa	92	5-021.52	2022	yes	yes	yes	yes
Corning	94	5-021.51	2022	yes	yes	yes	yes
Cosumnes	106	5-022.16	2022	yes	yes	yes	yes
Cuyama Valley	32	3-013	2020	yes	yes	yes	yes
Delta-Mendota: Aliso	7	5-022.07	2020	yes	yes	yes	no
Delta-Mendota: Farmers	14	5-022.07	2020	yes	yes	yes	yes
Delta-Mendota: Fresno County	20	5-022.07	2020	yes	yes	yes	yes
Delta-Mendota: Grassland	38	5-022.07	2020	yes	yes	yes	yes
Delta-Mendota: North Central	13	5-022.07	2020	yes	yes	yes	yes
Delta-Mendota: SJREC	15	5-022.07	2020	yes	yes	yes	yes
East Bay Plain	91	2-009.04	2022	no	yes	yes	yes
East Contra Costa	120	5-022.19	2022	yes	yes	yes	yes

Basin Name	GSP ID	Basin Number	Year Submitted	Agriculture	Domestic	Environment	Disadvantaged Communities
East Side Aquifer	114	3-004.02	2022	yes	yes	yes	yes
Eastern San Joaquin	47	5-022.01	2020	yes	yes	yes	yes
Eel River Valley	70	1-010	2022	yes	yes	yes	yes
Elsinore Valley	119	8-004.01	2022	yes	yes	yes	yes
Enterprise	82	5-006.04	2022	yes	yes	yes	yes
Fillmore	73	4-004.05	2022	yes	yes	yes	yes
Forebay Aquifer	116	3-004.04	2022	yes	yes	yes	yes
Indian Wells Valley	59	6-054	2020	yes	yes	yes	yes
Kaweah: East Kaweah	58	5-022.11	2020	yes	yes	yes	yes
Kaweah: Greater Kaweah	30	5-022.11	2020	yes	yes	yes	yes
Kaweah: Mid-Kaweah	50	5-022.11	2020	yes	yes	yes	yes
Kern County: BVGSA	51	5-022.14	2020	yes	yes	yes	yes
Kern County: Henry Miller	37	5-022.14	2020	yes	no	yes	no
Kern County: KGA	36	5-022.14	2020	yes	yes	yes	yes
Kern County: KRGSA	54	5-022.14	2020	yes	yes	yes	yes
Kern County: Olcese	44	5-022.14	2020	yes	no	yes	no
Kings: Central	22	5-022.08	2020	yes	yes	yes	yes
Kings: River East	23	5-022.08	2020	yes	yes	yes	yes
Kings: James	31	5-022.08	2020	yes	yes	yes	yes

Basin Name	GSP ID	Basin Number	Year Submitted	Agriculture	Domestic	Environment	Disadvantaged Communities
Kings: McMullin Area	28	5-022.08	2020	yes	yes	yes	yes
Kings: North Forks	25	5-022.08	2020	yes	yes	yes	yes
Kings: North Kings	24	5-022.08	2020	yes	yes	yes	yes
Kings: South	26	5-022.08	2020	yes	yes	yes	yes
Langley Area	113	3-004.09	2022	yes	yes	yes	yes
Las Posas Valley	18	4-008	2022	yes	yes	yes	no
Los Molinos	139	5-021.56	2022	yes	yes	yes	yes
Madera: GFWD	61	5-022.06	2020	yes	yes	no	yes
Madera: Madera Joint	21	5-022.06	2020	yes	yes	yes	yes
Madera: New Stone	49	5-022.06	2020	yes	yes	no	no
Madera: RCWD	45	5-022.06	2020	yes	yes	yes	no
Merced	9	5-022.04	2020	yes	yes	yes	yes
Modesto	85	5-022.02	2022	yes	yes	yes	yes
Monterey	128	3-004.10	2022	yes	yes	yes	no
Mound	19	4-004.03	2022	yes	no	yes	no
Napa Valley	124	2-002.01	2022	yes	yes	yes	yes
North American	100	5-021.64	2022	yes	yes	yes	yes
North San Benito	105	3-003.05	2022	yes	yes	yes	yes
North Yuba	53	5-021.60	2020	yes	yes	yes	yes
Ojai Valley	130	4-002	2022	yes	yes	yes	no

Basin Name	GSP ID	Basin Number	Year Submitted	Agriculture	Domestic	Environment	Disadvantaged Communities
Oxnard	16	4-004.02	2020	yes	yes	yes	yes
Paso Robles Area	35	3-004.06	2020	yes	yes	yes	yes
Petaluma Valley	132	2-001	2022	yes	yes	yes	yes
Piru	72	4-004.06	2022	yes	yes	yes	yes
Pleasant Valley	17	4-006	2020	yes	yes	yes	no
Pleasant Valley	145	5-022.10	2022	yes	yes	yes	yes
Red Bluff	140	5-021.50	2022	yes	yes	yes	yes
San Antonio Creek Valley	87	3-014	2022	yes	yes	yes	yes
San Gorgonio Pass	121	7-021.04	2022	no	yes	yes	yes
San Jacinto	71	8-005	2022	yes	yes	yes	yes
San Luis Obispo Valley	118	3-009	2022	yes	yes	yes	yes
San Pasqual Valley	75	9-010	2022	yes	yes	yes	no
Santa Clara River Valley East	131	4-004.07	2022	yes	yes	yes	yes
Santa Cruz Mid-County	11	3-001	2020	yes	yes	yes	yes
Santa Margarita	74	3-027	2022	yes	yes	yes	yes
Santa Monica	129	4-011.01	2022	no	no	yes	no
Santa Rosa Plain	136	1-055.01	2022	yes	yes	yes	yes
Santa Ynez River Valley: Central	79	3-015	2022	yes	yes	yes	yes
Santa Ynez River Valley: East	80	3-015	2022	yes	yes	yes	yes

Basin Name	GSP ID	Basin Number	Year Submitted	Agriculture	Domestic	Environment	Disadvantaged Communities
Santa Ynez River Valley: West	78	3-015	2022	yes	yes	yes	yes
Scott River Valley	89	1-005	2022	yes	yes	yes	yes
Shasta Valley	90	1-004	2022	yes	yes	yes	yes
Sierra Valley	125	5-012.01	2022	yes	yes	yes	yes
Solano	117	5-021.66	2022	yes	yes	yes	yes
Sonoma Valley	101	2-002.02	2022	yes	yes	yes	yes
South American	111	5-021.65	2022	yes	yes	yes	yes
South Yuba	52	5-021.61	2020	yes	yes	yes	yes
Sutter	112	5-021.62	2022	yes	yes	yes	yes
Temescal	81	8-002.09	2022	yes	yes	yes	yes
Tracy	122	5-022.15	2022	yes	yes	yes	yes
Tulare Lake	42	5-022.12	2020	yes	yes	yes	yes
Tule: Alpaugh	48	5-022.13	2020	yes	yes	yes	yes
Tule: LTRID	56	5-022.13	2020	yes	yes	yes	yes
Tule: TCWA	57	5-022.13	2020	yes	yes	yes	yes
Tule: DEID	63	5-022.13	2020	yes	yes	yes	yes
Tule: ETGSA	43	5-022.13	2020	yes	yes	yes	yes
Tule: Pixley	65	5-022.13	2020	yes	yes	yes	yes
Tulelake	97	1-002.01	2022	yes	yes	yes	yes
Turlock	110	5-022.03	2022	yes	yes	yes	yes
Ukiah Valley	84	1-052	2022	yes	yes	yes	yes

Basin Name	GSP ID	Basin Number	Year Submitted	Agriculture	Domestic	Environment	Disadvantaged Communities
Upper San Luis Rey Valley	76	9-007.01	2022	yes	yes	yes	yes
Upper Valley Aquifer	115	3-004.05	2022	yes	yes	yes	yes
Upper Ventura River	77	4-003.01	2022	yes	yes	yes	no
Vina	86	5-021.57	2022	yes	yes	yes	yes
Westside	8	5-022.09	2020	yes	yes	yes	yes
White Wolf	123	5-022.18	2022	yes	yes	yes	yes
Wyandotte Creek	99	5-021.69	2022	yes	yes	yes	yes
Yolo	96	5-021.67	2022	yes	yes	yes	yes
Yucaipa	104	8-002.07	2022	yes	yes	yes	yes

Supplementary Table 2.3. Detailed determination of Stakeholder presence and absence (i.e., Not Applicable) across stakeholder groups and disadvantaged communities (DAC) sub-group by Sustainability Plan. Basin name is the name of the basin, using the Department of Water Resources official name. Sustainability Plan ID (GSP ID) is the Groundwater Sustainability Plan identification number in the Department of Water Resources Database. Basin Number is the groundwater basin identification number in the Department of Water Resources database. The Trigger for potential NA includes the rationale for examining whether a stakeholder group is present or absent. We assumed all stakeholder groups were present unless one of three things suggested their absence: 1) the Sustainability Plan described the stakeholder group as not applicable, 2) the Sustainability Plan scored zero across all integration components for the stakeholder in the rubric analysis, or 3) there were no wells or groundwater-dependent ecosystems present in the Sustainability Plan area in our spatial analysis. The Stakeholder column lists which stakeholder group is under consideration for presence or absence in the Sustainability Plan area. The Plan Text column lists the verbatim text from the Sustainability Plan regarding the stakeholder group and the Summary of Plan Text column summarizes whether the plan text suggests stakeholder presence or absence. The Total Number of Wells list the number of wells found in our spatial analysis in the Sustainability Plan area associated with the stakeholder group and the GIS Review summarizes our spatial analysis review results as to whether the stakeholder group is present or absent. Finally, the Final NA column lists our final NA determination for the stakeholder group in the specific Sustainability Plan.

Basin Name	GSP ID	Basin Number	Trigger for Potential NA	Stakeholder	Plan Text	Summary of Plan Text	Plan NA	Total # of Wells	GIS Review	Final NA
Delta-Mendota: Aliso	7	5-022.07	Described as NA in plan	Disadvantaged communities	"Disadvantaged Communities (DAC) – There are no disadvantaged communities within the AWD GSA. Nearby disadvantaged communities include Firebaugh and Mendota." Page 2-28 / 2.5.1, pdf pg. 63	Plan states that there are no disadvantaged communities in the Plan area.	NA		No DAC in jurisdiction	NA
East Bay Plain	91	2-009.04	Described as NA in plan	Agriculture	"Figure 2-2 depicts land use in the EBP Subbasin, which is classified primarily as urban (94%),	Plan makes no mention of current agricultural users.	NA	180	Satellite data suggest entire Plan is urban; well data indicate only a single	NO

Basin Name	GSP ID	Basin Number	Trigger for Potential NA	Stakeholder	Plan Text	Summary of Plan Text	Plan NA	Total # of Wells	GIS Review	Final NA
					with the remaining area classified as native vegetation, barren land, and water surface. Urban land uses include commercial, industrial, and residential. The vast majority of the Subbasin's land area is classified as medium- to high-intensity urban development." Pg 2-2, pdf page 59<br><br>Appendices describe the history of agriculture in the area, but urbanization has led to no agriculture in the plan boundaries.				ag well post 2000	
Elsinore Valley	119	8-004.01	all zero responses	Agriculture	"The overlying land uses have converted to primarily urban uses, however there are a	Plan states that there is agriculture in the plan area.	present	25	Satellite data suggest very little area that is not urban; some barren plots	present

Basin Name	GSP ID	Basin Number	Trigger for Potential NA	Stakeholder	Plan Text	Summary of Plan Text	Plan NA	Total # of Wells	GIS Review	Final NA
					limited number of larger parcels that still have agricultural production." Appendix F, Exhibit 8, pdf pg. 652 [From the Interested Parties List]				that appear to historically have been ag plots near ag wells, but area is barren	
Kern County: Henry Miller	37	5-022.14	Described as NA in plan	Disadvantaged communities	" Municipal and Domestic Supply (MUN) - Includes uses of water for one domestic well user for non-potable purposes. Community and military water supply systems including, but not limited to, drinking water supply are not present within the GSA. The one domestic well within HMWD is located at the HMWD office and is not used for drinking water purposes." Page 13 /	Plan states they have no domestic water supply in the Plan area.	NA		No domestic wells within jurisdiction	NA

Basin Name	GSP ID	Basin Number	Trigger for Potential NA	Stakeholder	Plan Text	Summary of Plan Text	Plan NA	Total # of Wells	GIS Review	Final NA
					1.5.5.1, pdf pg. 28					
Kern County: Henry Miller	37	5-022.14	Described as NA in plan	Domestic	"Municipal and Domestic Supply (MUN) - Includes uses of water for one domestic well user for non-potable purposes. Community and military water supply systems including, but not limited to, drinking water supply are not present within the GSA. The one domestic well within HMWD is located at the HMWD office and is not used for drinking water purposes." Page 13 / 1.5.5.1, pdf pg. 28	Plan states that there are no active domestic users in the Plan area.	NA	4	Four dom wells within the jurisdictional boundary; dom wells on the border and due to TSR locations, it is unclear if these wells are within the jurisdictional boundary; satellite data suggest only ag land, no residential plots	NA
Kern County: Olcese	44	5-022.14	all zero responses	Agriculture	"The primary land uses within the Olcese GSA Area, based on the Kern County historical crop	Plan states that there is agriculture in the plan area.	present	1	Satellite data suggest nearly entire jurisdiction is ag plots; only one ag	present

Basin Name	GSP ID	Basin Number	Trigger for Potential NA	Stakeholder	Plan Text	Summary of Plan Text	Plan NA	Total # of Wells	GIS Review	Final NA
					records for 2018, are shown on Figure PA-3. Approximately 1,196 acres within the Olcese GSA Area are irrigated and used for agricultural purposes. Of the irrigated area, 83% is used for cultivation of citrus (991 acres), 16% for cultivation of deciduous fruits and nuts (193 acres), and the remaining 1% for pasture (12 acres) (Figure PA-3). These lands are irrigated primarily by Kern River water, diverted pursuant to a combination of riparian water rights held by the Nickel Family, LLC and managed by the district and non-riparian				well within jurisdiction but jurisdiction is small	

Basin Name	GSP ID	Basin Number	Trigger for Potential NA	Stakeholder	Plan Text	Summary of Plan Text	Plan NA	Total # of Wells	GIS Review	Final NA
					rights held by the district. The district also pumps groundwater to supplement the Kern River surface water." Page 20/pdf page 31					
Kern County: Olcese	44	5-022.14	Described as NA in plan	Disadvantaged communities	"Disadvantaged Communities - There are no Disadvantaged Community Places, Tracts, or Block Groups identified within the Olcese GSA Area (U.S. Census, 2015)." Appendix C, page 8, pdf pg. 218	Plan states that there are no disadvantaged communities in the Plan area.	NA		No domestic wells within jurisdiction	NA
Kern County: Olcese	44	5-022.14	all zero responses	Domestic	"No active private domestic wells or commercial/industrial uses of groundwater exist within the Olcese GSA Area." Page 30 / 5.5.1, pdf pg. 41<br><br>"Domestic Well Owners -	Plan states that there are no active domestic wells in the Plan area	NA	2	Two wells within jurisdictional boundary; wells within rural ag areas; satellite data suggest residential areas; due to TSR location rather than	NA

Basin Name	GSP ID	Basin Number	Trigger for Potential NA	Stakeholder	Plan Text	Summary of Plan Text	Plan NA	Total # of Wells	GIS Review	Final NA
					According to well completion reports compiled by the DWR1, there are five domestic wells within Public Land Survey System (PLSS) sections that overlie, at least partially, the Olcese GSA Area. However, the extent of active wells is currently unknown. Based on District knowledge, there are no active domestic wells." Appendix C/pdf page 217				real gps location it is unclear whether dom well exists within or outside the jurisdictional boundary	
Kings South	26		all zero responses	Agriculture	"Table 2-1 shows the acreages of area for each land use classification with largest land use being Urban/Residenti al at 72%. The remaining 28% includes various agricultural uses, such as citrus and vineyards,	Plan states that there is agriculture in the plan area.	present	43	Satellite data suggest ag wells near some farm plots; few plots but the plots do appear to be actively farmed	present

Basin Name	GSP ID	Basin Number	Trigger for Potential NA	Stakeholder	Plan Text	Summary of Plan Text	Plan NA	Total # of Wells	GIS Review	Final NA
					and industrial." Page 2-1/pdf page 44					
Kings: South	26	5-022.08	all zero responses	Domestic	"Domestic well owners will have the opportunity to seek representation as an interested party on a committee of the GSA or through public participation." Page 2-20 / 2.5.1	Plan states that there are domestic wells in the Plan area.	present	61	Tens of dom wells within the jurisdictional boundary; dom wells primarily in suburban hotspots within rural ag areas	present
Las Posas Valley	18	4-008	No wells mapped in DAC census block group	DAC	"Disadvantaged Communities. The only Disadvantaged Community shown on the DWR mapping tool (DWR 2017) within the LPVB is within the City of Moorpark and is represented by the City, as discussed earlier in this section." 1.8.2 / pg. 1-34 / pdf pg. 64	Plan describes and maps DAC communities in the Plan Area	present		No wells mapped in DAC census block group	NA

Basin Name	GSP ID	Basin Number	Trigger for Potential NA	Stakeholder	Plan Text	Summary of Plan Text	Plan NA	Total # of Wells	GIS Review	Final NA
Las Posas Valley	18	4-008	all zero responses	Domestic	"Domestic Users. The majority of domestic groundwater users in the LPV are supplied water from a city, special district, or mutual water company. FCGMA maintains a database of well owners, including domestic well owners." Page 1-32/pdf page 62	Plan states that there are domestic wells in the Plan area.	present	26	Tens of dom wells within the jurisdictional boundary; dom wells primarily within areas with little development	present
Madera: GFWD	61	5-022.06	Described as NA in plan	DAC		Plan does not mention or map DACs.	present		There is a DAC and there are domestic wells within it	present
Madera: GFWD	61	5-022.06	Described as NA in plan	Environment	"Impacts on groundwater dependent ecosystems: There are not any known areas of ecosystems being dependent on groundwater within the boundary of the	Plan states that there are no groundwater-dependent ecosystems in the Plan area.	NA			NA

Basin Name	GSP ID	Basin Number	Trigger for Potential NA	Stakeholder	Plan Text	Summary of Plan Text	Plan NA	Total # of Wells	GIS Review	Final NA
					District." Page 2-12 / 2.1.4, pdf pg. 21					
Madera: GFWD	61	5-022.06	all zero responses	Domestic	"The predominant existing, and planned, beneficial use of groundwater and surface water in the GSP area is irrigated crop production. A small amount of groundwater is pumped for domestic use." Page 2-12 / 2.1.5, pdf pg. 21	Plan states that there are domestic wells in the Plan area.	present	7	Seven dom wells within jurisdictional boundary; wells within rural ag area; jurisdiction is small	present
Madera: New Stone	49	5-022.06	Described as NA in plan	DAC		Plan does not mention or map DACs.	NA		No DAC in jurisdiction	NA
Madera: New Stone	49	5-022.06	Described as NA in plan	Environment	"There are not any groundwater dependent ecosystems within the district. The depth of groundwater ranges from 50 to 110 feet below ground surface and there are not any interconnected	Plan states that there are no groundwater-dependent ecosystems in the Plan area.	NA	2		NA

Basin Name	GSP ID	Basin Number	Trigger for Potential NA	Stakeholder	Plan Text	Summary of Plan Text	Plan NA	Total # of Wells	GIS Review	Final NA
					surface water systems throughout NSWD." Page 2-18/pdf pave 38					
Madera: New Stone	49	5-022.06	all zero responses	Domestic	"The District is predominantly agriculture and consists of two landowners." Page ES-1/ pdf pg. 12<br><br>"Minimum thresholds for water quality have not been set for domestic users due to the lack of beneficial users for his water use." Page 4-14/pdf page 123<br><br>"The domestic wells are the shallowest in the district; meaning if levels decline enough to cause any wells to go dry, residential water users will be impacted first." Page 4-21/pdf page 130	Plan states that there are two landowners in the area and references existence of domestic wells with regard to undesirable results	present	2	Two dom wells within jurisdictionary boundary; wells within rural ag area; jurisdiction is small; satellite data suggest at least one residential plot; due to TSR location rather than real gps location it is unclear whether all dom wells exist within or outside the jurisdictional boundary	present

Basin Name	GSP ID	Basin Number	Trigger for Potential NA	Stakeholder	Plan Text	Summary of Plan Text	Plan NA	Total # of Wells	GIS Review	Final NA
Madera: RCWD	45	5-022.06	all zero responses	DAC		Plan does not mention or map DACs.	NA		No DAC in jurisdiction	NA
Monterey	128	3-004.10	No wells mapped in DAC census block group	DAC	"There are no Disadvantaged Community Places identified within the area4. Some of these disadvantaged community areas are missing income data and may include the student population from California State University Monterey Bay. These recognized disadvantaged communities are located within the urban areas of the City of Marina and receive water service from MCWD." pg. 2-5. pdf pg. 69	Plan describes and maps DAC communities in the Plan Area	present		No wells mapped in DAC census block group	NA
Mound	19	4-004.03	all zero responses	DAC	"Disadvantaged communities - There are no disadvantaged communities served by private	Plan states that they have DACs, but they are not served by domestic	NA		No domestic wells within jurisdiction	NA

Basin Name	GSP ID	Basin Number	Trigger for Potential NA	Stakeholder	Plan Text	Summary of Plan Text	Plan NA	Total # of Wells	GIS Review	Final NA
					domestic wells or small community water systems located within the Basin." Appendix D, sect 4.2, page 6 (pdf pg. 463)	wells or small community water systems.				
Mound	19	4-004.03	all zero responses	Domestic	"There are no active or recently active domestic wells in the Basin...There are currently no active domestic well users within the Basin or private water companies; drinking water supply within the Basin is provided exclusively by the City of Ventura." Page ES-iii/pdf page 5	Plan states that there are no domestic wells in the Plan area.	NA	1	Area surrounding single domestic well is suburban development ; the location of the domestic well co-exists with location of public supply; due to Township/section/range location rather than real gps location it is unclear whether this dom well exists within or outside the	NA

Basin Name	GSP ID	Basin Number	Trigger for Potential NA	Stakeholder	Plan Text	Summary of Plan Text	Plan NA	Total # of Wells	GIS Review	Final NA
									jurisdictional boundary	
North Yuba	53	5-021.60	all zero responses	DAC	"The City of Marysville is a GSA in the North Yuba Subbasin. In addition to fulfilling specific requirements, such as disadvantaged community (DAC) and stakeholder outreach, representatives from the City of Marysville participated in GSA and GSP meetings, contributing to the development of this GSP." Page 1-5 /pdf page 35	Plan does not mention or map DACs, but references GSAs as responsible for DAC representation and outreach.	present		There are multiple DACs; at least one has domestic wells within it; it is difficult to tell if the other DACs have domestic wells given the data are only accurate the TSR	present
Ojai Valley	130	4-002	Described as NA in plan	DAC	"Based on 2016 DAC mapping at the Census Block Group level, approximately 1,220 acres of the OVGB are	Plan states that there are no DACs in the Plan area. States that regions previously listed as	NA		No DAC in jurisdiction	NA

Basin Name	GSP ID	Basin Number	Trigger for Potential NA	Stakeholder	Plan Text	Summary of Plan Text	Plan NA	Total # of Wells	GIS Review	Final NA
					identified as severely disadvantaged with a median household income of $26,250 per year, and 640 acres are disadvantaged with a median household income of $50,200 per year (DWR 2020b). More recent 2018 DAC mapping at the Census Block Group Level indicates the areas of the OVGB previously identified as disadvantaged are no longer designated as disadvantaged (DWR 2020b)." Page 2-41 / 2.1.3.1	DACs were delisted in most recent DAC mapping.				
Oxnard	16	4-004.02	all zero responses	DAC	"Disadvantaged Communities. The majority of the Disadvantaged Communities	Plan states that there are DACs in the Plan area.	present		There are multiple DACs, and they have domestic	present

Basin Name	GSP ID	Basin Number	Trigger for Potential NA	Stakeholder	Plan Text	Summary of Plan Text	Plan NA	Total # of Wells	GIS Review	Final NA
					(DACs) within the Oxnard Plain receive water from cities, special districts, or mutual water companies." Page 1-49 / 1.8.2, pdf pg. 75				wells within them.	
Oxnard	16	4-004.02	all zero responses	Domestic	"Domestic Users. The majority of domestic groundwater users in the Subbasin are supplied water from a city, special district, or mutual water company. FCGMA maintains a database of well owners, including domestic well owners." Page 1-48/pdf page 74	Plan states that there are domestic wells in the Plan area.	present	26	Tens of dom wells within the jurisdictional boundary; dom wells primarily within rural ag areas of the jurisdiction	present
Pleasant Valley	17	4-006	No wells mapped in DAC census block group	DAC	"Disadvantaged Communities. The only Disadvantaged Communities shown on the DWR mapping tool (DWR	Plan describes and maps DAC communities in the Plan Area	present		No wells mapped in DAC census block group	NA

Basin Name	GSP ID	Basin Number	Trigger for Potential NA	Stakeholder	Plan Text	Summary of Plan Text	Plan NA	Total # of Wells	GIS Review	Final NA
					2017) within the PVB is within the City of Camarillo and is represented by the City as discussed earlier in this section." Page 1-39 / 1.8.2, pdf pg. 63					
Pleasant Valley	17	4-006	all zero responses	Domestic	"Domestic Users. The majority of domestic groundwater users in the PVB are supplied water by a city, special district, or mutual water company. FCGMA maintains a database of well owners, including domestic well owners. Email addresses within the database have been added to the list of interested parties who receive electronic newsletters regarding the status and	Plan states that there are domestic wells in the Plan area.	present	9	Nine wells within jurisdictional boundary; most wells within rural ag areas	present

Basin Name	GSP ID	Basin Number	Trigger for Potential NA	Stakeholder	Plan Text	Summary of Plan Text	Plan NA	Total # of Wells	GIS Review	Final NA
					development of the PVB GSP." Page 1-38 pdf/ page 62					
San Antonio Creek Valley	87	3-014	Described as NA in plan	DAC	"No disadvantaged communities (DACs) were identified within the Basin, based on several datasets (refer to the IRWMP (Dudek, 2019); California Air Resources Board's (CARB) California Climate Investments (CCI) Priority Populations online mapping tool7; California Office of Environmental Health Hazard Assessment's CalEnviroScreen online mapping tool of Senate Bill 535 DACs8; and DWR's DACs online mapping tool)." 2.2.2.6 / pg. 2-10 / pdf pg. 63	Plan states that there are no DACs in the Plan area.	present		There is a DAC and there are domestic wells within it	present

Basin Name	GSP ID	Basin Number	Trigger for Potential NA	Stakeholder	Plan Text	Summary of Plan Text	Plan NA	Total # of Wells	GIS Review	Final NA
San Gorgonio Pass	121	7-021.04	all zero responses	Agriculture			NA	NA		NA
San Pasqual Valley	75	9-010	Described as NA in plan	DAC	"According to the DWR's Disadvantaged Communities mapping tool (DWR, 2021), there are no Disadvantaged Communities located within the Basin. Although there are no tribal reservation lands in the Basin, the San Pasqual Tribe has cultural interests in the Basin." Page 1-6 / 1.4.3, pdf pg. 32	Plan states that there are no DACs in the Plan area.	NA		No DAC in jurisdiction	NA
Santa Margarita	74	3-027	DAC shapefile indicated "no data available"	DAC			NA		This Sustainability Plan shows an area that indicates "no data available" as a DAC, and there appears to be domestic wells within the DAC	present

Basin Name	GSP ID	Basin Number	Trigger for Potential NA	Stakeholder	Plan Text	Summary of Plan Text	Plan NA	Total # of Wells	GIS Review	Final NA
Santa Cruz Mid-County	11	3-001	No wells mapped in DAC census block group	DAC	"Disadvantaged Communities (DAC) – Data from DWR's DAC mapping tool identifies seven DACs, including one severely disadvantaged community within the Basin; all seven DACs are located within the City of Santa Cruz water supply service area (Figure 2-11)." Page 2-5 / 2.1.5.1.1, pdf pg. 102	Plan describes and maps DAC communities in the Plan Area	present		No wells mapped in DAC census block group	NA
Santa Monica	129	4-011.01	all zero responses	DAC	" DACs within the City of Santa Monica are the only DACs in the Subbasin that receive local groundwater produced within the Subbasin. DACs within the Subbasin that are outside the jurisdiction of the City of Santa Monica's Department of	Plan states that they have DACs served by local groundwater	present		No domestic wells within jurisdiction	NA

Basin Name	GSP ID	Basin Number	Trigger for Potential NA	Stakeholder	Plan Text	Summary of Plan Text	Plan NA	Total # of Wells	GIS Review	Final NA
					Public Works (i.e., within the City of Los Angeles and County of Los Angeles) receive imported water, not local groundwater, through their respective water supplier..." Page 2-46 / 2.1.5.1					
Santa Monica	129	4-011.01	all zero responses	Domestic	"Currently, the City of Santa Monica produces the majority of the groundwater in the Subbasin and is the sole producer of drinking water in the Subbasin. There are no domestic users of groundwater in the Subbasin." Page 2-94 / 2.5.2.1	Plan states that there are no domestic wells in the Plan area.	NA	1	Area surrounding domestic wells is suburban development	NA
Santa Monica	129	4-011.01	Described as NA in plan	Agriculture	“Municipal and industrial water demand constitutes 97% of the total water demand in MWD’s service	Plan states there are no agricultural users in the plan area.	NA	10	Satellite data suggest ag wells near parks or golf courses, rather than ag land	NA

Basin Name	GSP ID	Basin Number	Trigger for Potential NA	Stakeholder	Plan Text	Summary of Plan Text	Plan NA	Total # of Wells	GIS Review	Final NA
					area, and agricultural demand constitutes the remaining 3% of water demand. Due to urbanization and the increasing price of water, the share of agricultural demand has decreased over the past 50 years (MWD 2021). There are no agricultural users of water in the Plan Area." Page 2-6 / 2.1.1.2.1, pdf pg. 68					
Santa Ynez River Valley: Central	79	3-015	Described as NA in plan	DAC	"Disadvantaged Communities: There currently are no areas within the CMA GSA that are mapped as Disadvantaged Communities. Areas within the COB have been mapped as Disadvantaged Communities in the past and are	Plan states that there are not DACs in the Plan area, but areas that used to be considered DACs and are no longer.	present		There is a DAC and there are domestic wells within it	present

Basin Name	GSP ID	Basin Number	Trigger for Potential NA	Stakeholder	Plan Text	Summary of Plan Text	Plan NA	Total # of Wells	GIS Review	Final NA
					represented on the CMA GSA by the COB." App B of App 1c-A, pdf pg. 712					
Santa Ynez River Valley: East	80	3-015	all zero responses	DAC	"Based on several datasets, no disadvantaged communities were identified within the EMA (refer to the IRWM Plan [Dudek, 2019]; 2020 California Air Resources Board and 2018 California Climate Investments Priority Populations online maps13; and DWR's DAC mapping data from 2018 at the places and tract scales14)." Page 2-31&2-32/pdf pages 87-88<br><br>"Disadvantaged Communities - There are several	Plan states in the body of the Plan that there are not DACs in the Eastern Management Area, but in the appendix states that there are DACs in the Eastern Management Plan area.	present		There is a DAC block group in the south and there are domestic wells within the block group	present

Basin Name	GSP ID	Basin Number	Trigger for Potential NA	Stakeholder	Plan Text	Summary of Plan Text	Plan NA	Total # of Wells	GIS Review	Final NA
					disadvantaged communities within the EMA portion of the Basin based on block groups, census tracts, and census places. These areas are in the southeast portion of the EMA, as shown on Figure 3 below. " Appendix B, pdf pg. 515					
South Yuba	52	5-021.61	all zero responses	DAC	"The City of Marysville is a GSA in the North Yuba Subbasin. In addition to fulfilling specific requirements, such as disadvantaged community (DAC) and stakeholder outreach, representatives from the City of Marysville participated in GSA and GSP meetings, contributing to	Plan does not mention or map DACs, but references GSAs as responsible for DAC representation and outreach.<br><br>[same plan as North Yuba]	present		There are multiple DACs, and they have domestic wells within them.	present

Basin Name	GSP ID	Basin Number	Trigger for Potential NA	Stakeholder	Plan Text	Summary of Plan Text	Plan NA	Total # of Wells	GIS Review	Final NA
					the development of this GSP." Page 1-5 /pdf page 35					
Tulare Lake	42	5-022.12	all zero responses	Domestic	"There are domestic wells within the SFKGSA, and it is understood that many domestic users will fall into the “de minimis extractor” category. Further work is being conducted to understand to what extent domestic users will be affected by GSP requirements." Appendix B-8/pdf pg. 425	Plan states that there are domestic wells in the Plan area.	present	1370	Hundreds of dom wells within the jurisdictional boundary; dom wells primarily within rural areas	present
Tule: Alpaugh	48	5-022.13	all zero responses	Domestic	"Domestic Well Owners - Domestic wells within Alpaugh GSA are operated in agricultural areas outside of the Alpaugh Community, which is served	Plan states that there are domestic wells in the Plan area.	present	5	Five wells within the jurisdiction, primarily within rural areas	present

Basin Name	GSP ID	Basin Number	Trigger for Potential NA	Stakeholder	Plan Text	Summary of Plan Text	Plan NA	Total # of Wells	GIS Review	Final NA
					by ACSD. The SGMA excludes “de minimis extractors” from its requirements; at this time, Alpaugh GSA is also excluding all domestic wells from GSP requirements." Page 27 / 1.4.1.2, pdf pg. 27					
Tule: DEID	63	5-022.13	all zero responses	Domestic	"Domestic Well Owners – These are farmsteads located throughout the DEID GSA that are served by small domestic wells. In most cases they are also agricultural users and are considered by the DEID GSA through pre-existing relationships." Appendix 1-F, page II-2, pdf pg. 99	Plan states that there are domestic wells in the Plan area.	present	38	Tens of dom wells within the jurisdictional boundary; dom wells primarily within rural areas	present
Tule: ETGSA	43	5-022.13	all zero responses	Domestic	"Domestic well owners - Domestic well	Plan states that there are domestic	present	711	Hundreds of dom wells within the	present

Basin Name	GSP ID	Basin Number	Trigger for Potential NA	Stakeholder	Plan Text	Summary of Plan Text	Plan NA	Total # of Wells	GIS Review	Final NA
					owners are located primarily in East Porterville, around the edges of City of Porterville's service area, and across ETGSA in farmsteads and ranches." Appendix 8A, page 17, pdf pg. 339	wells in the Plan area.			jurisdictional boundary; dom wells primarily within rural areas	
Tule: TCWA	57	5-022.13	all zero responses	Domestic	"There is a small number of domestic wells that serve farmsteads and private homes and dairies, and there is a small rural community west of the town of Earlimart that has 1.5 – 2.5 acre-parcels that are supplied by private wells." ES-3/pdf page 17	Plan states that there are domestic wells in the Plan area.	present	35	Tens of dom wells within the jurisdictional boundary; dom wells primarily within rural areas	present
Upper Ventura River	77	4-003.01	No wells mapped in DAC census block group	DAC	"Disadvantaged communities, including, but not limited to, those served by private domestic	Plan describes and maps DAC communities in the Plan Area	present		No wells mapped in DAC census block group	NA

Basin Name	GSP ID	Basin Number	Trigger for Potential NA	Stakeholder	Plan Text	Summary of Plan Text	Plan NA	Total # of Wells	GIS Review	Final NA
					wells or small community water systems. The community of Casitas Springs is recognized as a disadvantaged community. The community is served by Casitas Mutual Water Company, Ventura River Water District, and Casitas Municipal Water District, the latter two being signatory members to the JPA Agreement forming the Agency. Thus, the community is represented on the Agency's Board of Directors." Appendix E / pg. 8 / pdf pg. 838"					

Supplementary Table 2.4. Integration component scores and aggregate score comparisons for stakeholder groups and sub-groups using Kruskal-Wallis, Dunn, and Mann-Whitney Wilcoxon Rank-Sum tests. Significant thresholds are denoted as * ($p < 0.05$), ** ($p < 0.01$), *** ($p < 0.001$).

Comparisons	Test (statistic)	***Engage* Score**	***Describe* Score**	***Analyze* Score**	***Act* Score**	***Aggregate* Score**
Agriculture, Domestic and Environment group-wise comparison	Kruskal-Wallis (χ^2 statistic)	19.68***	94.71***	18.18***	30.49***	10.83**
Agriculture - Domestic pairwise comparison	Post-Hoc Dunn (z score)	4.34***	-3.02**	-3.10**	5.33***	0.98
Agriculture - Environment pairwise comparison	Post-Hoc Dunn (z score)	1.38	-9.52***	0.99	1.42	-2.23*
Domestic - Environment pairwise comparison	Post-Hoc Dunn (z score)	-2.97**	-6.47***	4.09***	-3.93***	-3.21**
Agriculture - Small Farms pairwise	Mann-Whitney Wilcoxon Rank-Sum (W score)	8312***	9201***	8564.5***	9917.5***	10767.5***
Domestic - Disadvantaged Communities pairwise comparison	Mann-Whitney Wilcoxon Rank-Sum (W score)	5211	3451***	7930***	5665.5*	6111**

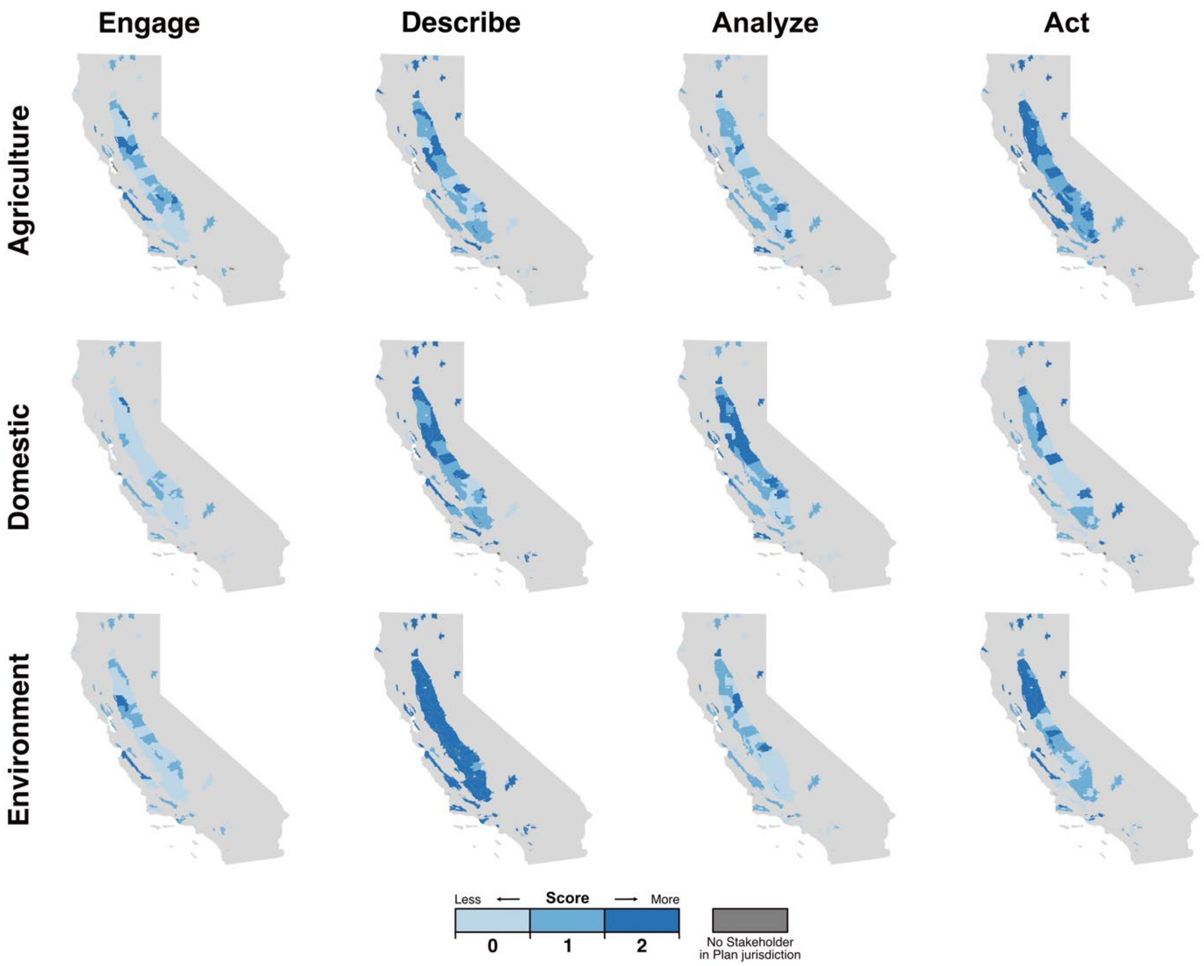

Supplementary Fig. 2.1. Agriculture, Domestic, and Environment stakeholder integration into Sustainability Plans mapped by geographical plan area in California.

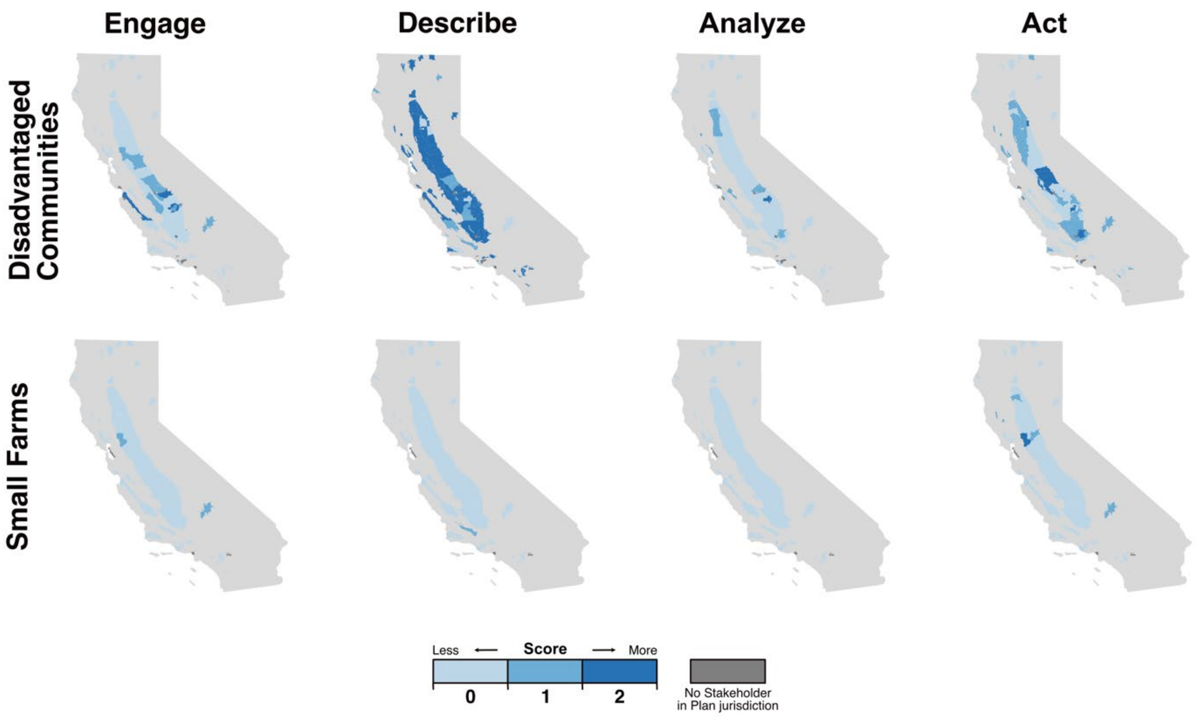

Supplementary Fig. 2.2. Disadvantaged Community (DAC) and Small Farm stakeholder integration into Sustainability Plans mapped by geographical plan area in California.

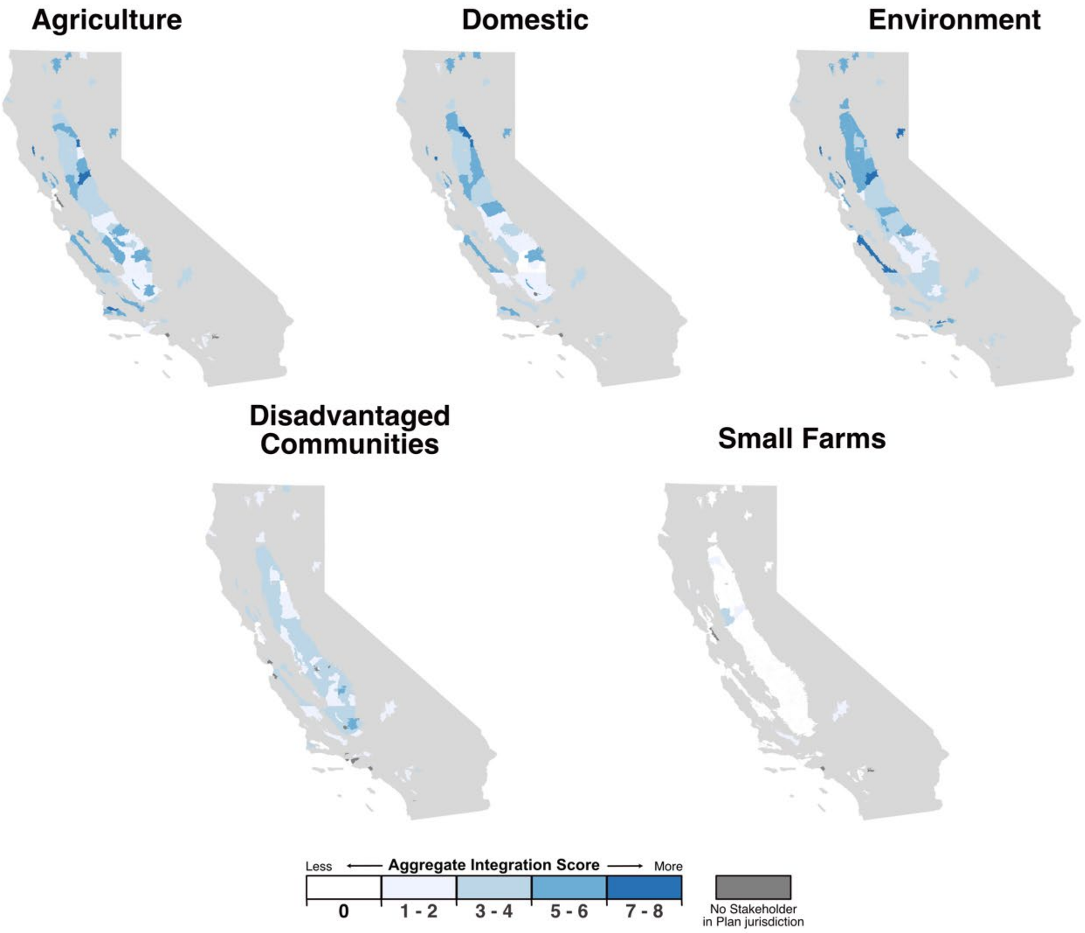

Supplementary Fig. 2.3. Stakeholder aggregate integration score for all stakeholders mapped by geographical plan area in California.

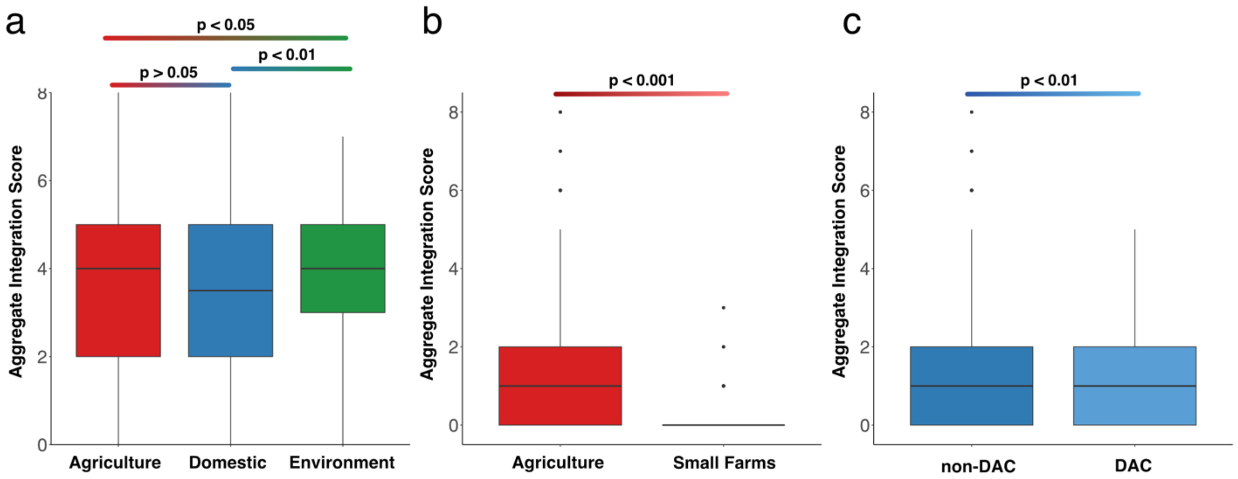

Supplementary Fig. 2.4. Stakeholder aggregate integration score comparisons for a) agricultural domestic and environment groups showing pair-wise Dunn test significance, b) agriculture and small farms sub-group comparison showing Mann-Whitney Wilcoxon Rank-Sum test significance, and c) domestic and disadvantaged communities (DAC) farms sub-group comparison showing Mann-Whitney Wilcoxon Rank-Sum test significance.

Supplementary Section 3. Sensitivity Analysis for Agriculture *Engage* Component

Under California's Sustainable Groundwater Management Act, water utilities that provide irrigation water for agriculture, including irrigation and reclamation districts, are able to form Groundwater Sustainability Agencies to write Sustainability Plans, or collaborate with other Groundwater Sustainability Agencies to write a Sustainability Plan. Agriculture is the only stakeholder group, of the main three stakeholder groups we analyze, in which stakeholders, e.g., those farmers served by an irrigation or reclamation district, have the ability to form a Groundwater Sustainability Agency and represent themselves in the implementation of the Sustainable Groundwater Management Act. There is no similar organization for environment or domestic well users that can form a Groundwater Sustainability Agency pursuant to California's Sustainable Groundwater Management Act (CA Water Code § 10723). This likely creates differential access for each of the three stakeholder groups to access decision-making on a groundwater basin's Sustainability Plan.

The standard coding of the *engage* integration component in our analysis did not include this type of representation on a Groundwater Sustainability Agency board. As described in the beginning of Supplementary Section 2: *Operationalizing stakeholder integration*, and in Supplementary Table 2.1, the standard *engage* component only considered the presence of stakeholder-specific invited seats on the board, or in other words, a designated seat on the board for an agricultural stakeholder. We focus on these invited spaces as they are areas for stakeholders to voice needs, preferences and perspectives that may not be represented by a governing authority – here the governing authorities are the Groundwater Sustainability Agency(s). As such, a stakeholder designated seat represents a priority of the Groundwater Sustainability Agency(s) involved in Sustainability Plan development. Additionally, this provides for a balanced comparison between the stakeholder groups: agricultural, domestic, and environmental users could all be invited to participate in Sustainability Plan decision making with the inclusion of a designated stakeholder voting seat on the board for the respective group.

For agriculture, we differentiated between stakeholder-invited seats and the presence of an irrigation or reclamation district on the board, because we saw this as confounding different types of power. We employ the Power Cube framework[26] to define the 'spaces' of power that stakeholders have access to, which describe the "opportunities, moments, and channels where citizens can act to potentially affect policies, discourses, decisions and relationships that affect their lives and interests" (pg. 26). We differentiate between each of the stakeholder group's access to 'invited' and 'closed' spaces. The stakeholder designated seat represents an 'invited' form of participation, which is not a form of power that comes from the stakeholder group, but rather originates from the Groundwater Sustainability Agency(s) involved in Sustainability Plan development. According to the Power Cube framework, 'invited' spaces are those in which "people (as users, citizens or beneficiaries) are invited to participate by various kinds of authorities" (pg. 26).

Alternatively, the presence of an irrigation or reclamation district acting as, or part of, a Groundwater Sustainability Agency developing a Sustainability Plan represents a form of formal governance authority, or power that can originate within the stakeholder group. An irrigation or reclamation district self-determines their participation in the Sustainability Plan development process. This aligns with the definition of a 'closed' space[26], one in which "decisions are made by a set of actors behind closed doors, without any pretense of broadening the boundaries for inclusion" (pg. 26). Groundwater Sustainability Agency decision making is not 'closed' in the strictest sense: as a public entity, the Groundwater Sustainability Agencies are required to host public meetings and take public comments. Nevertheless, they are 'closed' in that only entities defined in the Sustainable Groundwater Management Act legislation

have the authority to become Groundwater Sustainability Agencies and utilize the governing powers vested in them to write and implement groundwater sustainability plans. Only agricultural stakeholders, and those that are part of a reclamation or irrigation district, have access to the “closed” space of Groundwater Sustainability Agencies formation and decision making.

Irrigation and reclamation districts are entities that provide water primarily for agricultural use, as compared to more general purpose entities like cities or counties that manage water, land, and other domains. While other entities that manage water in California, such as California Water Districts, also represent ‘closed’ decision making spaces, these entities typically manage water supply for many different uses (i.e., agricultural, domestic, industrial, municipal). Therefore, we focus on irrigation and reclamation districts in our sensitivity analysis as they represent a special case in which primarily agricultural water users have access to the ‘closed’ space of Groundwater Sustainability Agency decision making in the Sustainable Groundwater Management Act implementation.

To explore the role of these different types of power for agricultural stakeholders, we ran a sensitivity analysis using two variations of the *engage* integration component for agriculture, defined in Supplementary Table 3.1. *Engage2* considers only the presence of a Groundwater Sustainability Agency specific to agriculture on the board, including irrigation and reclamation districts. E*ngage3* includes both the presence of a Groundwater Sustainability Agency and the presence of an invited, designated seat for an agricultural stakeholder. In our sensitivity analysis, we provide additional versions of the analyses that include the standard *engage* integration component and provide comparisons of the outputs for the three different types of *engage* (i.e., *engage*, *engage2, engage3*).

Supplementary Table 3.1. Variations in the coding of the *engage* integration component for agricultural users to test for the influence of agricultural representation via agriculture-specific Groundwater Sustainability Agencies on Sustainability Plan voting boards.

Component	Definition	Distinction from standard Engage component	Question	Response Levels
engage2: engage via Groundwater Sustainability Agency representation on Plan board (agriculture only)	Stakeholder participation in Plan deliberation or decision-making	Engagement of agricultural stakeholders in Plan voting is coded as the presence of a Groundwater Sustainability Agency that explicitly represents agriculture, including irrigation and reclamation districts. Invited stakeholder-specific seats for agriculture are not included. Agricultural presence on advisory boards is coded the same as the standard *engage*.	Does the Sustainability Plan document opportunities for 1) explicit stakeholder involved in consistent, broad-ranging discussion and two-way engagement on Sustainability Plan content (e.g., citizen advisory committees and/or working groups), and/or 2) presence of an explicit agriculturally oriented Groundwater Sustainability Agency on the Plan voting board of a (i.e., an irrigation district or a reclamation district).	NA<br>0. No<br>1. Only one<br>2. Both
engage3: engage via [Groundwater Sustainability Agency representation or stakeholder-specific seat] or on Plan board (agriculture only)	Stakeholder participation in Plan deliberation or decision-making	Engagement of agricultural stakeholders in Plan voting is coded as the presence of a Groundwater Sustainability Agency that explicitly represents agriculture, including irrigation and reclamation districts OR invited stakeholder-specific seats for agriculture. The presence of either of these types of representation was coded the same: as the presence of agriculture in Plan voting. Agricultural presence on advisory boards is coded the same as the standard *engage*.	Does the Sustainability Plan document opportunities for 1) explicit stakeholder involved in consistent, broad-ranging discussion and two-way engagement on Sustainability Plan content (e.g., citizen advisory committees and/or working groups), and/or 2) presence of an explicit agriculturally-oriented GSA on the Plan voting board of a (i.e. an irrigation district or a reclamation district) OR explicit stakeholder involved in voting on Plan related process and outputs (e.g., stakeholder seat on the Sustainability Plan board)?	NA<br>0. No<br>1. Only one<br>2. Both

Supplementary Table 3.2. Comparison of the three variations in *engage* for agriculture, including the standard *engage*, *engage2* score and *engage3*. Tests are Kruskal-Wallis, Dunn, and Mann-Whitney Wilcoxon Rank-Sum tests. Significant thresholds are denoted as * ($p < 0.05$), ** ($p < 0.01$), *** ($p < 0.001$).

**Comparisons for variation in agriculture *engage* scores**	***Engage* Test Statistic**	
Standard *engage*, *engage2, engage3*	Kruskal-Wallis (χ^2 statistic)	10.54**
Standard *engage* - *engage2* pairwise comparison	Post-Hoc Dunn (z score)	-1.85
Standard *engage* - *engage3* pairwise comparison	Post-Hoc Dunn (z score)	-3.24**
Engage2 – *engage3* pairwise comparison	Post-Hoc Dunn (z score)	-1.39

Supplementary Table 3.3. Comparison of variations in *engage* and *aggregate* for agriculture, including *engage2* score and *engage3* score (Agriculture2 and Agriculture3, respectively), with the standard *engage* and *aggregate* scores for environment and domestic stakeholder groups. The only change for the *aggregate2* score and *aggregate3* score for agriculture is the inclusion of *engage2* and *engage3,* respectively, instead of the standard *engage* score for agriculture. As such, the *aggregate* variations for agriculture are a sum of the scores for the respective *engage* variation with the standard *describe, analyze*, and *act* scores for agriculture. Tests are Kruskal-Wallis, Dunn, and Mann-Whitney Wilcoxon Rank-Sum tests. Significant thresholds are denoted as * (p < 0.05), ** (p < 0.01), *** (p < 0.001).

**Comparisons**	**Test (statistic)**	***Engage* Score**	***Aggregate* Score**
Agriculture2, Domestic and Environment group-wise comparison	Kruskal-Wallis (χ^2 statistic)	42.90***	9.85**
Agriculture2 - Domestic pairwise comparison	Post-Hoc Dunn (z score)	6.54***	1.73
Agriculture2 - Environment pairwise comparison	Post-Hoc Dunn (z score)	3.61***	-1.40
Domestic - Environment pairwise comparison	Post-Hoc Dunn (z score)	-2.96**	-3.13**
Agriculture3, Domestic and Environment group-wise comparison	Kruskal-Wallis (χ^2 statistic)	55.65***	10.30**
Agriculture3 - Domestic pairwise comparison	Post-Hoc Dunn (z score)	7.39***	2.23*
Agriculture3 - Environment pairwise comparison	Post-Hoc Dunn (z score)	4.60***	-0.89
Domestic - Environment pair-wise comparison	Post-Hoc Dunn (z score)	-2.81**	-3.12**

Supplementary Table 3.4. Comparison of the three variations in *aggregate* for agriculture, including the standard *engage*, *engage2* score and *engage3*. The only change for the *aggregate2* score and *aggregate3* score for agriculture is the inclusion of *engage2* and *engage3,* respectively, instead of the standard *engage* score for agriculture. As such, the *aggregate* variations for agriculture are a sum of the scores for the respective *engage* variation with the standard *describe, analyze*, and *act* scores for agriculture. Tests are Kruskal-Wallis, Dunn, and Mann-Whitney Wilcoxon Rank-Sum tests. Significant thresholds are denoted as * ($p < 0.05$), ** ($p < 0.01$), *** ($p < 0.001$).

**Comparisons**	***Aggregate* Test Statistic**	
Aggregate - Aggregate2 - Aggregate3 groupwise	Kruskal-Wallis (χ^2 statistic)	1.72
Aggregate - *Aggregate*2 pairwise comparison	Post-Hoc Dunn (z score)	-0.78
Aggregate - *Aggregate*3 pairwise comparison	Post-Hoc Dunn (z score)	-1.30
Aggregate2 – *Aggregate*3 pairwise comparison	Post-Hoc Dunn (z score)	-0.52

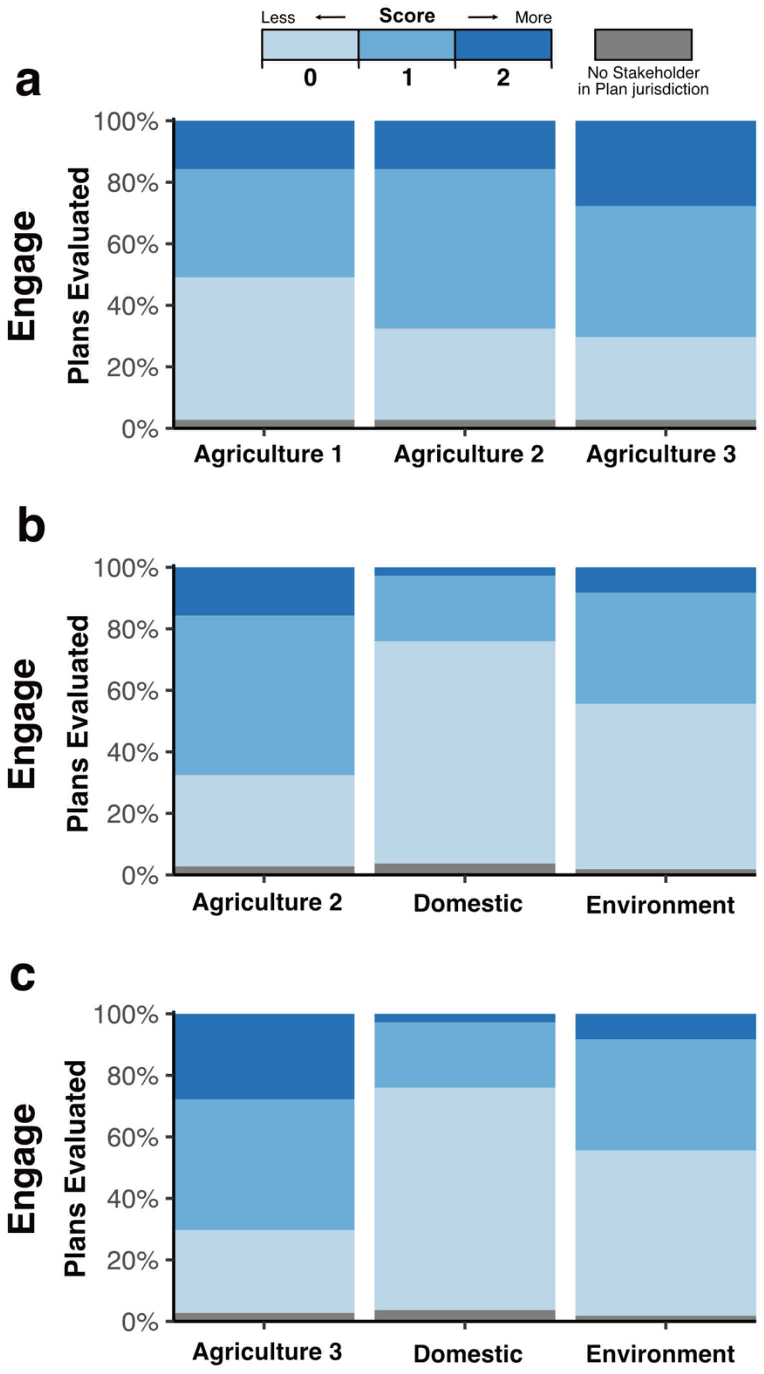

Supplementary Fig. 3.1. **a.** Bar plot comparison of the three variations of coding for the *engage* component. **b.** Comparison of variation *engage2* score for agriculture with standard stakeholder *engage* scores for domestic and environment. **c.** Comparison of variation *engage3* score for agriculture with standard Stakeholder *engage* scores for domestic and environment.

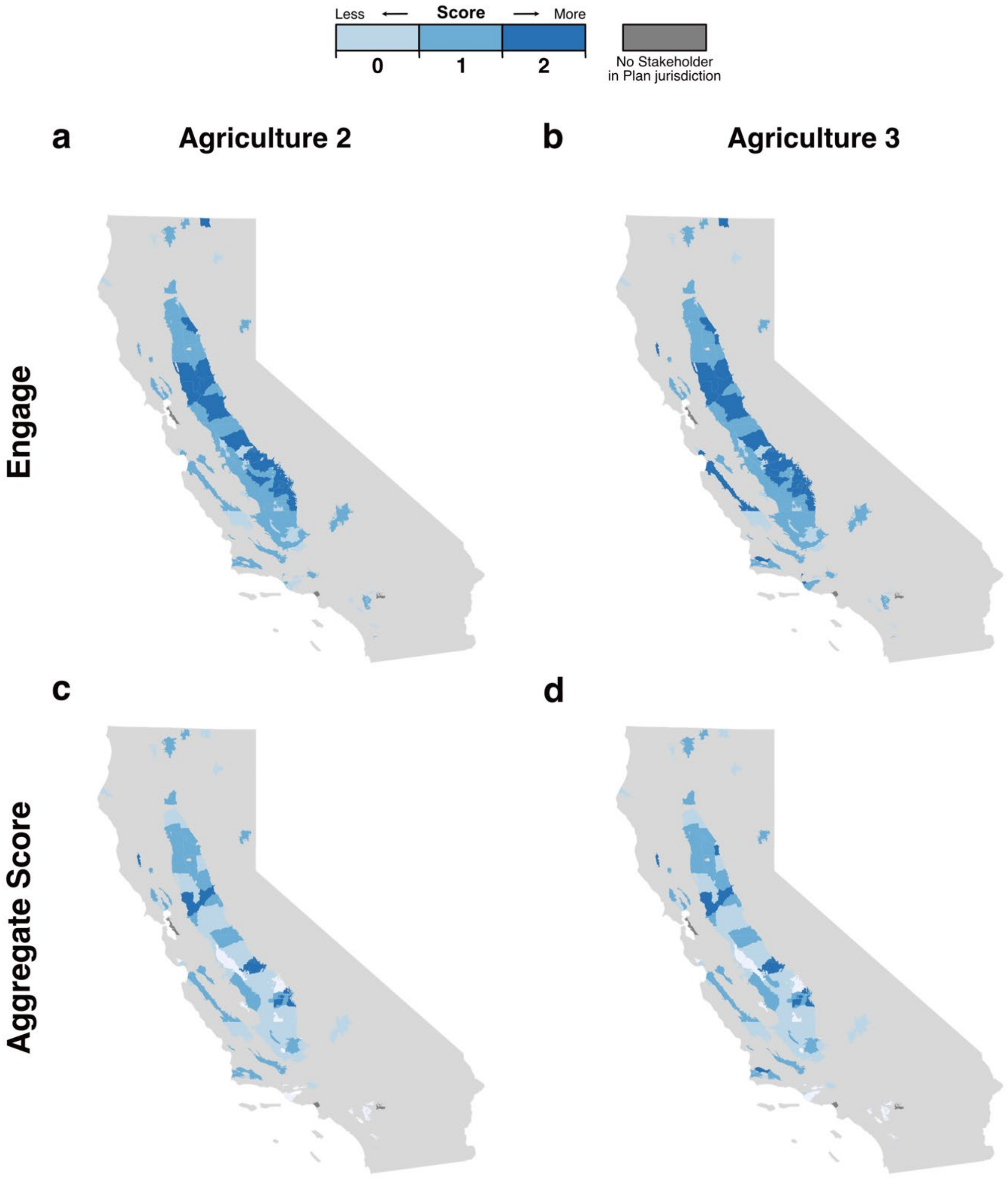

Supplementary Fig. 3.2. *Engage* score and *aggregate* score by Sustainability Plans based on the *engage2* score and *engage3* variations for agriculture (Agriculture 2 and Agriculture 3, respectively). Note the aggregate scores include the specified *engage* variation with the standard agricultural integration components for *describe*, *analyze*, and *act*.

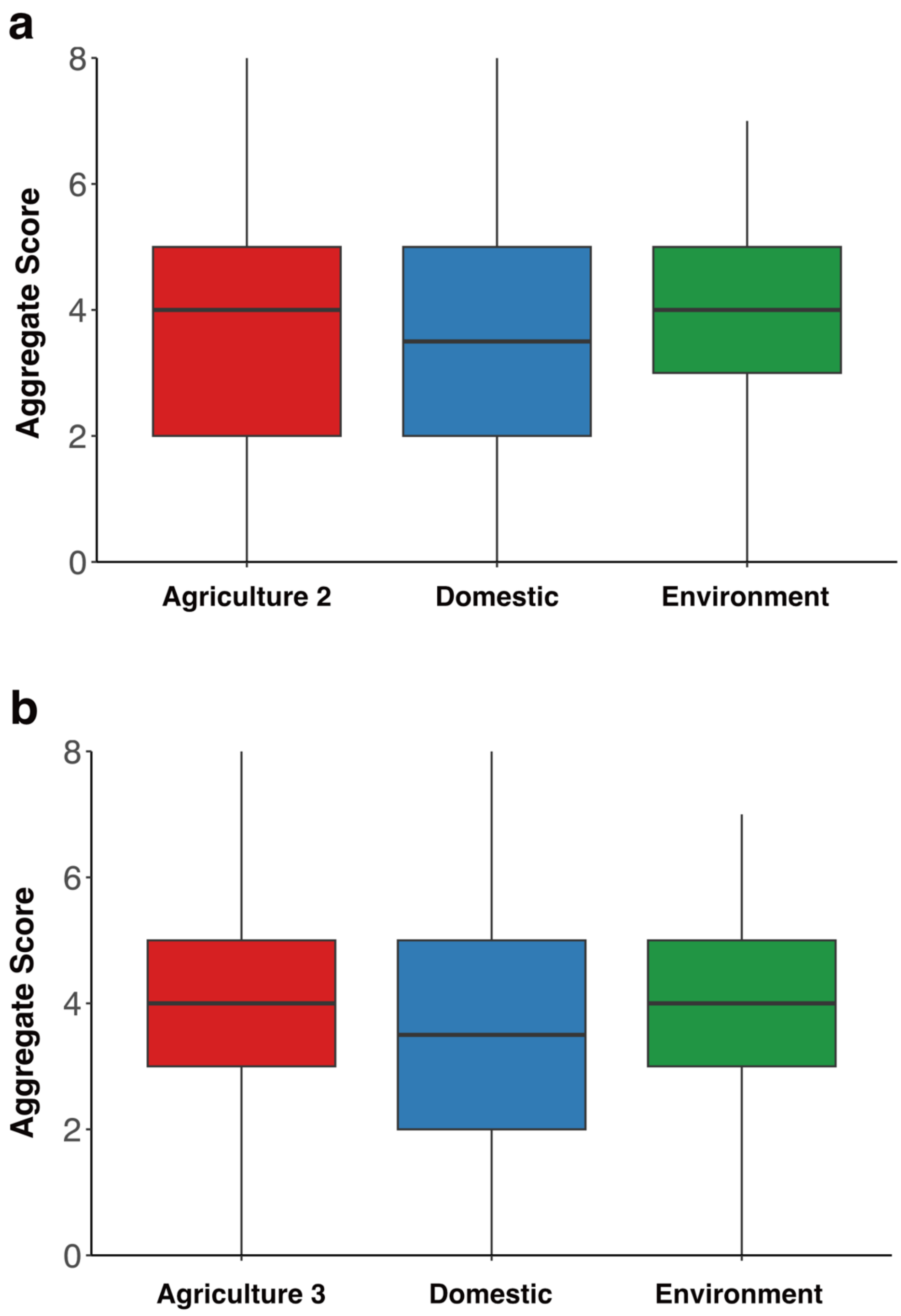

Supplementary Fig. 3.3. a. Box plot comparison of variation *aggregate agriculture2* score for agriculture with standard stakeholder *aggregate* scores for domestic and environment. The only change for the *aggregate agriculture2* score for agriculture is the inclusion of *engage agriculture2* instead of the standard *engage* score for agriculture. As such, the agriculture *aggregate2* score is a sum of the scores agriculture *engage2* and the standard *describe, analyze*, and *act* scores for agriculture. **b.** Box plot comparison of variation agriculture *aggregate3* score for agriculture with standard stakeholder *aggregate* scores for domestic and environment. The only change for the *aggregate3* score for agriculture is the inclusion of *engage3* instead of the standard *engage* score for agriculture.

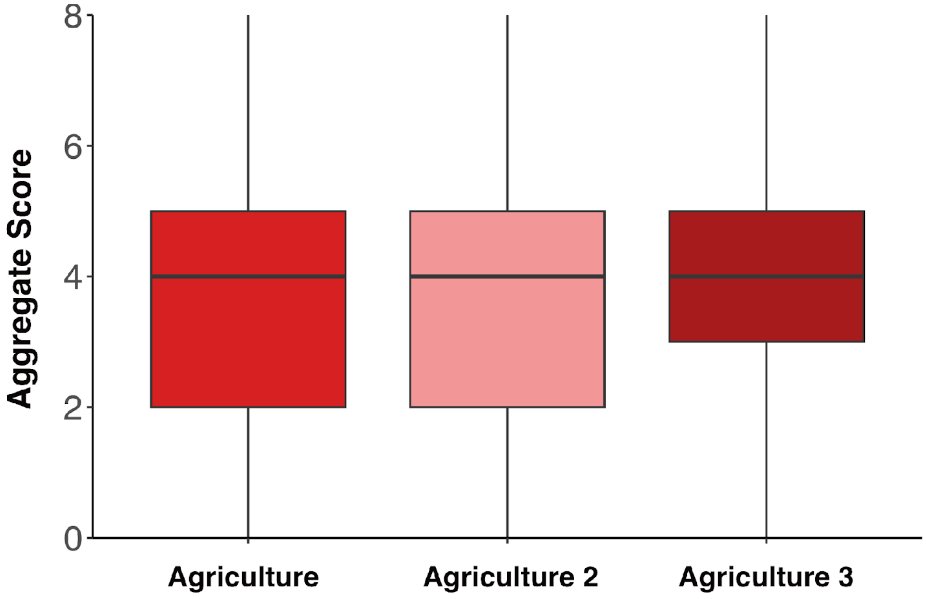

Supplementary Fig. 3.4. Boxplot Comparison of the three variations in *aggregate* for agriculture, including the standard *engage*, *engage2* score and *engage3*. The only change for the *aggregate2* score and *aggregate3* score for agriculture is the inclusion of *engage2* and *engage3,* respectively, instead of the standard *engage* score for agriculture. As such, the *aggregate* variations for agriculture are a sum of the scores for the respective *engage* variation with the standard *describe, analyze*, and *act* scores for agriculture.

Supplementary Section 4. Cover and Protection Analysis

Cover and Protection Sensitivity Analysis

We selected a range of buffer distances, between 0.8 km (0.5 mi) and 4.0 km (2.5 mi), that are generally considered by hydrologic professionals and researchers to be a reasonable distance from a monitoring well for detecting groundwater conditions for a particular user. This range of buffers enabled us to then perform a sensitivity analysis of how the results changed according to the buffer distance. The normal distribution that we observed in the 2.4 km (1.5 mi; Supplementary Fig. 4.1) buffer tells us that monitoring well coverage in the basin will be more statistically representative across groundwater basins and less prone to skewed results.

The Department of Water Resources states in its Monitoring Networks and Identification of Data Gaps Best Management Practice Guidance Document (https://water.ca.gov/-/media/DWR-Website/Web-Pages/Programs/Groundwater-Management/Sustainable-Groundwater-Management/Best-Management-Practices-and-Guidance-Documents/Files/BMP-2-Monitoring-Networks-and-Identification-of-Data-Gaps_ay_19.pdf) that "there is no definitive rule for the density of groundwater monitoring points needed in a basin". Instead, the guidance document defers to professional judgment. Because groundwater conditions can be spatially heterogeneous within a groundwater basin, particularly near surface water features that have been subject to fluvial morphology, monitoring wells need to be sufficiently close enough to production wells (agriculture and domestic) and ecosystems to monitor groundwater conditions for that user.

Analysis of Undesirable Results and Minimum Threshold Criteria in Sustainability Plans

Under the Sustainable Groundwater Management Act, Sustainability Plans are required to include quantitative metrics to define sustainability, which include minimum thresholds. Groundwater elevation minimum thresholds define “significant and unreasonable” conditions at individual representative monitoring wells. Each jurisdiction has the discretion to quantify these metrics and determine quantitatively how to aggregate minimum threshold exceedances to trigger a ‘significant and unreasonable’ condition (“undesirable result”; i.e., an undesirable amount of groundwater elevation decline). Each Agency determines local definitions of groundwater elevation minimum thresholds and undesirable results. Here, we analyzed and synthesized the various definitions of groundwater elevation undesirable results (Supplementary Table 4.5) and groundwater elevation minimum thresholds (Supplementary Table 4.6). First, we cataloged each Sustainability Plans definition for groundwater elevation undesirable results and groundwater elevation minimum thresholds. Then, through two rounds of qualitative coding, we categorized the definitions into groups. Supplementary Tables 4.5-4.6 show the results of this analysis and the counts of Sustainability Plans that used each of the definitional criteria.

Supplementary Table 4.1. Coverage and protection summary statistics by stakeholder.

Stakeholder	Buffer (km)	Covered		Protected	
		Count	%	Count	%
Agriculture	0.8	3,372 wells	8.9	2,599 wells	6.9
	1.6	11,299 wells	29.8	9,045 wells	23.9
	2.4	18,520 wells	48.9	14,964 wells	39.5
	3.2	25,329 wells	66.9	20,612 wells	54.4
	4.0	29,491 wells	77.9	24,113 wells	63.7
Big Farms	0.8	NA	NA	NA	NA
	1.6	NA	NA	NA	NA
	2.4	NA	NA	NA	NA
	3.2	NA	NA	NA	NA
	4.0	NA	NA	NA	NA
Small Farms	0.8	NA	NA	NA	NA
	1.6	NA	NA	NA	NA
	2.4	NA	NA	NA	NA
	3.2	NA	NA	NA	NA
	4.0	NA	NA	NA	NA
Domestic	0.8	7,719 wells	8.8	5,966 wells	6.8
	1.6	25,391 wells	28.9	19,333 wells	22.0
	2.4	42,716 wells	48.7	32,449 wells	37.0
	3.2	58,612 wells	66.8	43,993 wells	50.1
	4.0	67,901 wells	77.4	51,539 wells	58.7
Domestic Wells within Disadvantaged Communities	0.8	2,431 wells	8.3	1,727 wells	5.9
	1.6	8,197 wells	27.9	5,870 wells	20.0
	2.4	14,194 wells	48.3	10,106 wells	34.4
	3.2	19,161 wells	65.2	13,577 wells	46.2

Stakeholder	Buffer (km)	Covered		Protected	
		Count	%	Count	%
	**4.0**	22,307 wells	75.9	15,893 wells	54.0
**Domestic Wells within Non-Disadvantaged Communities**	**0.8**	5,288 wells	9.1	4,239 wells	7.3
	**1.6**	17,194 wells	29.5	13,463 wells	23.1
	**2.4**	28,522 wells	48.9	22,343 wells	38.3
	**3.2**	39,451 wells	67.6	30,416 wells	52.1
	**4.0**	45,594 wells	78.1	35,646 wells	61.1
**Environment**	**0.8**	213 km^2 (52,633 acres)	13.8	39 km^2 (9,637 acres)	2.5
	**1.6**	459 km^2 (113,421 acres)	29.8	89 km^2 (21,992 acres)	5.8
	**2.4**	645 km^2 (159,383 acres)	41.9	138 km^2 (34,101 acres)	9.0
	**3.2**	807 km^2 (199,414 acres)	52.4	181 km^2 (44,726 acres)	11.8
	**4.0**	932 km^2 (230,302 acres)	60.5	226 km^2 (55,846 acres)	14.7

Supplementary Table 4.2. Chi-squared test results to compare coverage and protection between stakeholder groups. Significant thresholds are denoted as * (p≤0.05), ** (p≤0.01), *** (p≤0.001).

	Buffer	Degrees of Freedom	Chi-squared Statistic	
			Covered	Protected
Agriculture versus Domestic	0.8	1	0.37	0.05
	1.6	1	10.38***	68.01***
	2.4	1	0.54	172.50***
	3.2	1	0.10	398.00***
	4.0	1	3.77*	407.00***
Environment versus Agriculture	0.8	1	33.3***	35030***
	1.6	1	829***	40040***
	2.4	1	1670***	40200***
	3.2	1	3607***	39910***
	4.0	1	4443***	37960***
Environment versus Domestic	0.8	1	39.9***	41800***
	1.6	1	965***	47400***
	2.4	1	2395***	49570***
	3.2	1	5522***	48510***
	4.0	1	6819***	47130***
Domestic: Disadvantaged Communities versus non-Disadvantaged Communities	0.8	1	15.32***	78.43***
	1.6	1	24.00***	136.30***
	2.4	1	2.90	264.00***
	3.2	1	52.79***	268.00***
	4.0	1	57.88***	393.40***

Supplementary Table 4.3. Summary statistics of Sustainability Plans with ≥50% of individual wells covered and protected in Sustainability Plan.

Purpose	Buffer (km)	Total Plans	Covered		Protected	
			Number of Plans	Percentage of Plans	Number of Plans	Percentage of Plans
Agriculture	**0.8**	105	6	5.7	3	2.9
	**1.6**	105	39	37.1	19	18.1
	**2.4**	105	67	63.8	39	37.1
	**3.2**	105	90	85.7	62	59.0
	**4.0**	105	97	92.4	73	69.5
Domestic	**0.8**	104	4	3.8	2	1.9
	**1.6**	104	32	30.8	12	11.5
	**2.4**	104	66	63.5	30	28.8
	**3.2**	104	87	83.7	54	51.9
	**4.0**	104	97	93.3	61	58.7

Supplementary Table 4.4. Summary statistics of Sustainability Plans with >=50% of individual groundwater-dependent ecosystems covered and protected in Plan.

Buffer	Total Plans	Covered		Protected	
		Number of Plans	Percentage of Plans	Number of Plans	Percentage of Plans
**0.8 km**	106	9	8.5	2	1.9
**1.6 km**	106	31	29.2	6	5.7
**2.4 km**	106	57	53.8	8	7.5
**3.2 km**	106	71	67.0	11	10.4
**4.0 km**	106	82	77.4	14	13.2

Supplementary Table 4.5. Counts of groundwater elevation undesirable results (UR) definitional criteria used by Sustainability Plans. The count column does not sum to 108 as some Sustainability Plans used multiple definitions.

Groundwater elevation undesirable result definition criteria	Count
UR occurs when a proportion of the Representative Monitoring Sites in the Plan Area measure groundwater elevations lower than the Minimum Threshold	63
Any Representative Monitoring Site measures groundwater elevations lower than the Minimum Threshold	12
UR occurs if continued groundwater production/development of new wells is negatively impacted by lowered GW levels	12
UR occurs if other Sustainable Management Criteria (land subsidence, water quality degradation, seawater intrusion) occurs	11
UR occurs if beneficial users (domestic wells, agriculture wells, groundwater-dependent ecosystems) are negatively impacted by lowering of groundwater levels	10
UR occurs if a fixed number of Representative Monitoring Sites measure GW elevations lower than the Minimum Threshold	9
UR occurs if the Minimum Threshold is exceeded in a fixed number of Management Areas, a sub-unit of a managed subbasin	4
UR occurs if the Minimum Threshold is exceeded	4
UR occurs if groundwater levels decline to a certain point, or if the rate of GW decline increases	3
UR occurrence set based on criteria that do not fit into other categories listed here	3
No trigger for UR has been set	1

Supplementary Table 4.6. Counts of groundwater elevation minimum threshold (MT) definitional criteria used by Sustainability Plans. The count column does not sum to 108 as some Sustainability Plans used multiple definitions.

**Groundwater elevation minimum threshold definition criteria**	**Count**
MT set at the lowest historic groundwater elevation, and may have an additional buffer above or below the historic low	45
MT set specifically to be protective of domestic wells, ag wells, and/or environmental users (groundwater-dependent ecosystems)	26
MT set based on groundwater conditions in recent years	20
MT set based on groundwater conditions in projected scenarios	19
MT set as a fixed groundwater elevation below ground surface, above mean sea level, above a Representative Monitoring Site, or below the Measurable Objective	16
MT set based on measured or projected drought conditions	10
MT set based on criteria that do not fit into other categories	6
Unclear how MT was set	4
MT set to mitigate or reverse future seawater intrusion	2
MT set to allow flexibility under projected climate change conditions	2
No MT set	1

Supplementary Table 4.7. Maximum rooting depths for vegetation species identified within the Sustainability Plans as indicated in the Natural Communities Commonly Associated with Groundwater dataset. Maximum rooting depth (in units of meters and feet) are based on published literature and downloaded from The Nature Conservancy's Plant Rooting Depth Database (https://groundwaterresourcehub.org/sgma-tools/gde-rooting-depths-database-for-gdes/). Dominant species without published maximum rooting depth information were assigned the average maximum rooting depth (3.04 meters or 9.98 feet).

Dominant Species Name	Maximum Rooting Depth meters	feet	Assigned the average?
Acacia greggii	5.5	18.04	No
Acer negundo	3.23	10.61	No
Ailanthus altissima	1.2	3.94	No
Allenrolfea occidentalis	0.83	2.71	No
Alnus rhombifolia	3.04	9.98	Yes
Anemopsis californica	0.12	0.39	No
Artemisia douglasiana	3.04	9.98	Yes
Artemisia tridentata spp. Parishii	3.04	9.98	Yes
Arundo donax	4.9	16.08	No
Atriplex lentiformis	3.04	9.98	Yes
Atriplex parryi	3.04	9.98	Yes
Atriplex spinifera	3.04	9.98	Yes
Baccharis salicifolia	0.6	1.97	No
Betula occidentalis	3.04	9.98	Yes
Bolboschoenus maritimus	3.04	9.98	Yes
Carex barbarae	3.04	9.98	Yes
Celtis reticulata	4.6	15.09	No
Cephalanthus occidentalis	3.04	9.98	Yes
Chilopsis linearis	1.6	5.25	No
Cressa truxillensis	3.04	9.98	Yes
Distichlis spicata	0.61	1.98	No
Forestiera pubescens	3.04	9.98	Yes
Fraxinus latifolia	3.04	9.98	Yes
Heterotheca oregona	3.04	9.98	Yes

Dominant Species Name	Maximum Rooting Depth meters	feet	Assigned the average?
Isocoma acradenia	3.04	9.98	Yes
Juglans californica	1.8	5.91	No
Juglans hindsii	3.04	9.98	Yes
Juncus acutus	3.04	9.98	Yes
Juncus arcticus	0.21	0.69	No
Lepidium latifolium	3.04	9.98	Yes
Lepidospartum squamatum	3.04	9.98	Yes
Leymus triticoides	3.04	9.98	Yes
Myoporum laetum	3.04	9.98	Yes
Not Applicable	3.04	9.98	Yes
Persicaria lapathifolia	3.04	9.98	Yes
Phragmites australis	1.6	5.25	No
Picea sitchensis	0.8	2.62	No
Pinus contorta	1.16	3.79	No
Platanus racemosa	3.04	9.98	Yes
Pluchea sericea	1.31	4.28	No
Populus fremontii	1.09	3.57	No
Populus tremuloides	1.32	4.33	No
Populus trichocarpa	0.95	3.12	No
Prosopis glandulosa	6.45	21.16	No
Prunus fasciculata	3.04	9.98	Yes
Quercus agrifolia	9.27	30.42	No
Quercus lobata	24.38	80	No
Rosa californica	3.04	9.98	Yes
Rosa woodsii	3.04	9.98	Yes
Rubus armeniacus	3.04	9.98	Yes
Salix exigua	3.04	9.98	Yes
Salix gooddingii	2.1	6.89	No

Dominant Species Name	Maximum Rooting Depth meters	feet	Assigned the average?
Salix laevigata	3.04	9.98	Yes
Salix lasiolepis	3.04	9.98	Yes
Salix lucida	3.04	9.98	Yes
Salix spp.	0.8	2.62	No
Sambucus mexicana	3	9.84	No
Sambucus nigra	3.04	9.98	Yes
Sarcobatus vermiculatus	3.73	12.25	No
Schoenoplectus acutus	0.6	1.97	No
Schoenoplectus acutus var. occidentalis	3.04	9.98	Yes
Schoenoplectus americanus	0.65	2.13	No
Schoenoplectus californicus	3.04	9.98	Yes
Sequoia sempervirens	4.2	13.78	No
Sesbania punicea	3.04	9.98	Yes
Sporobolus airoides	3.04	9.98	Yes
Suaeda moquinii	3.04	9.98	Yes
Tamarix parviflora	3.04	9.98	Yes
Tamarix ramosissima	6.32	20.73	No
Tamarix spp.	4.9	16.08	No
Typha angustifolia	3.04	9.98	Yes
Typha domingensis	0.27	0.89	No
Typha latifolia	0.25	0.82	No
Vitis californica	3.04	9.98	Yes

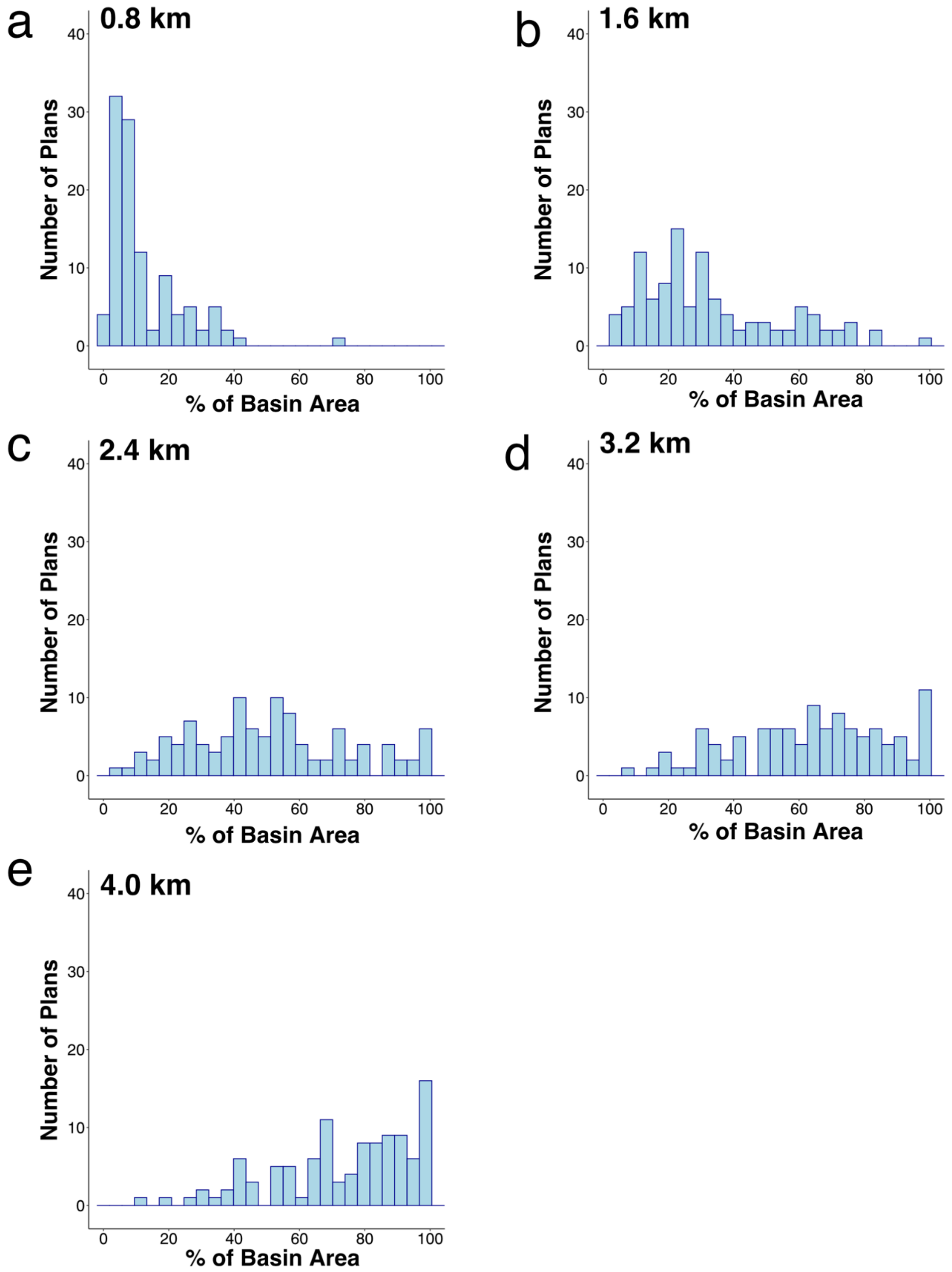

Supplementary Fig. 4.1. Histogram of the total basin area covered within a basin based on each buffer size around the representative monitoring wells in the basin: (a) 0.8 km (0.5 mi) buffer, (b) 1.6 km (1.0 mi) buffer, (c) 2.4 km (1.5 mi) buffer, (d) 3.2 km (2.0 mi) buffer, and (e) 4.0 km (2.5 mi) buffer.

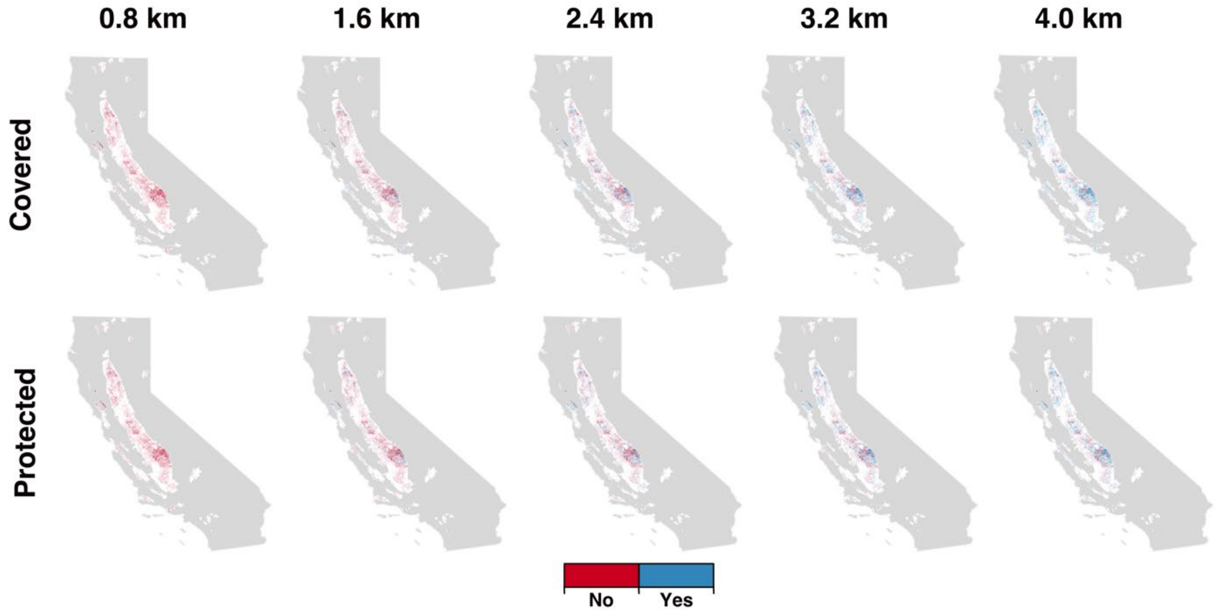

Supplementary Fig. 4.2. Agricultural coverage and protection by well for each buffer analyzed.

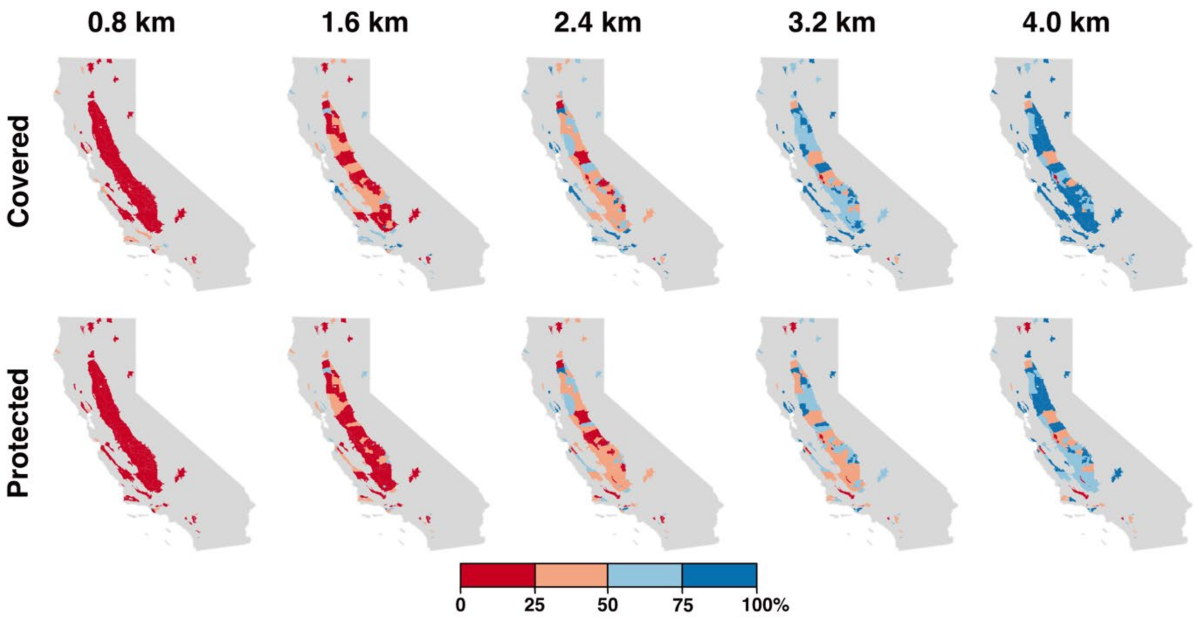

Supplementary Fig. 4.3. Agricultural coverage and protection by Groundwater Sustainability Plan for each buffer analyzed.

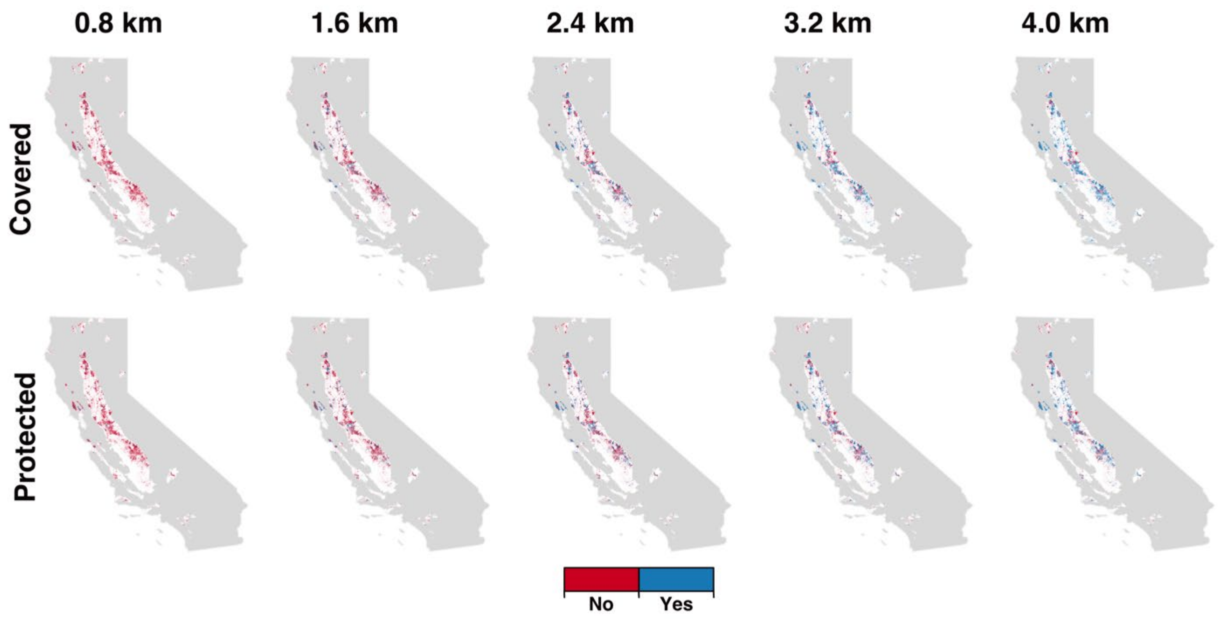

Supplementary Fig. 4.4. Domestic coverage and protection by well for each buffer analyzed.

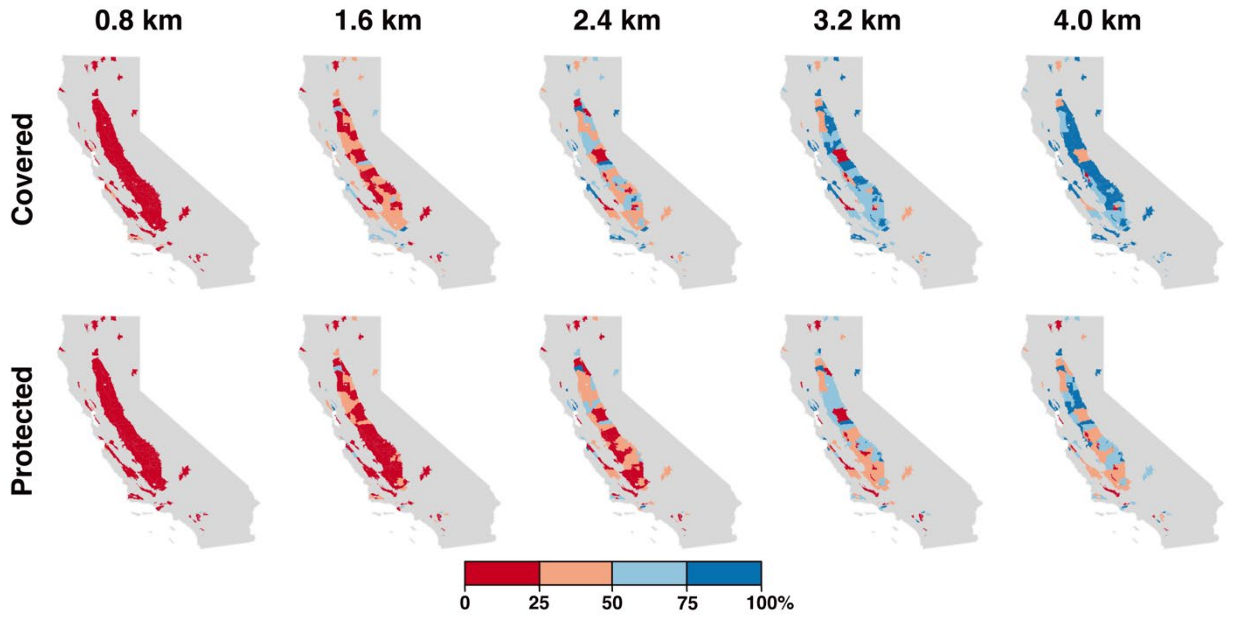

Supplementary Fig. 4.5. Domestic coverage and protection by Groundwater Sustainability Plan for each buffer analyzed.

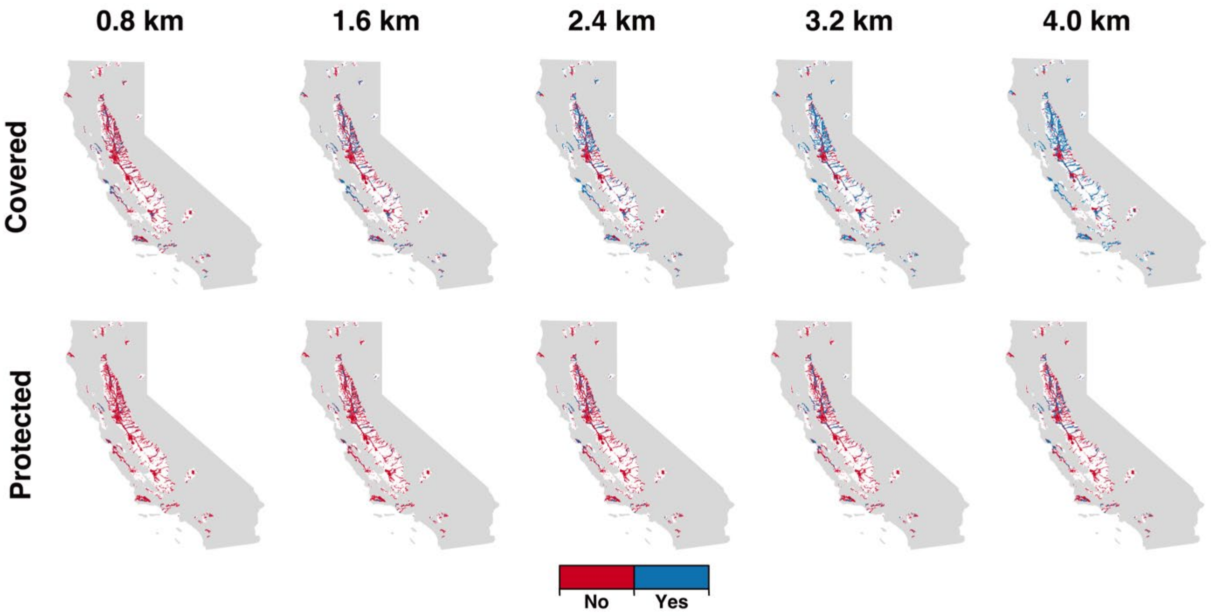

Supplementary Fig. 4.6. Environment coverage and protection by groundwater-dependent ecosystems for each buffer analyzed.

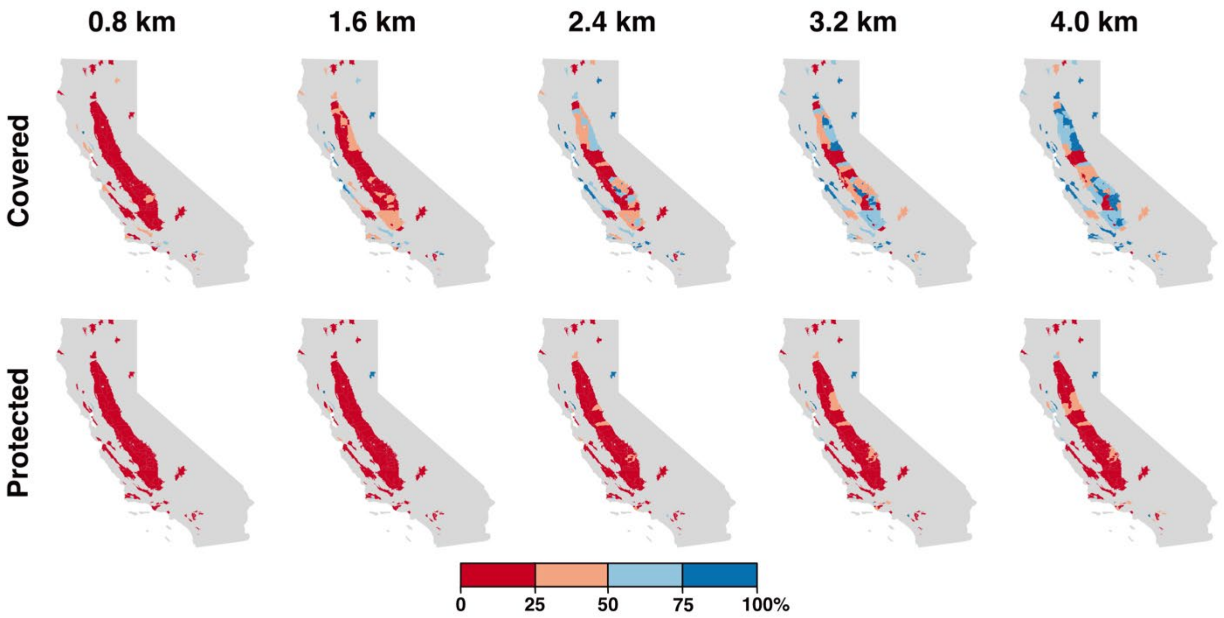

Supplementary Fig. 4.7. Environment coverage and protection by Groundwater Sustainability Plan for each buffer analyzed.

Supplementary Section 5. Coverage and Protection Results by Groundwater Sustainability Plan

For each Groundwater Sustainability Plan we provide maps depicting where the groundwater basin is located, and which individual wells (agriculture, domestic) or ecosystems were covered and protected by the Sustainability Plan minimum thresholds.

California does not provide actual latitude and longitude location for all well data. As a result, many wells overlap because their location is set to the centroid of a township-Section-range. **Therefore, well locations were nominally adjusted for visualization purposes ONLY (i.e., the latitudes and longitudes were adjusted randomly); the adjustment was performed <u>AFTER ANALYSIS</u>.** As a result of the adjusted locations, some wells in the forthcoming figures may appear outside of the Sustainability Plan boundary.

The legend below applies to each map and indicates whether individual wells or ecosystems are: (1) covered and protected (in blue), (2) covered but not protected (in pink), or (3) not covered and not protected (in red).

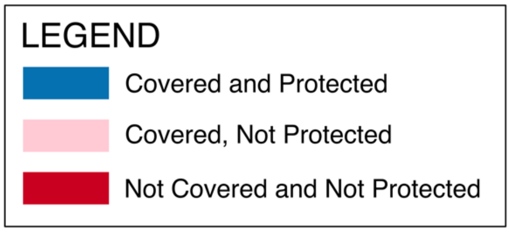

1−002.01 TULELAKE

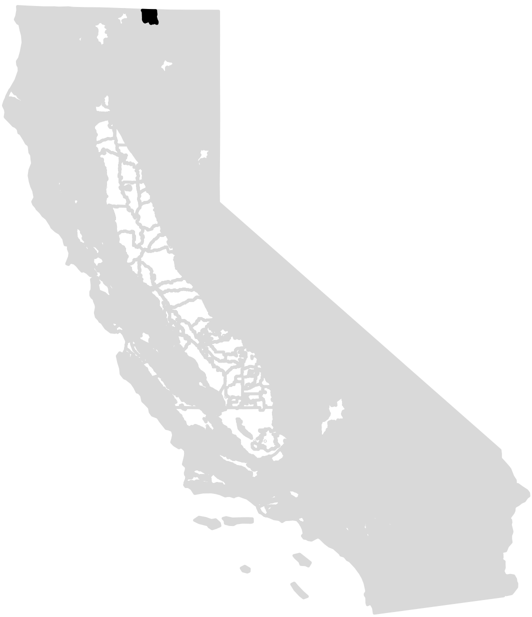

AGRICULTURE

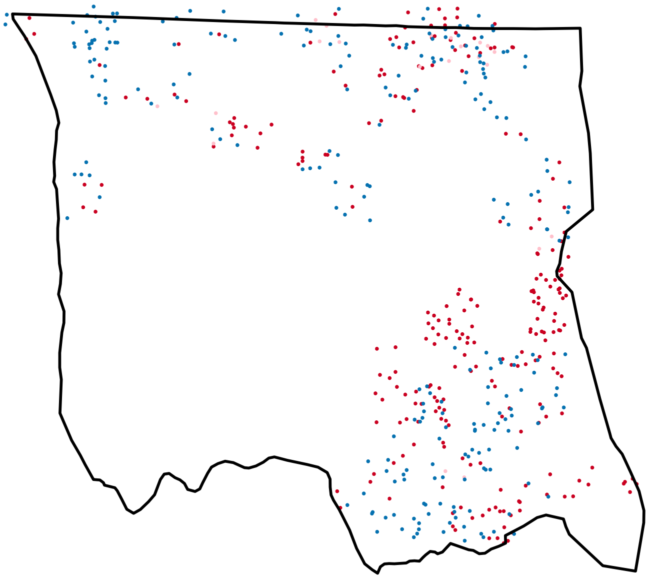

DOMESTIC

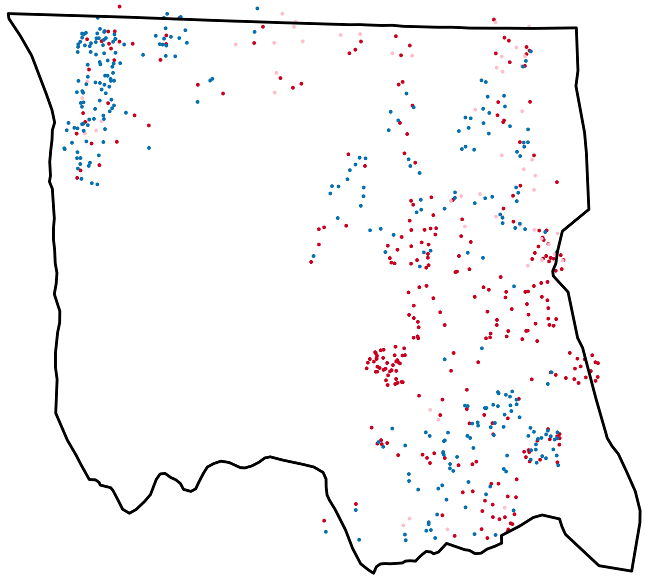

ENVIRONMENT

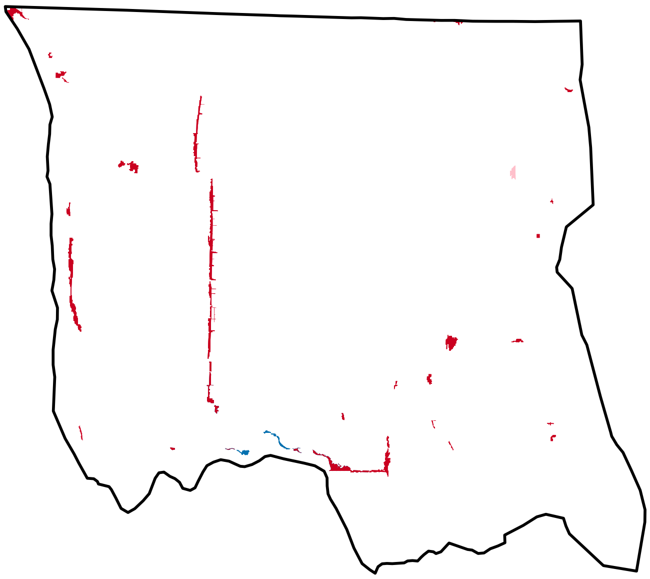

DISADVANTAGED COMMUNITIES

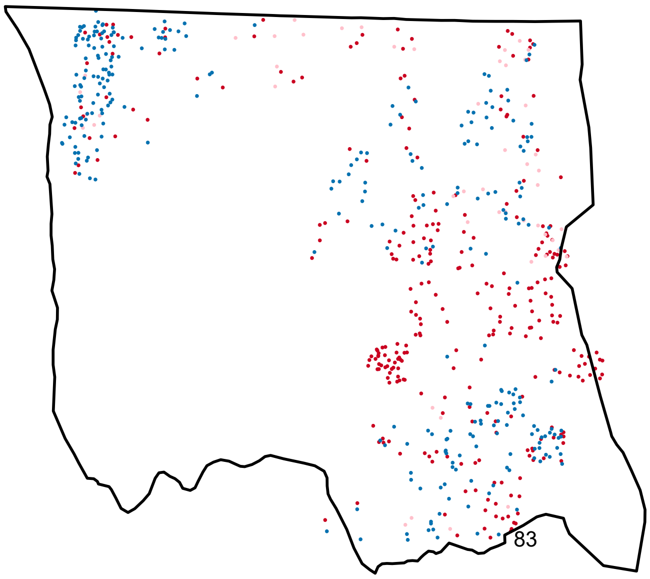

1−003 BUTTE VALLEY

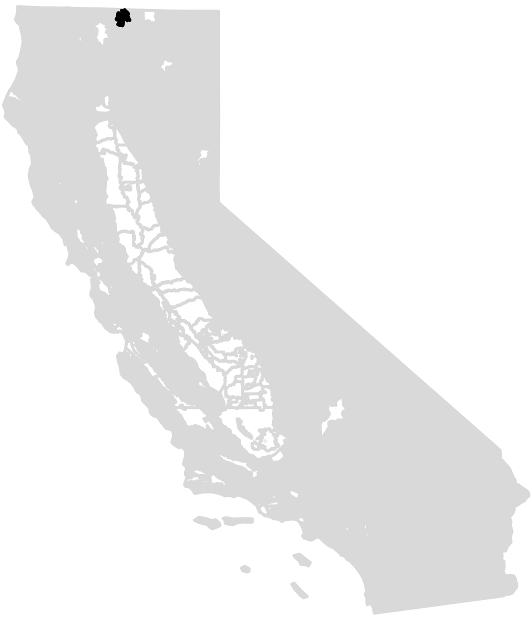

AGRICULTURE

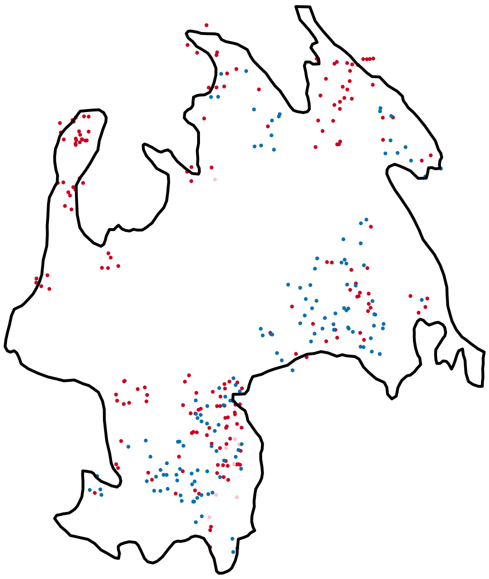

DOMESTIC

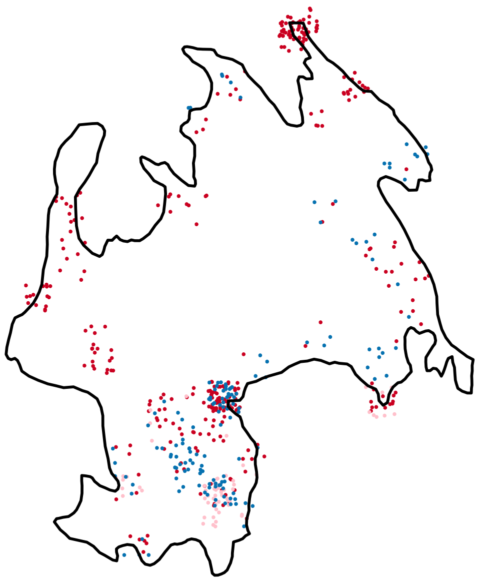

ENVIRONMENT

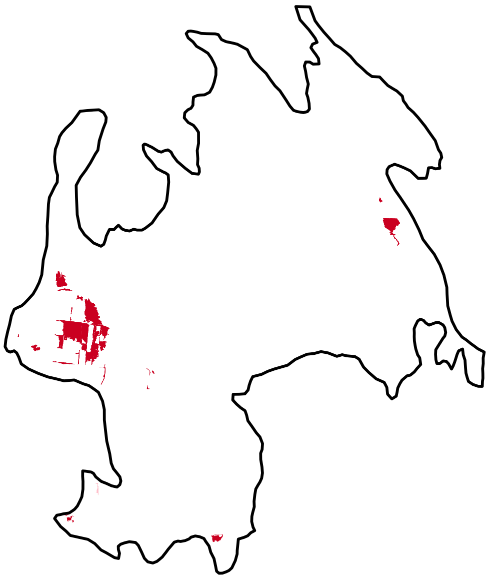

DISADVANTAGED COMMUNITIES

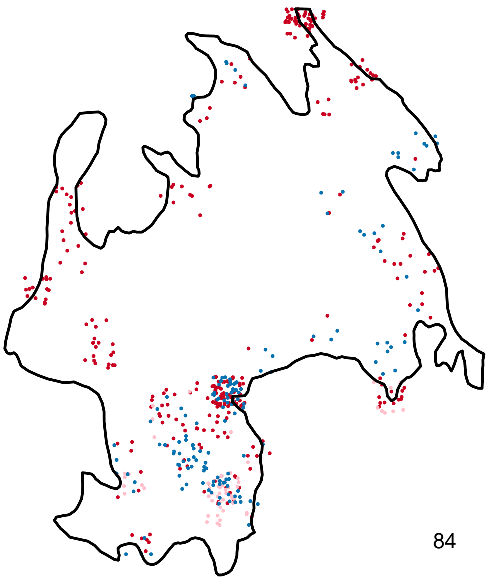

1−004 SHASTA VALLEY

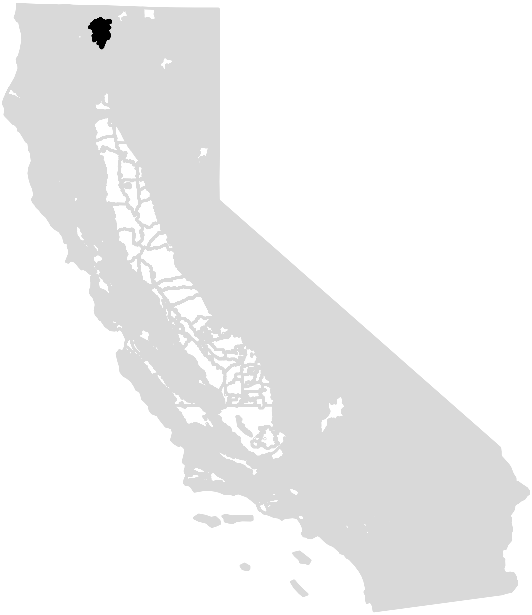

AGRICULTURE

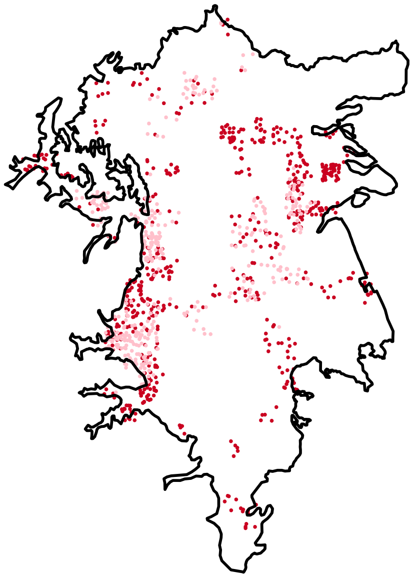

DOMESTIC

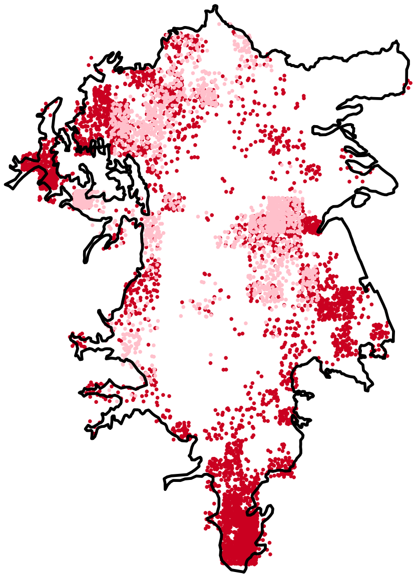

ENVIRONMENT

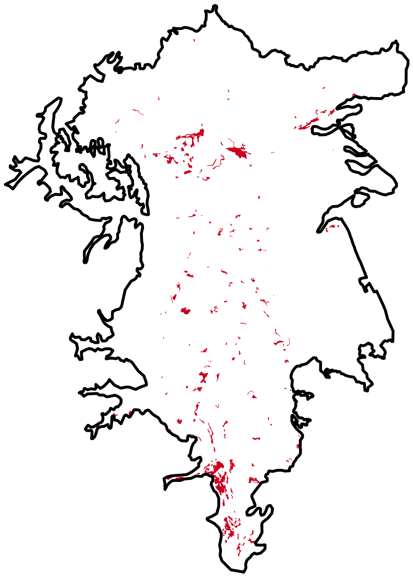

DISADVANTAGED COMMUNITIES

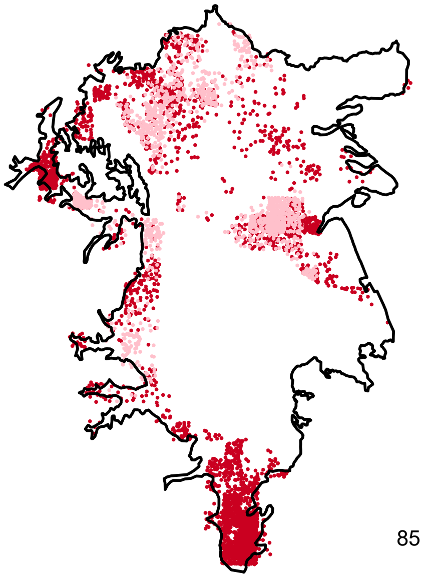

1−005 SCOTT RIVER VALLEY

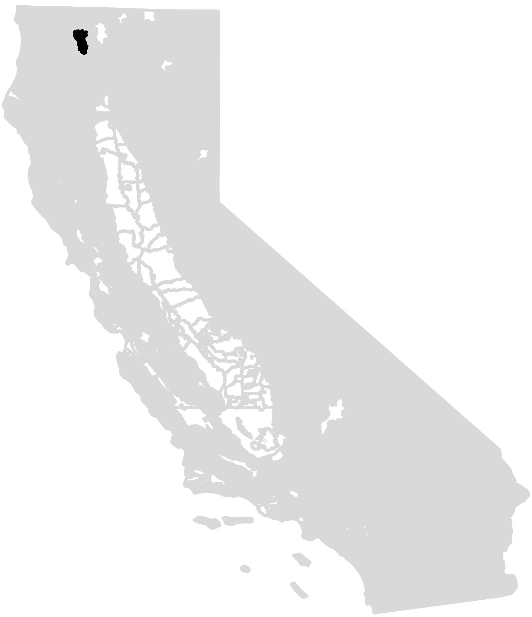

AGRICULTURE

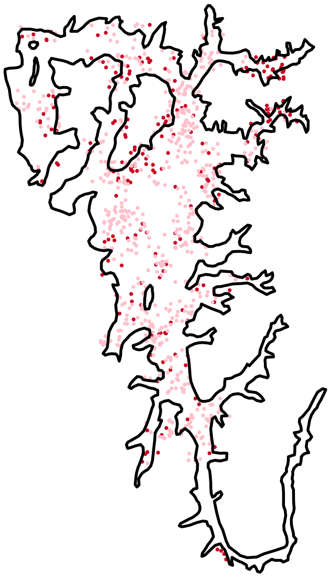

DOMESTIC

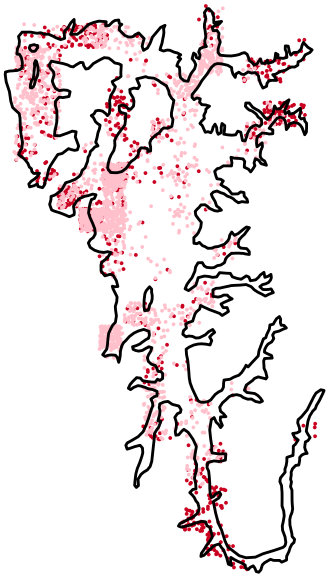

ENVIRONMENT

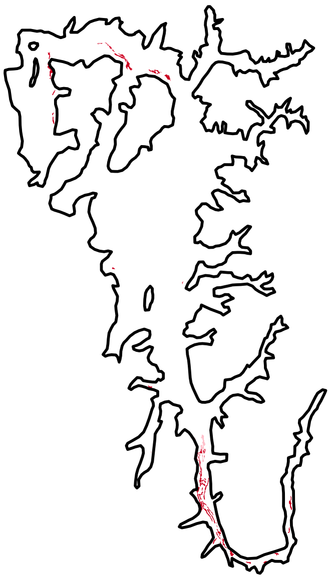

DISADVANTAGED COMMUNITIES

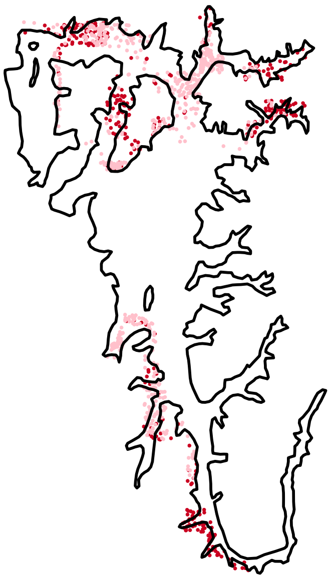

1−010 EEL RIVER VALLEY

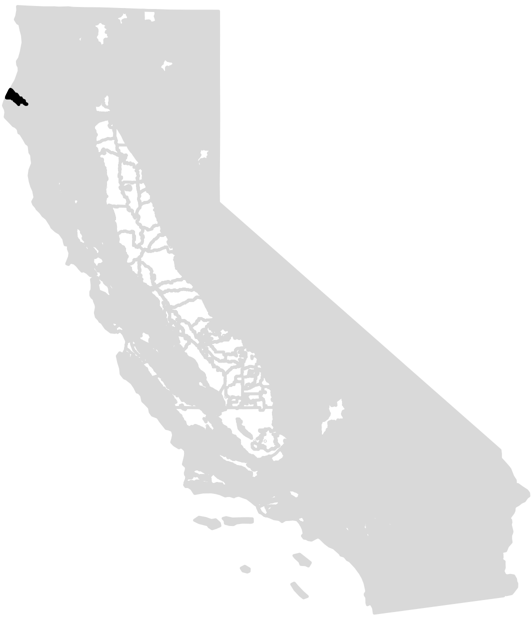

AGRICULTURE

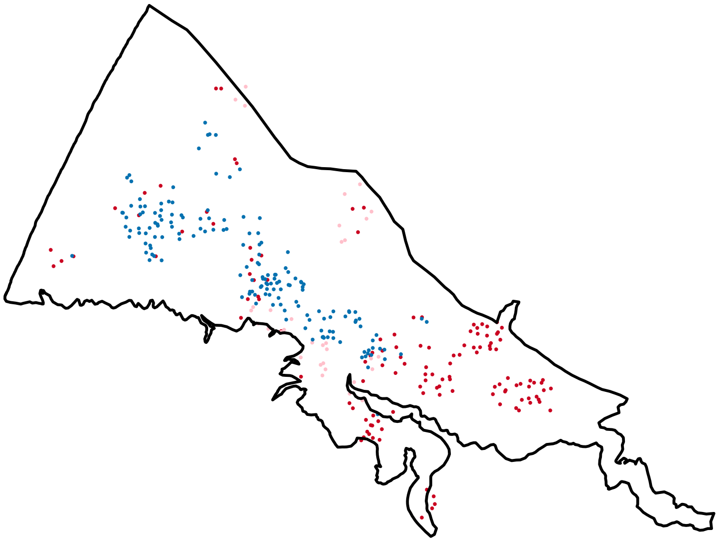

DOMESTIC

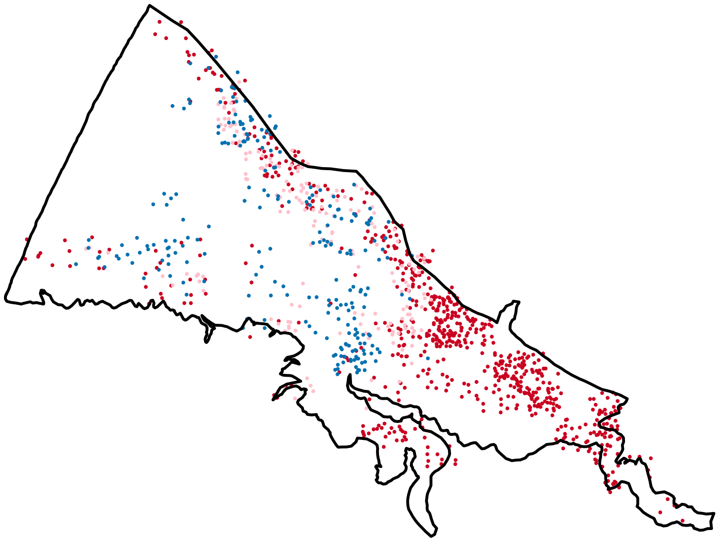

ENVIRONMENT

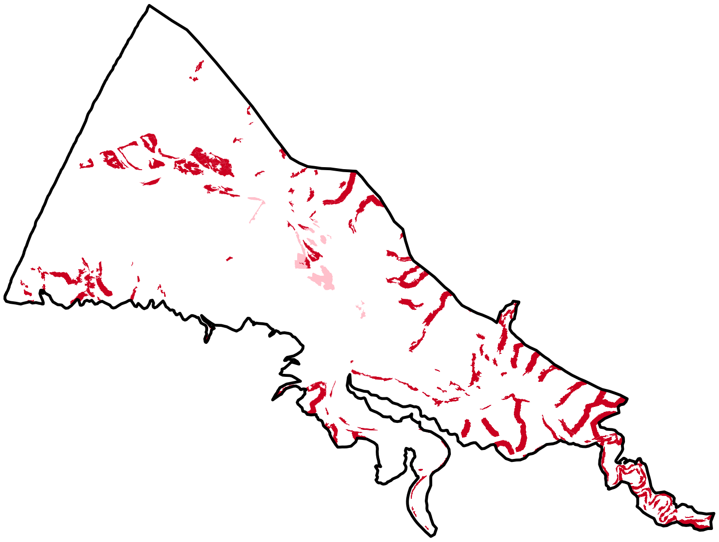

DISADVANTAGED COMMUNITIES

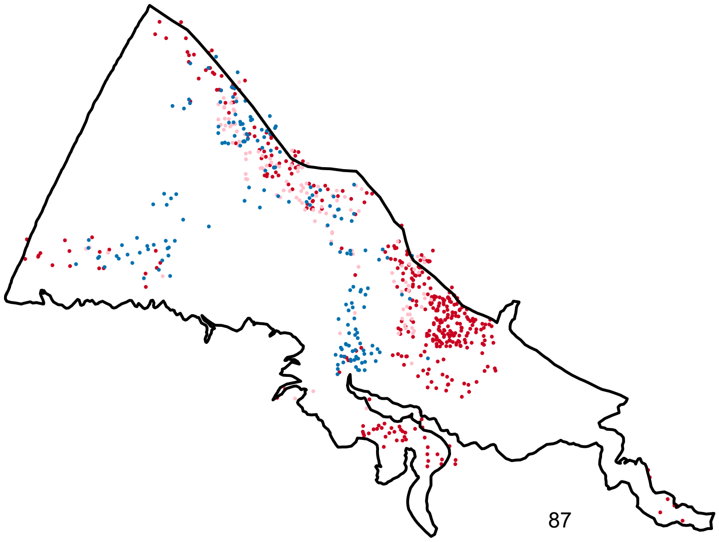

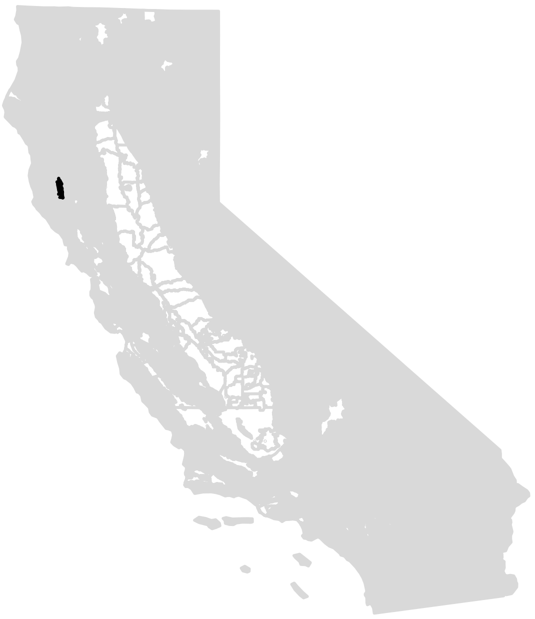

1−052 UKIAH VALLEY

AGRICULTURE

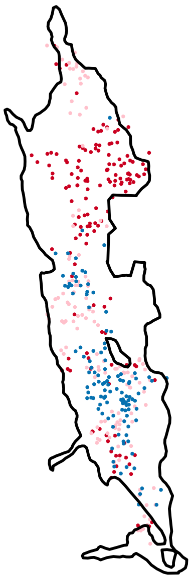

DOMESTIC

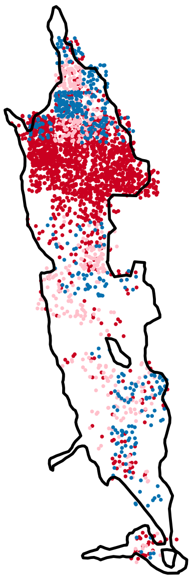

ENVIRONMENT

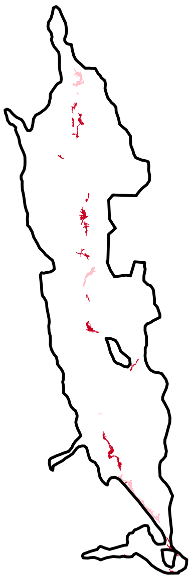

DISADVANTAGED COMMUNITIES

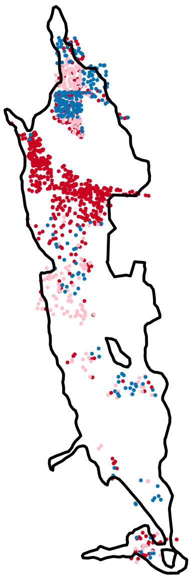

1−055.01 SANTA ROSA PLAIN

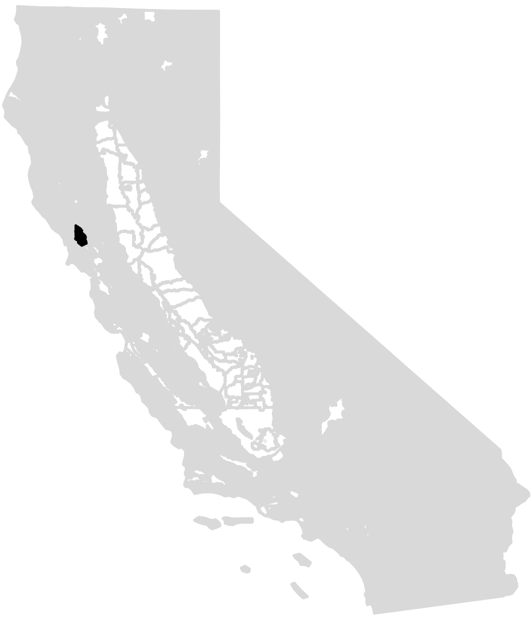

AGRICULTURE

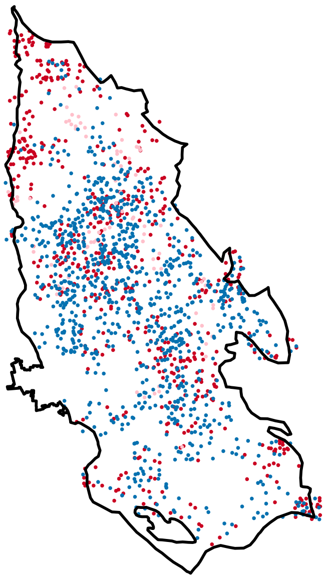

DOMESTIC

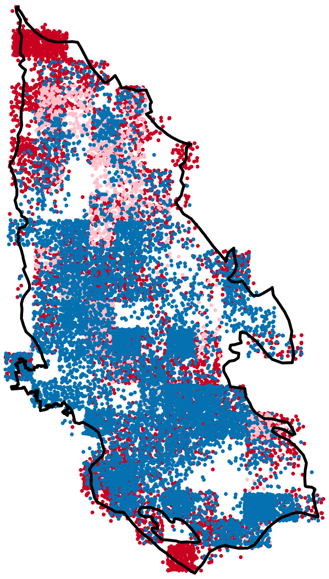

ENVIRONMENT

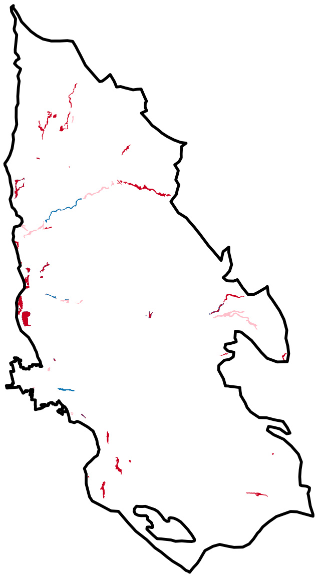

DISADVANTAGED COMMUNITIES

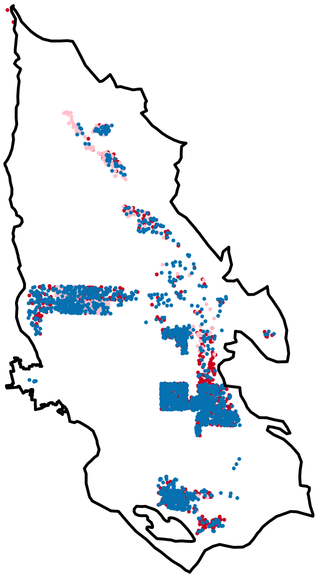

2−001 PETALUMA VALLEY

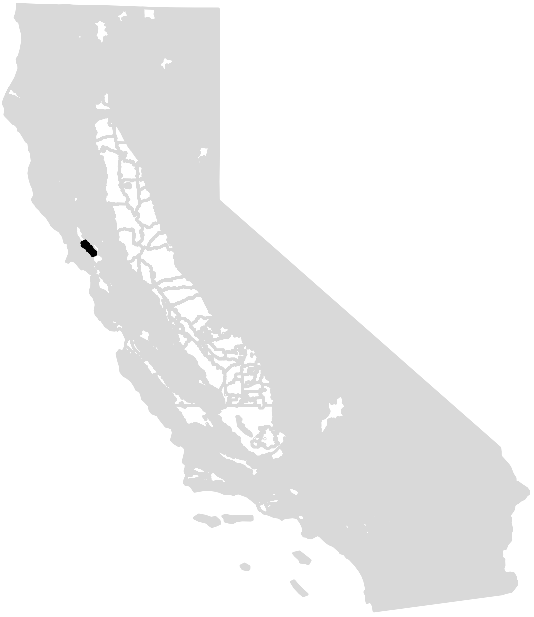

AGRICULTURE

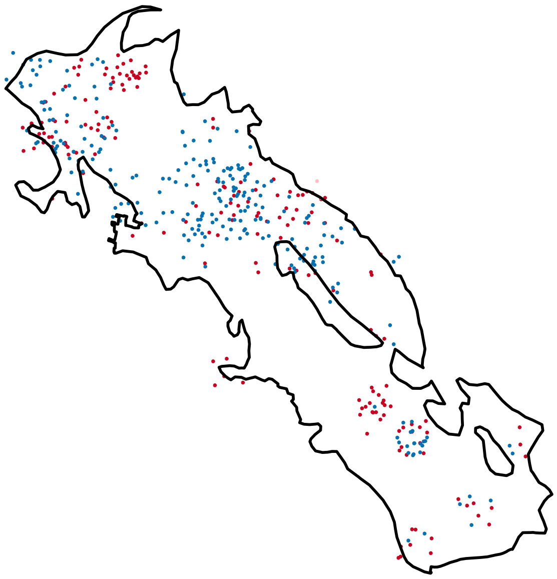

DOMESTIC

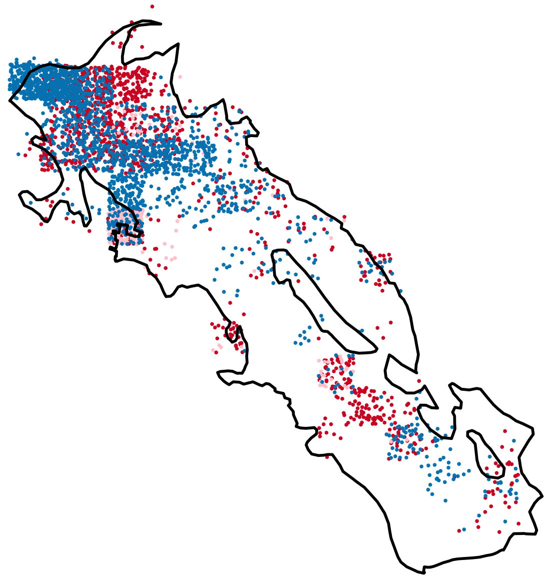

ENVIRONMENT

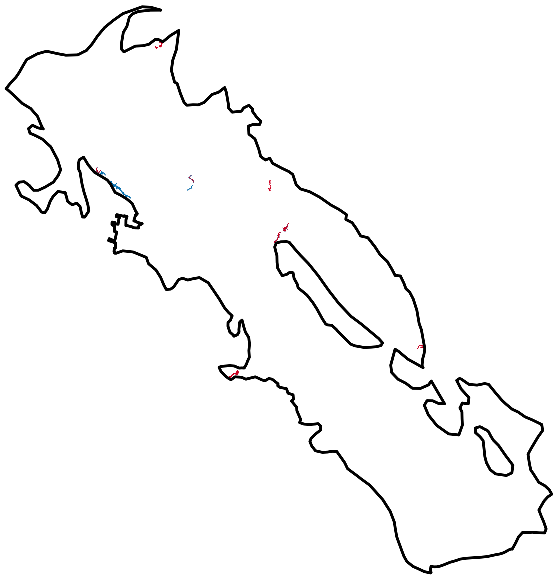

DISADVANTAGED COMMUNITIES

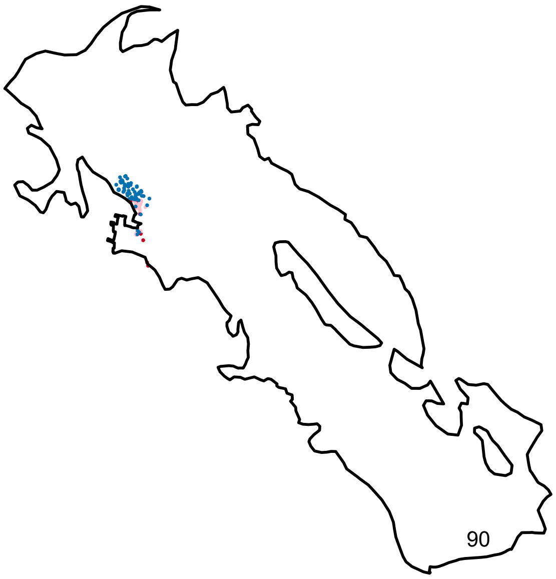

2−002.01 NAPA VALLEY

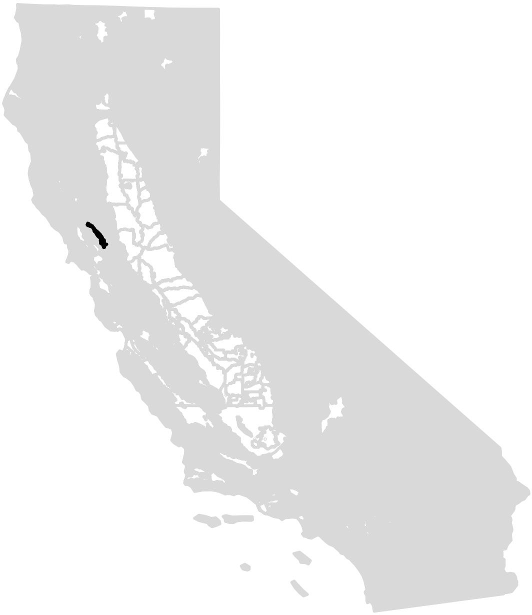

AGRICULTURE

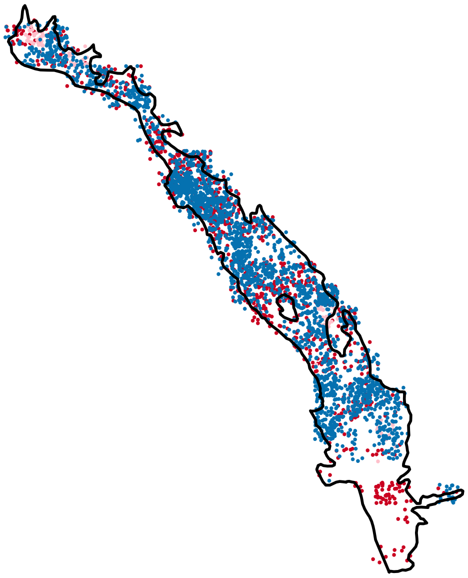

DOMESTIC

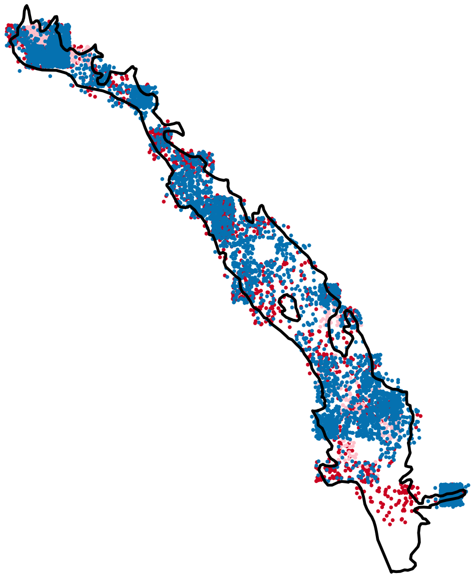

ENVIRONMENT

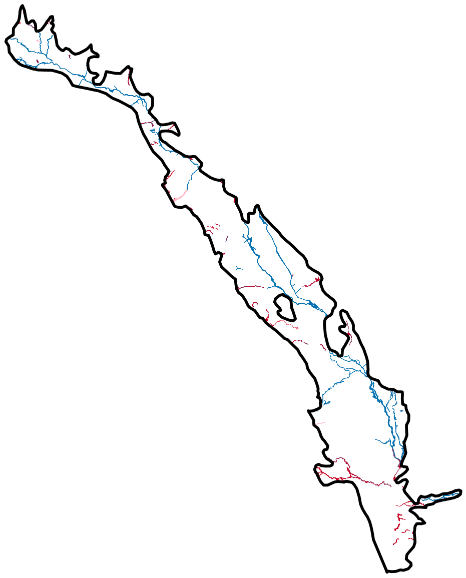

DISADVANTAGED COMMUNITIES

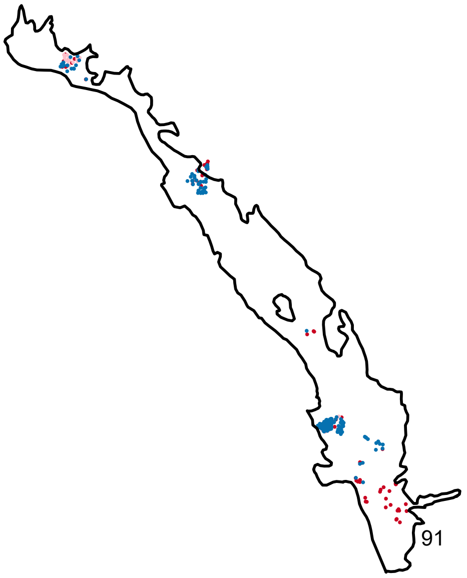

2−002.02 SONOMA VALLEY

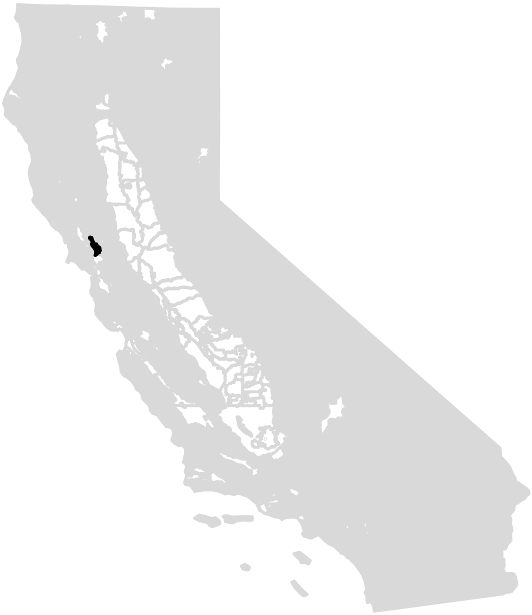

AGRICULTURE

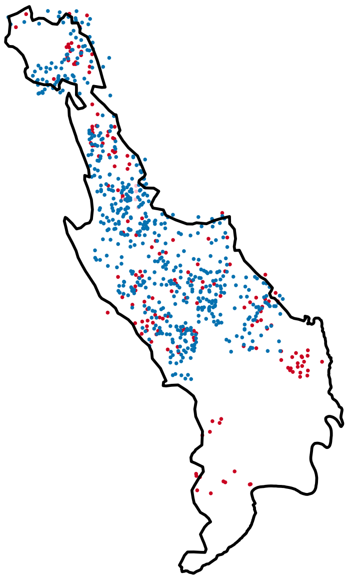

DOMESTIC

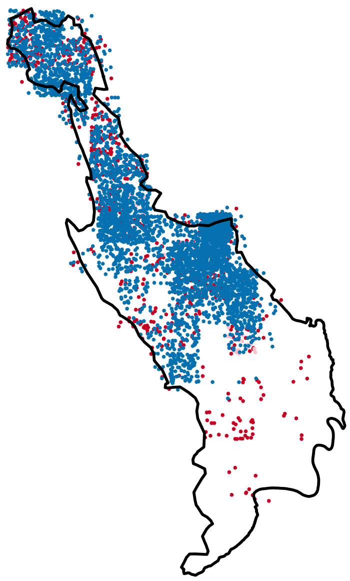

ENVIRONMENT

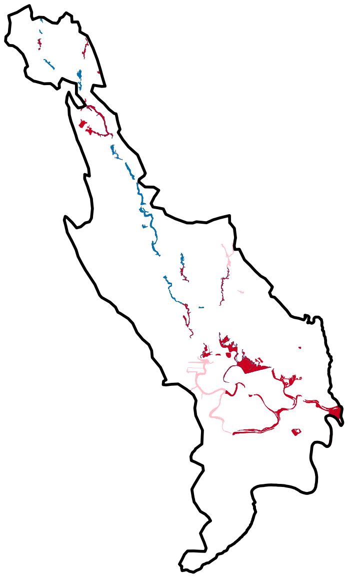

DISADVANTAGED COMMUNITIES

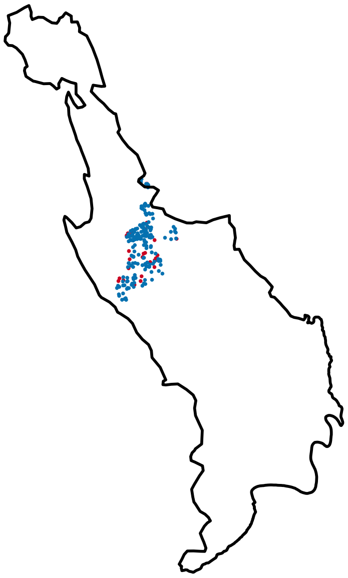

2−009.04 EAST BAY PLAIN

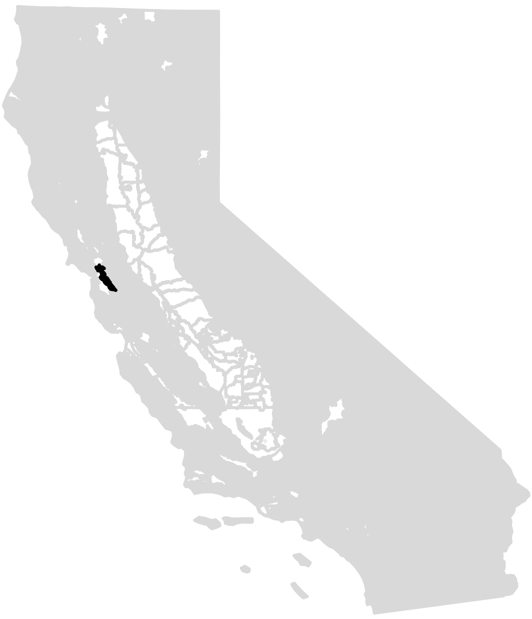

AGRICULTURE

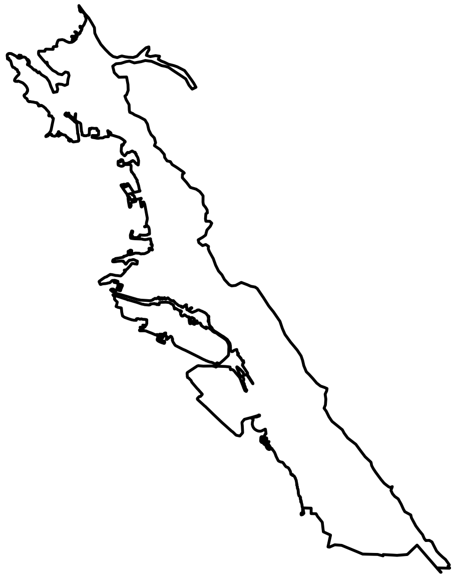

DOMESTIC

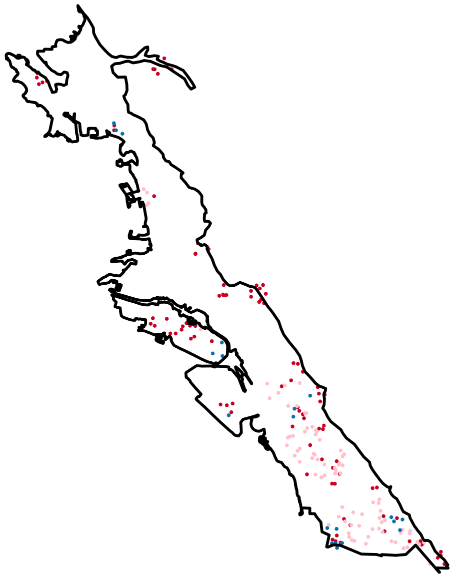

ENVIRONMENT

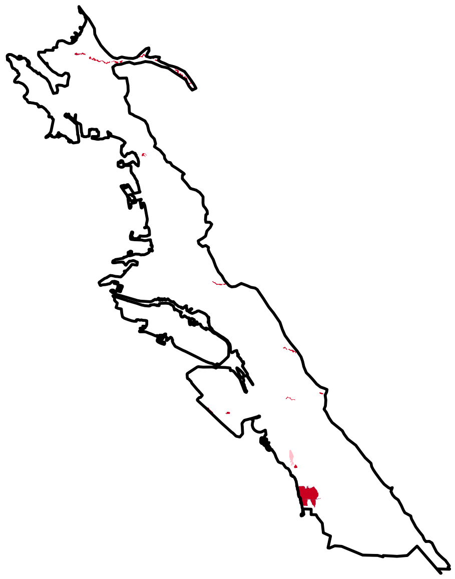

DISADVANTAGED COMMUNITIES

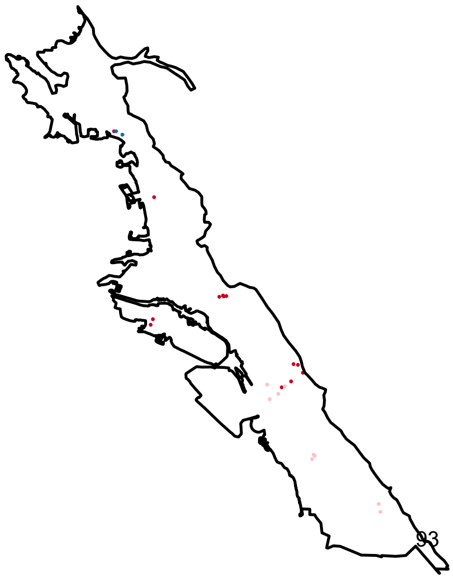

3−001 SANTA CRUZ MID−COUNTY

AGRICULTURE

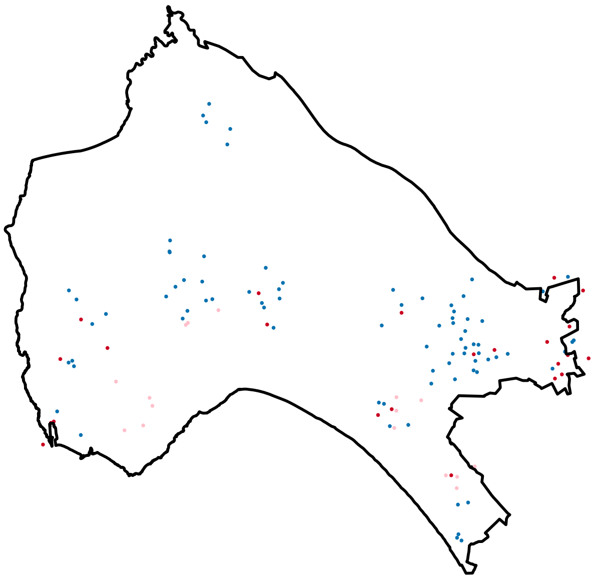

DOMESTIC

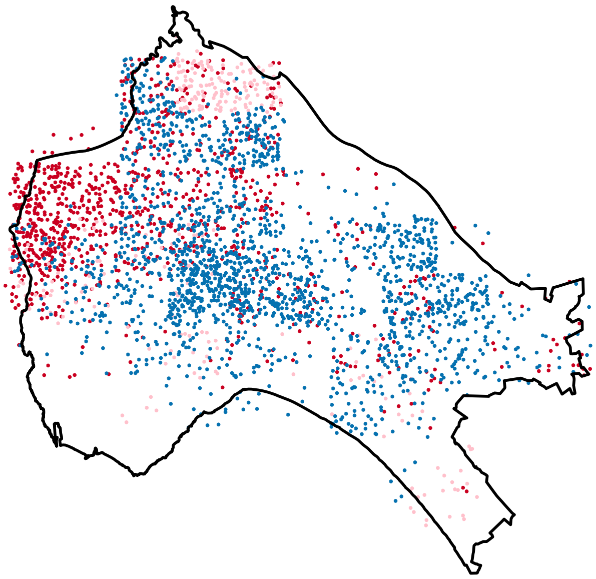

ENVIRONMENT

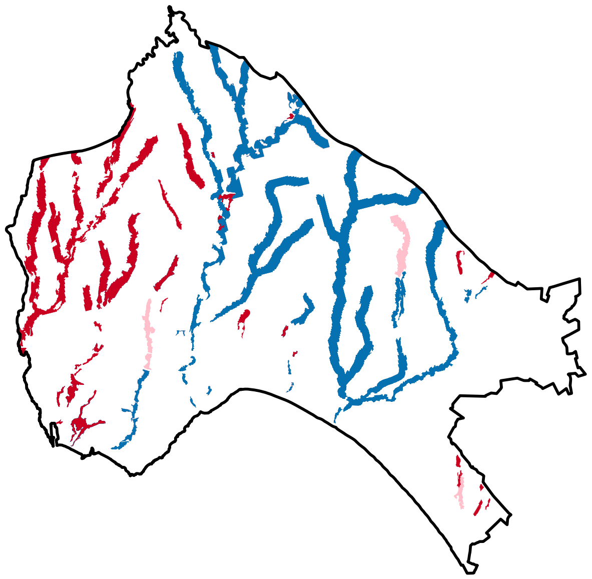

DISADVANTAGED COMMUNITIES

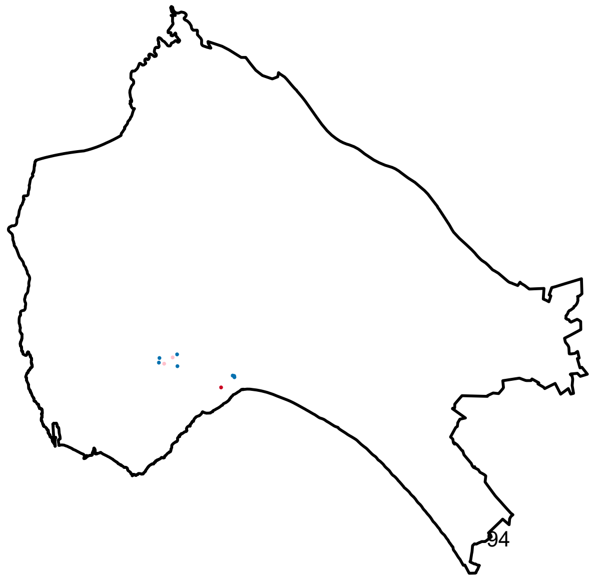

3−003.05 NORTH SAN BENITO

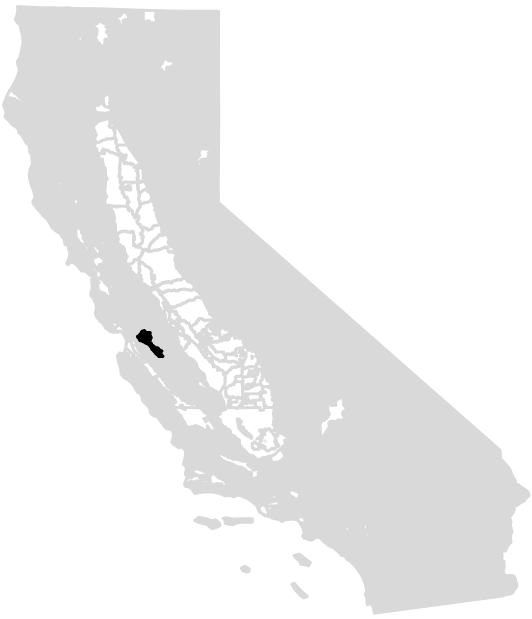

AGRICULTURE

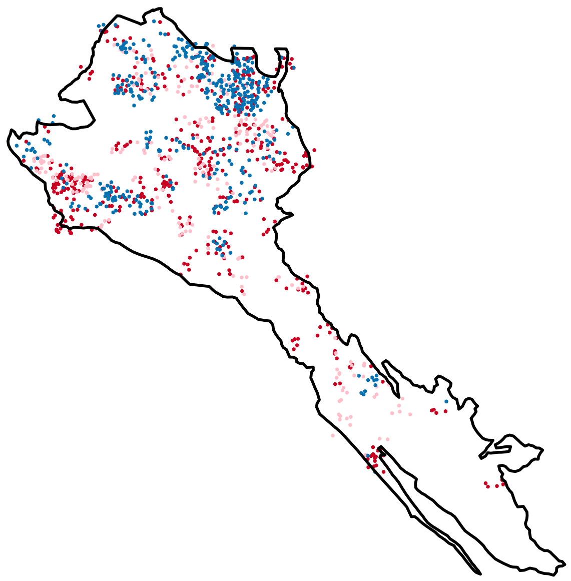

DOMESTIC

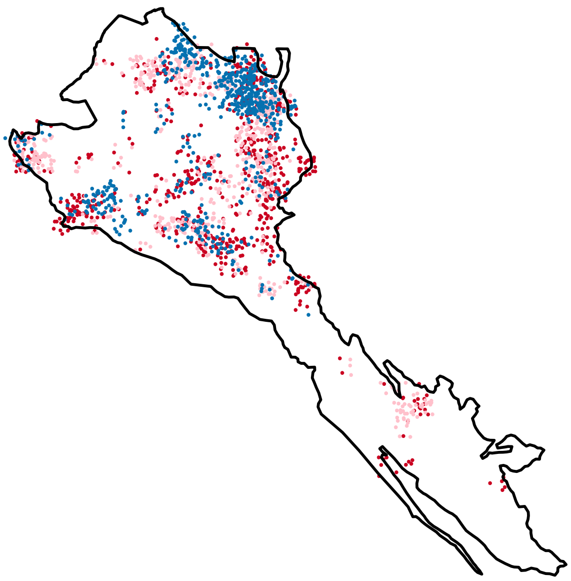

ENVIRONMENT

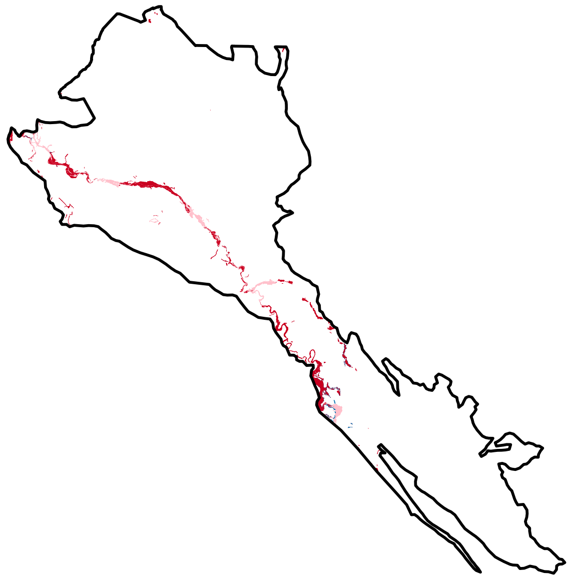

DISADVANTAGED COMMUNITIES

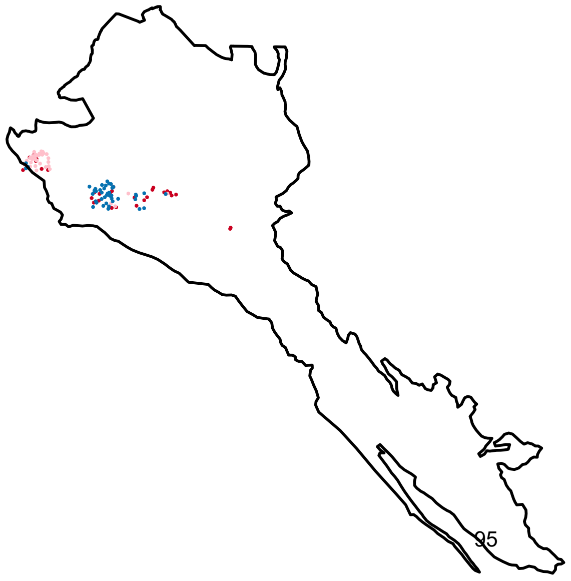

3−004.01 180−400 FOOT AQUIFER

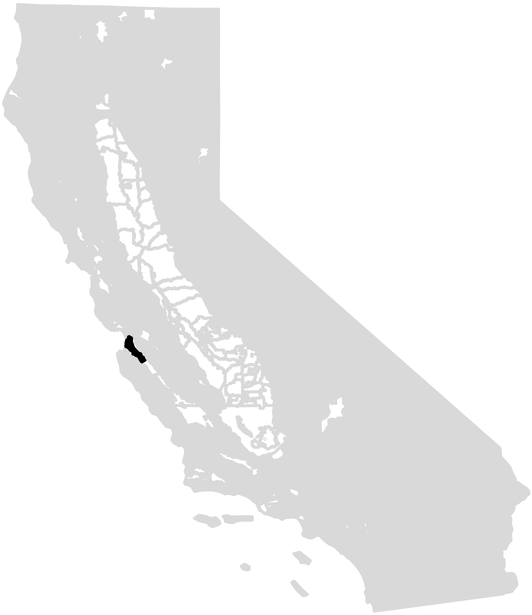

AGRICULTURE

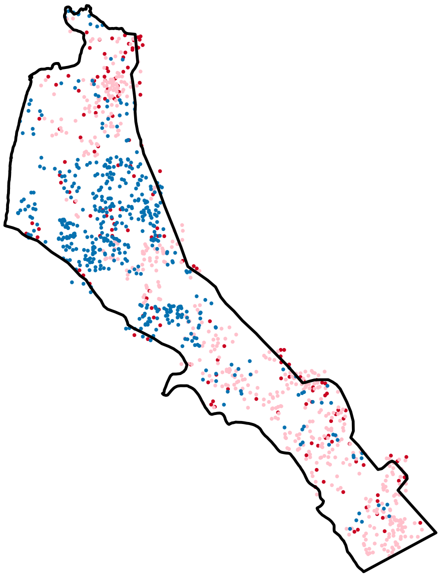

DOMESTIC

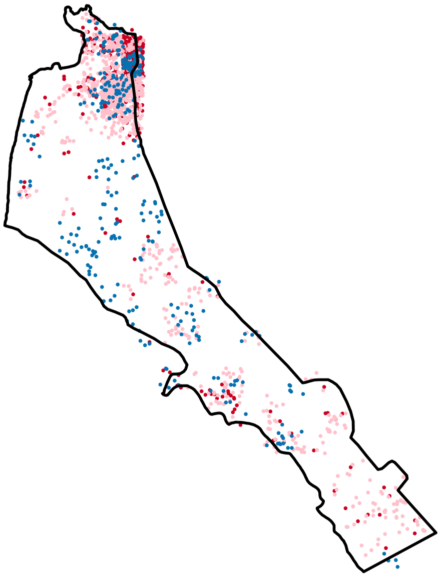

ENVIRONMENT

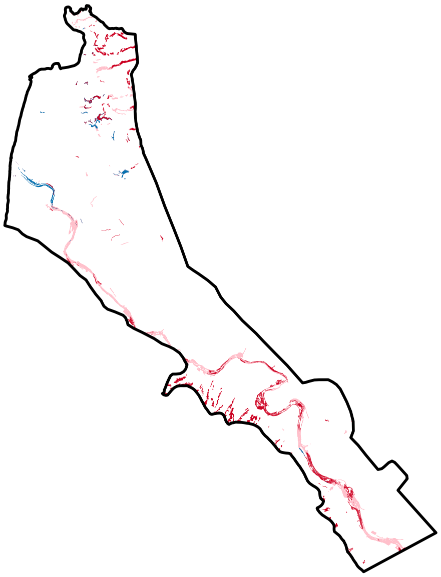

DISADVANTAGED COMMUNITIES

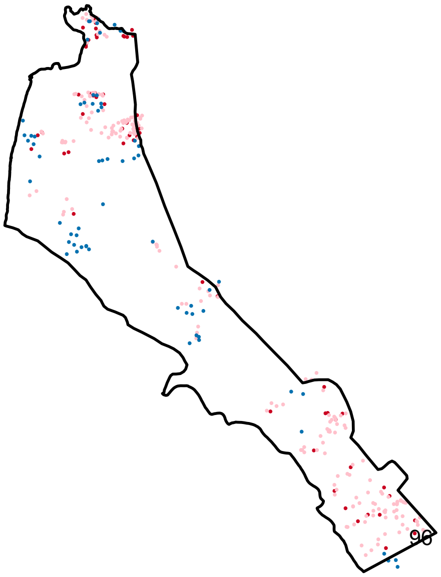

3−004.02 EAST SIDE AQUIFER

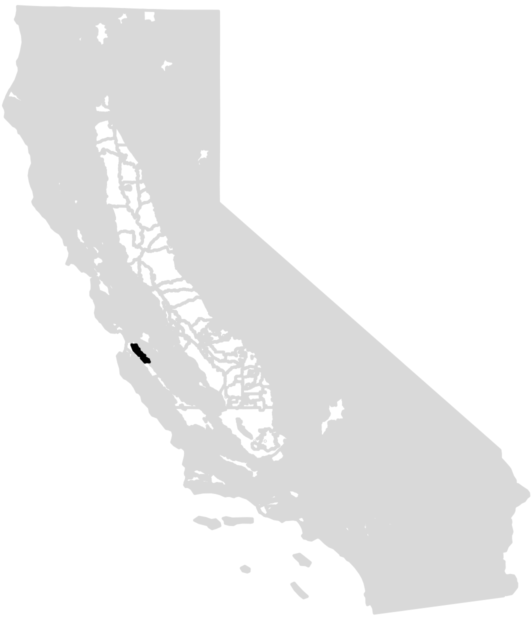

AGRICULTURE

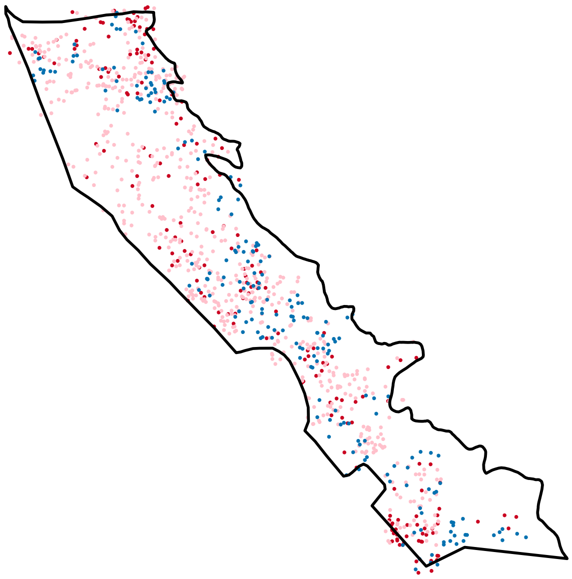

DOMESTIC

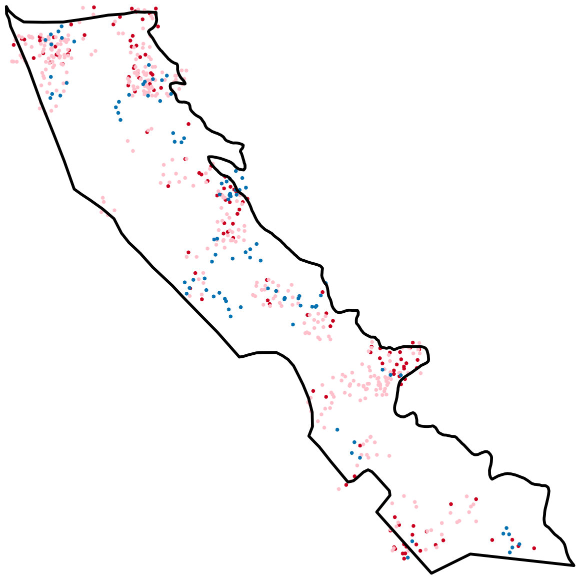

ENVIRONMENT

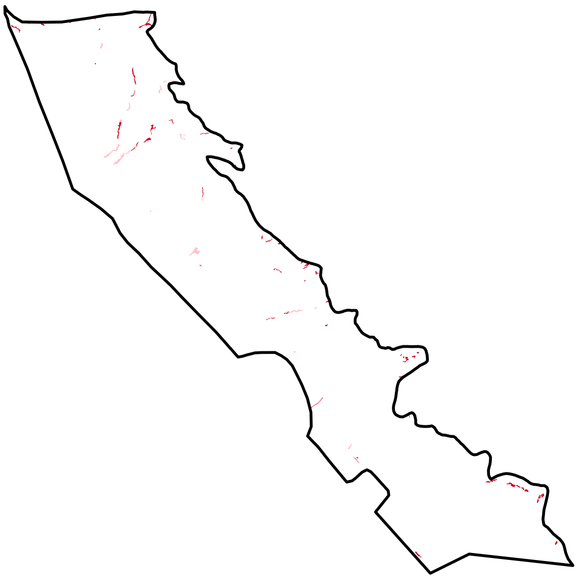

DISADVANTAGED COMMUNITIES

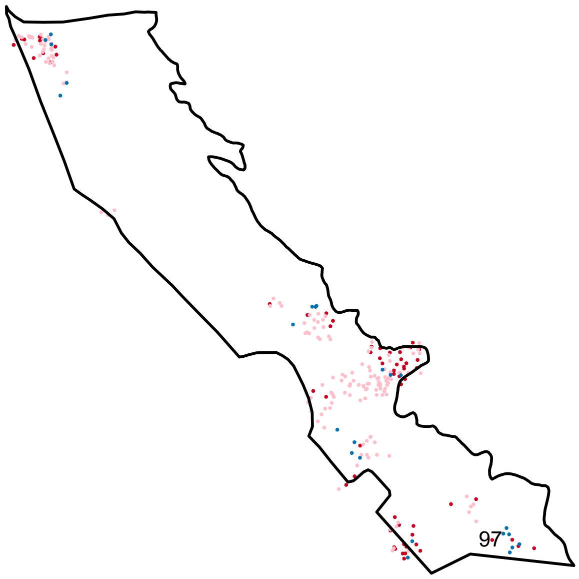

3−004.04 FOREBAY AQUIFER

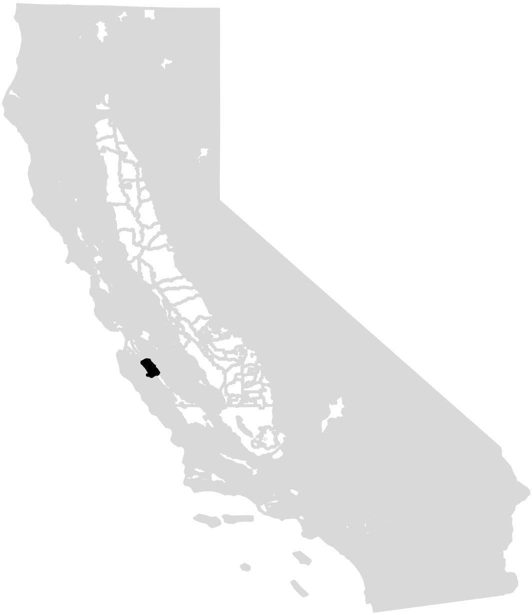

AGRICULTURE

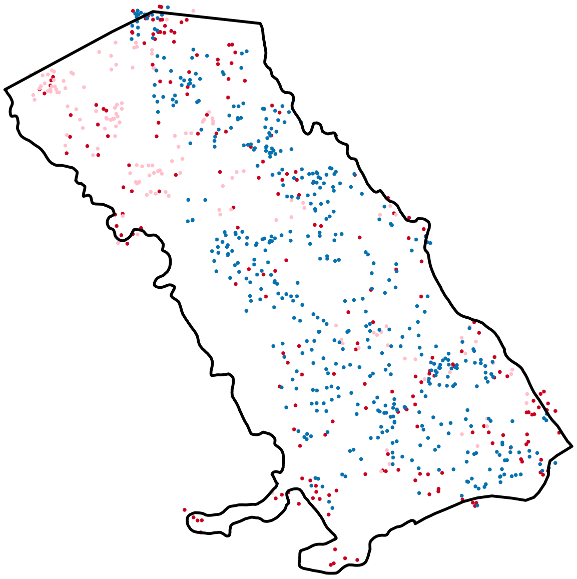

DOMESTIC

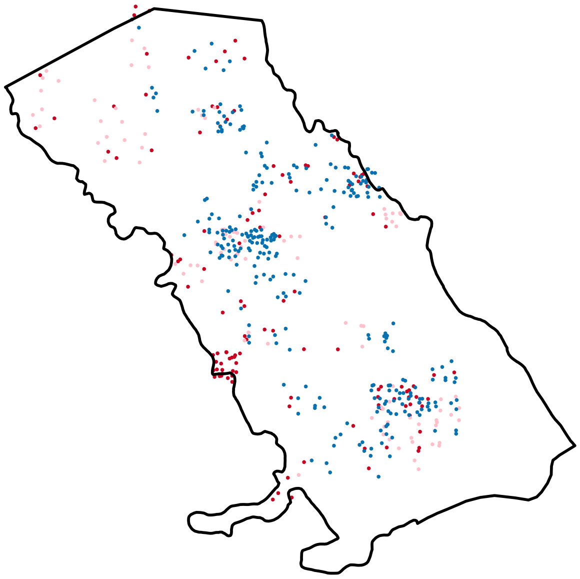

ENVIRONMENT

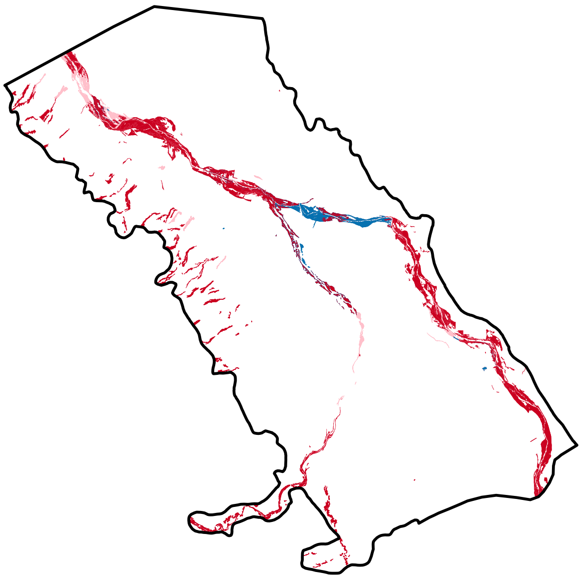

DISADVANTAGED COMMUNITIES

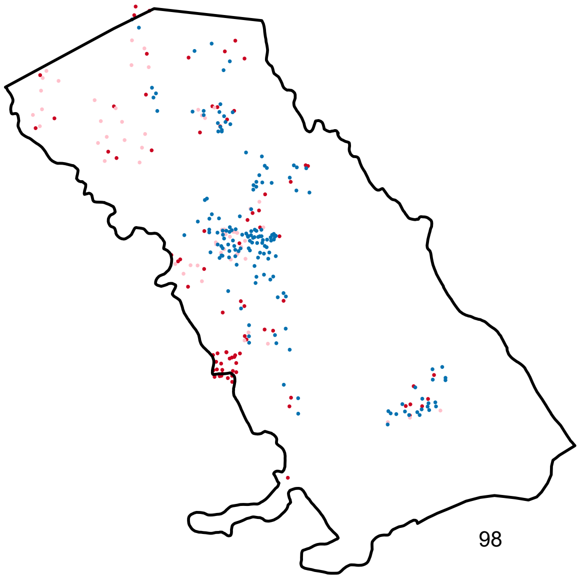

3−004.05 UPPER VALLEY AQUIFER

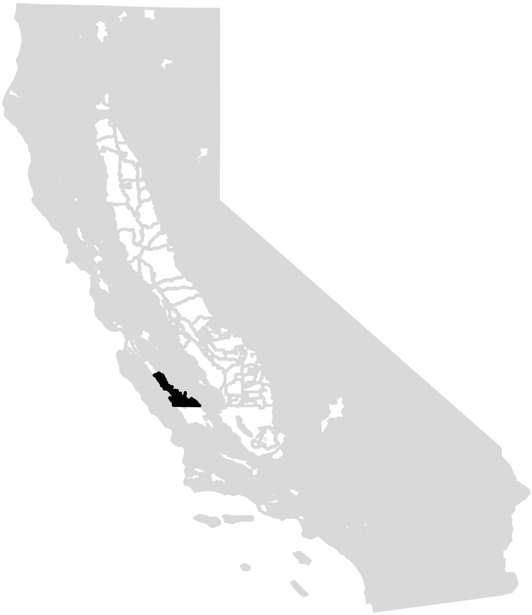

AGRICULTURE

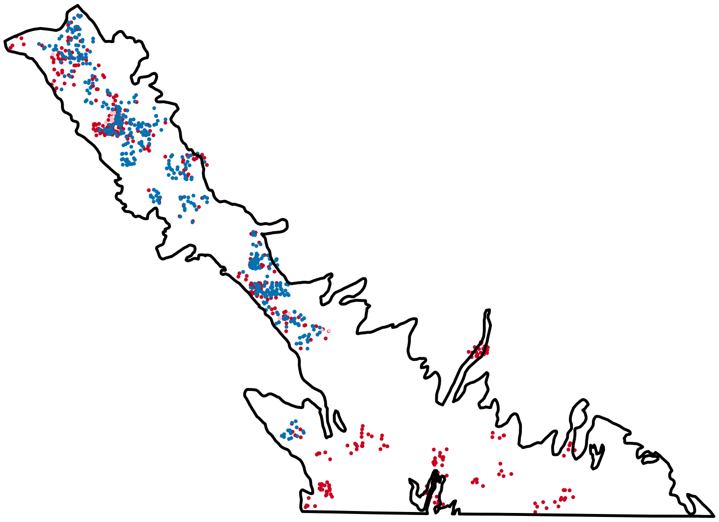

DOMESTIC

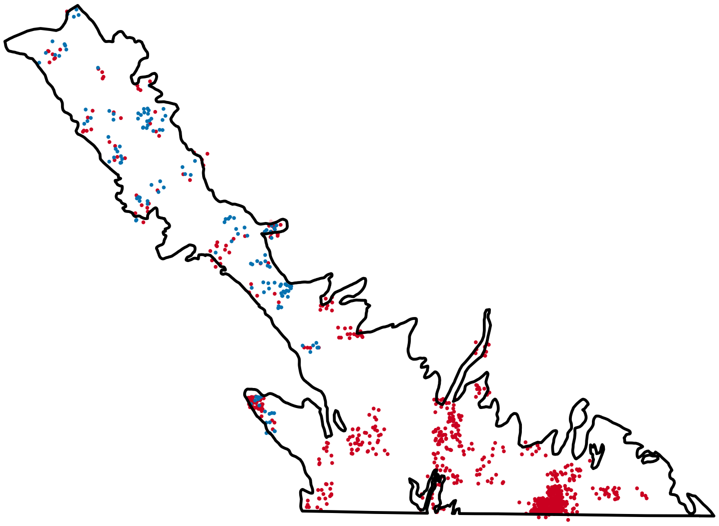

ENVIRONMENT

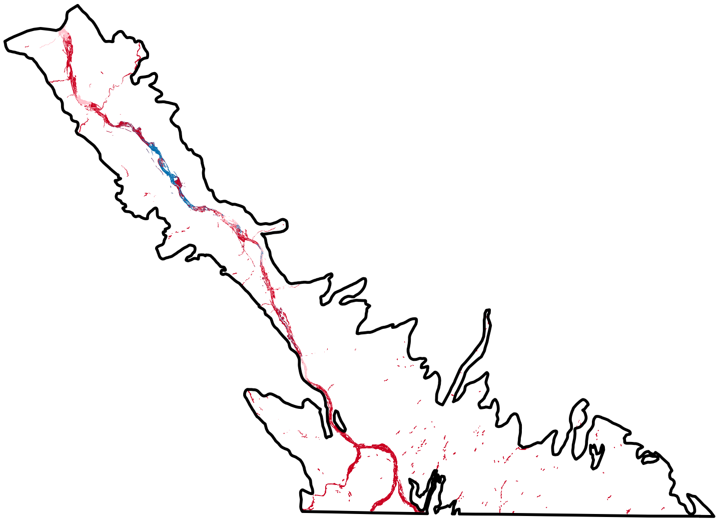

DISADVANTAGED COMMUNITIES

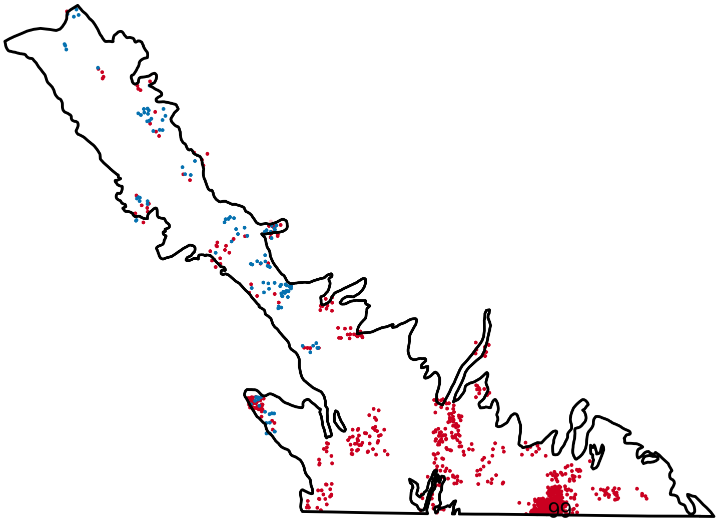

3−004.06 PASO ROBLES AREA

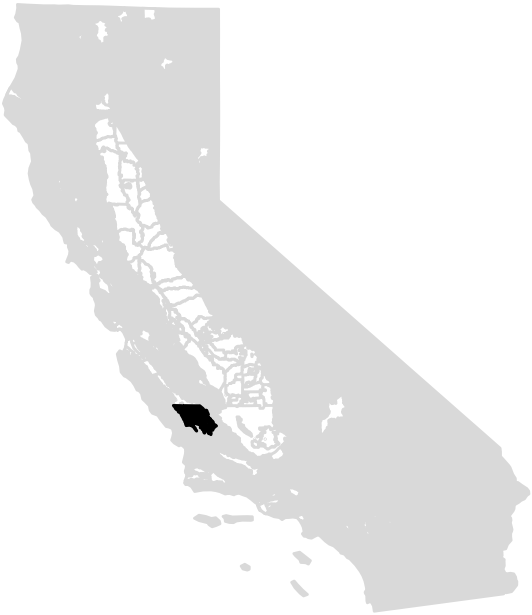

AGRICULTURE

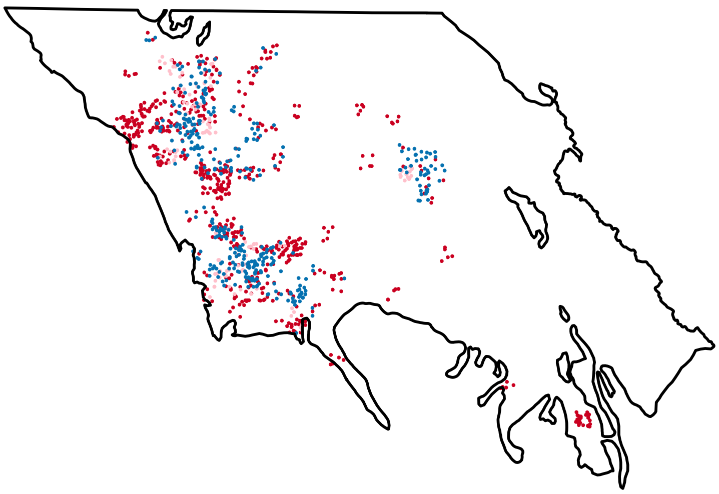

DOMESTIC

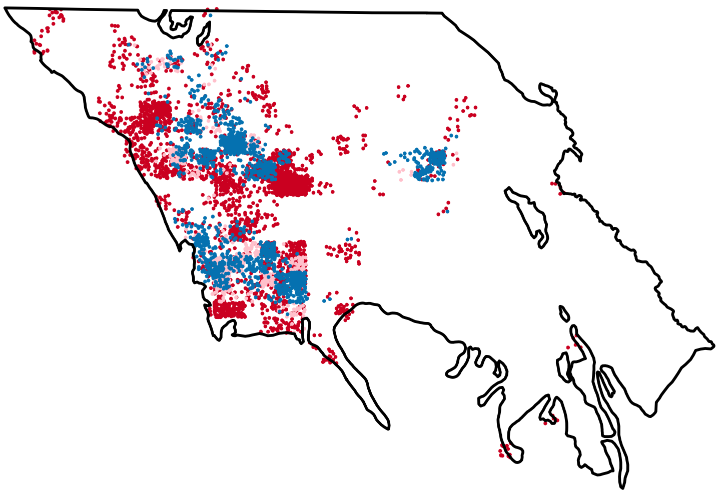

ENVIRONMENT

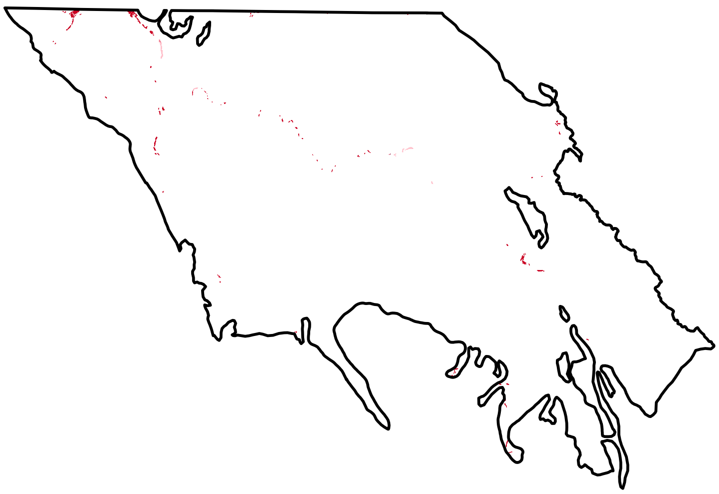

DISADVANTAGED COMMUNITIES

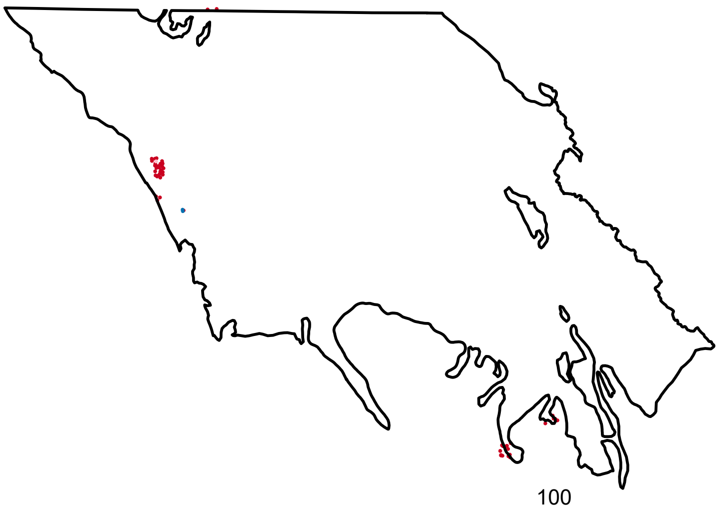

3−004.09 LANGLEY AREA

AGRICULTURE

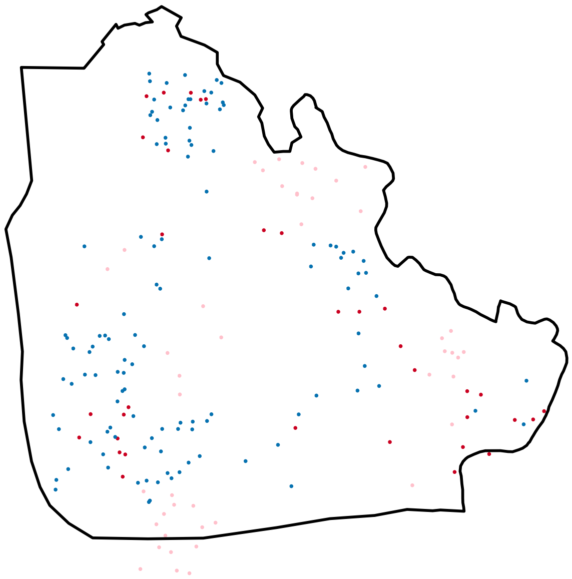

DOMESTIC

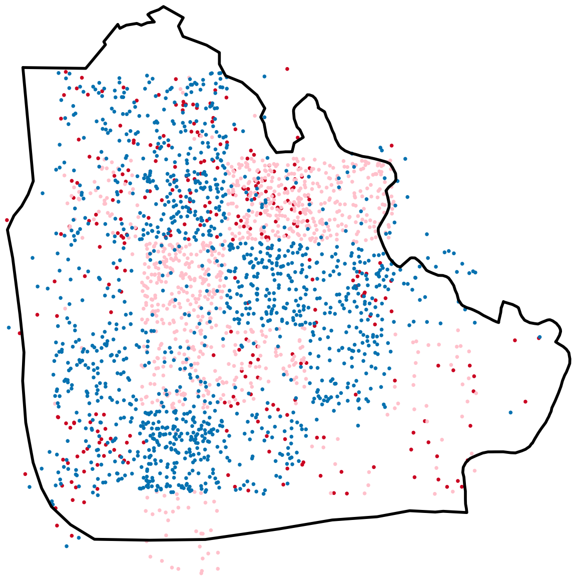

ENVIRONMENT

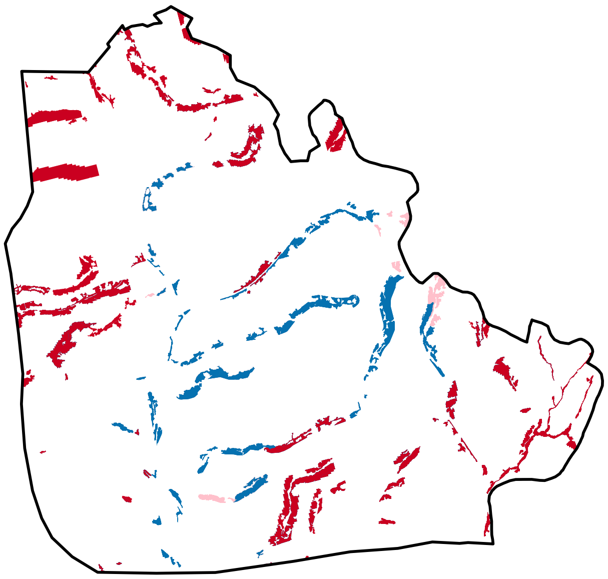

DISADVANTAGED COMMUNITIES

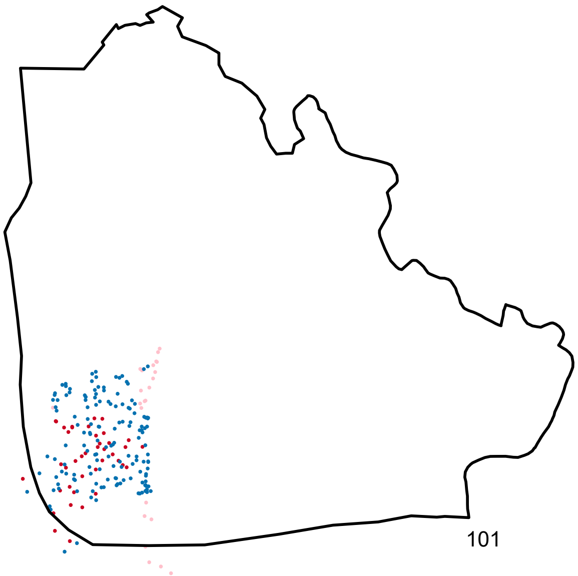

3−004.10 MONTEREY

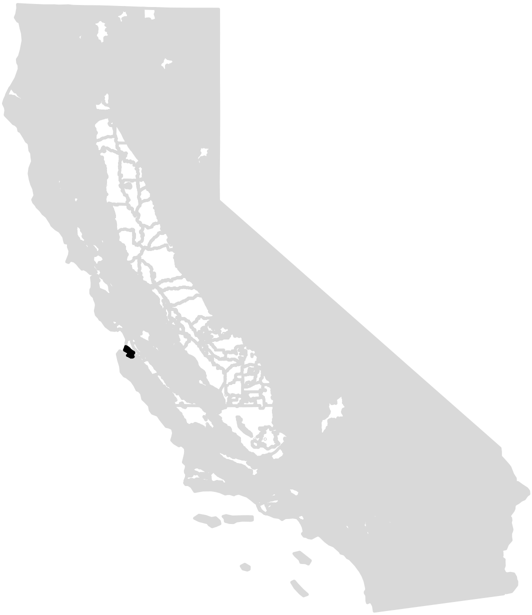

AGRICULTURE

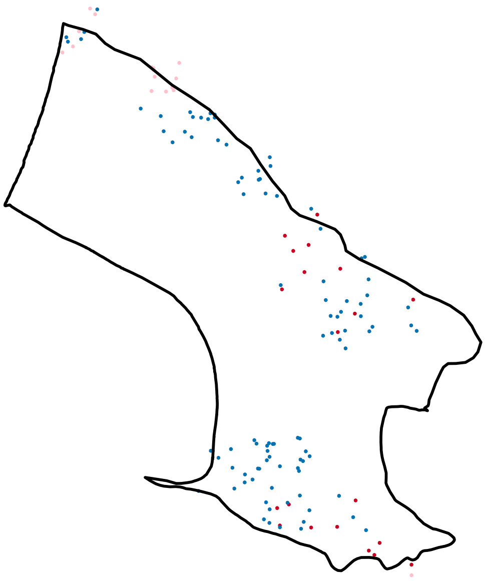

DOMESTIC

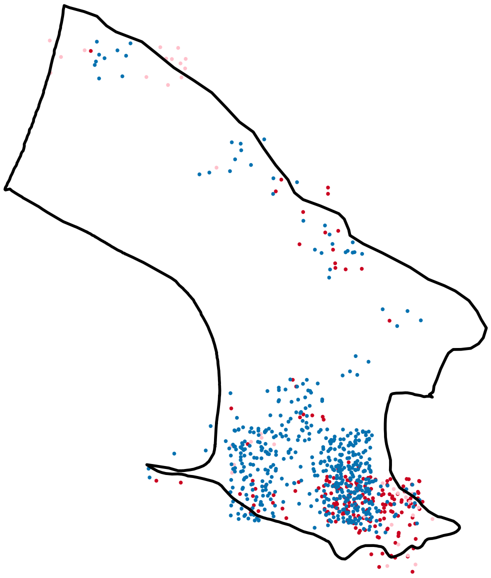

ENVIRONMENT

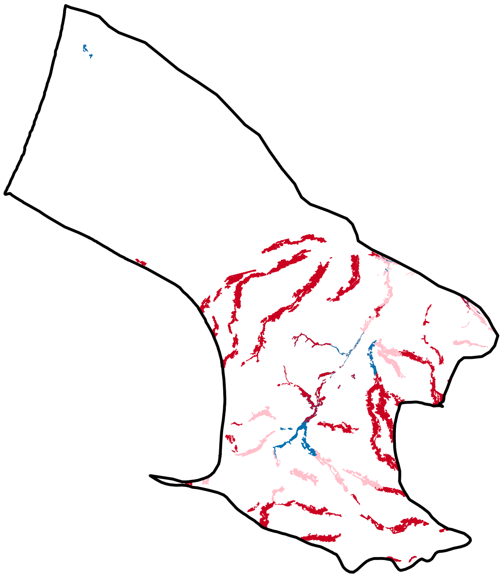

DISADVANTAGED COMMUNITIES

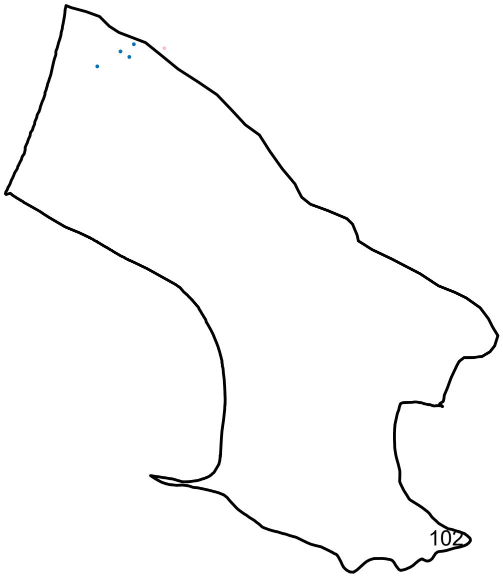

3−009 SAN LUIS OBISPO VALLEY

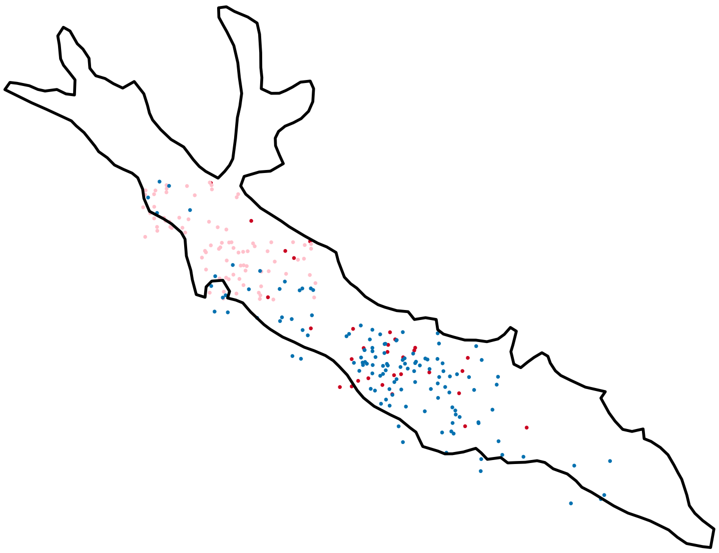

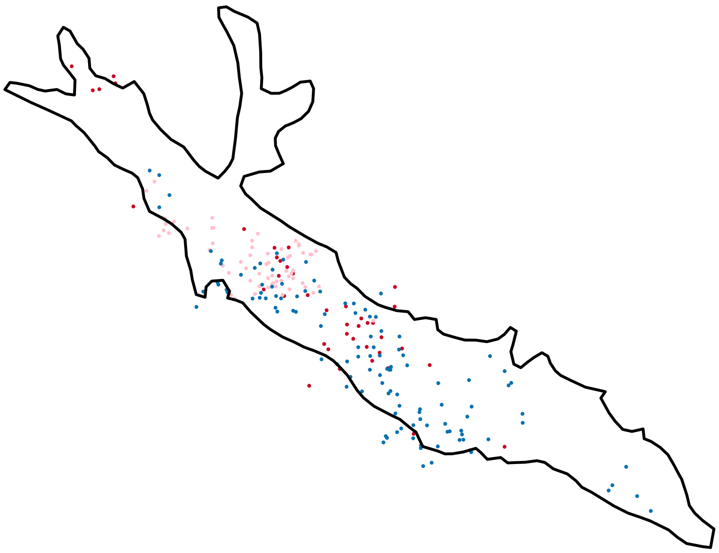

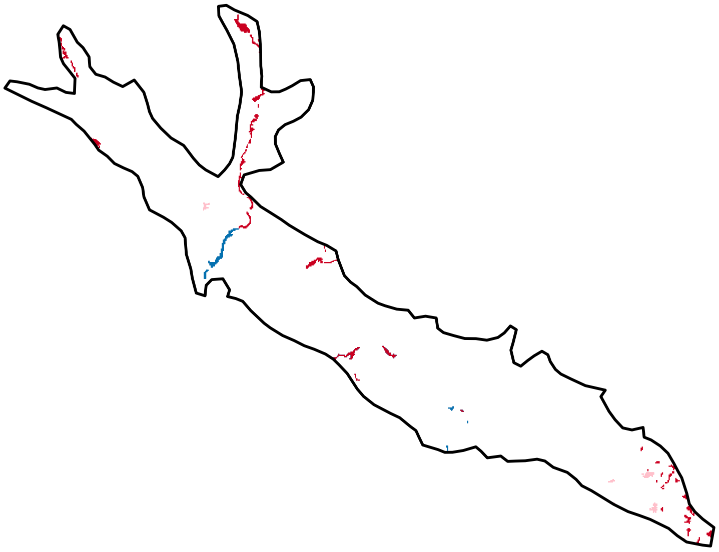

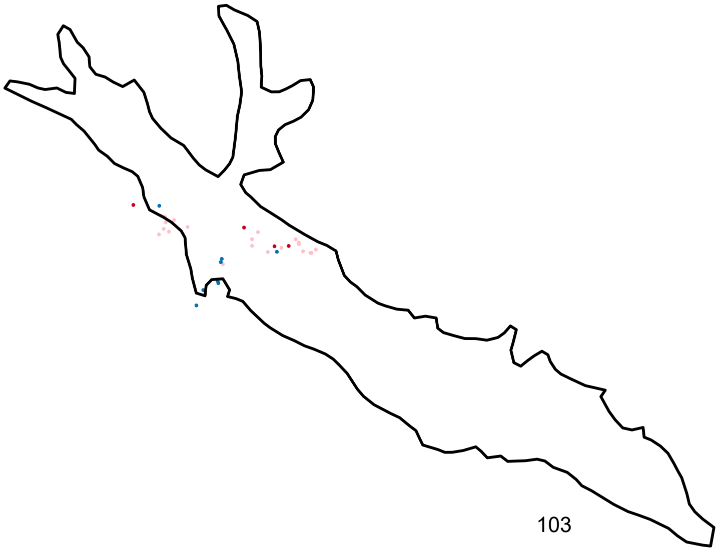

3−013 CUYAMA VALLEY

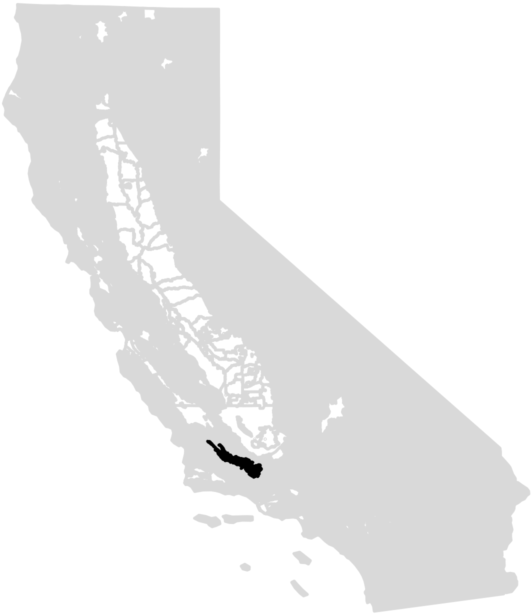

AGRICULTURE

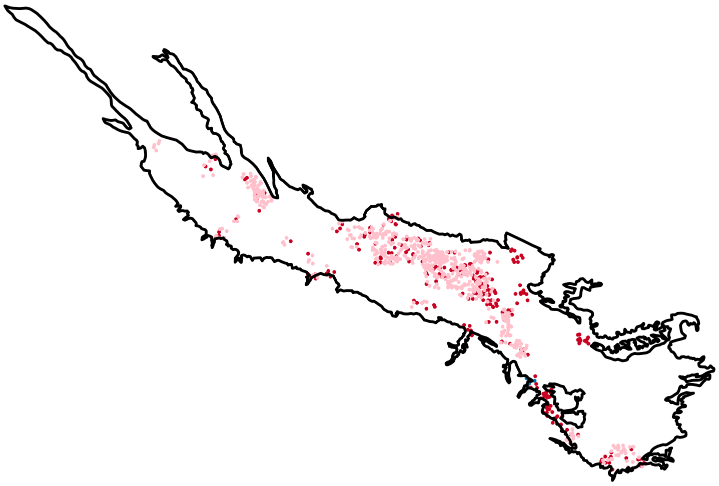

DOMESTIC

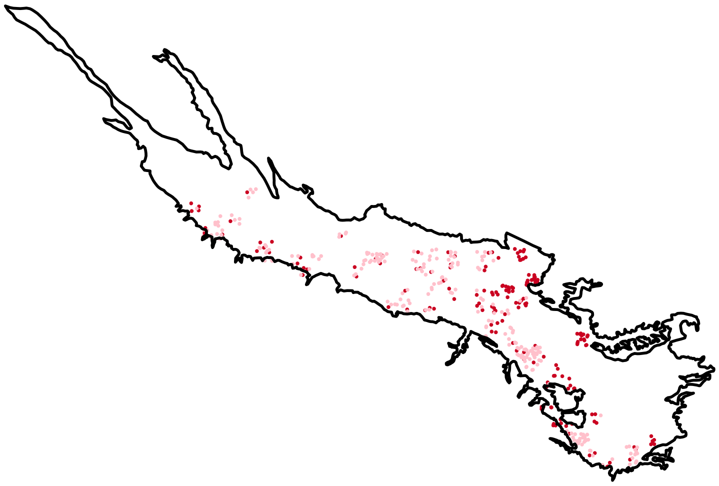

ENVIRONMENT

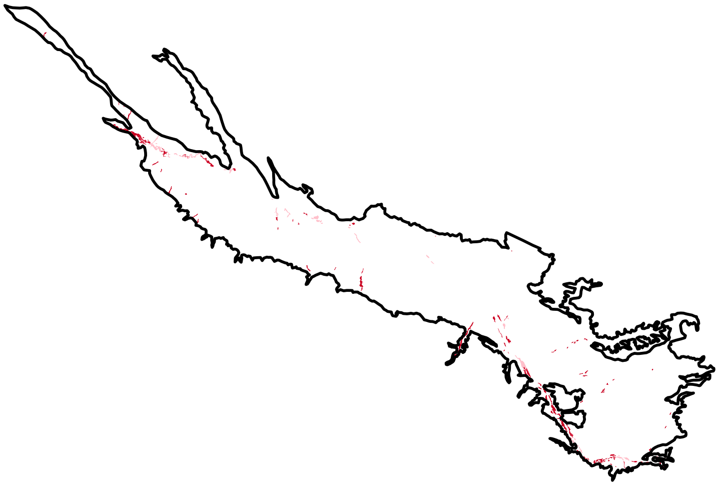

DISADVANTAGED COMMUNITIES

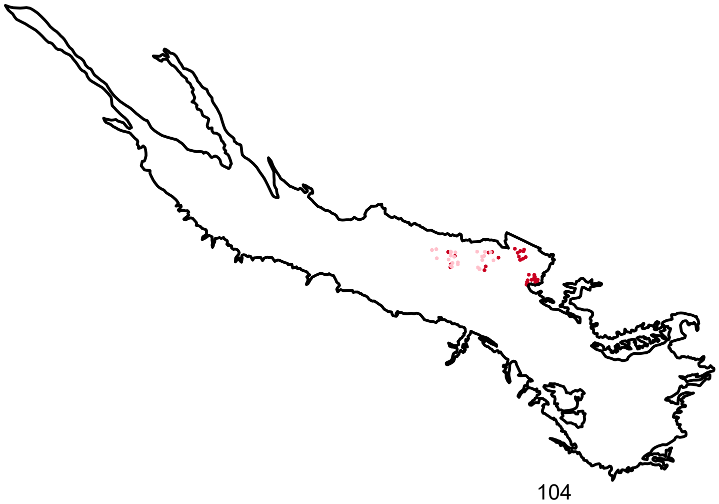

3−014 SAN ANTONIO CREEK VALLEY

AGRICULTURE

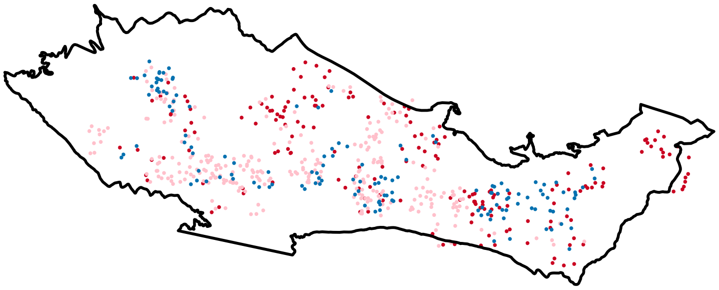

DOMESTIC

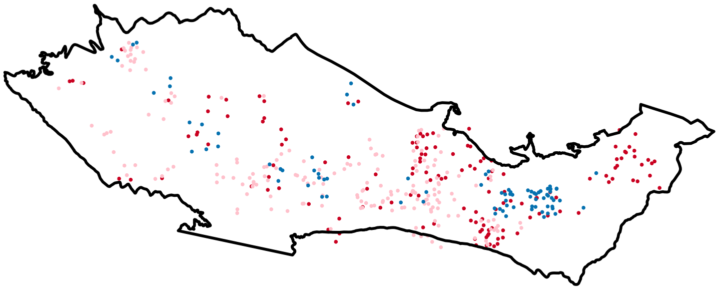

ENVIRONMENT

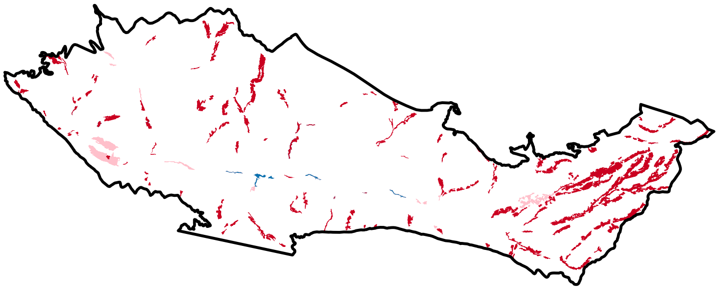

DISADVANTAGED COMMUNITIES

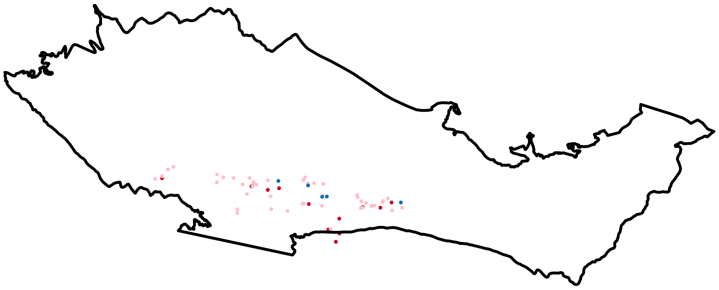

3−015 SANTA YNEZ RIVER VALLEY

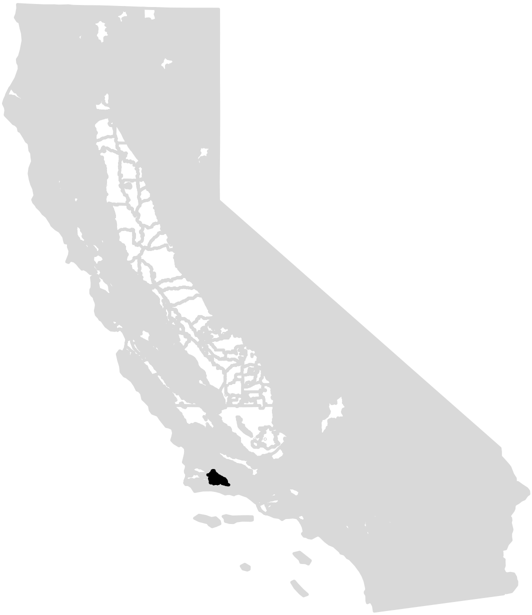

AGRICULTURE

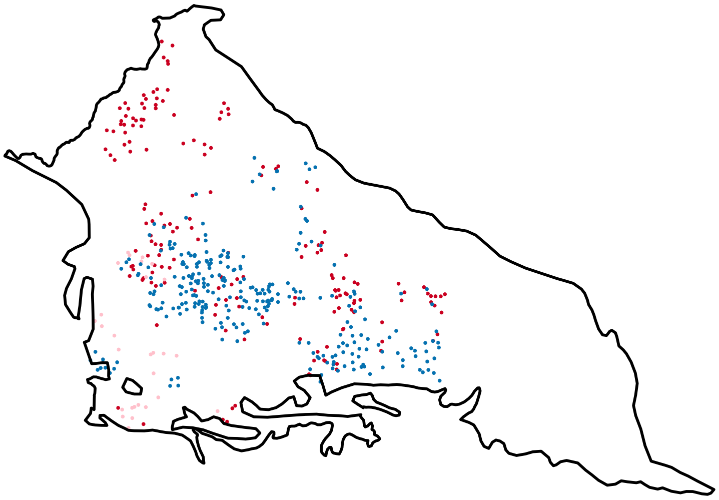

DOMESTIC

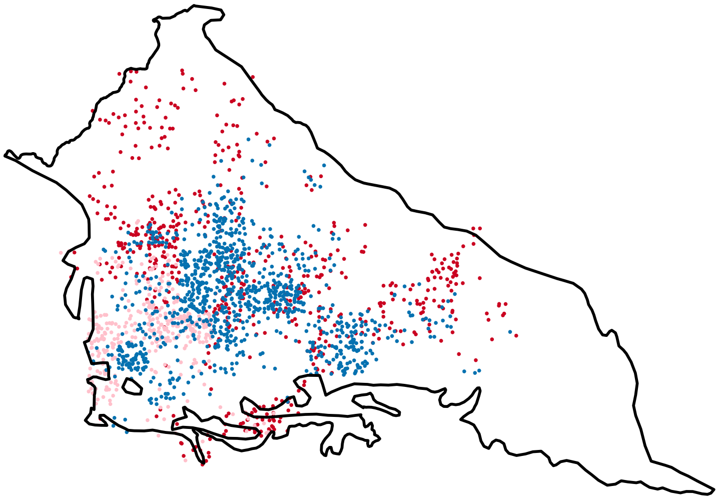

ENVIRONMENT

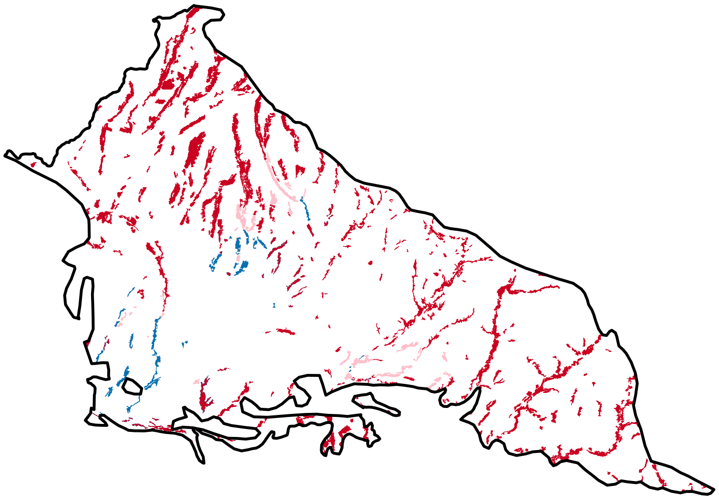

DISADVANTAGED COMMUNITIES

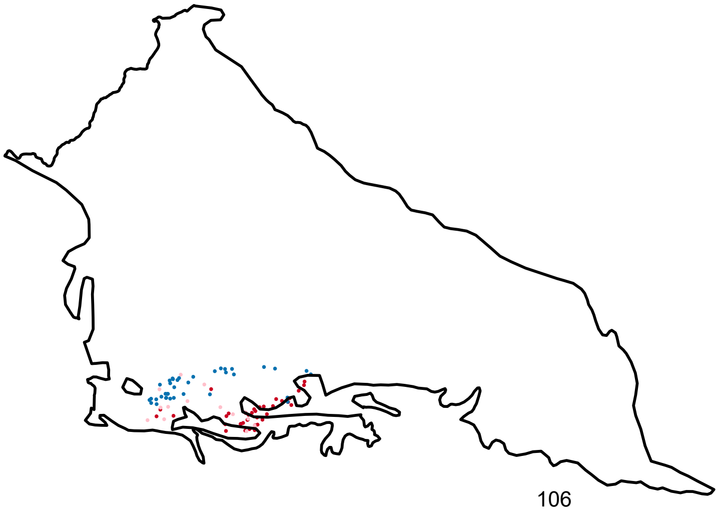

3−027 SANTA MARGARITA

AGRICULTURE

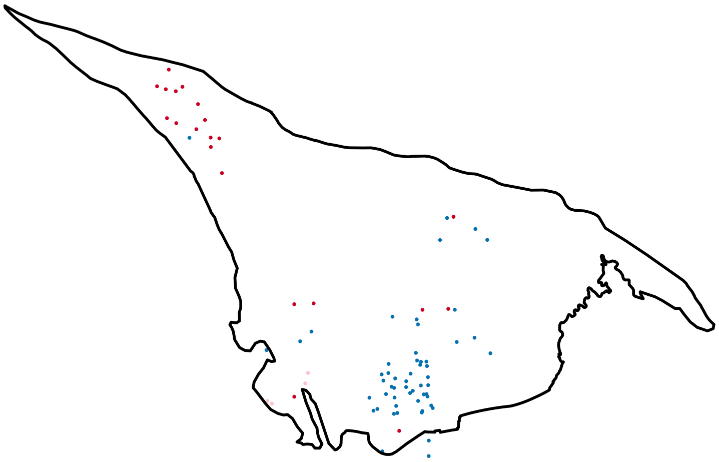

DOMESTIC

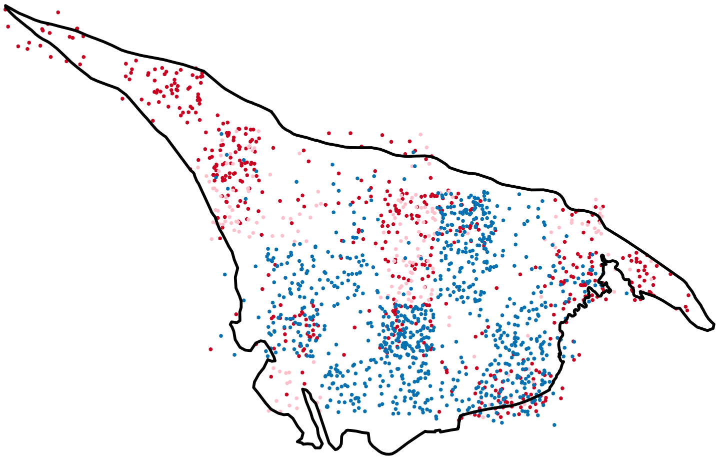

ENVIRONMENT

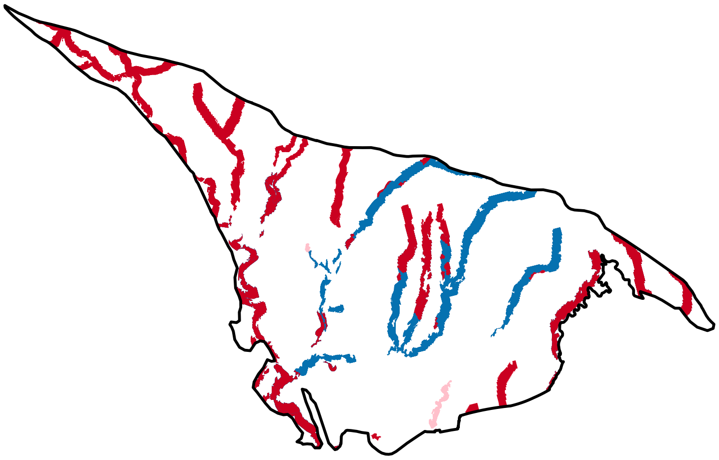

DISADVANTAGED COMMUNITIES

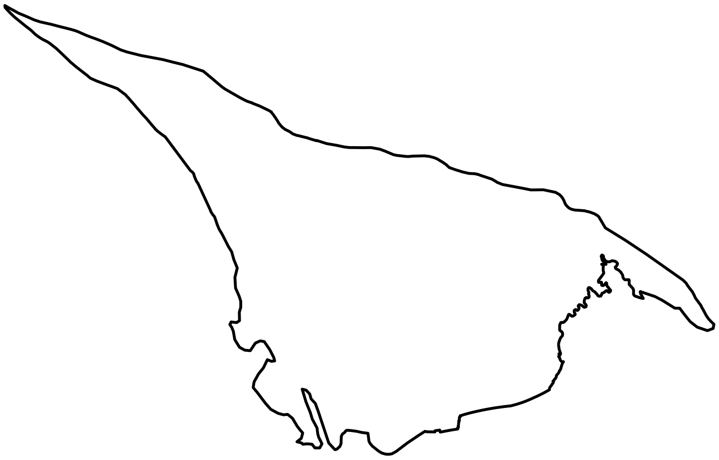

4−002 OJAI VALLEY

AGRICULTURE

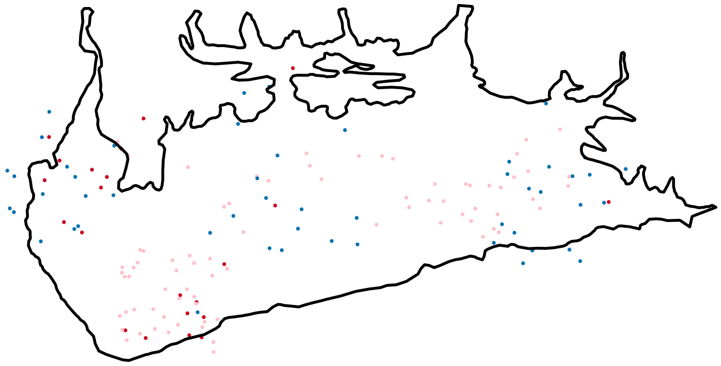

DOMESTIC

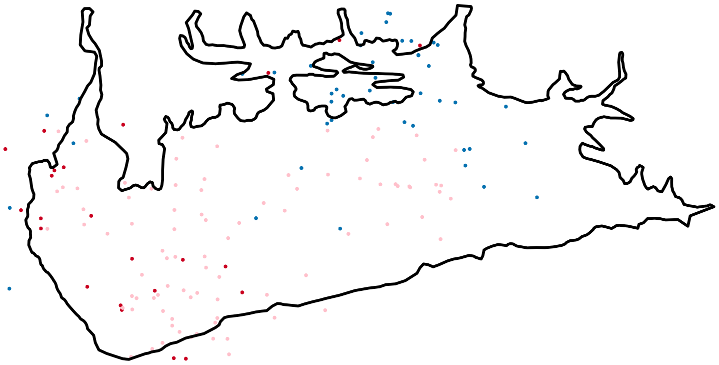

ENVIRONMENT

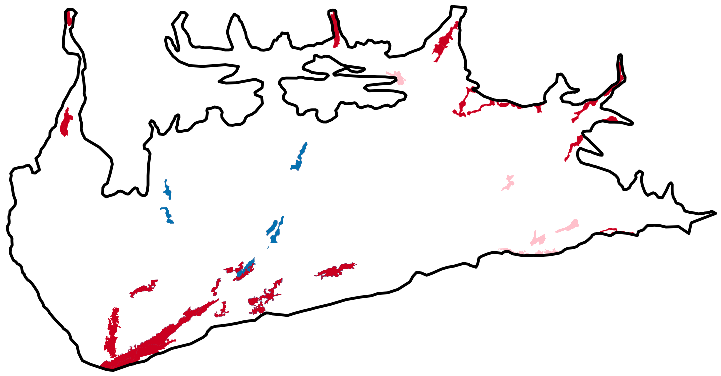

DISADVANTAGED COMMUNITIES

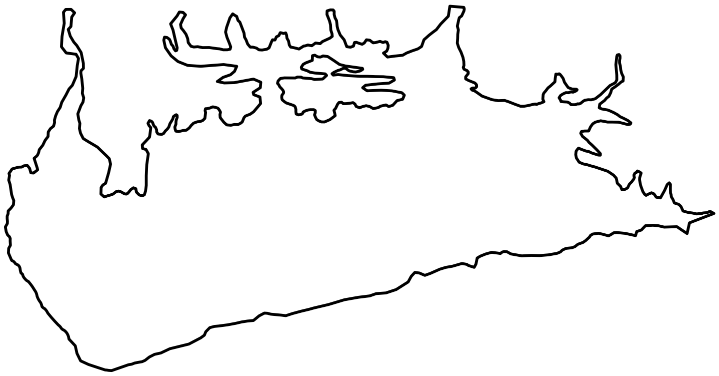

4−003.01 UPPER VENTURA RIVER

AGRICULTURE

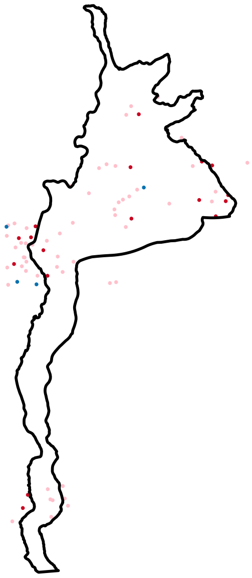

DOMESTIC

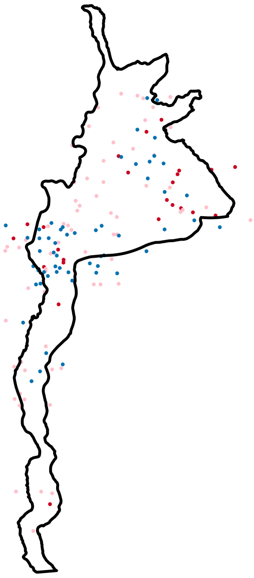

ENVIRONMENT

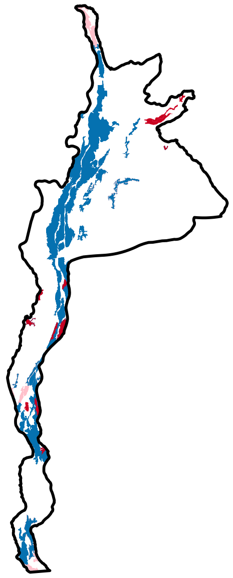

DISADVANTAGED COMMUNITIES

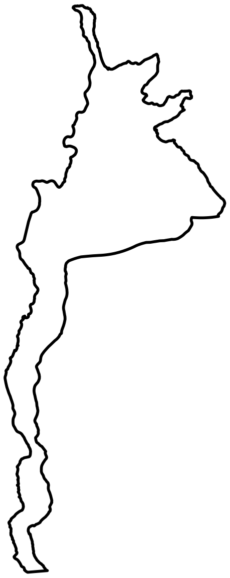

4−004.02 OXNARD

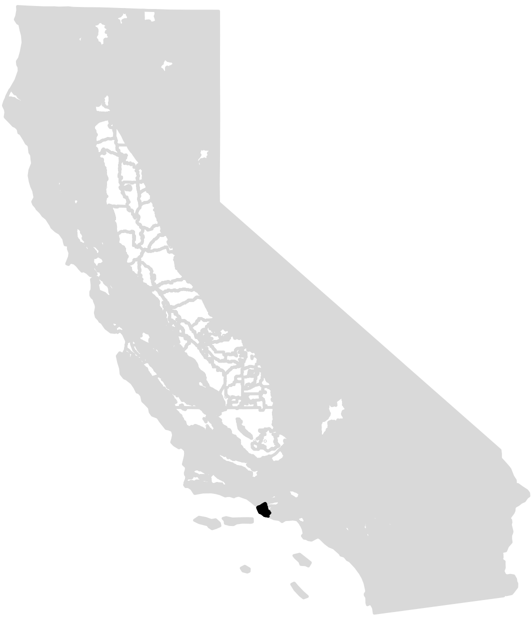

AGRICULTURE

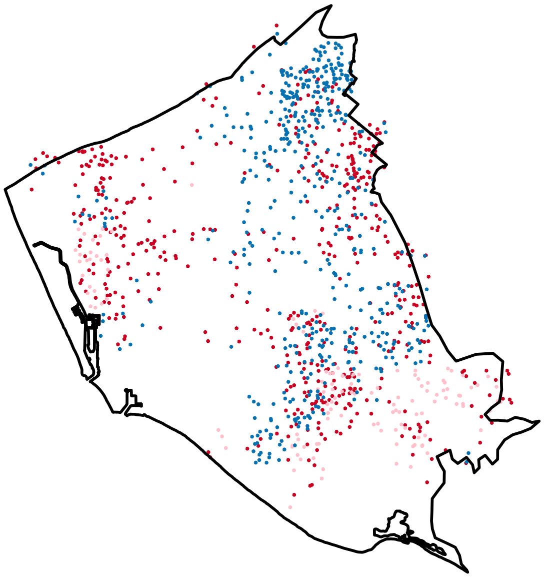

DOMESTIC

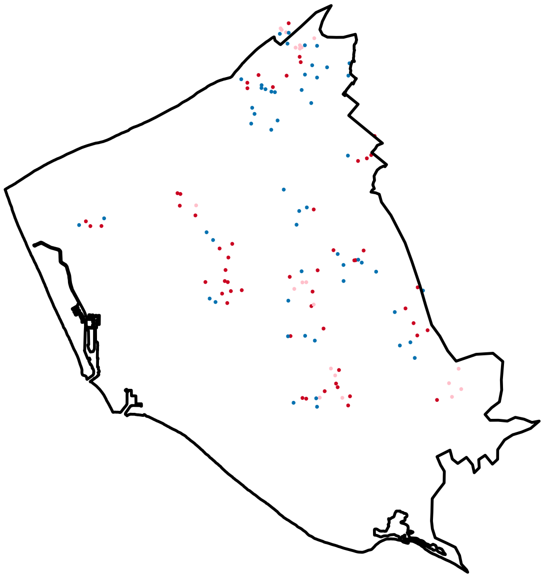

ENVIRONMENT

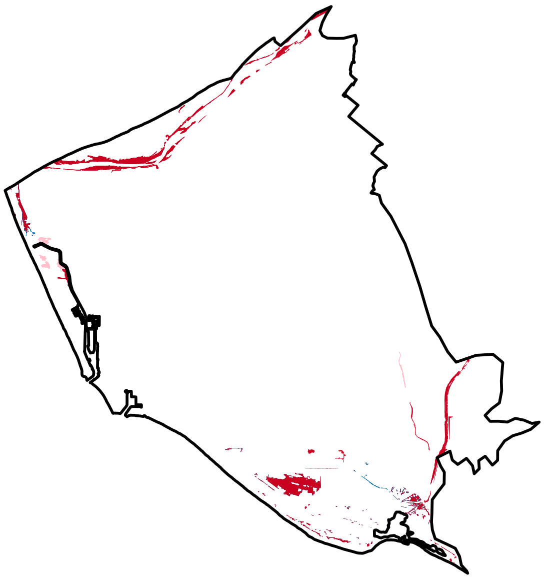

DISADVANTAGED COMMUNITIES

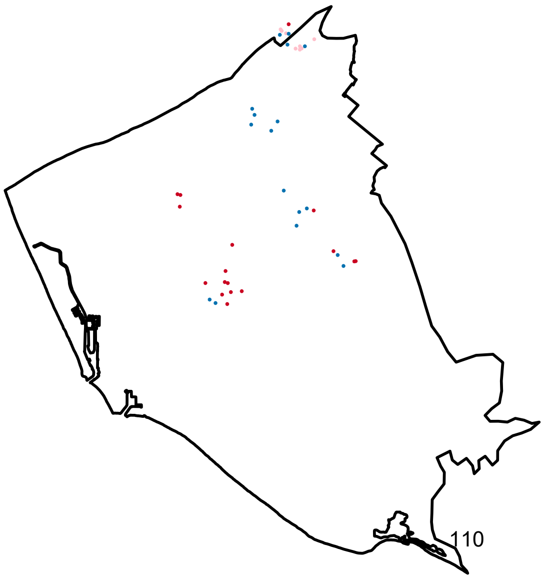

4−004.03 MOUND

AGRICULTURE

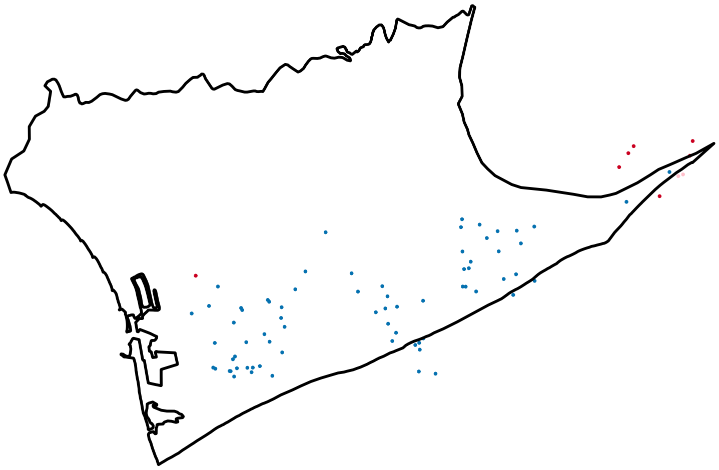

DOMESTIC

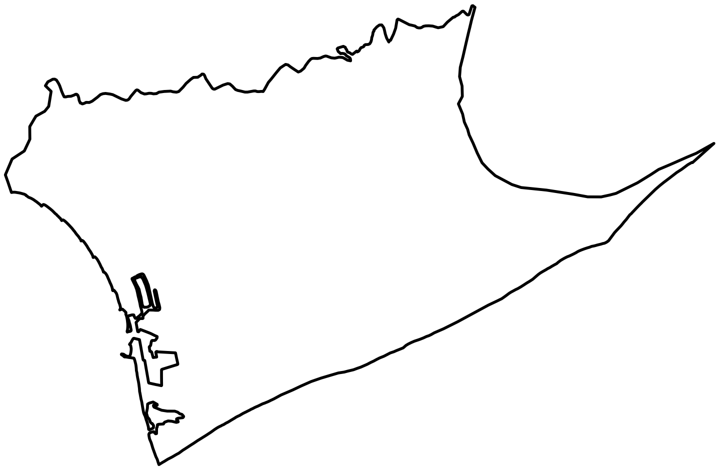

ENVIRONMENT

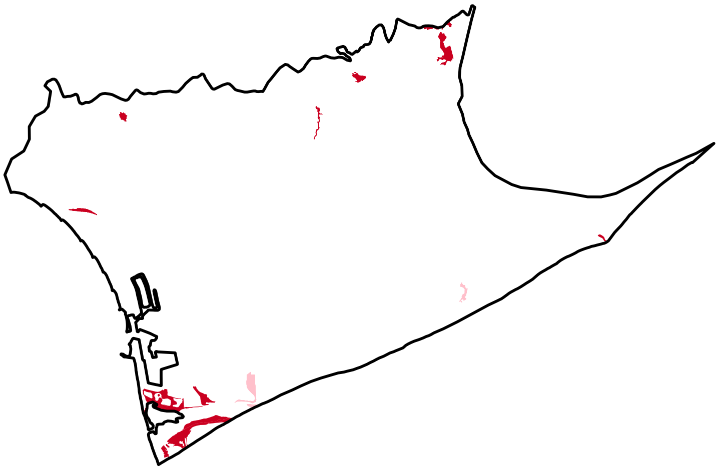

DISADVANTAGED COMMUNITIES

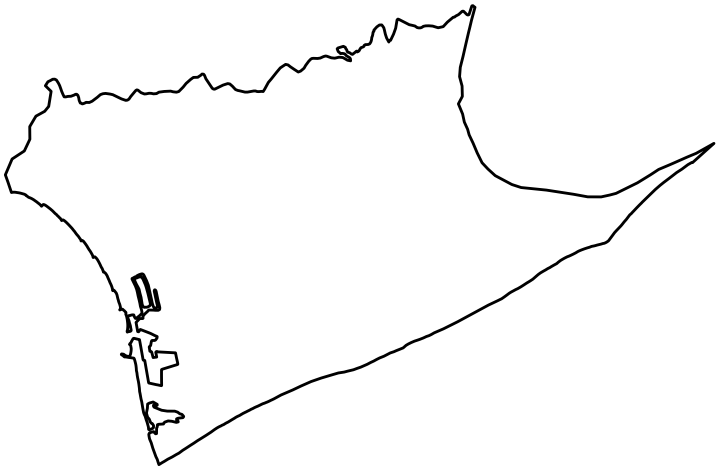

4−004.05 FILLMORE

AGRICULTURE

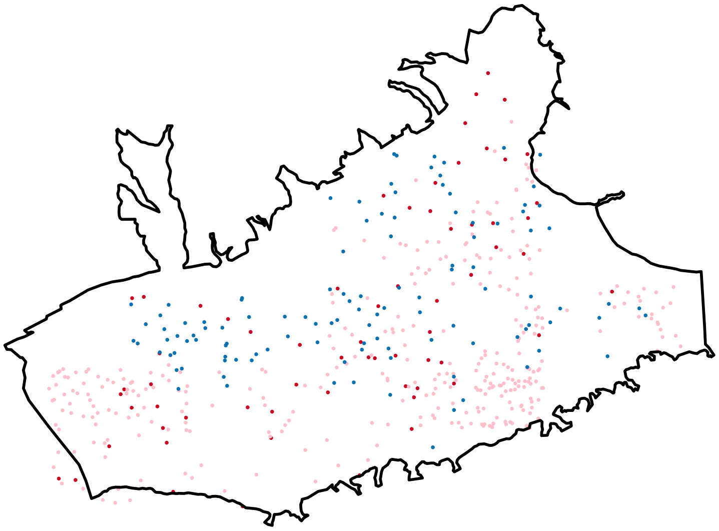

DOMESTIC

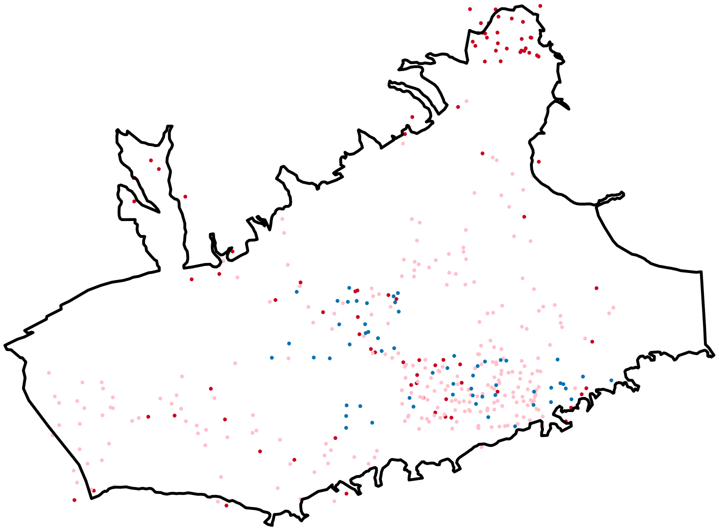

ENVIRONMENT

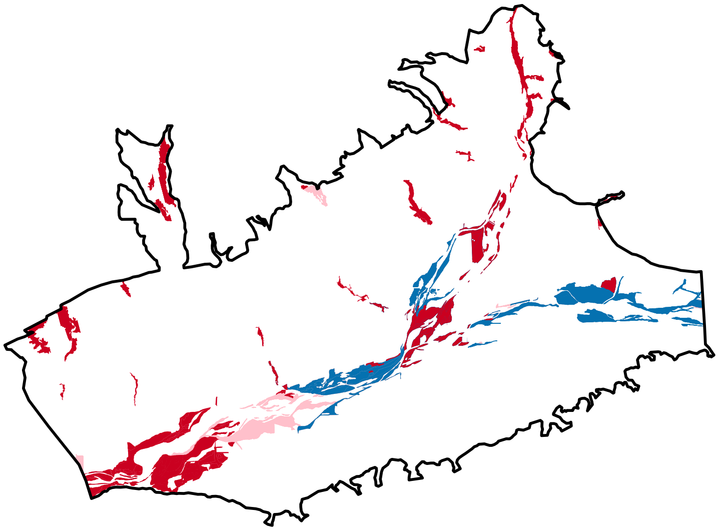

DISADVANTAGED COMMUNITIES

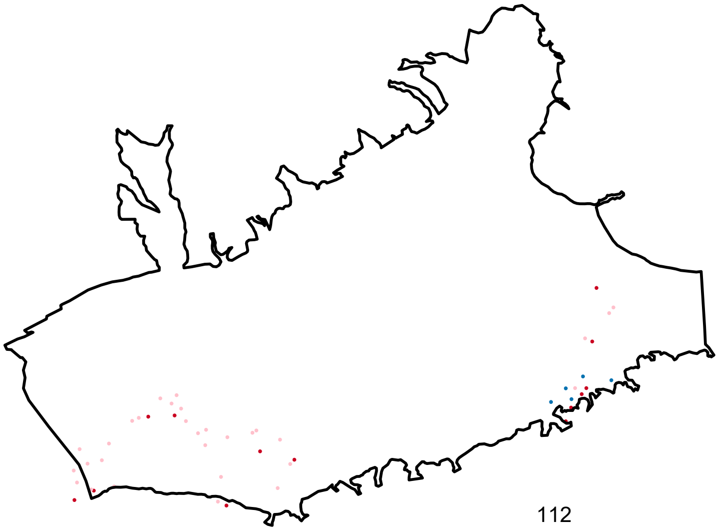

4−004.06 PIRU

AGRICULTURE

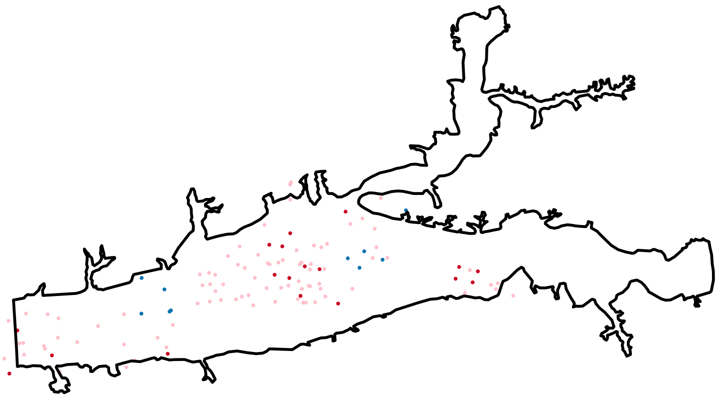

DOMESTIC

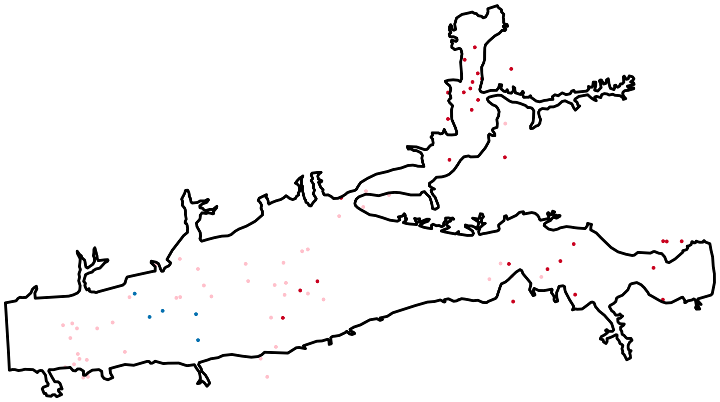

ENVIRONMENT

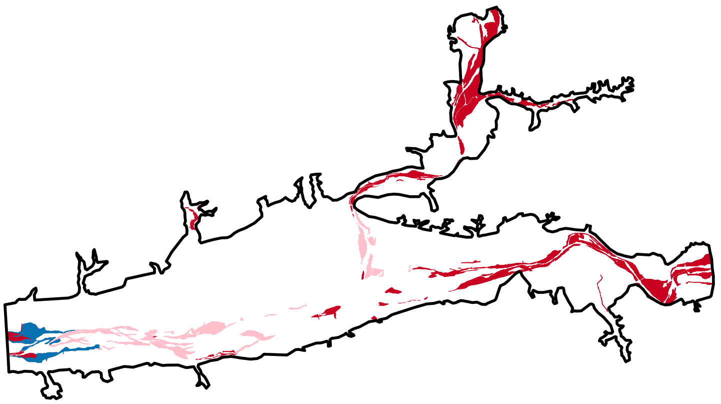

DISADVANTAGED COMMUNITIES

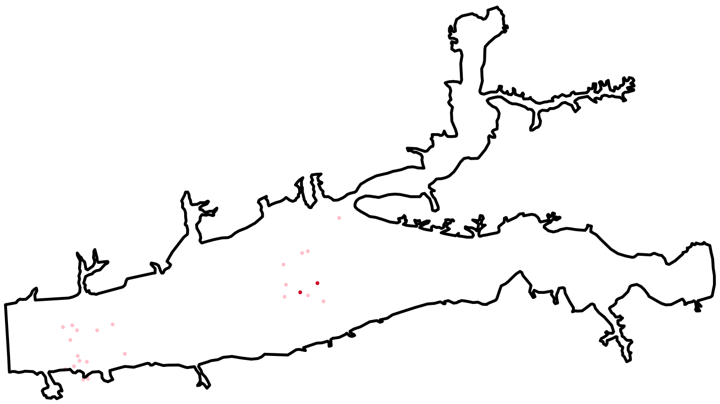

004.07 SANTA CLARA RIVER VALLEY EAS

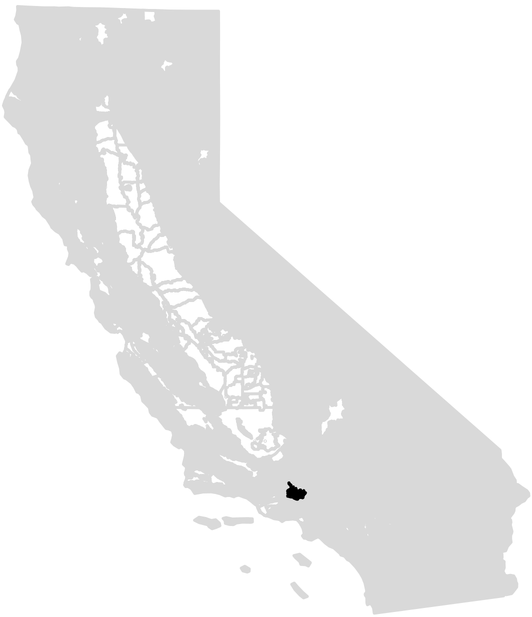

AGRICULTURE

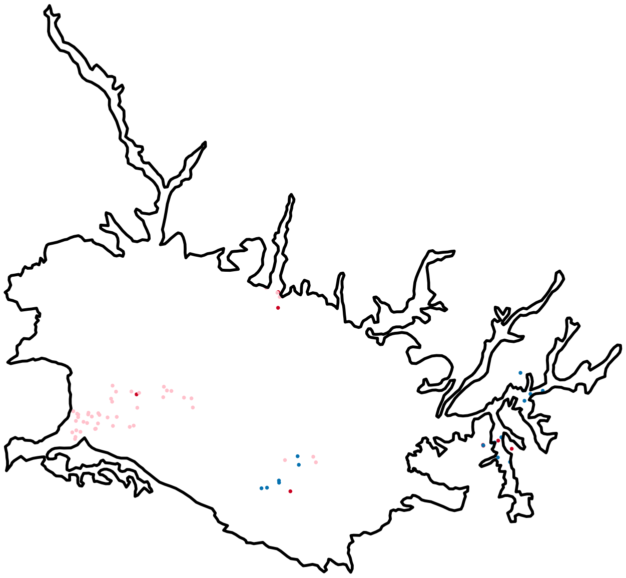

DOMESTIC

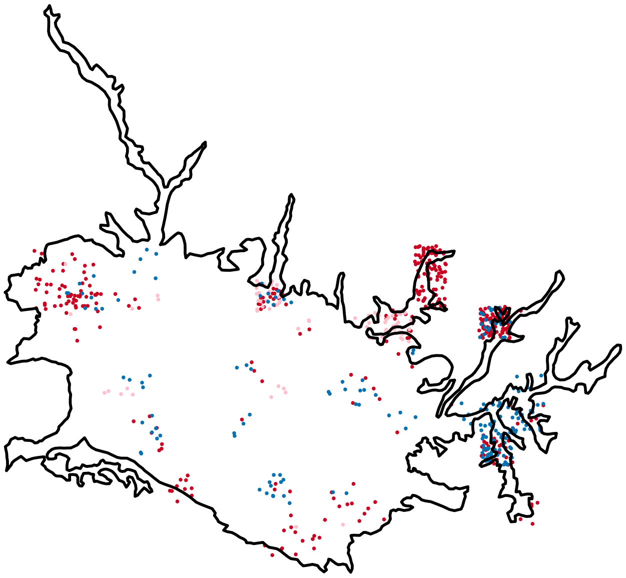

ENVIRONMENT

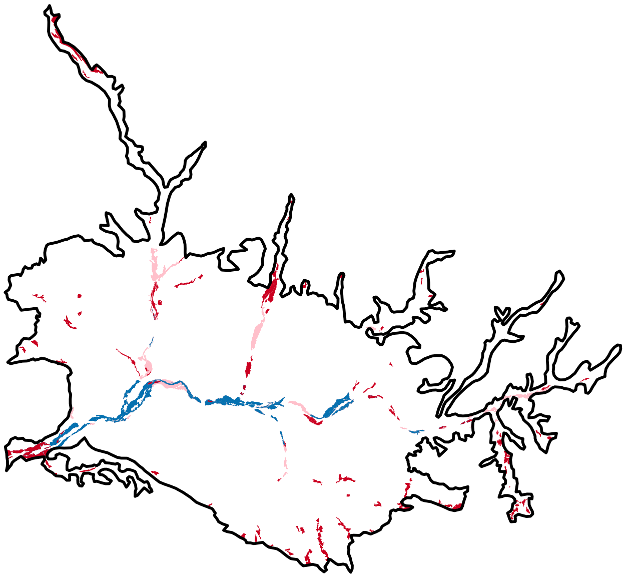

DISADVANTAGED COMMUNITIES

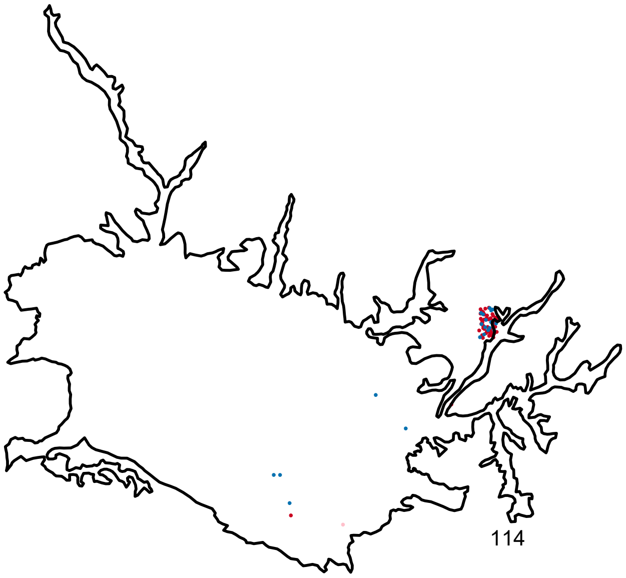

4−006 PLEASANT VALLEY

AGRICULTURE

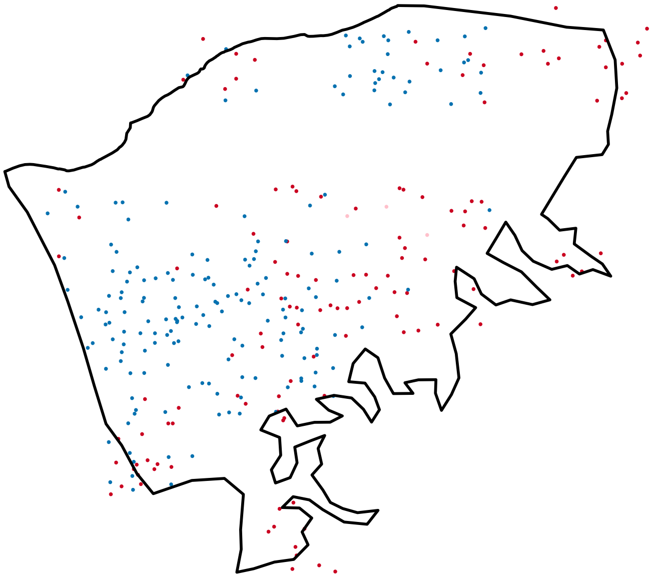

DOMESTIC

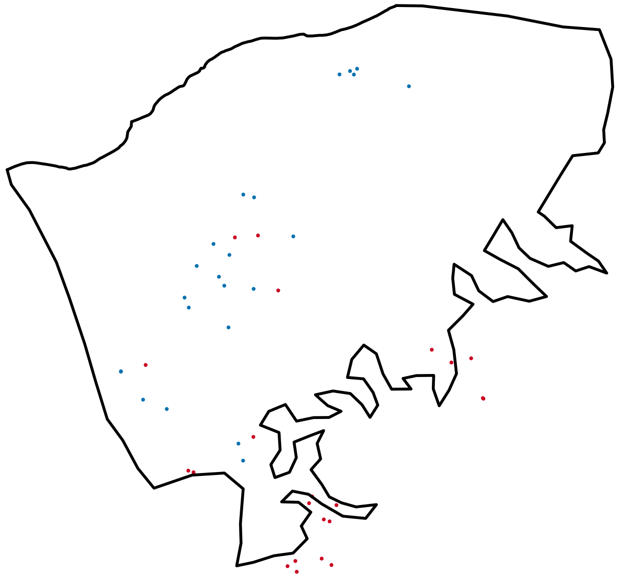

ENVIRONMENT

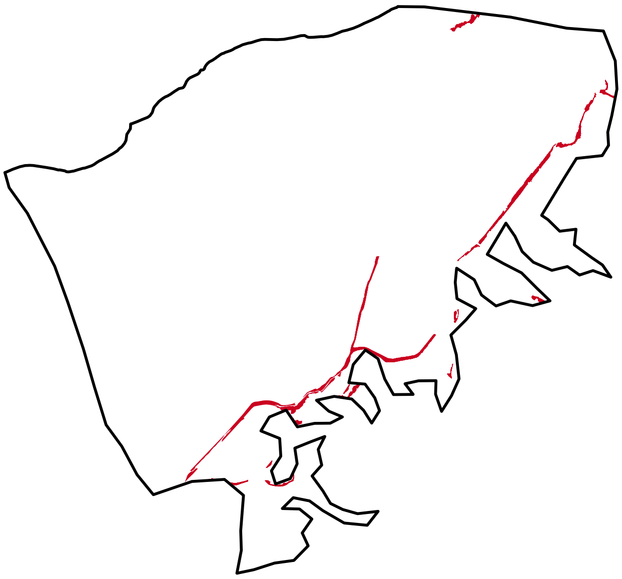

DISADVANTAGED COMMUNITIES

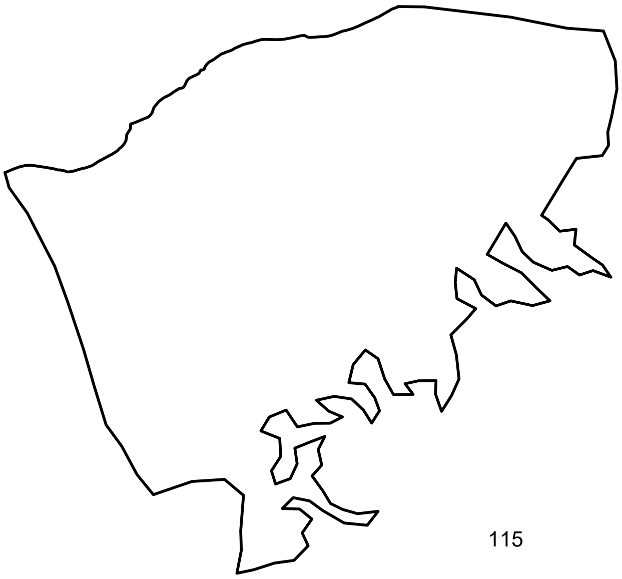

4−008 LAS POSAS VALLEY

AGRICULTURE

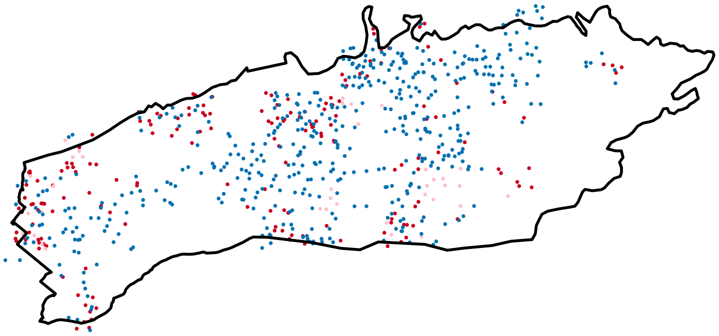

DOMESTIC

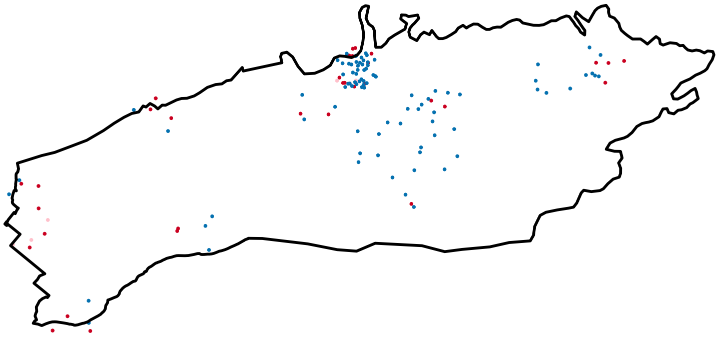

ENVIRONMENT

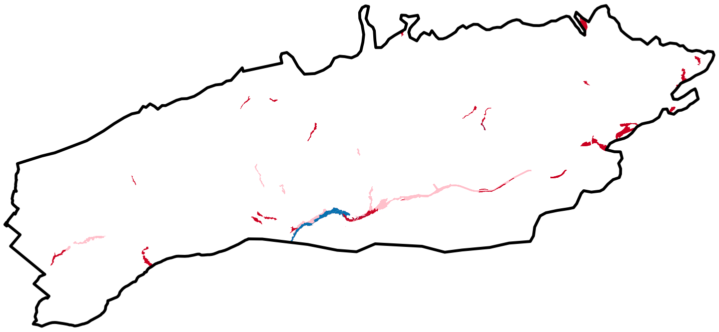

DISADVANTAGED COMMUNITIES

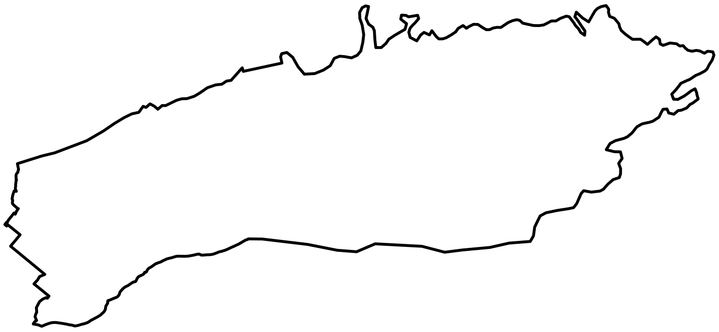

4−011.01 SANTA MONICA

AGRICULTURE

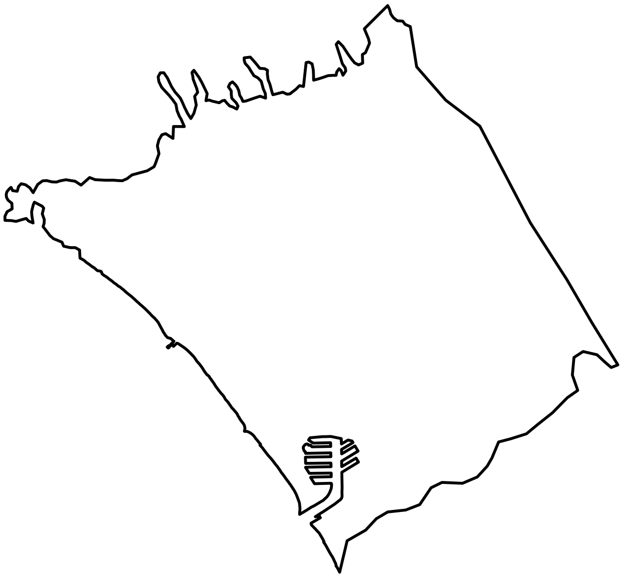

DOMESTIC

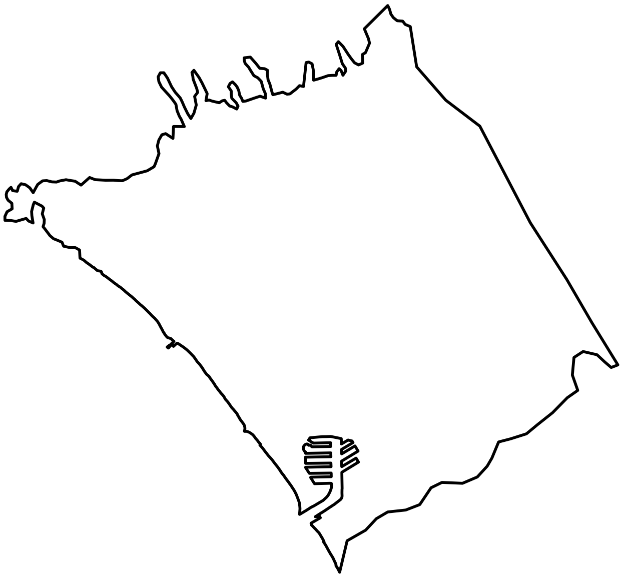

ENVIRONMENT

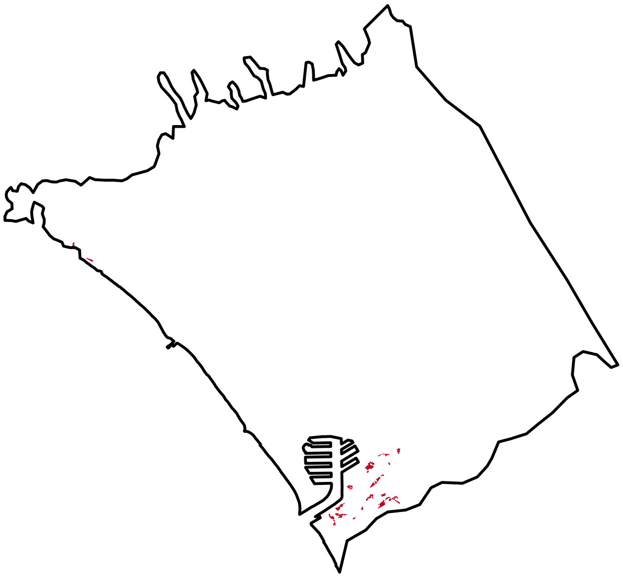

DISADVANTAGED COMMUNITIES

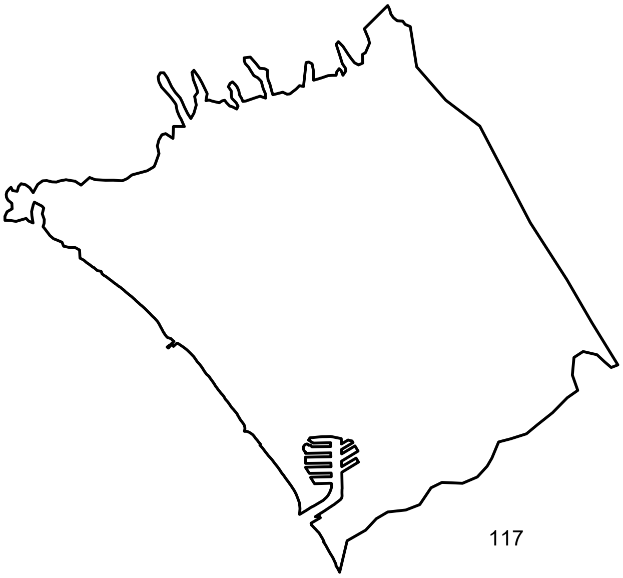

5−004 BIG VALLEY

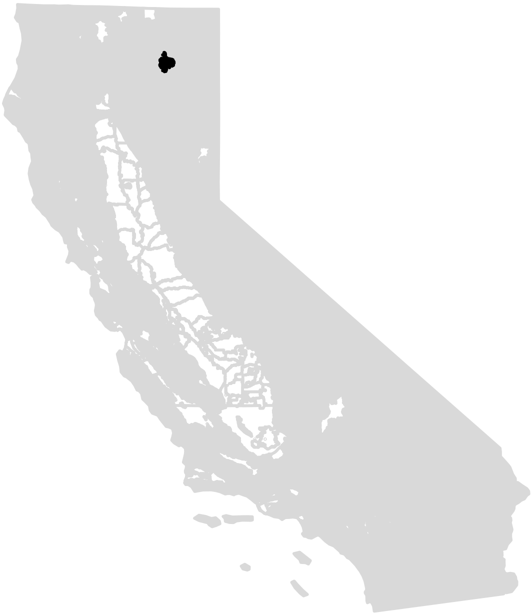

AGRICULTURE

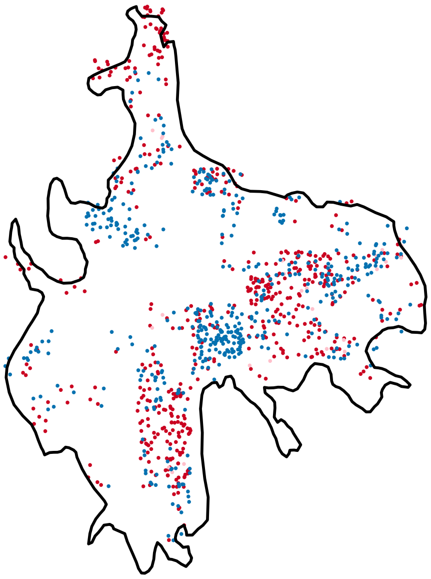

DOMESTIC

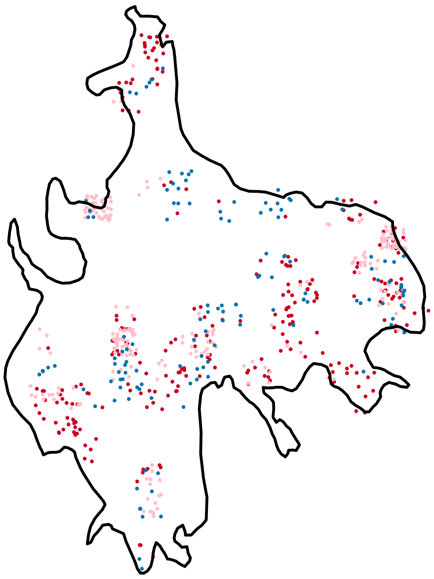

ENVIRONMENT

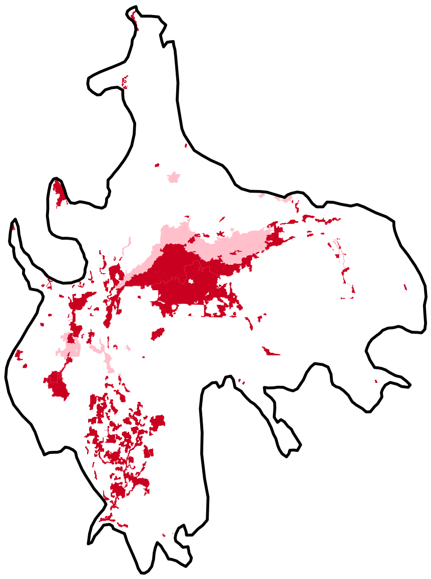

DISADVANTAGED COMMUNITIES

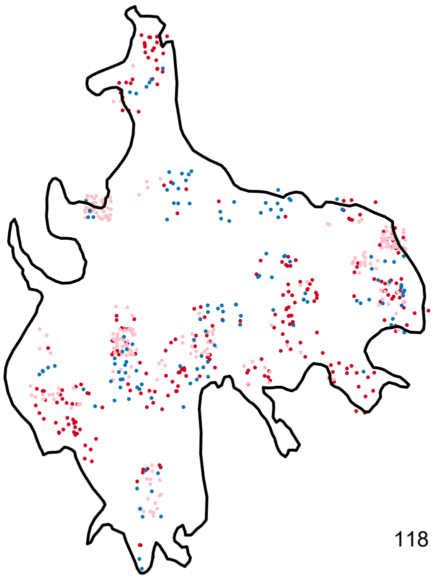

5−006.03 ANDERSON

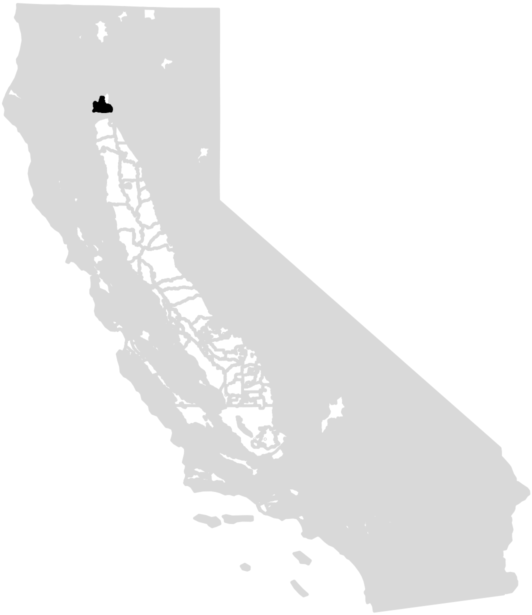

AGRICULTURE

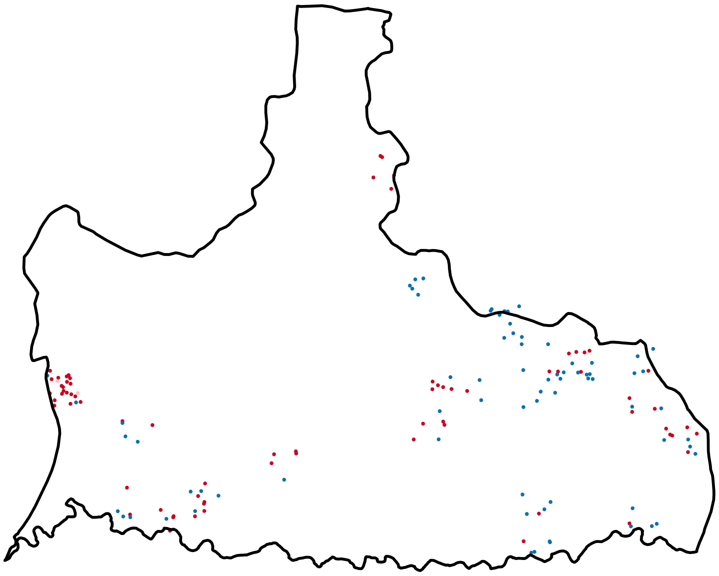

DOMESTIC

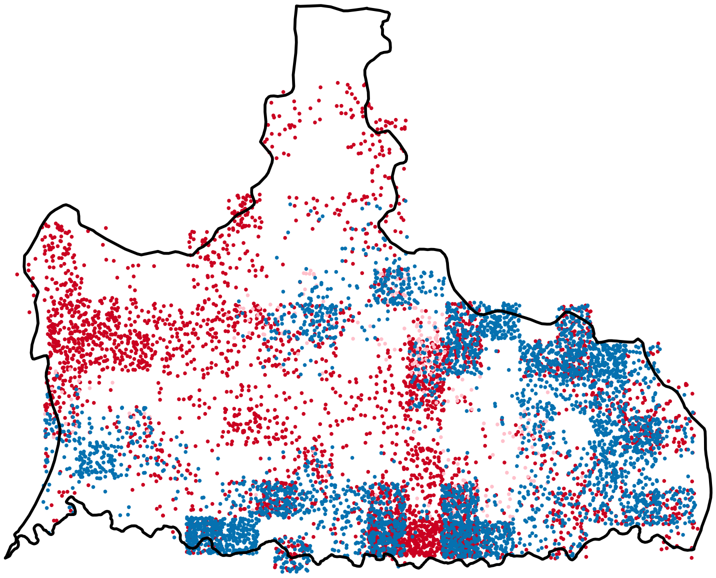

ENVIRONMENT

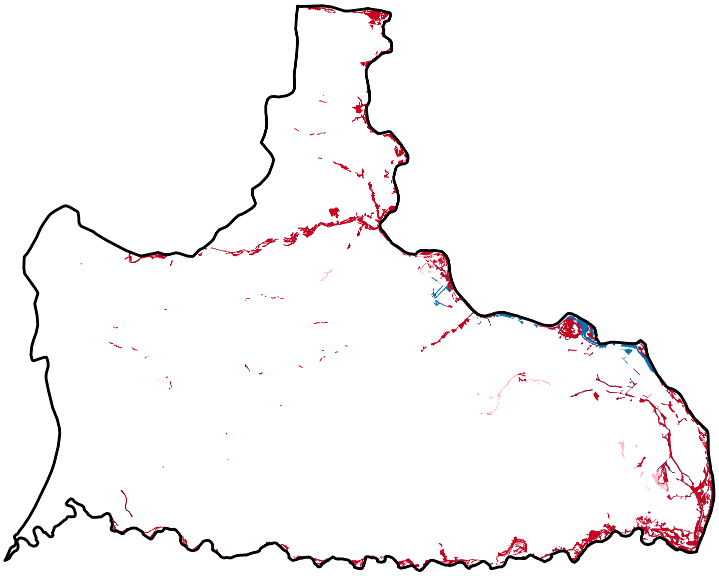

DISADVANTAGED COMMUNITIES

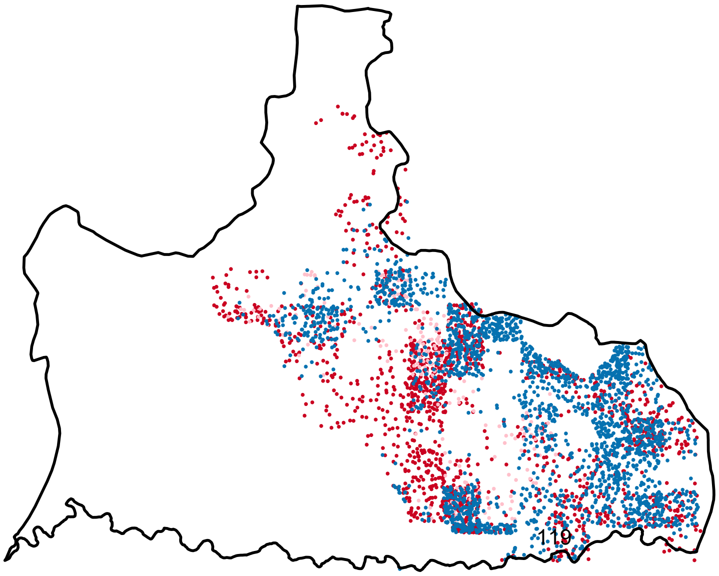

5−006.04 ENTERPRISE

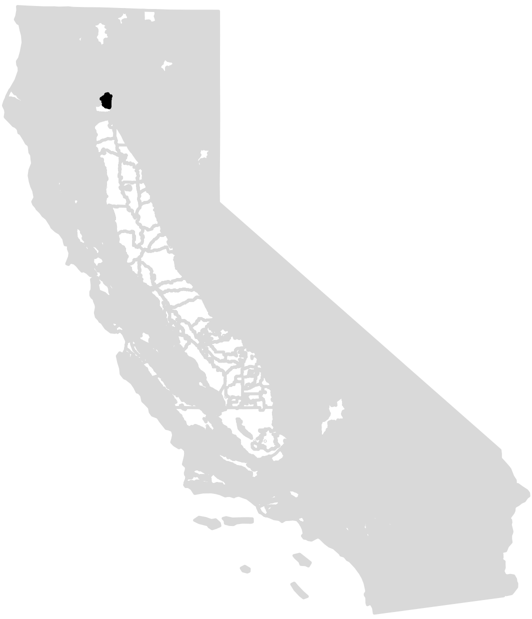

AGRICULTURE

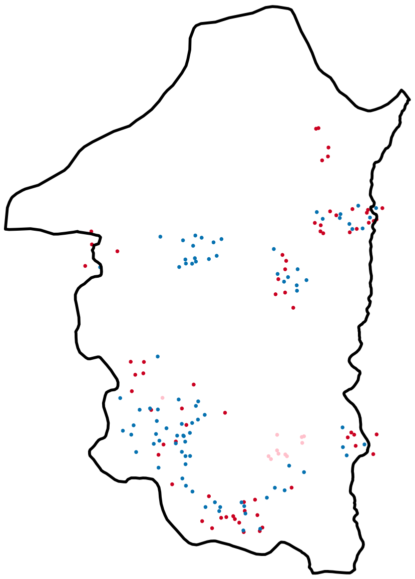

DOMESTIC

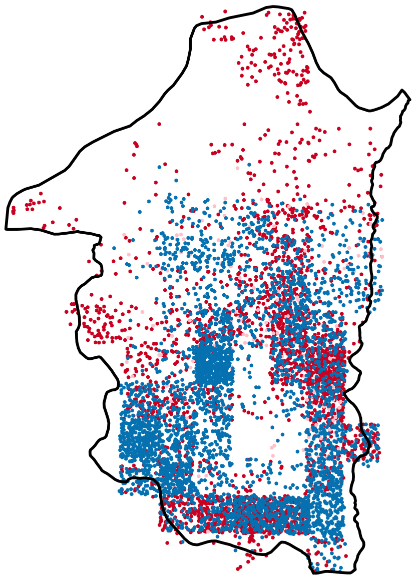

ENVIRONMENT

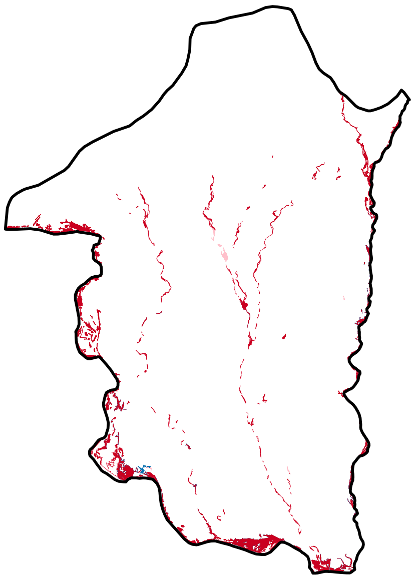

DISADVANTAGED COMMUNITIES

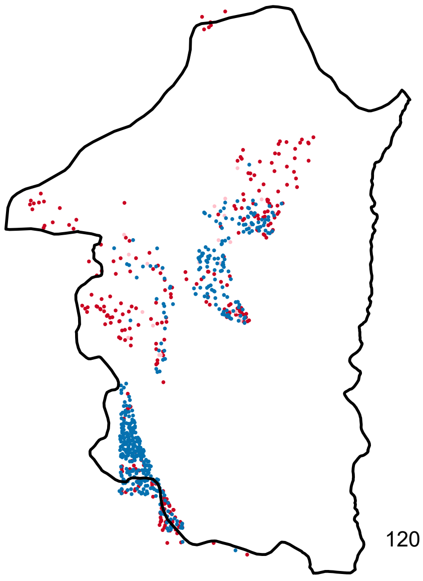

5−012.01 SIERRA VALLEY

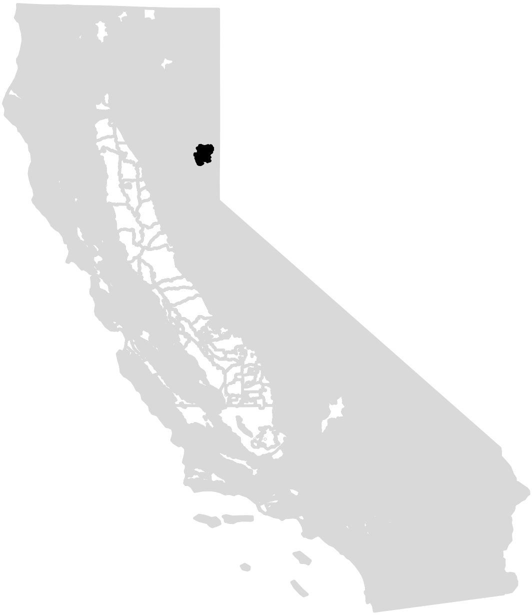

AGRICULTURE

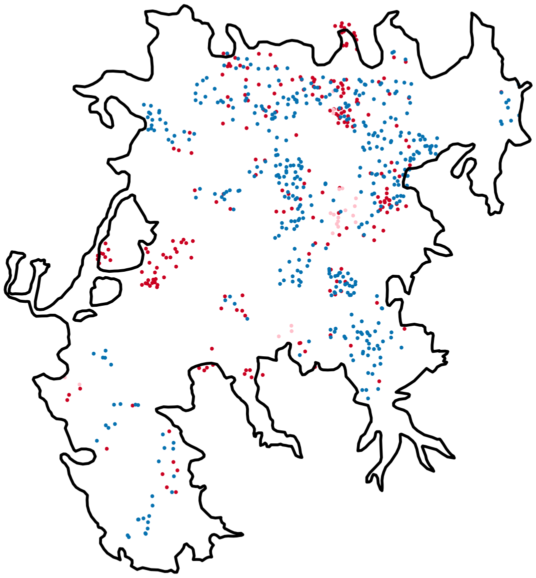

DOMESTIC

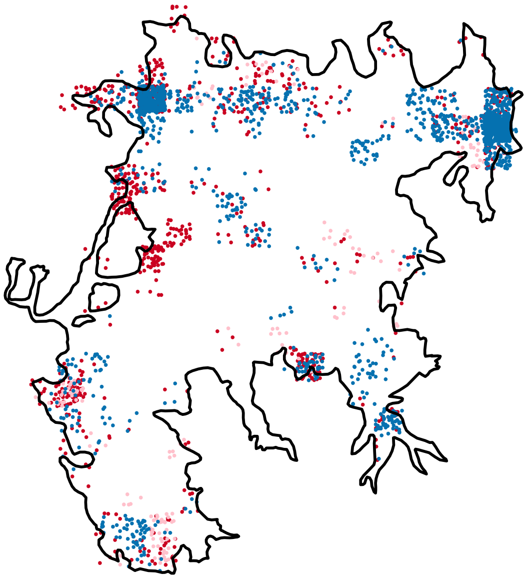

ENVIRONMENT

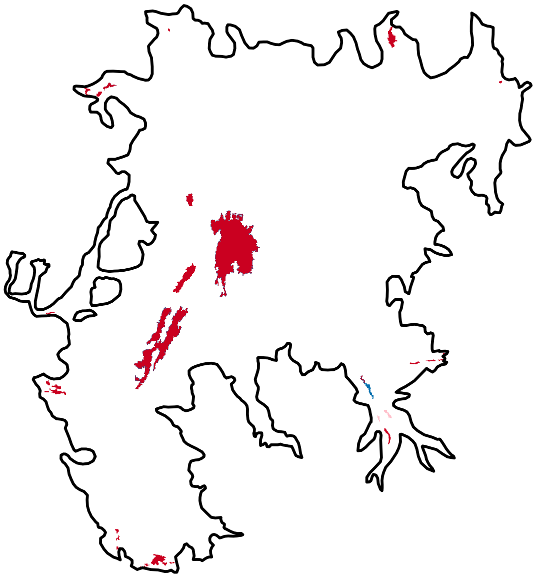

DISADVANTAGED COMMUNITIES

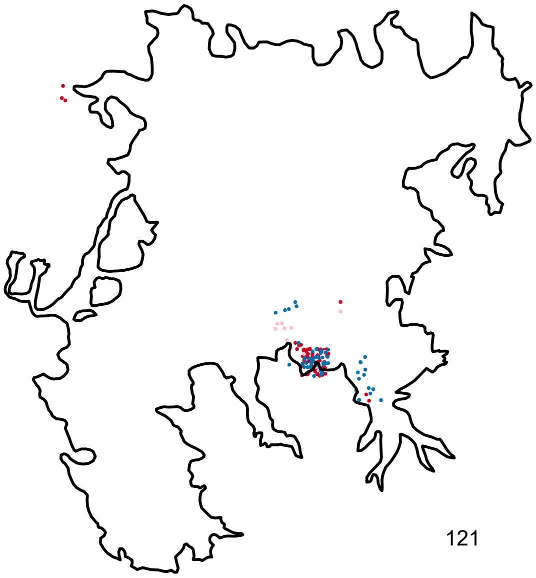

5−015 BIG VALLEY

AGRICULTURE

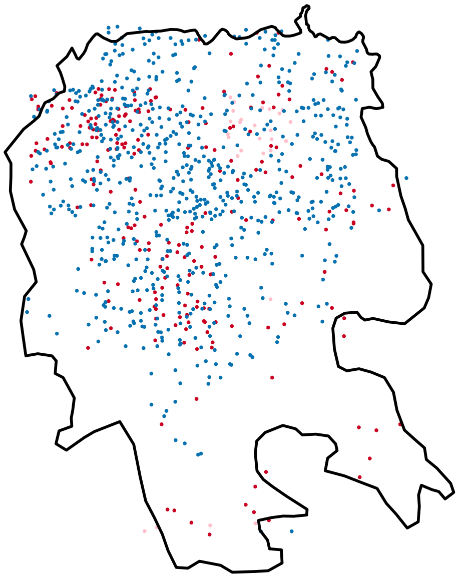

DOMESTIC

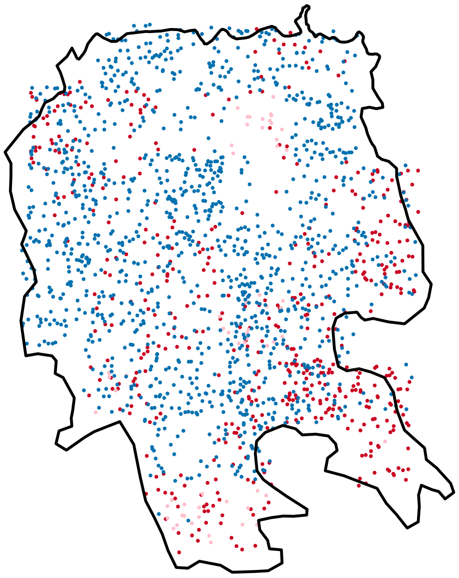

ENVIRONMENT

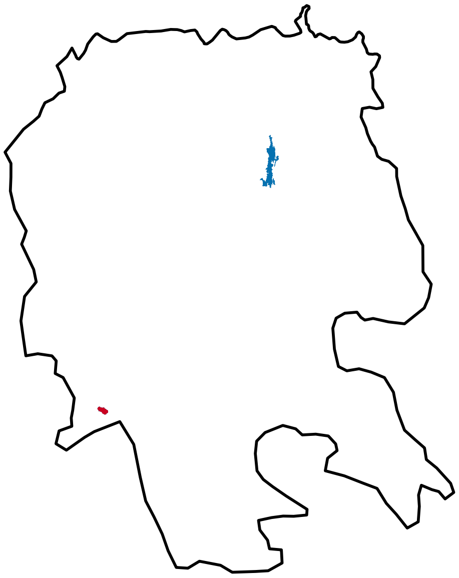

DISADVANTAGED COMMUNITIES

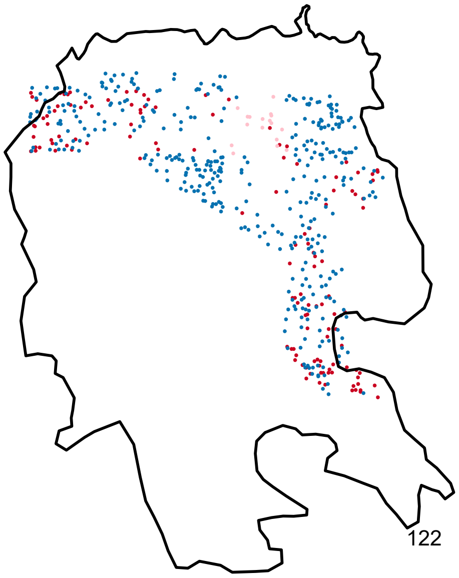

5−021.50 RED BLUFF

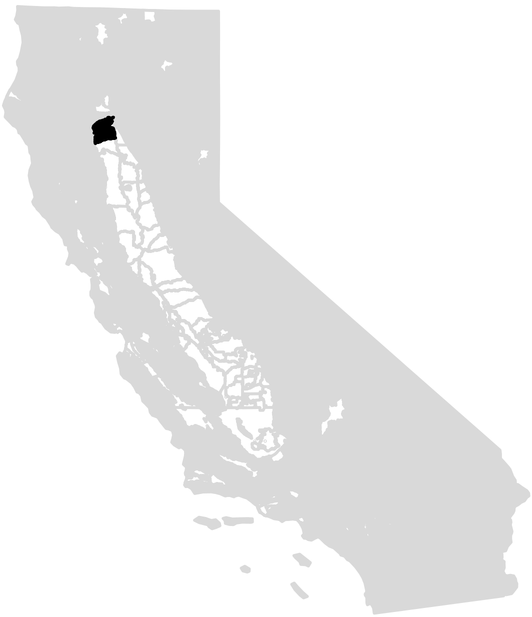

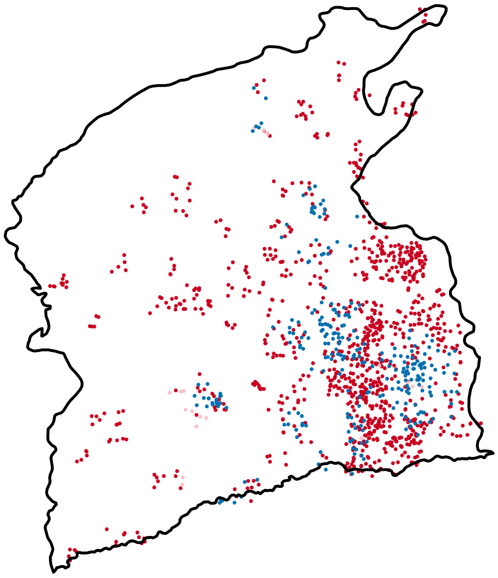

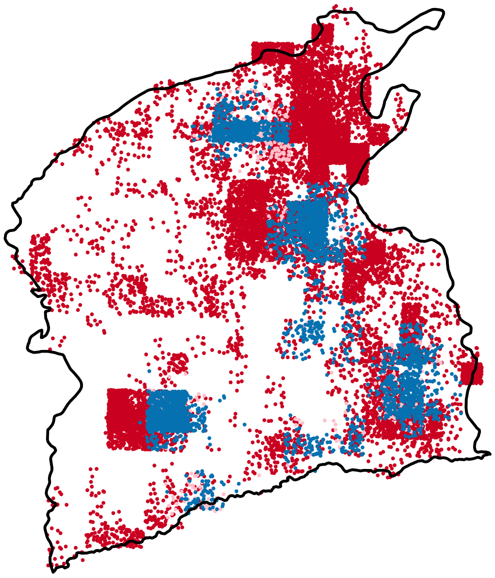

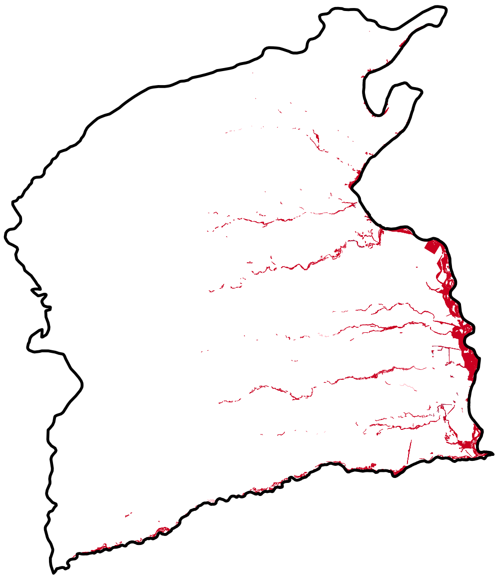

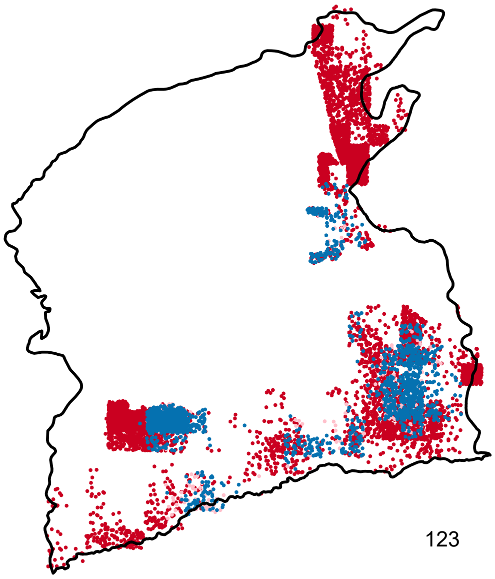

5−021.51 CORNING

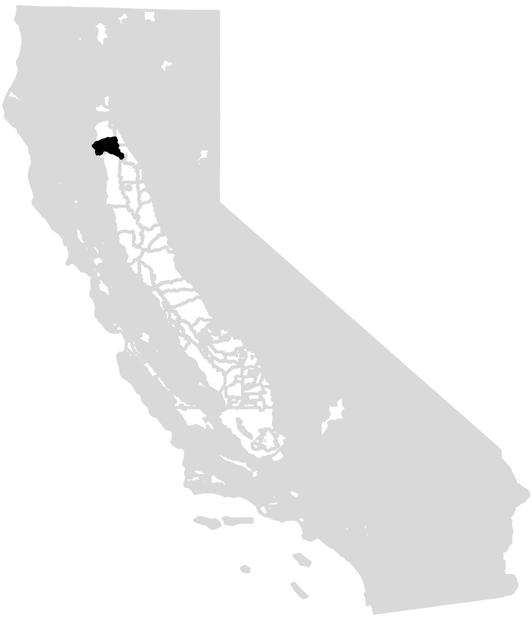

AGRICULTURE

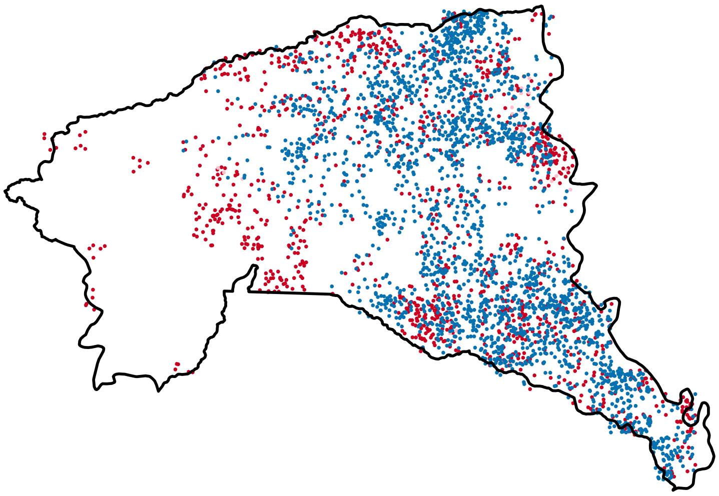

DOMESTIC

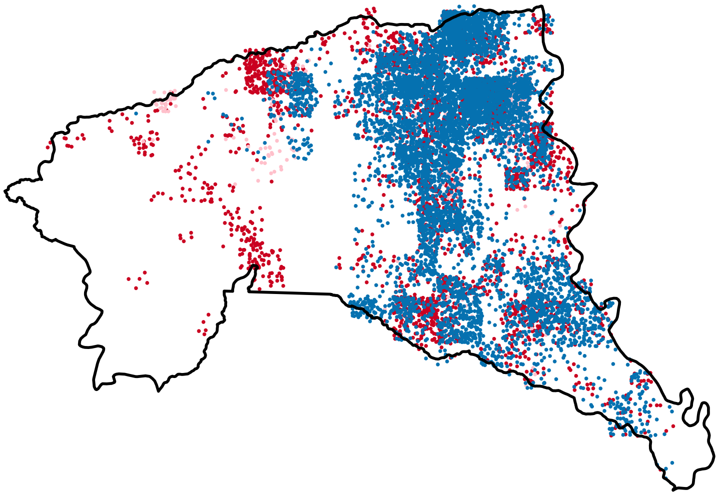

ENVIRONMENT

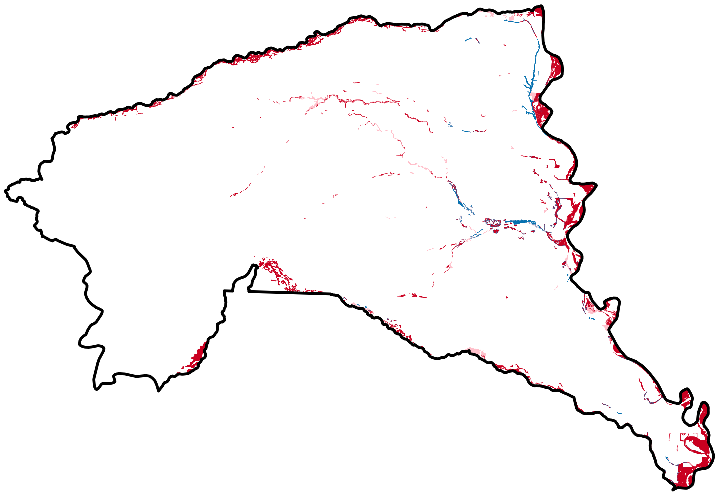

DISADVANTAGED COMMUNITIES

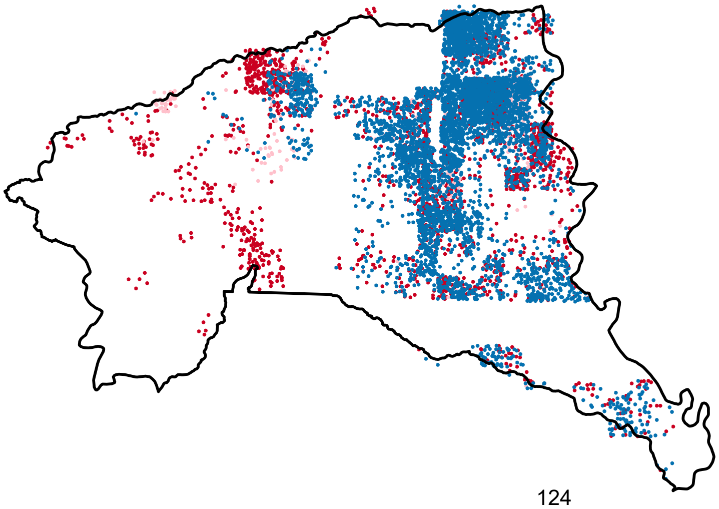

5−021.52 COLUSA

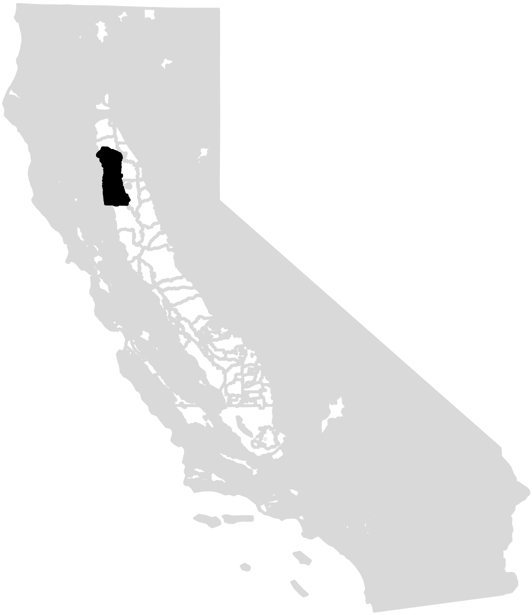

AGRICULTURE

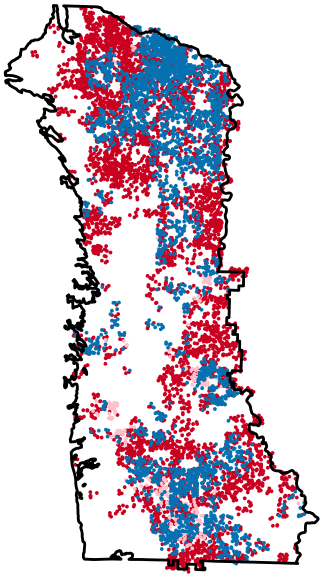

DOMESTIC

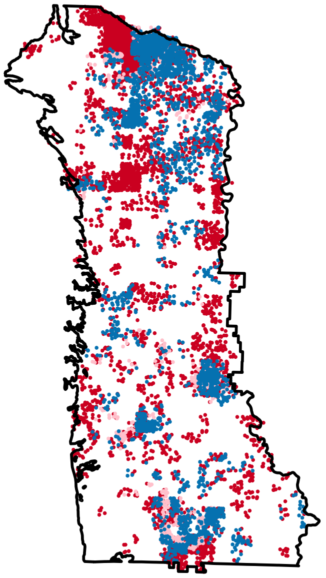

ENVIRONMENT

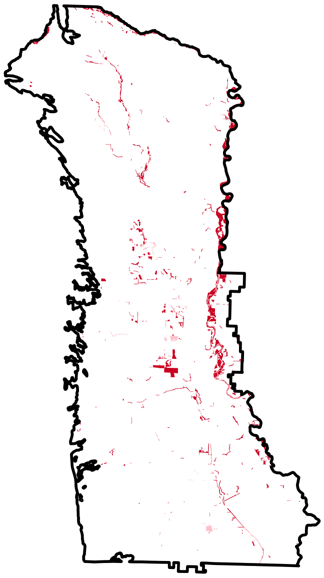

DISADVANTAGED COMMUNITIES

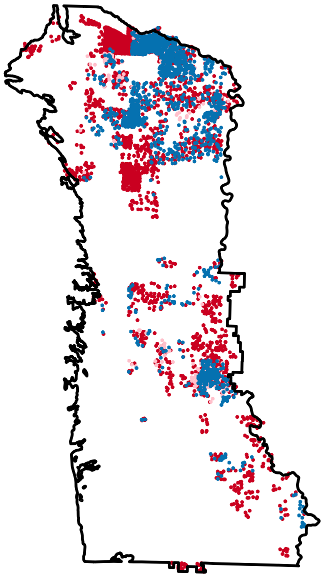

5−021.54 ANTELOPE

AGRICULTURE

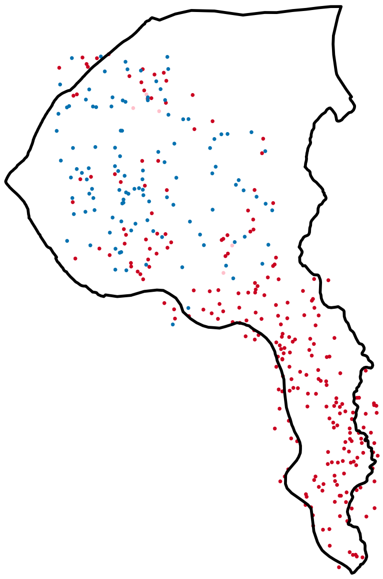

DOMESTIC

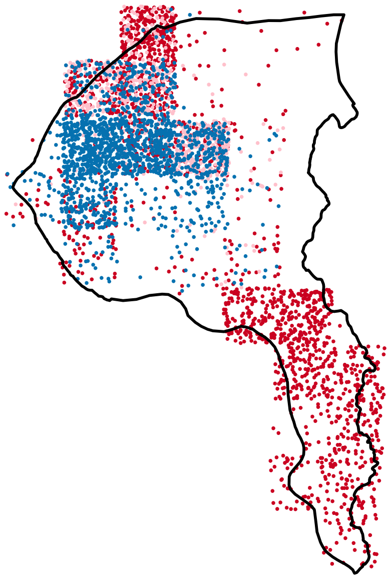

ENVIRONMENT

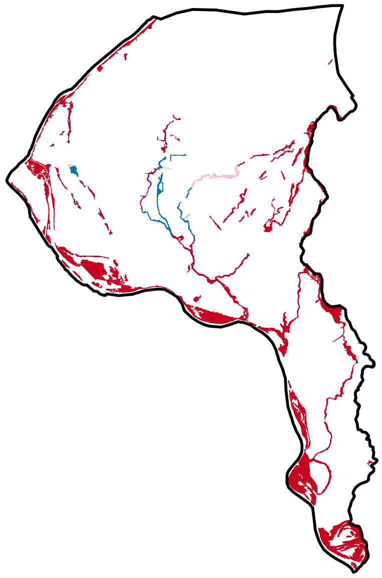

DISADVANTAGED COMMUNITIES

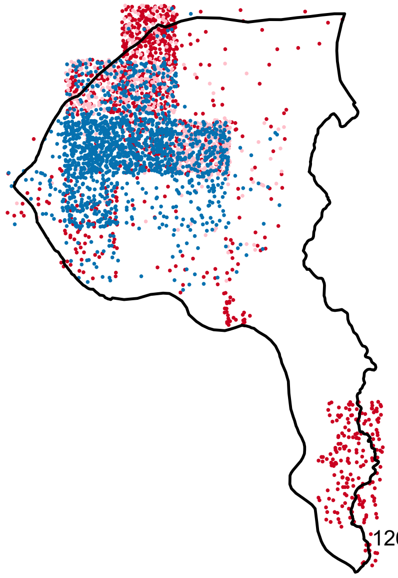

5−021.56 LOS MOLINOS

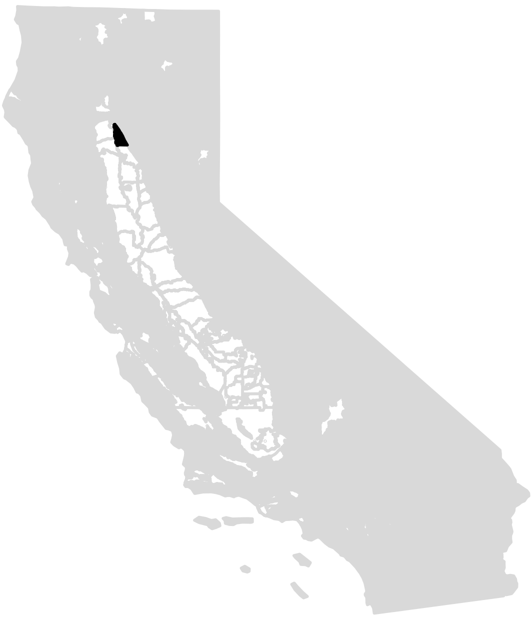

AGRICULTURE

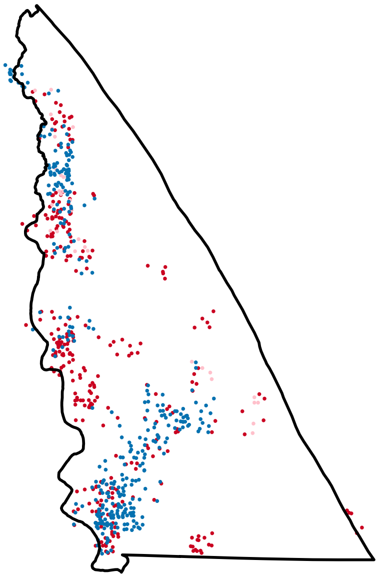

DOMESTIC

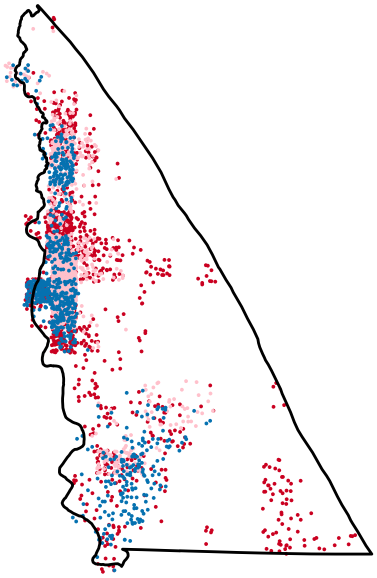

ENVIRONMENT

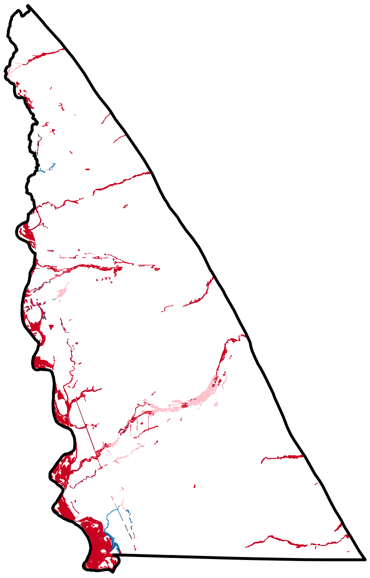

DISADVANTAGED COMMUNITIES

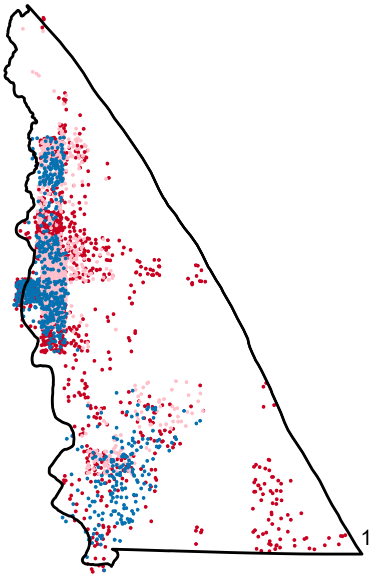

5−021.57 VINA

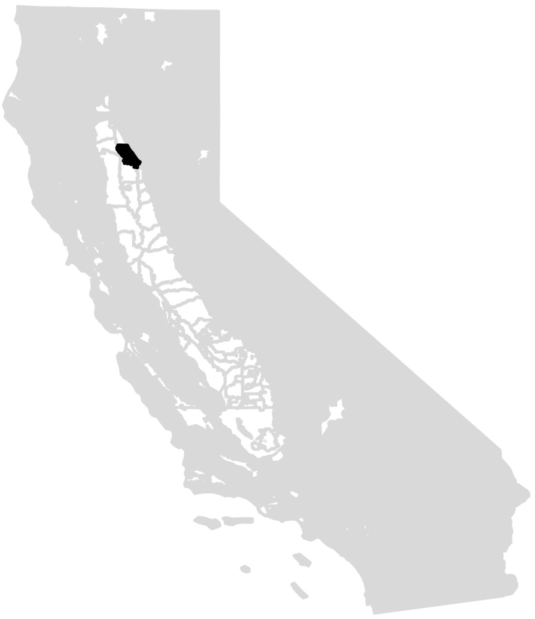

AGRICULTURE

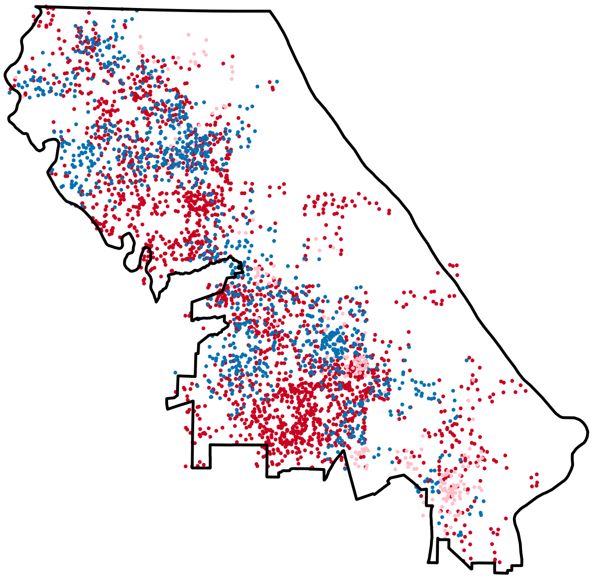

DOMESTIC

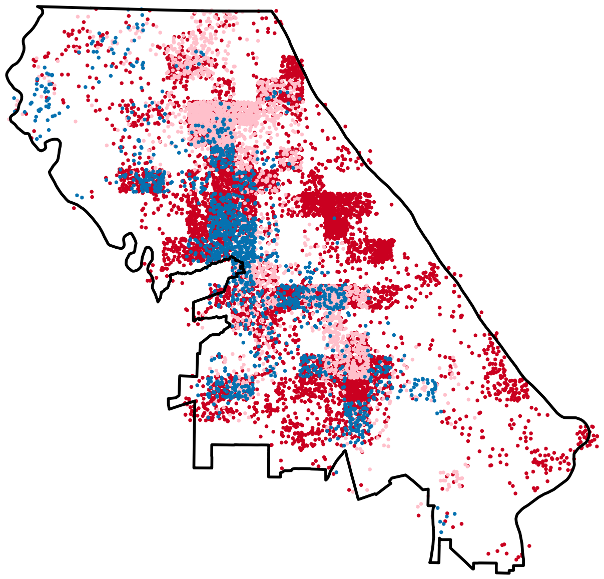

ENVIRONMENT

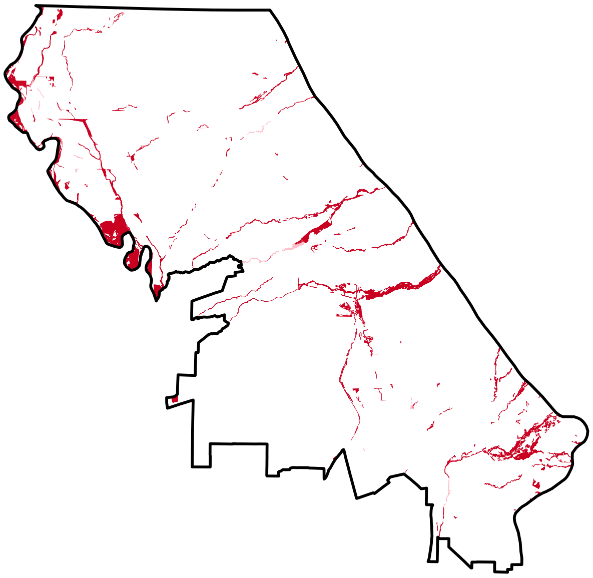

DISADVANTAGED COMMUNITIES

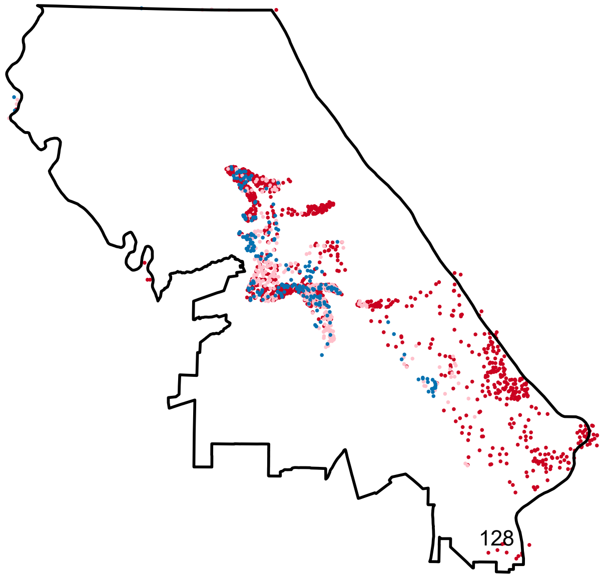

5−021.60 NORTH YUBA

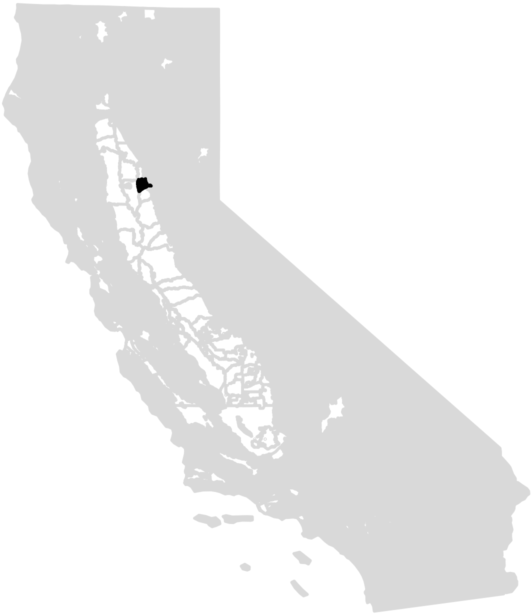

AGRICULTURE

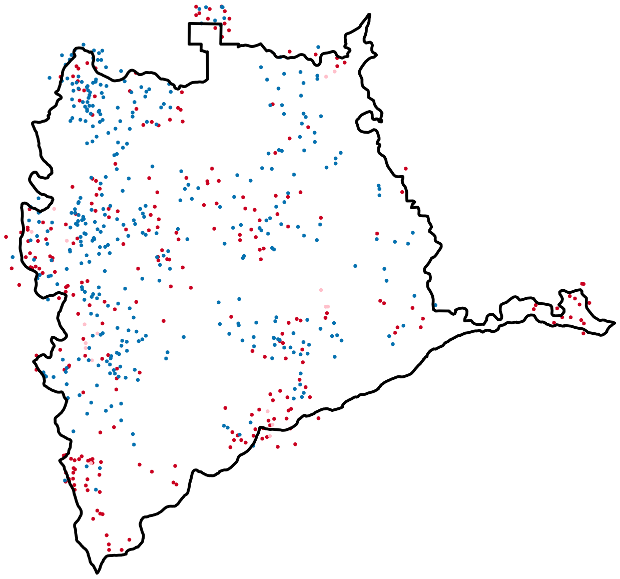

DOMESTIC

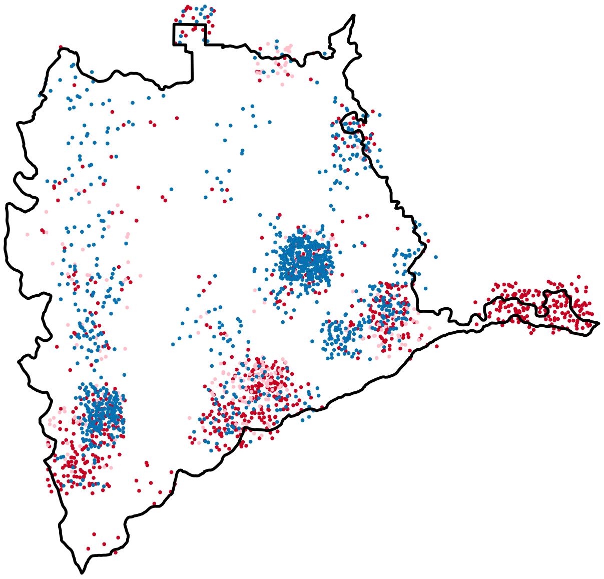

ENVIRONMENT

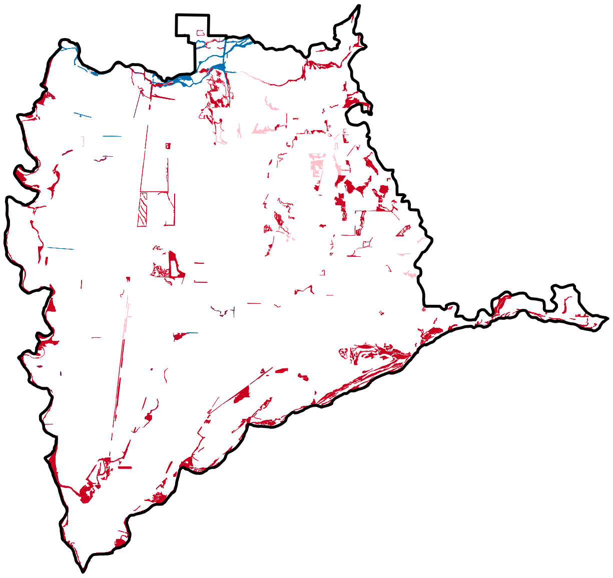

DISADVANTAGED COMMUNITIES

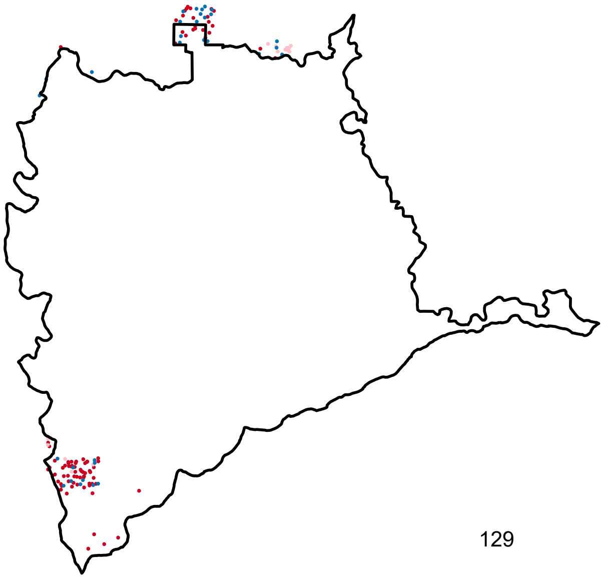

5−021.61 SOUTH YUBA

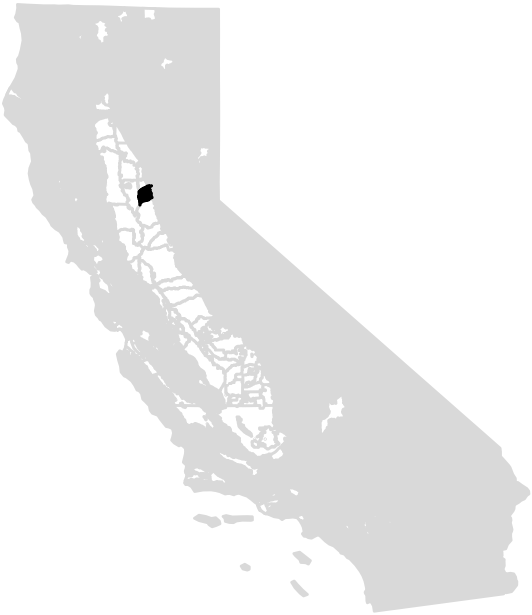

AGRICULTURE

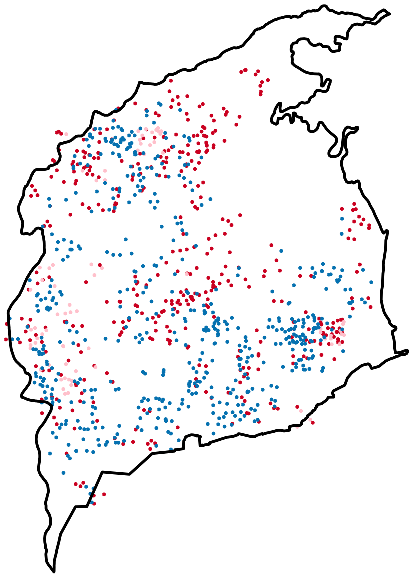

DOMESTIC

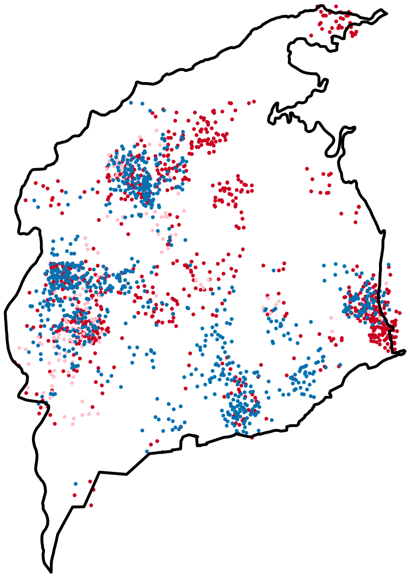

ENVIRONMENT

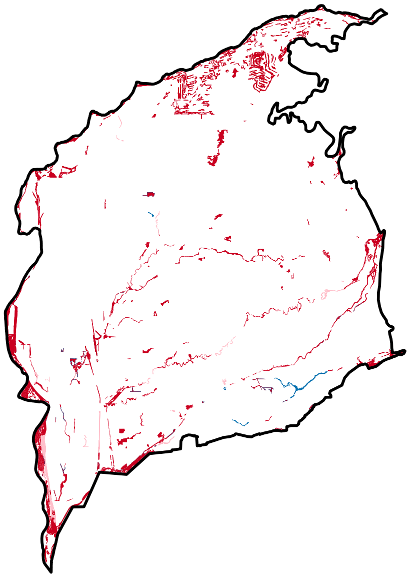

DISADVANTAGED COMMUNITIES

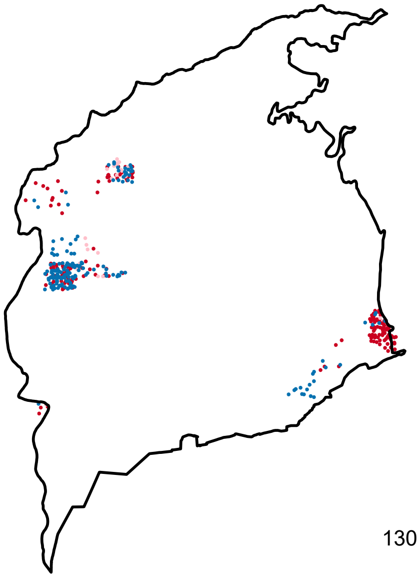

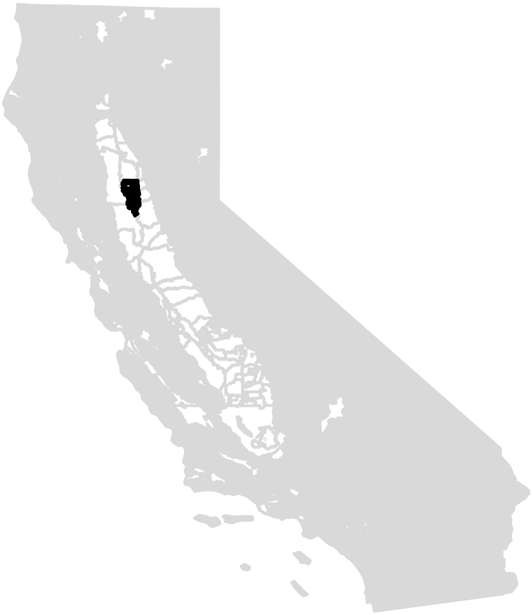

5−021.62 SUTTER

AGRICULTURE

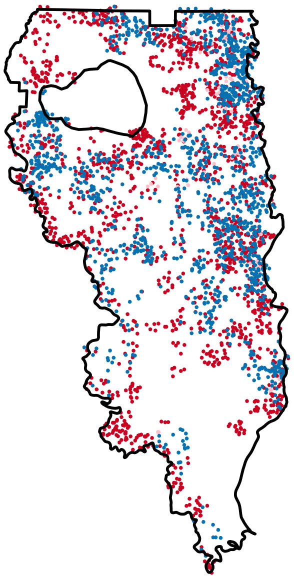

DOMESTIC

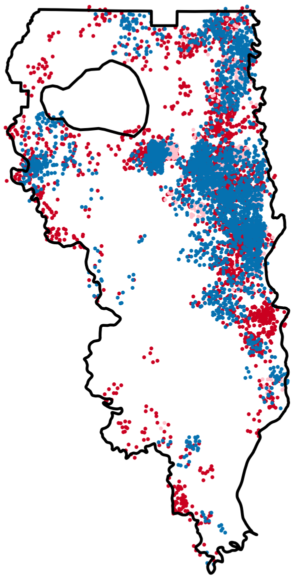

ENVIRONMENT

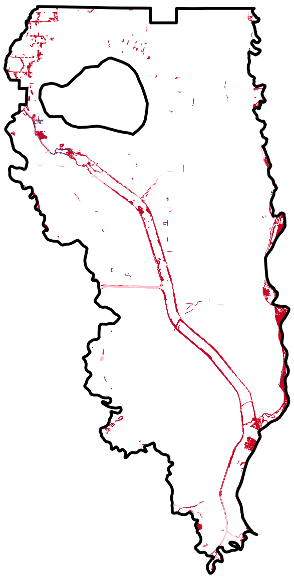

DISADVANTAGED COMMUNITIES

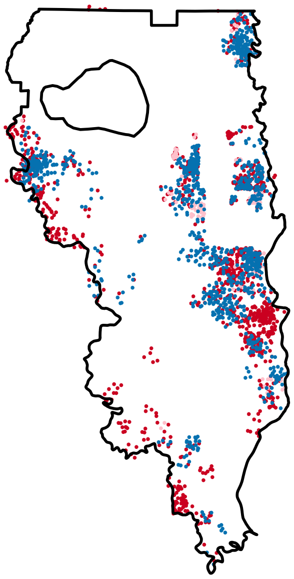

5−021.64 NORTH AMERICAN

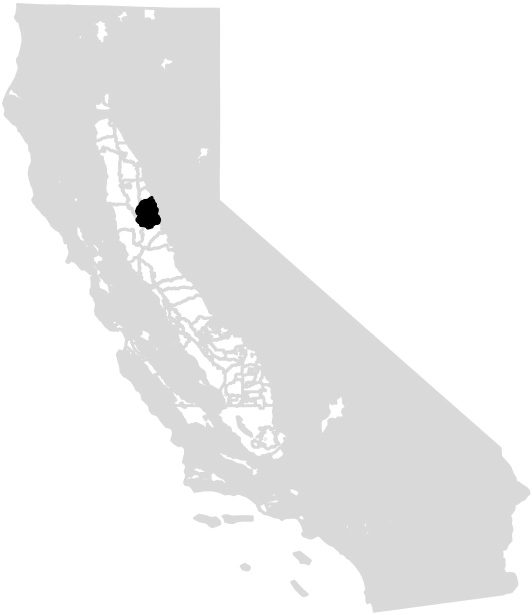

AGRICULTURE

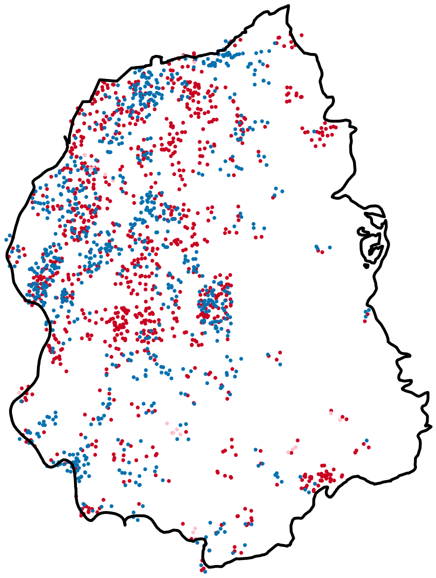

DOMESTIC

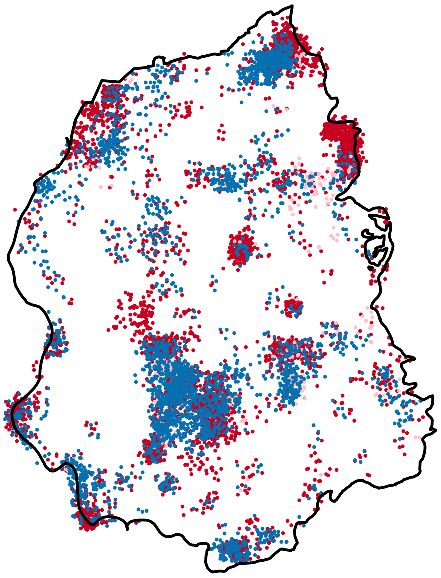

ENVIRONMENT

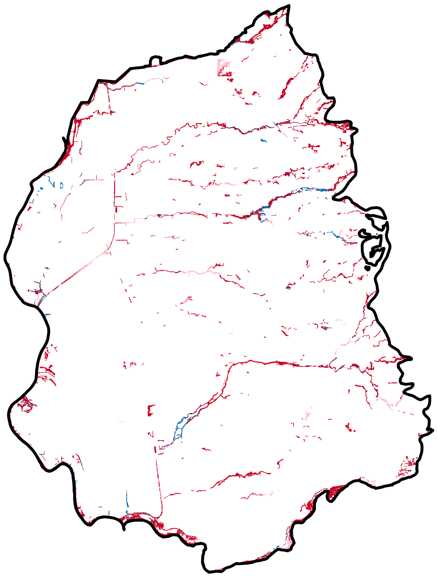

DISADVANTAGED COMMUNITIES

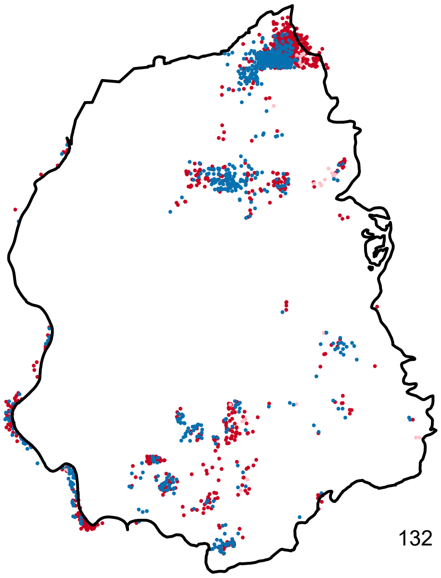

5−021.65 SOUTH AMERICAN

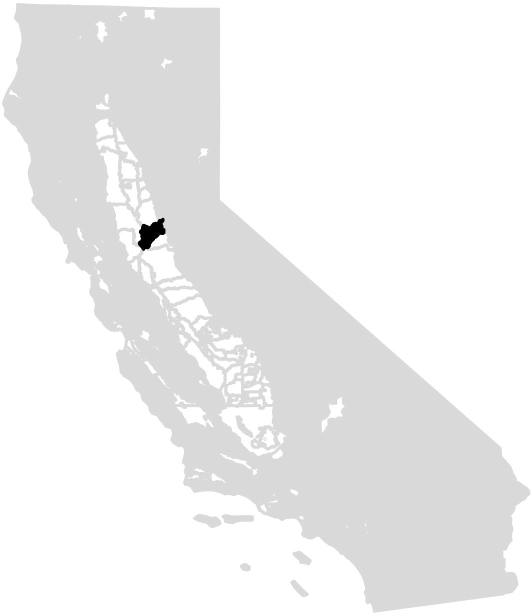

AGRICULTURE

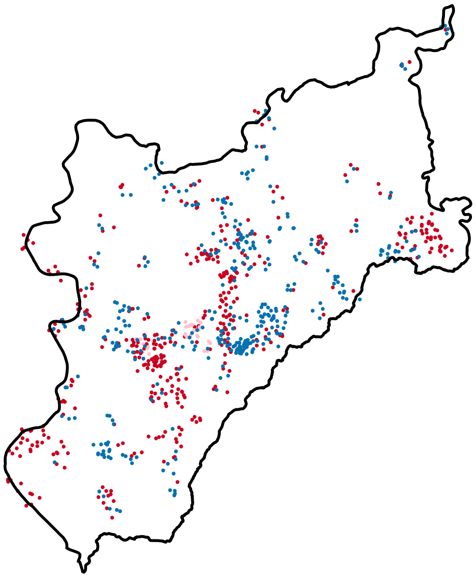

DOMESTIC

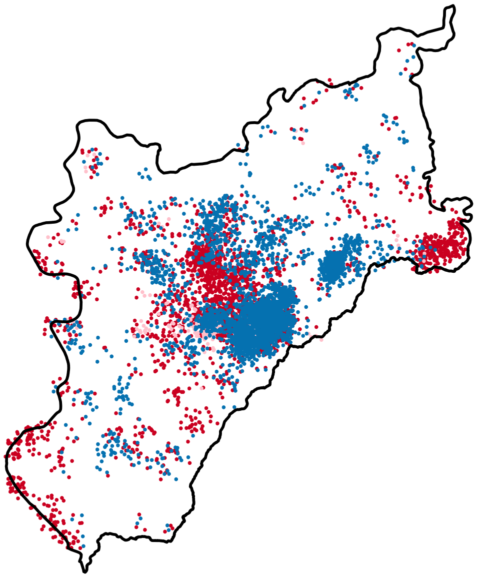

ENVIRONMENT

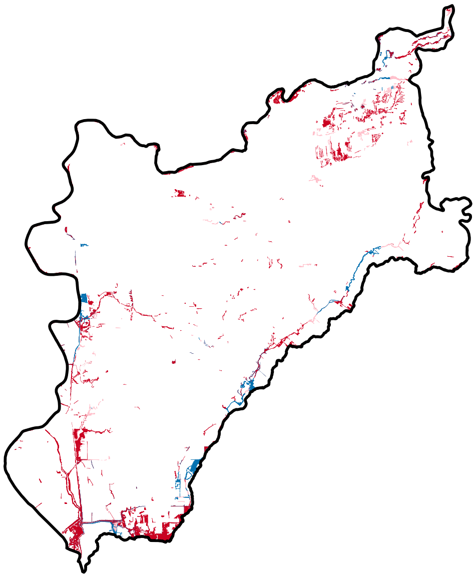

DISADVANTAGED COMMUNITIES

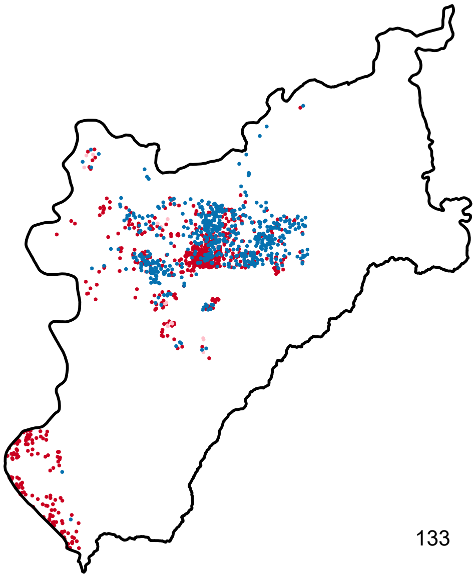

5−021.66 SOLANO

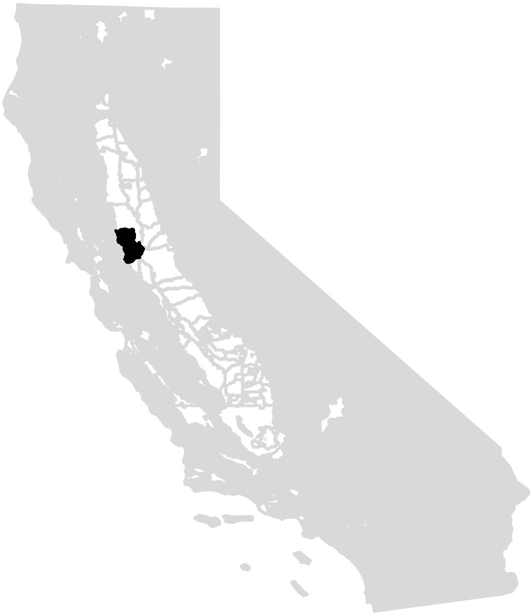

AGRICULTURE

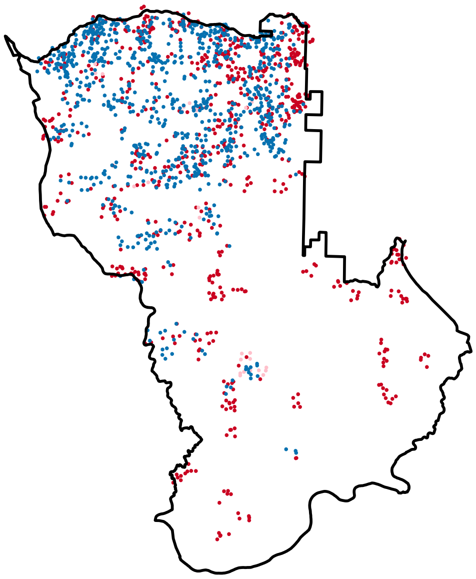

DOMESTIC

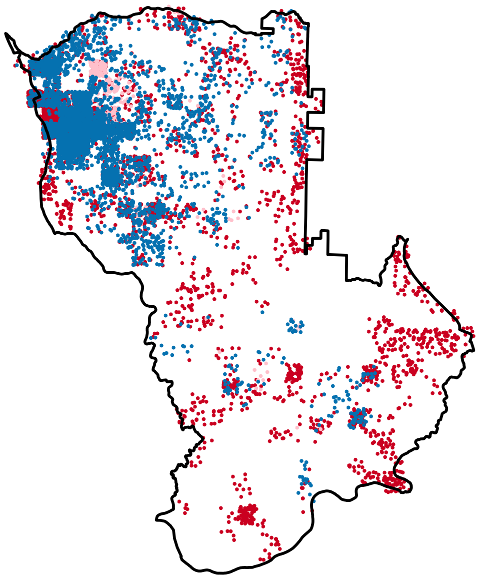

ENVIRONMENT

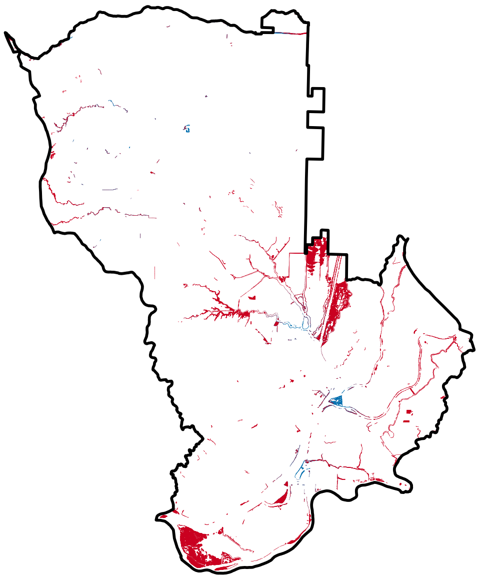

DISADVANTAGED COMMUNITIES

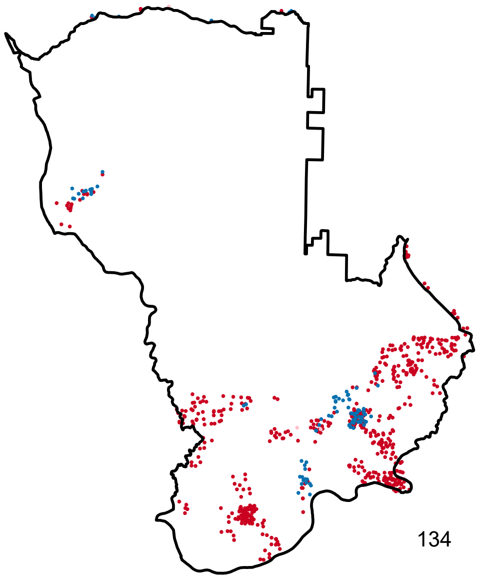

5−021.67 YOLO

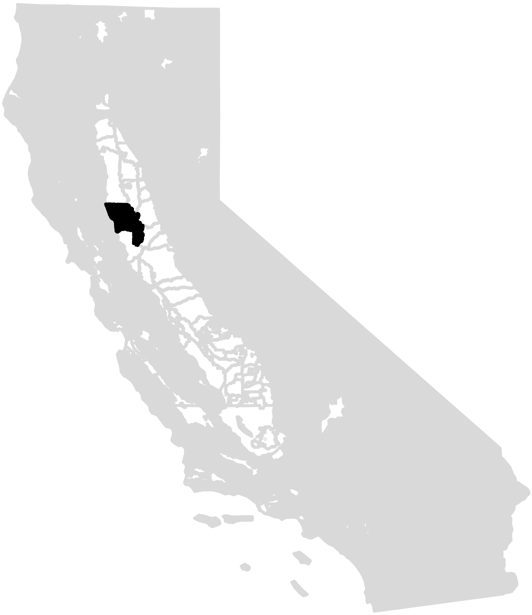

AGRICULTURE

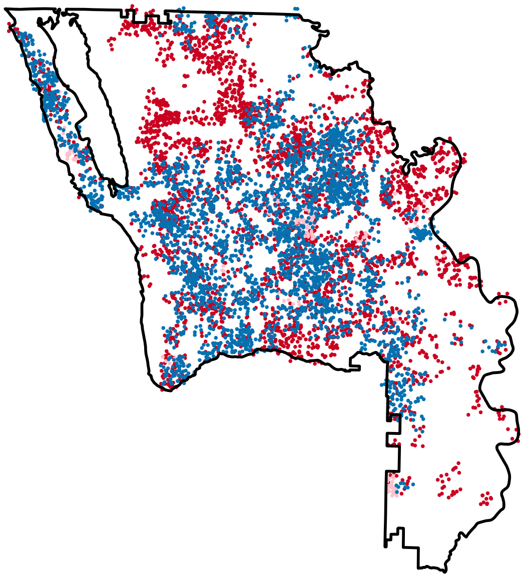

DOMESTIC

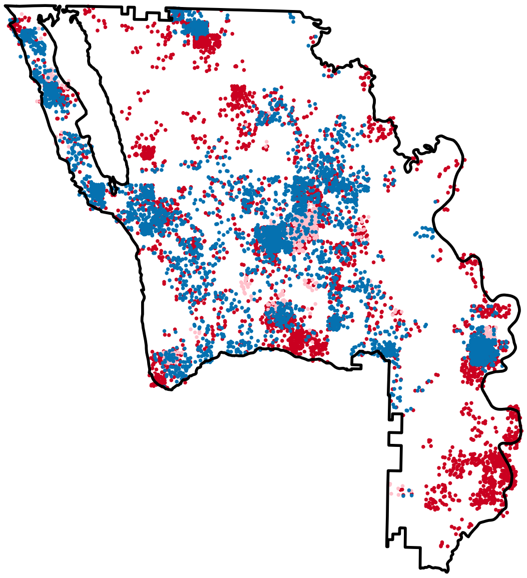

ENVIRONMENT

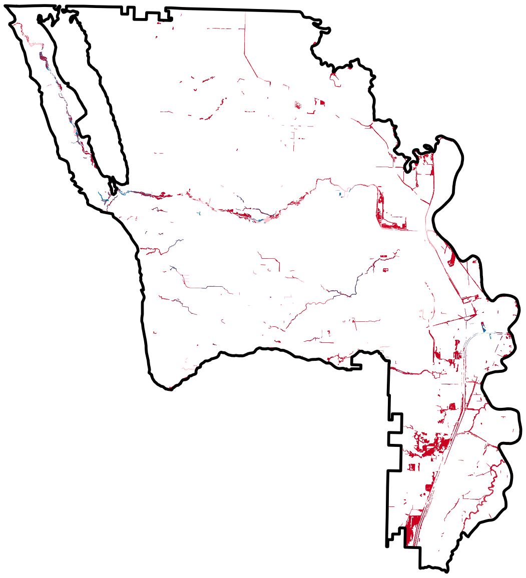

DISADVANTAGED COMMUNITIES

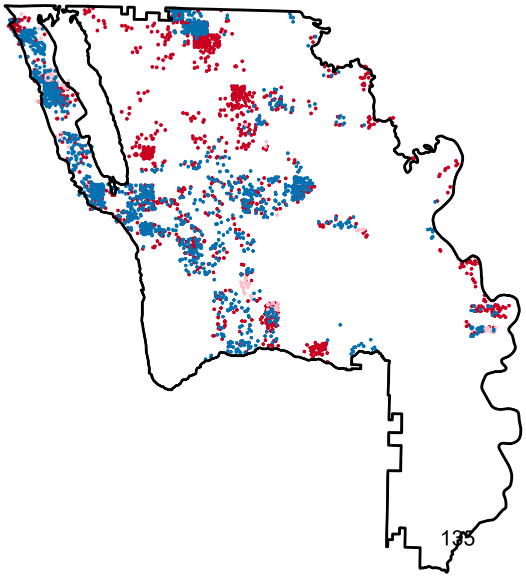

5−021.69 WYANDOTTE CREEK

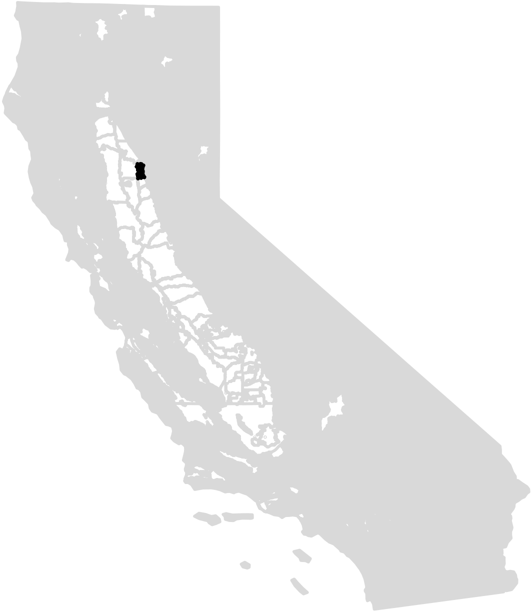

AGRICULTURE

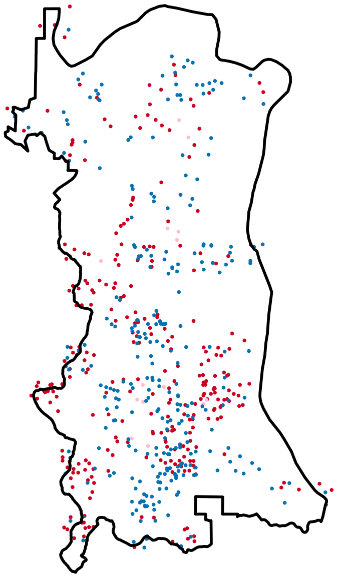

DOMESTIC

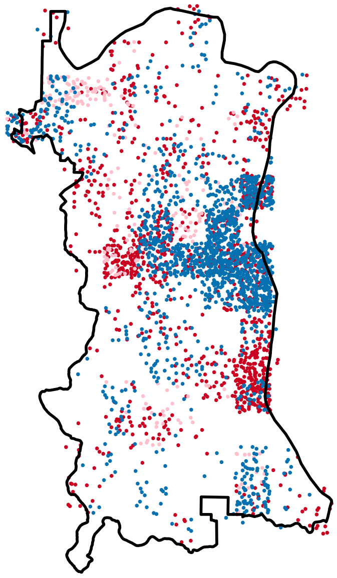

ENVIRONMENT

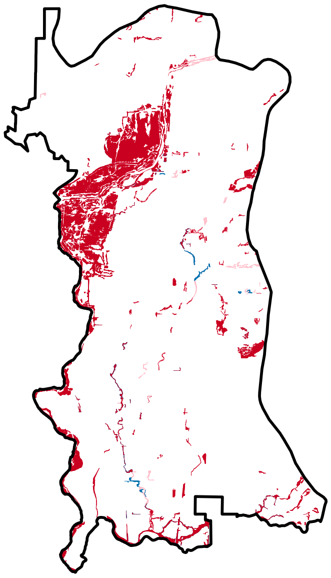

DISADVANTAGED COMMUNITIES

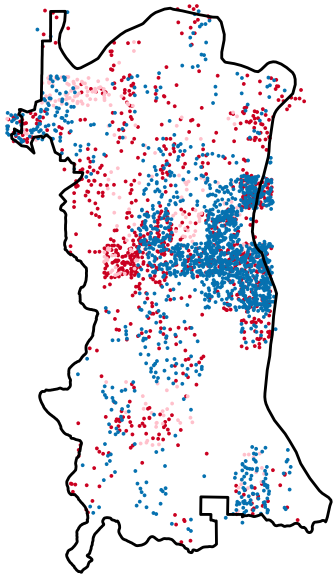

5−021.70 BUTTE

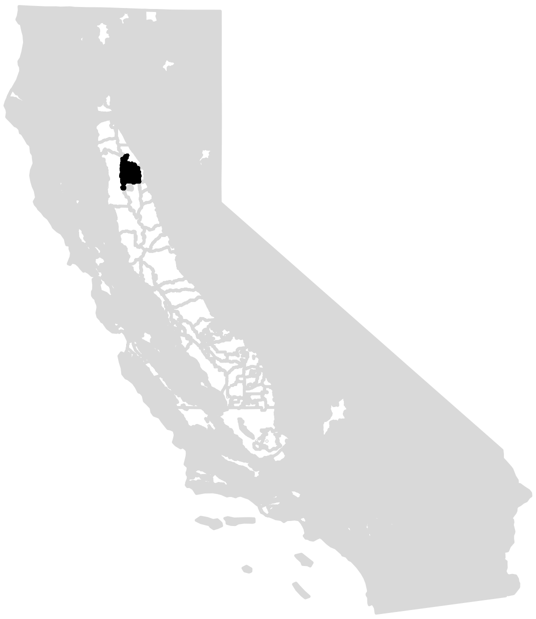

AGRICULTURE

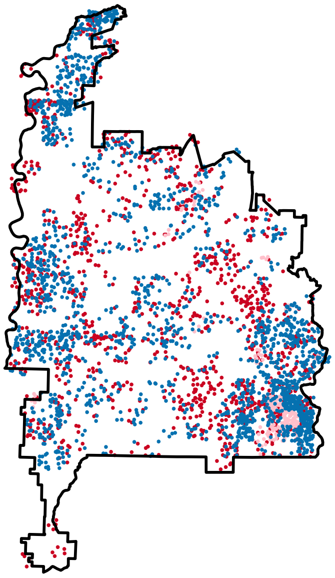

DOMESTIC

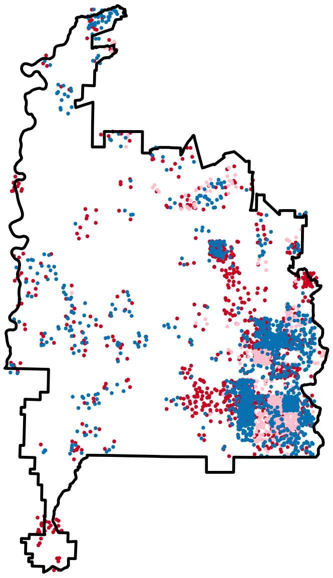

ENVIRONMENT

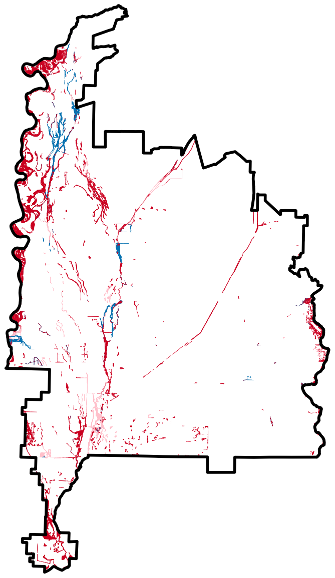

DISADVANTAGED COMMUNITIES

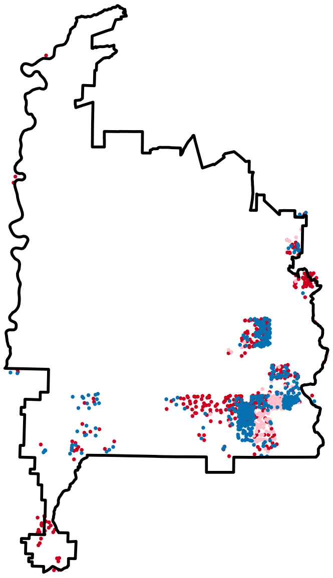

5−022.01 EASTERN SAN JOAQUIN

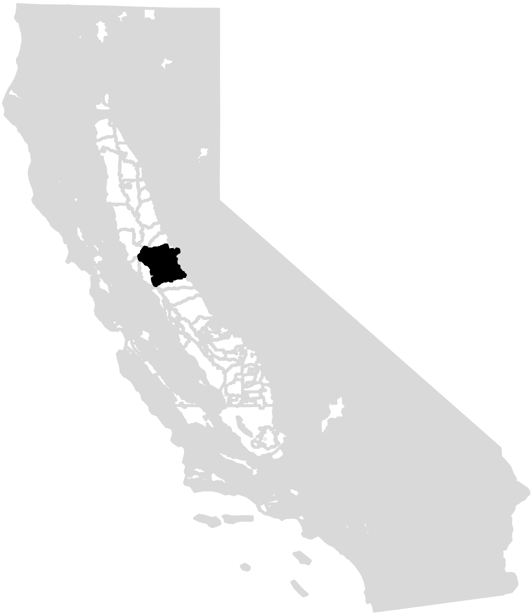

AGRICULTURE

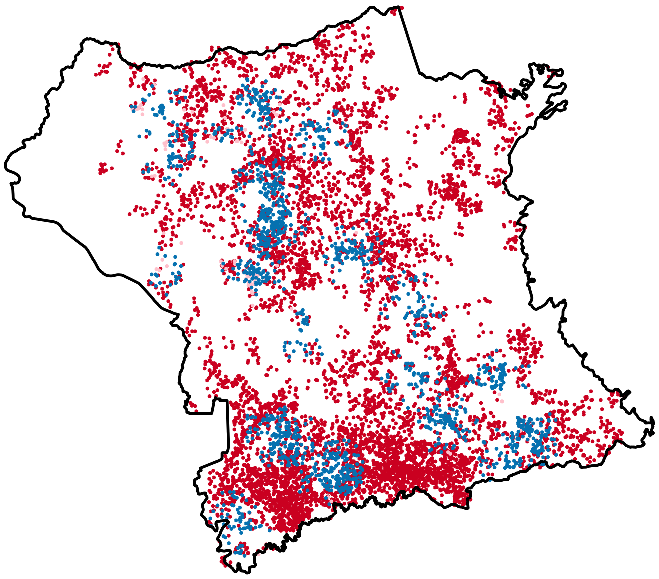

DOMESTIC

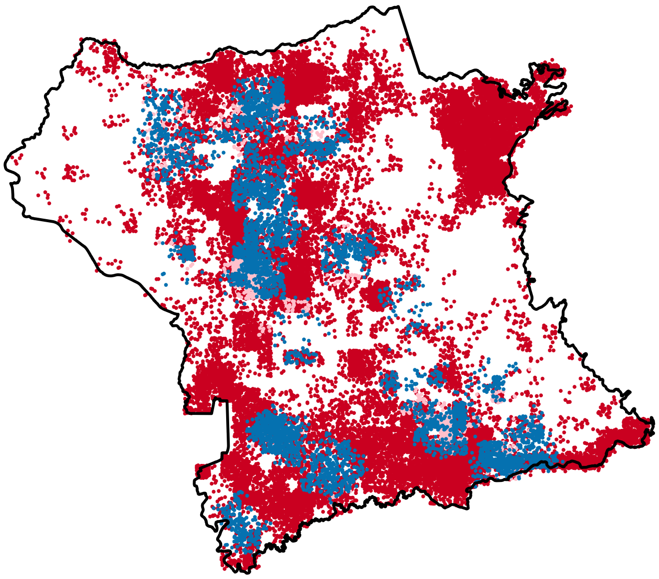

ENVIRONMENT

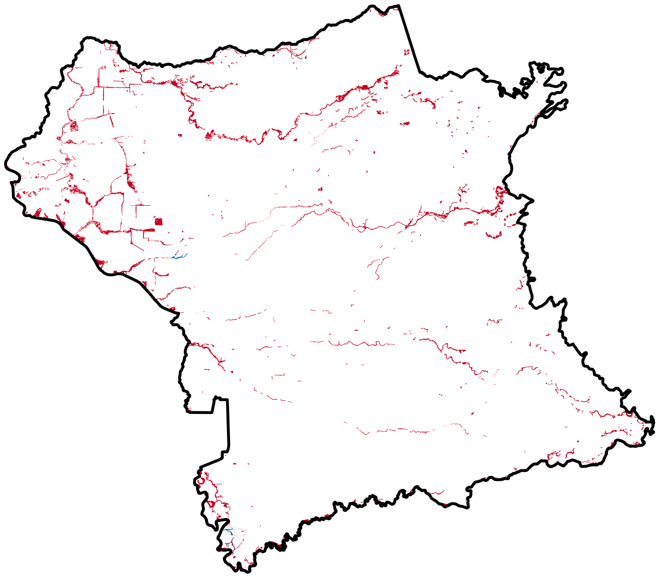

DISADVANTAGED COMMUNITIES

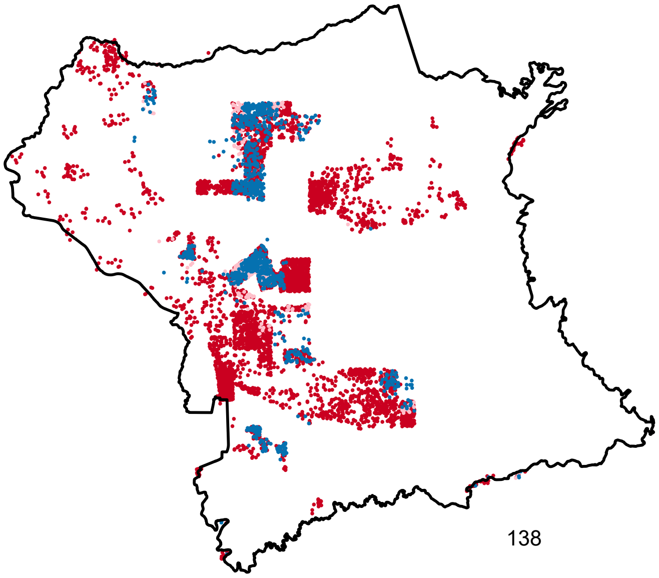

5−022.02 MODESTO

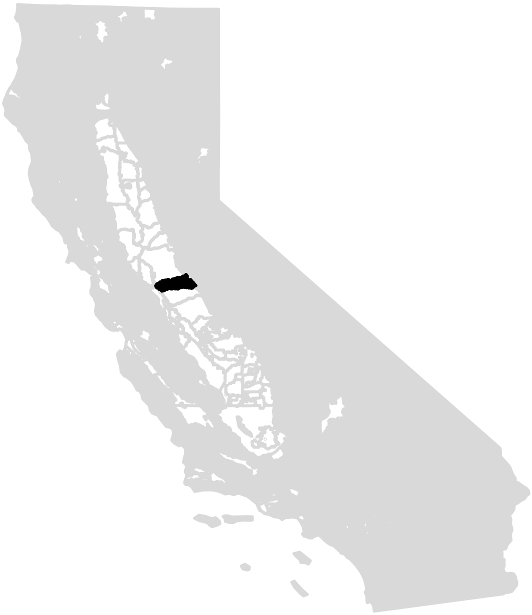

AGRICULTURE

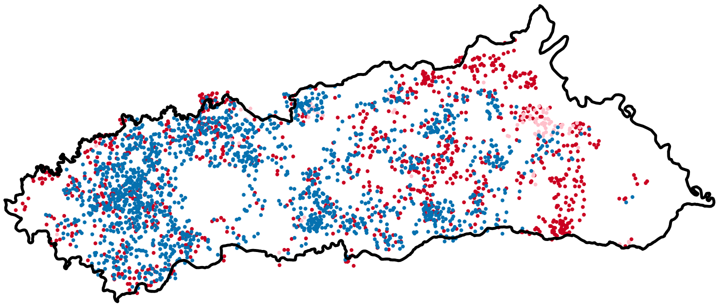

DOMESTIC

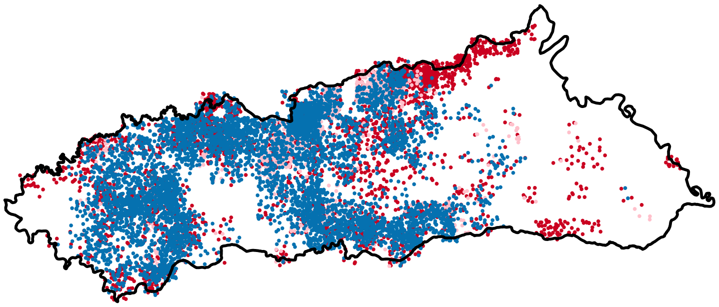

ENVIRONMENT

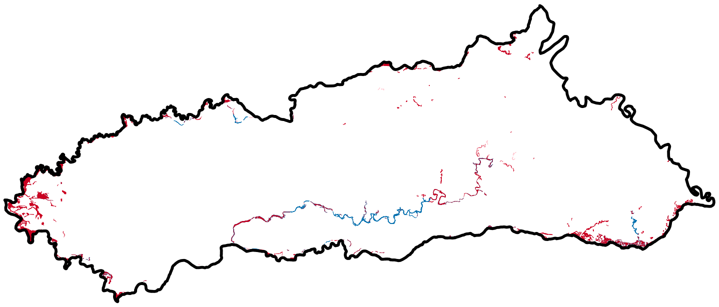

DISADVANTAGED COMMUNITIES

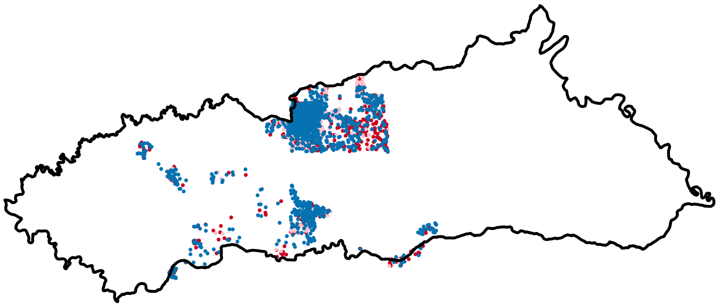

5−022.03 TURLOCK

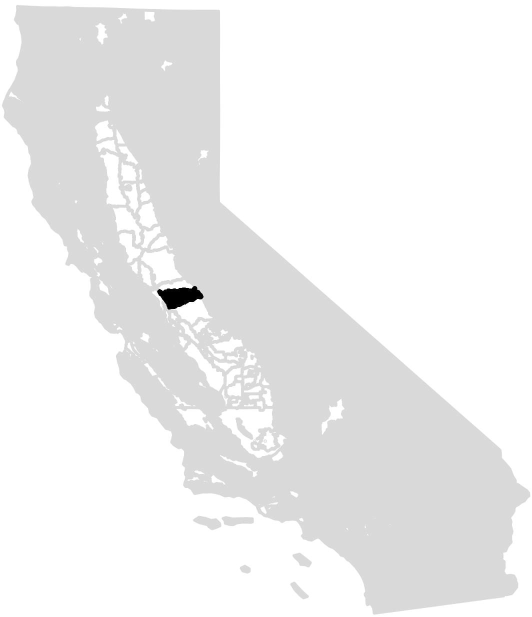

AGRICULTURE

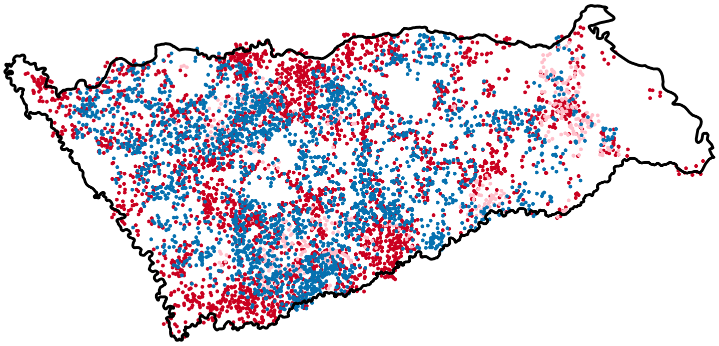

DOMESTIC

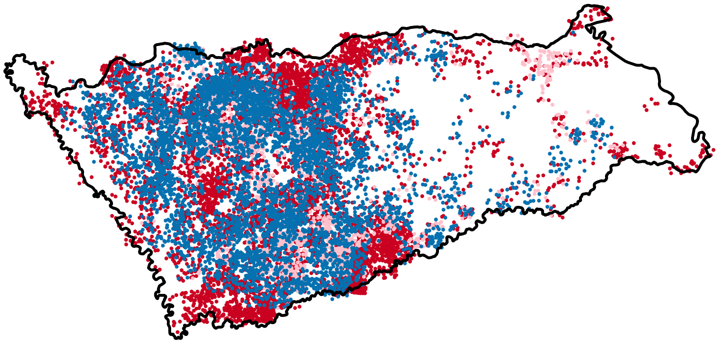

ENVIRONMENT

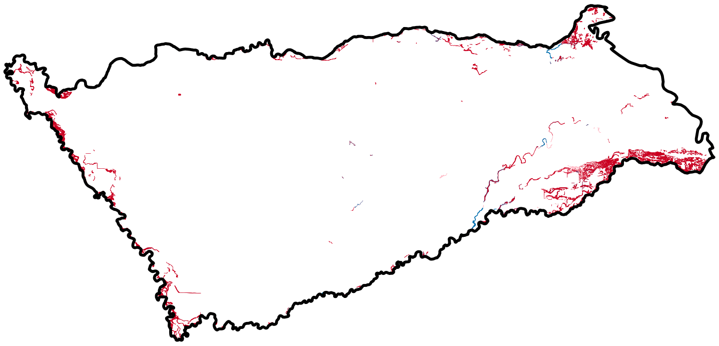

DISADVANTAGED COMMUNITIES

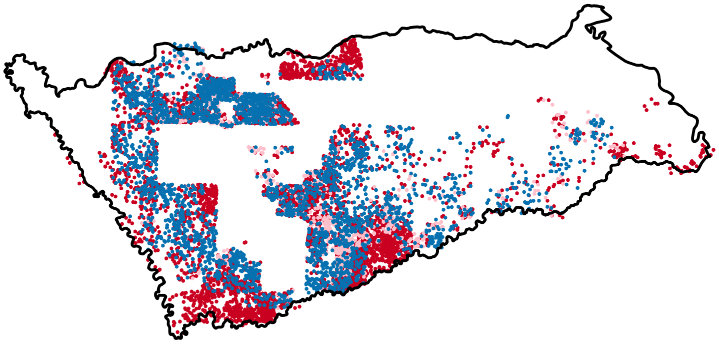

5−022.04 MERCED

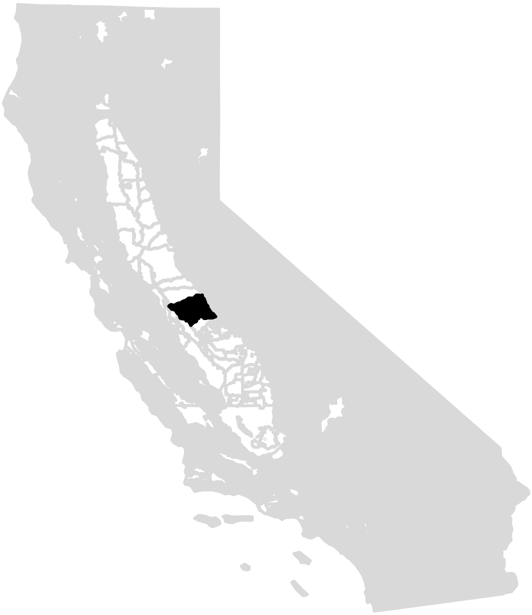

AGRICULTURE

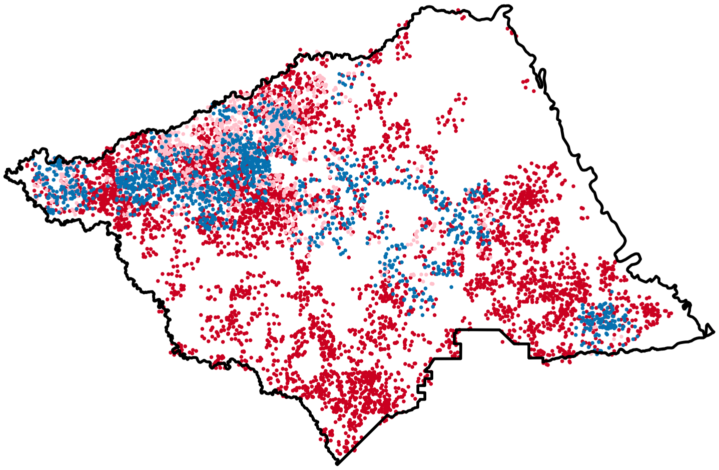

DOMESTIC

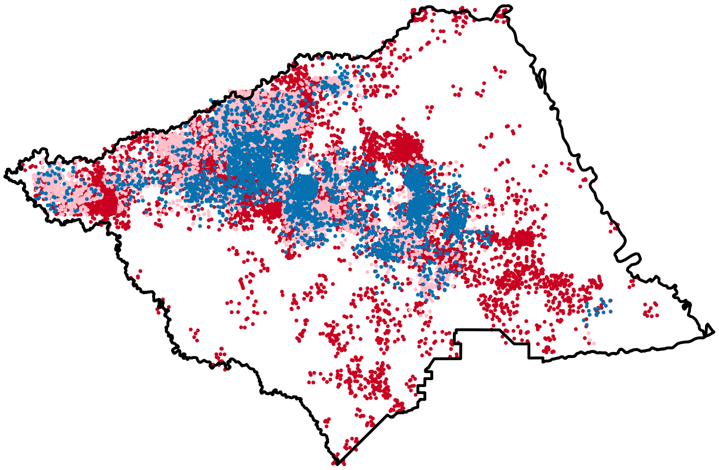

ENVIRONMENT

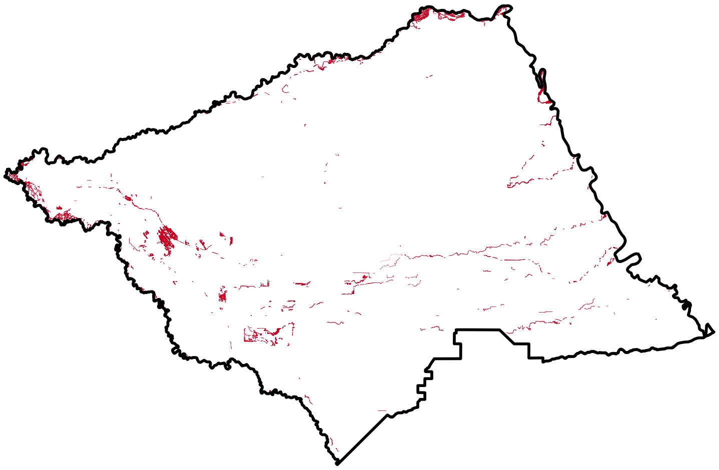

DISADVANTAGED COMMUNITIES

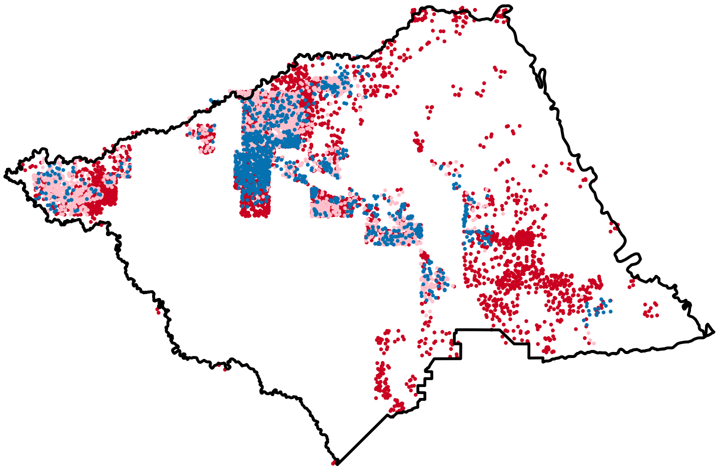

5−022.05 CHOWCHILLA

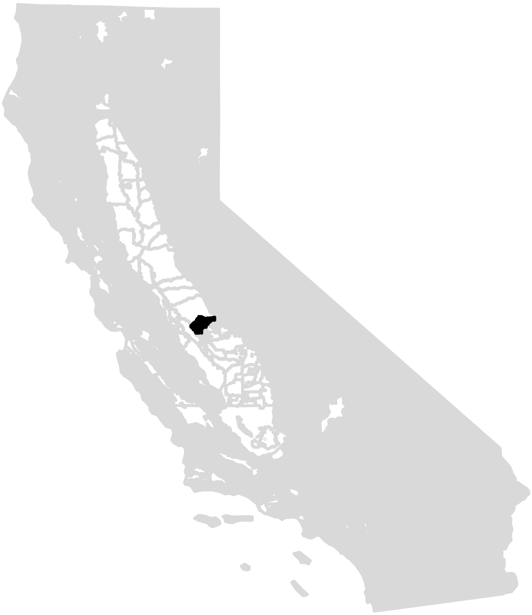

AGRICULTURE

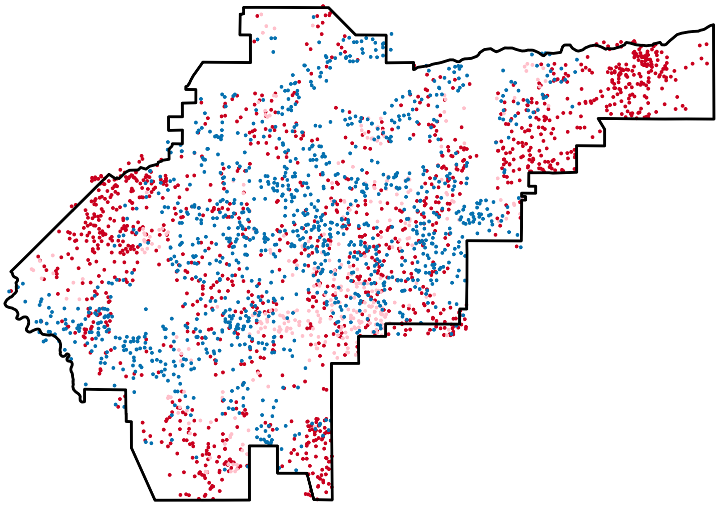

DOMESTIC

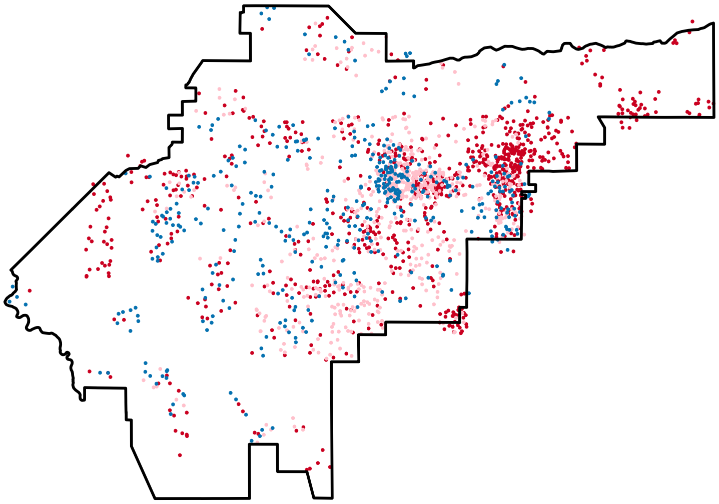

ENVIRONMENT

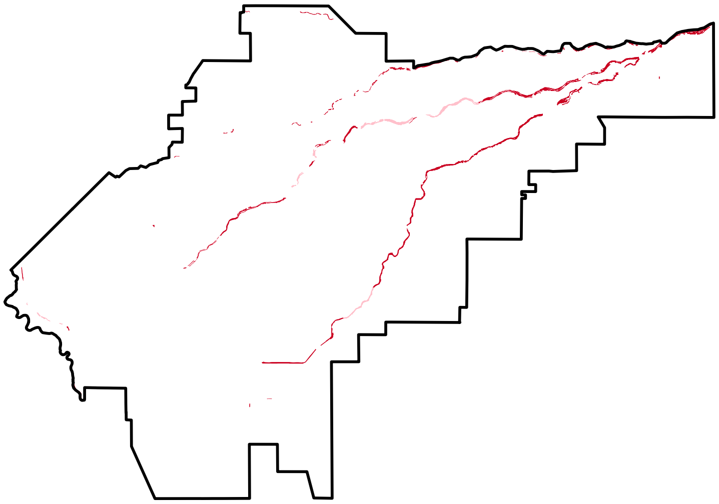

DISADVANTAGED COMMUNITIES

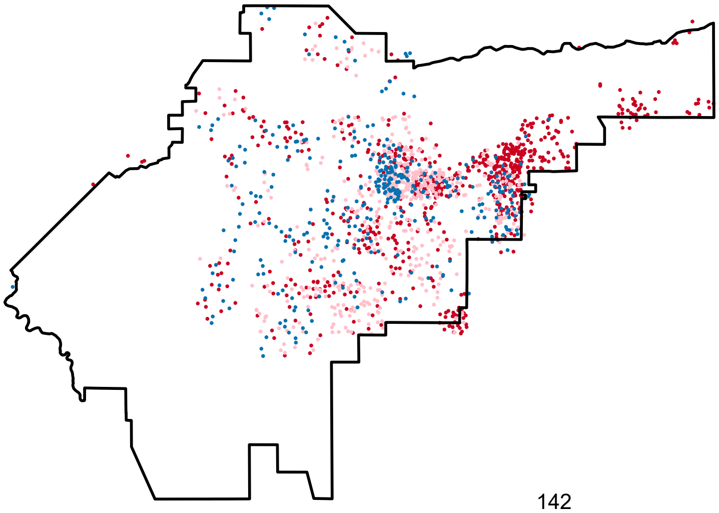

5−022.06 MADERA

AGRICULTURE

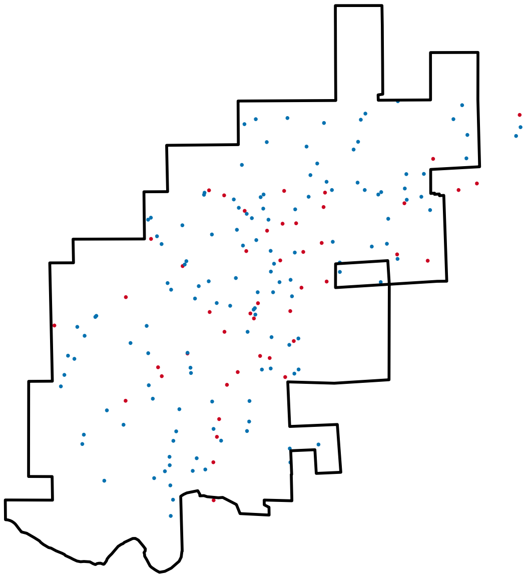

DOMESTIC

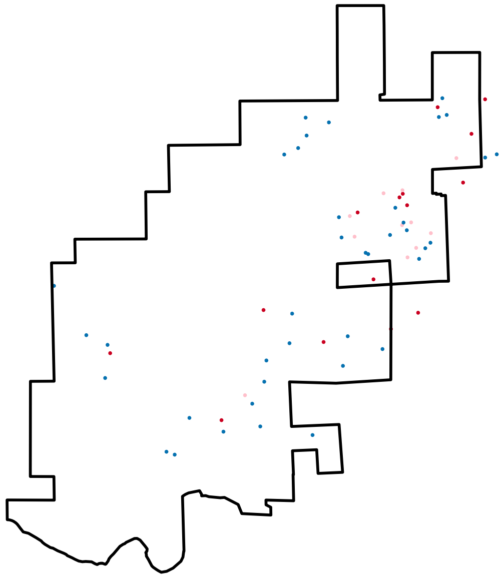

ENVIRONMENT

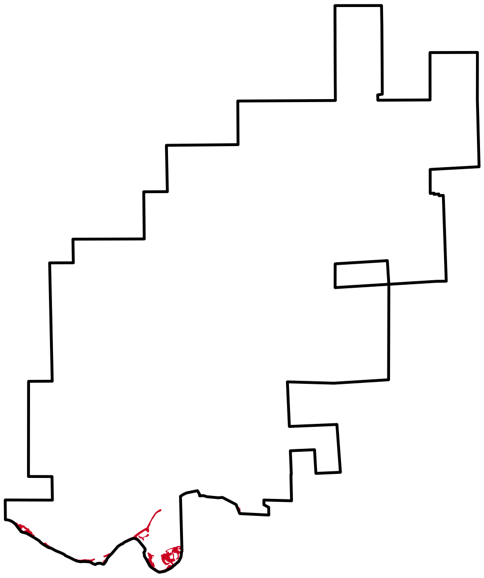

DISADVANTAGED COMMUNITIES

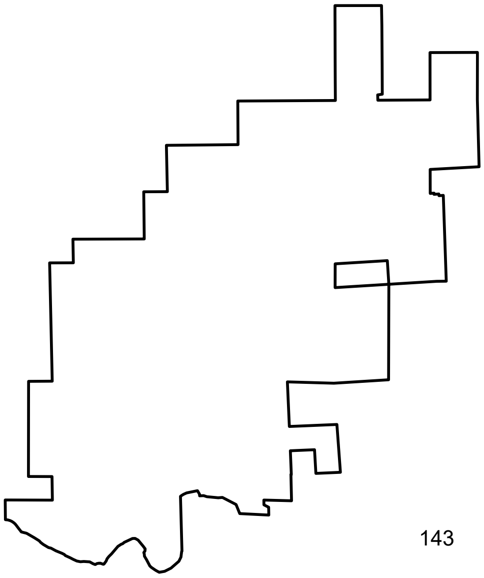

5−022.07 DELTA−MENDOTA

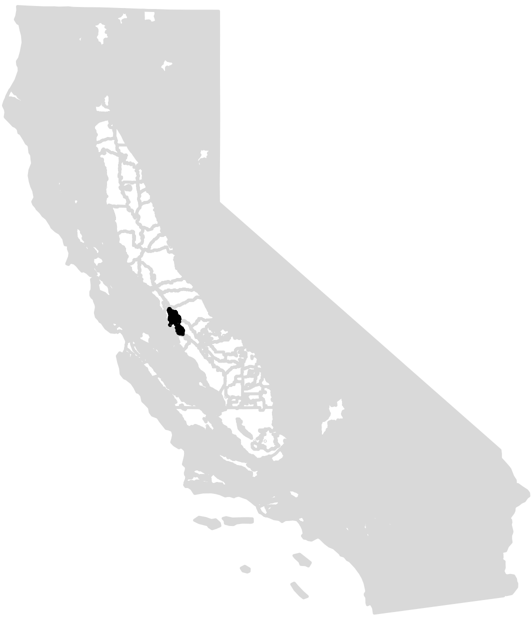

AGRICULTURE

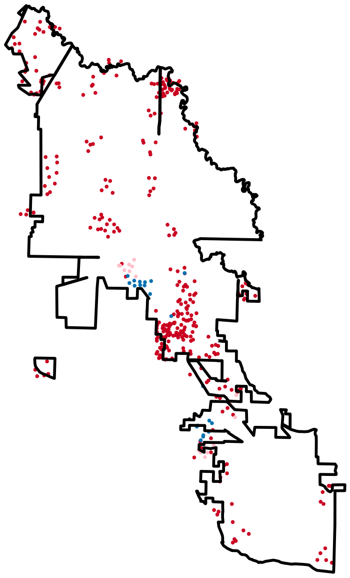

DOMESTIC

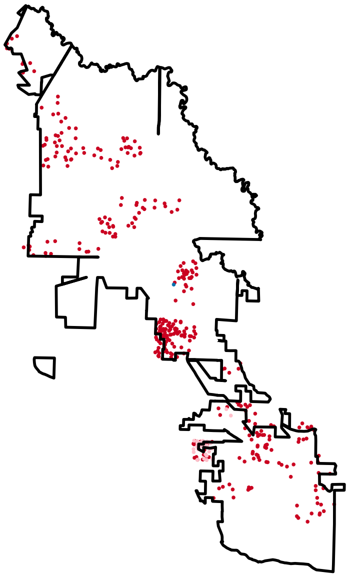

ENVIRONMENT

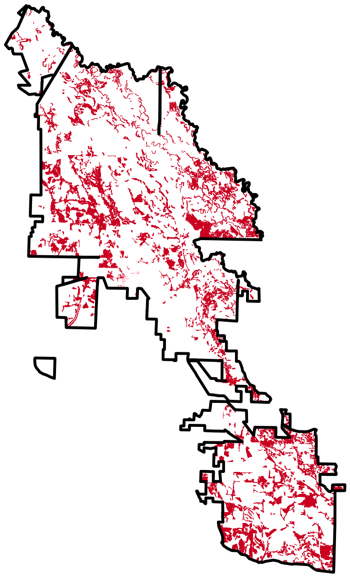

DISADVANTAGED COMMUNITIES

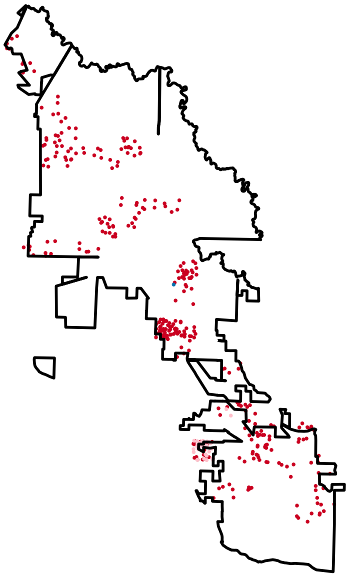

5−022.08 KINGS

AGRICULTURE

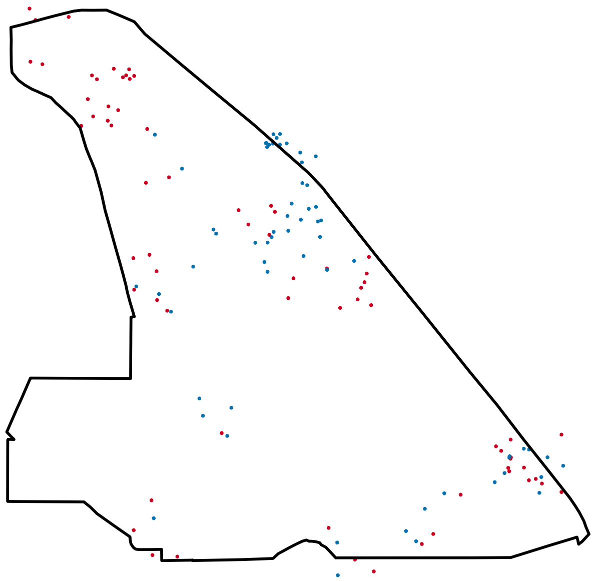

DOMESTIC

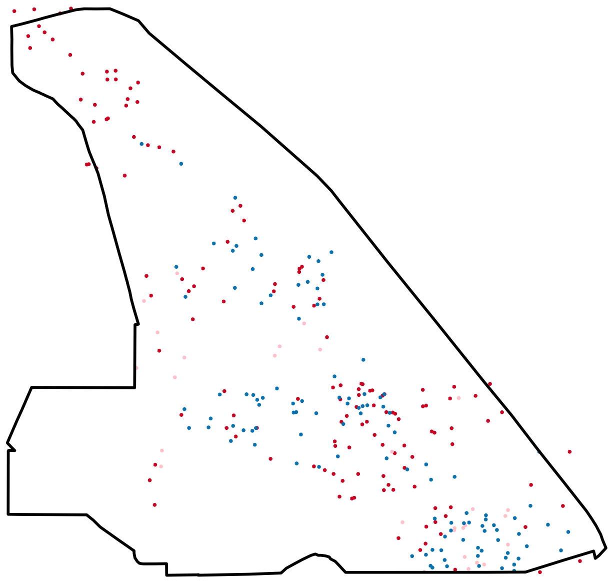

ENVIRONMENT

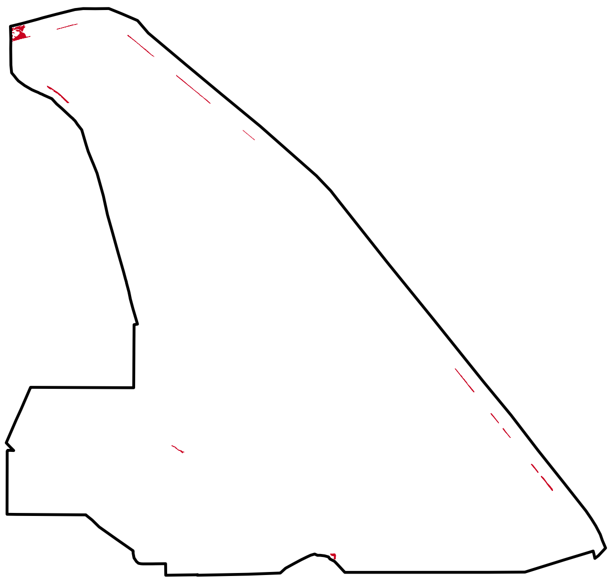

DISADVANTAGED COMMUNITIES

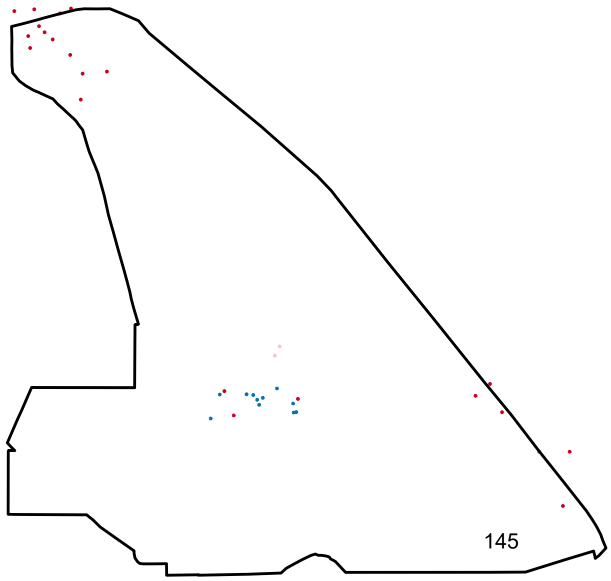

5−022.09 WESTSIDE

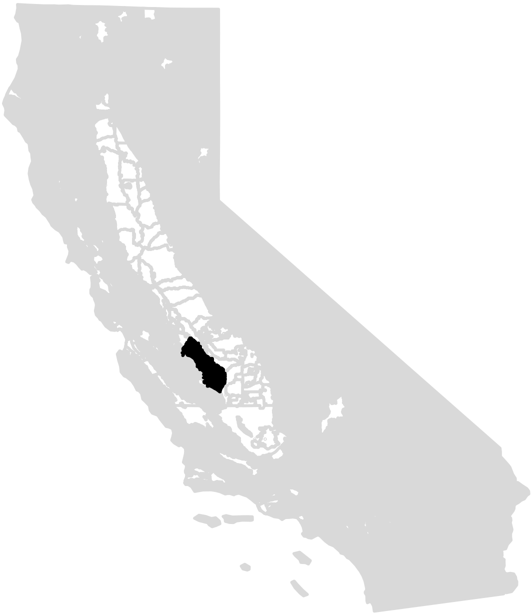

AGRICULTURE

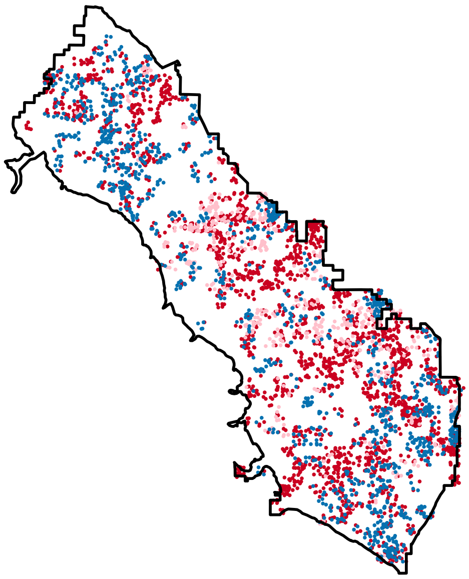

DOMESTIC

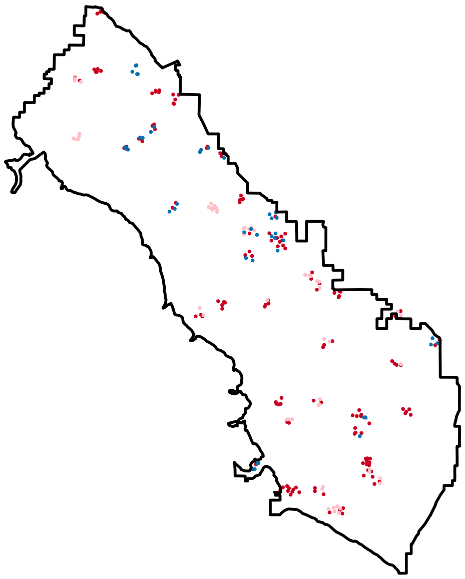

ENVIRONMENT

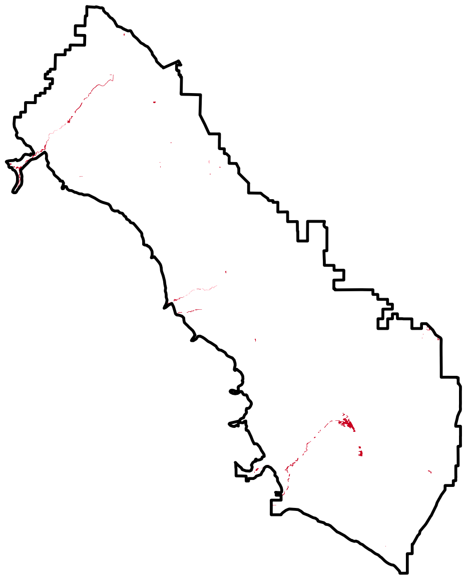

DISADVANTAGED COMMUNITIES

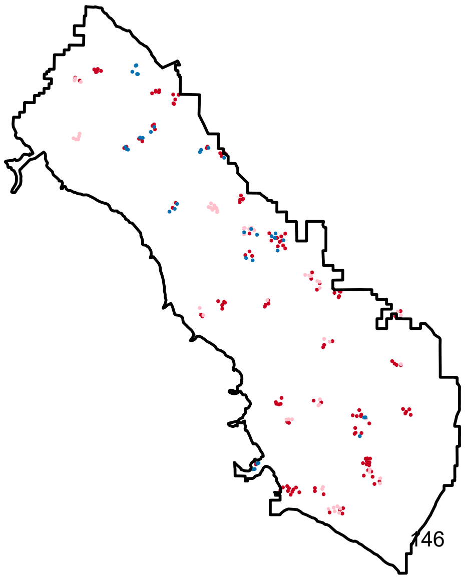

5−022.10 PLEASANT VALLEY

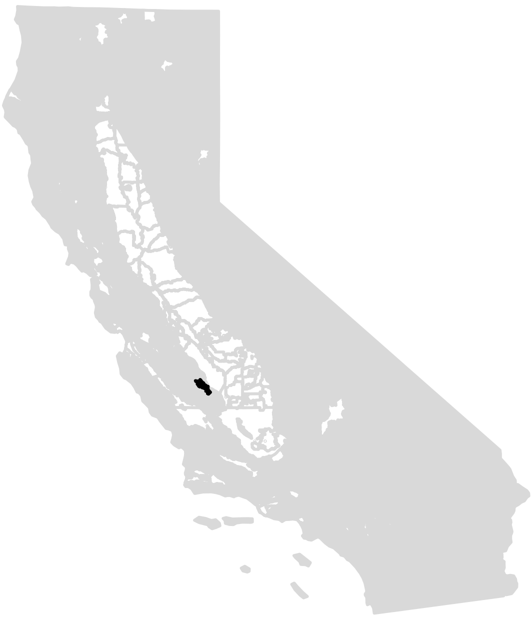

AGRICULTURE

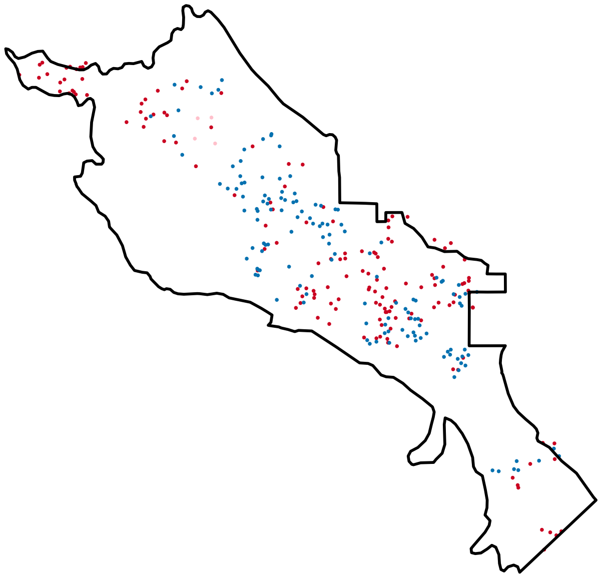

DOMESTIC

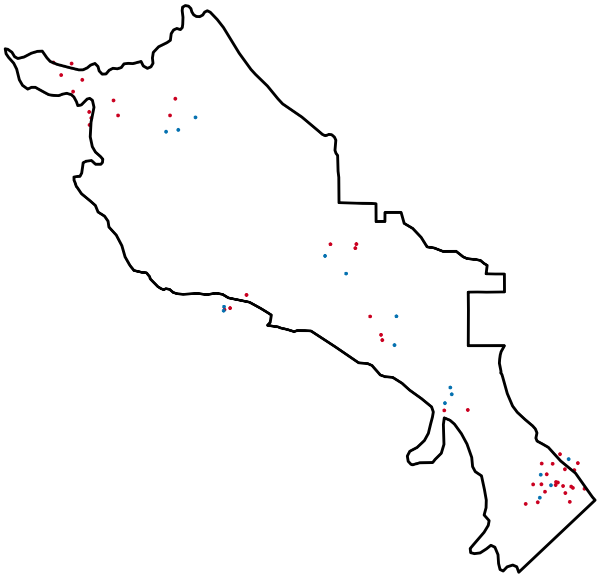

ENVIRONMENT

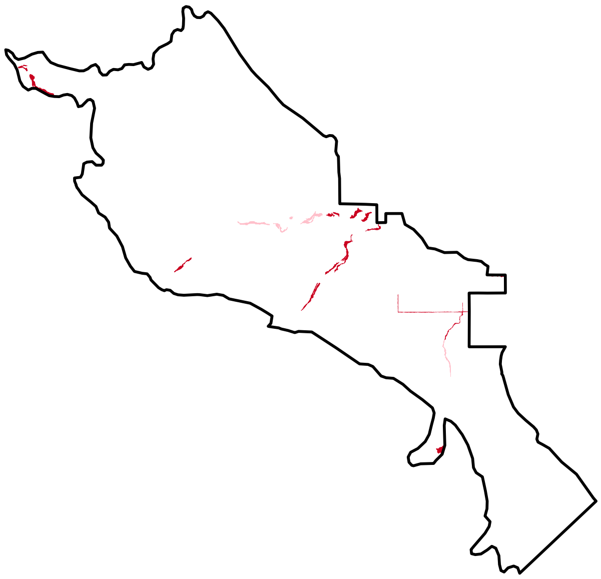

DISADVANTAGED COMMUNITIES

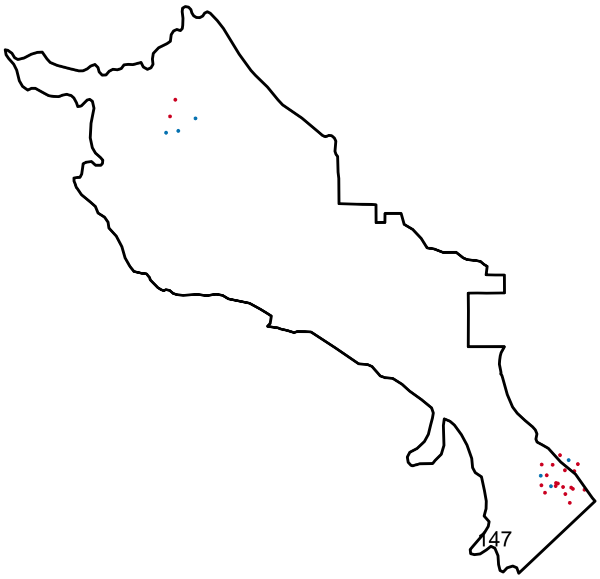

5−022.11 KAWEAH

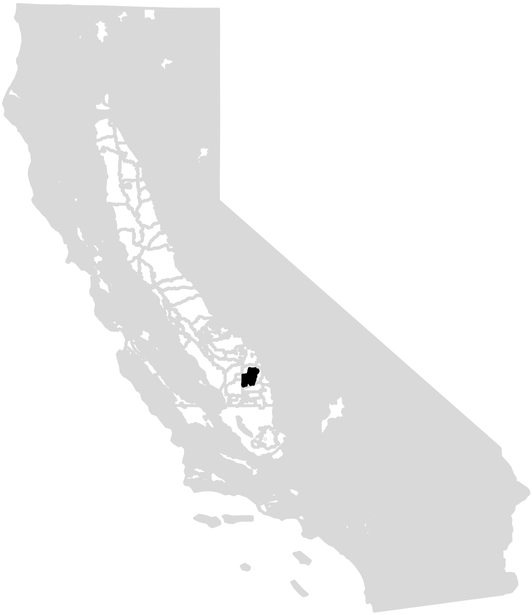

AGRICULTURE

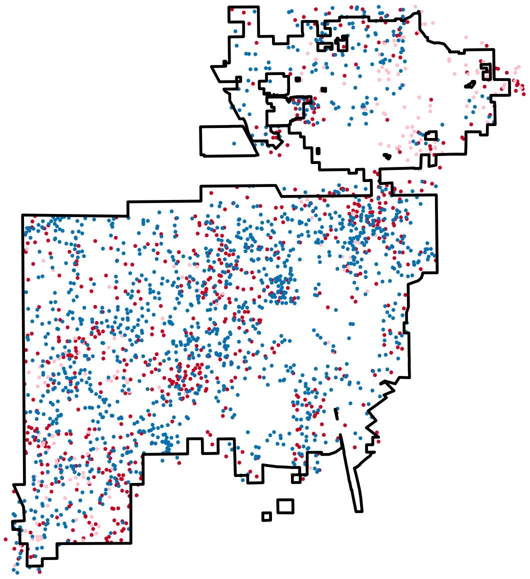

DOMESTIC

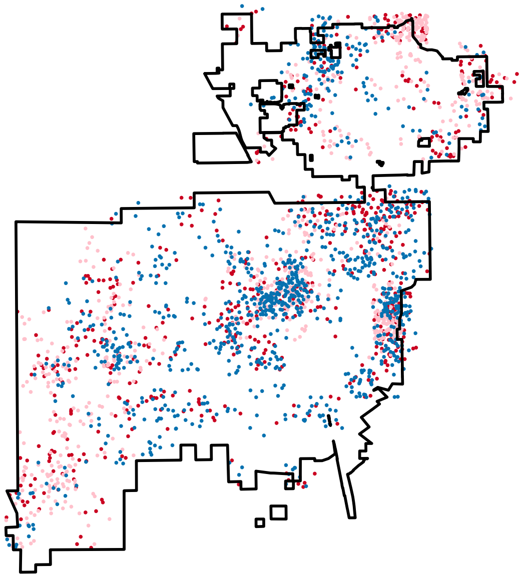

ENVIRONMENT

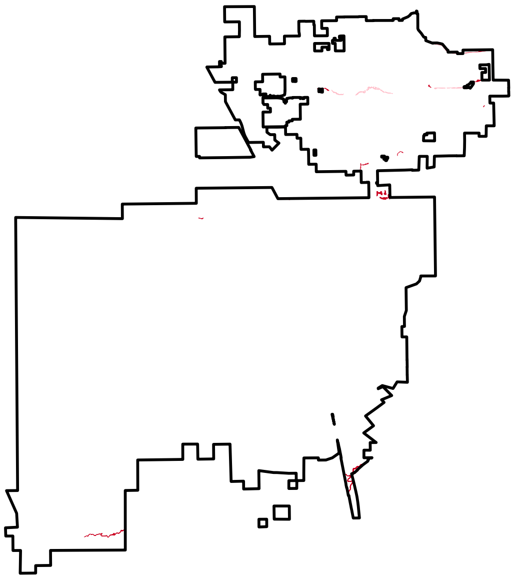

DISADVANTAGED COMMUNITIES

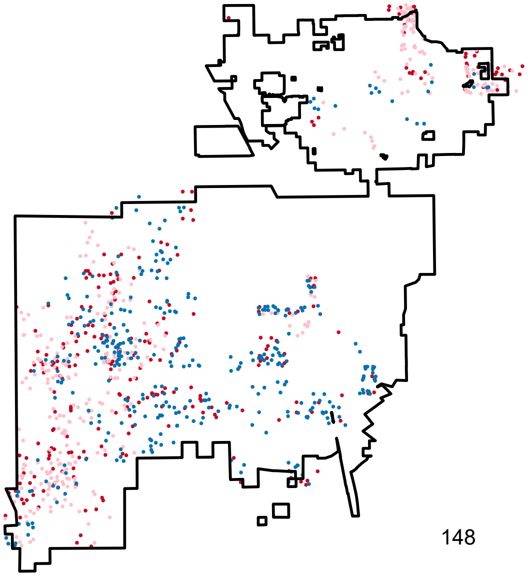

5−022.12 TULARE LAKE

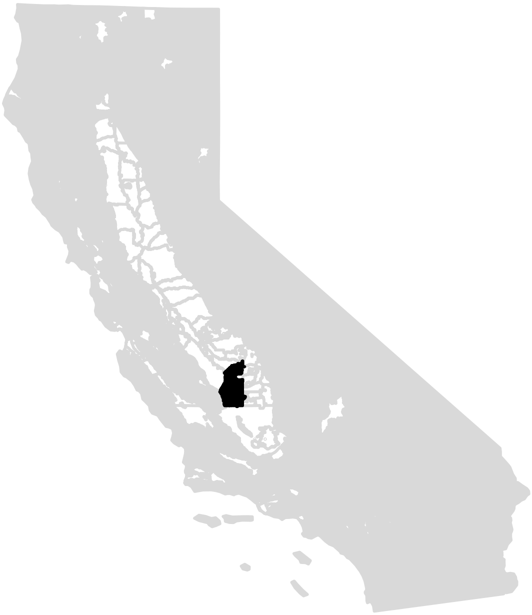

AGRICULTURE

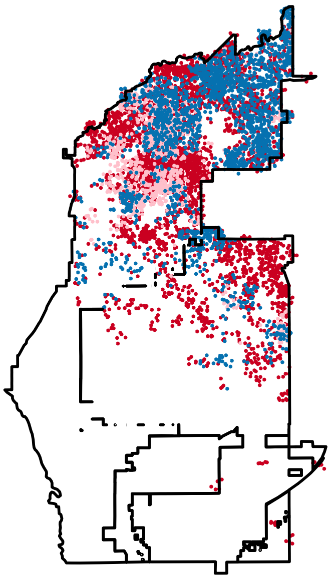

DOMESTIC

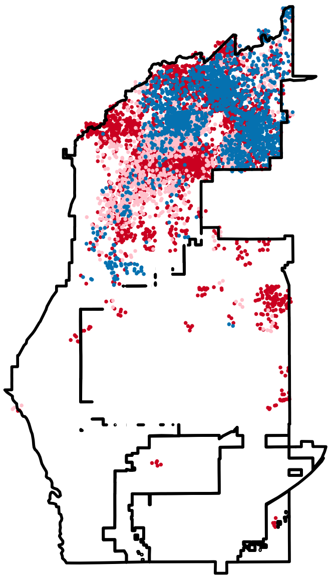

ENVIRONMENT

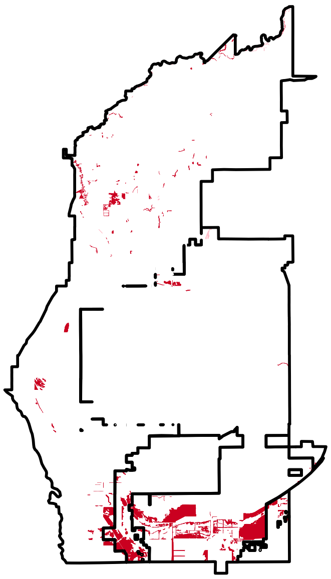

DISADVANTAGED COMMUNITIES

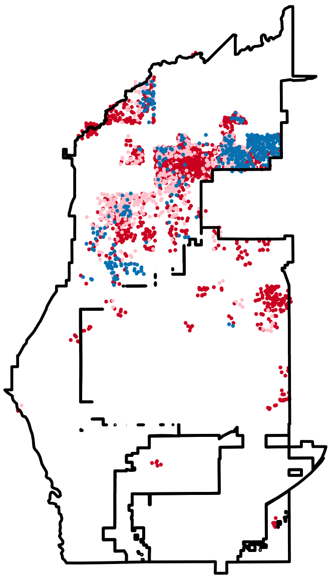

5−022.13 TULE

AGRICULTURE

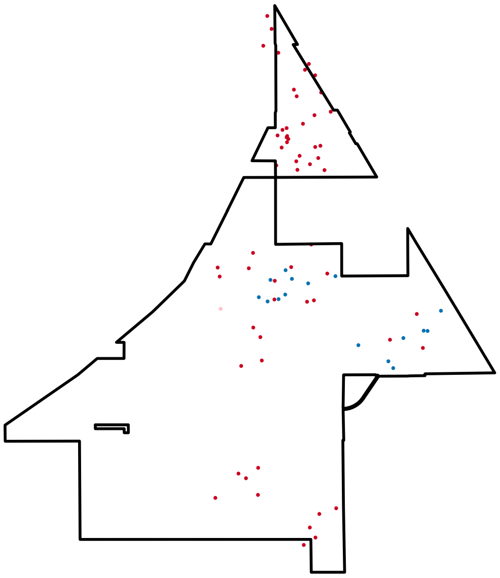

DOMESTIC

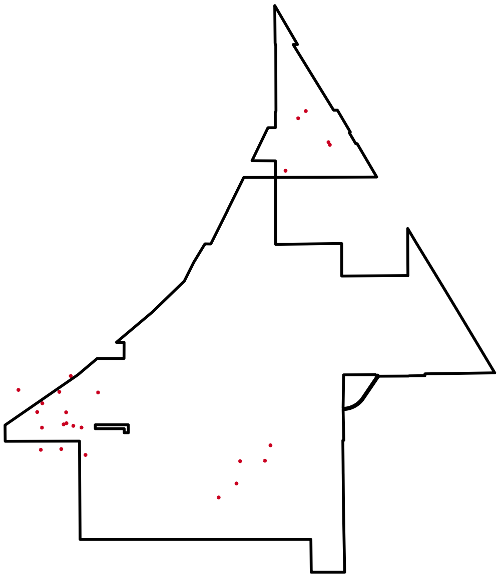

ENVIRONMENT

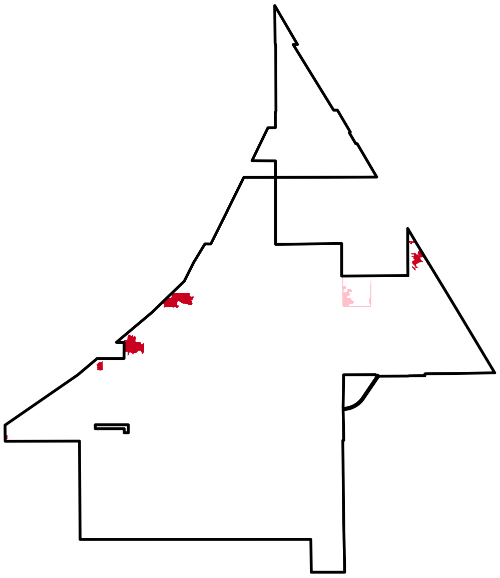

DISADVANTAGED COMMUNITIES

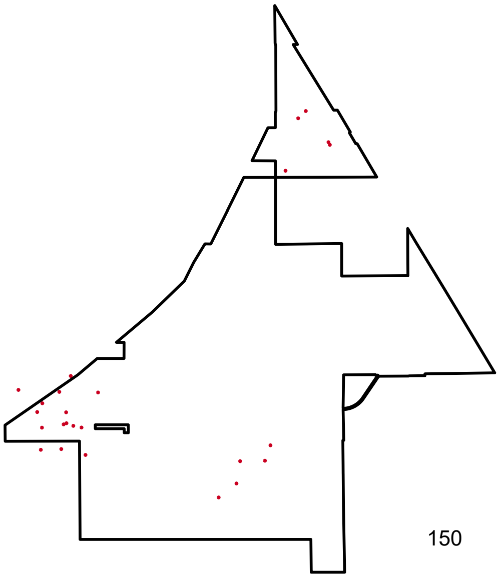

5−022.14 KERN COUNTY

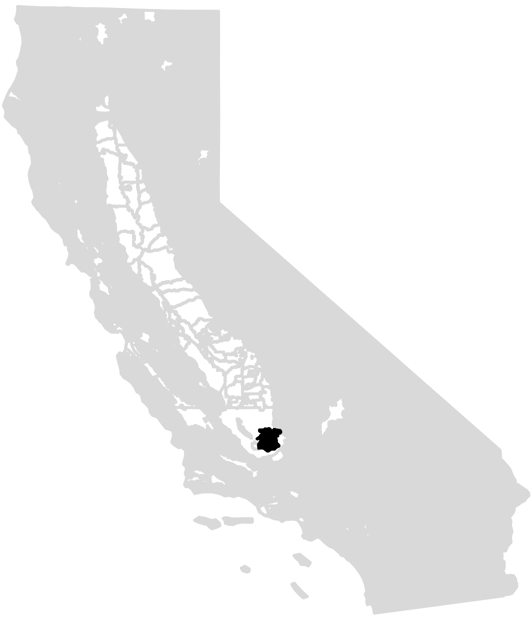

AGRICULTURE

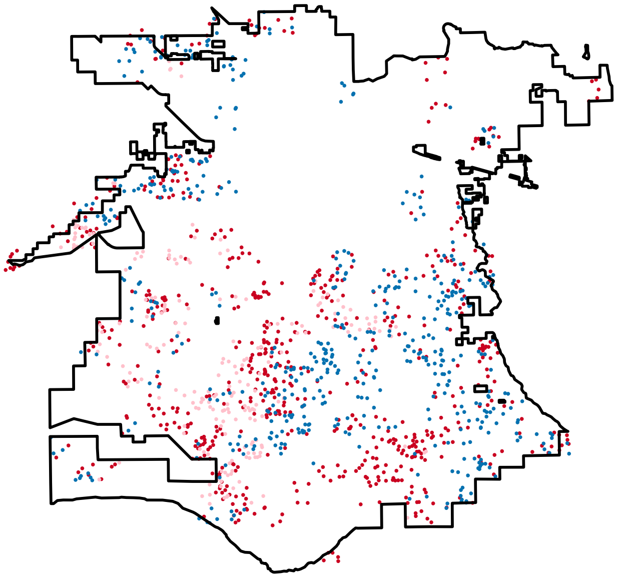

DOMESTIC

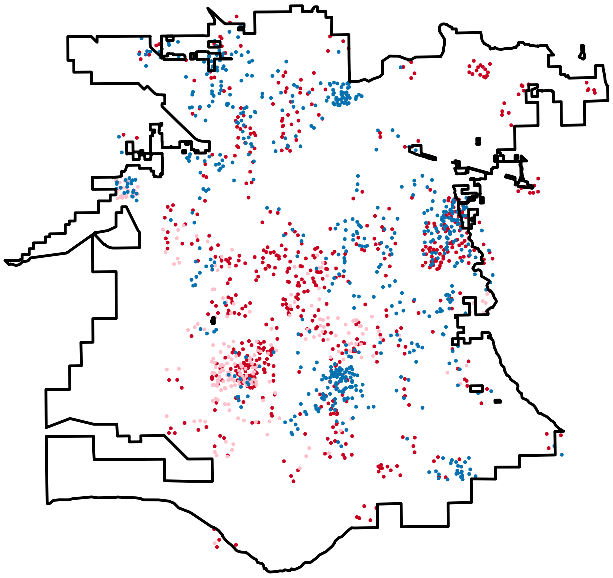

ENVIRONMENT

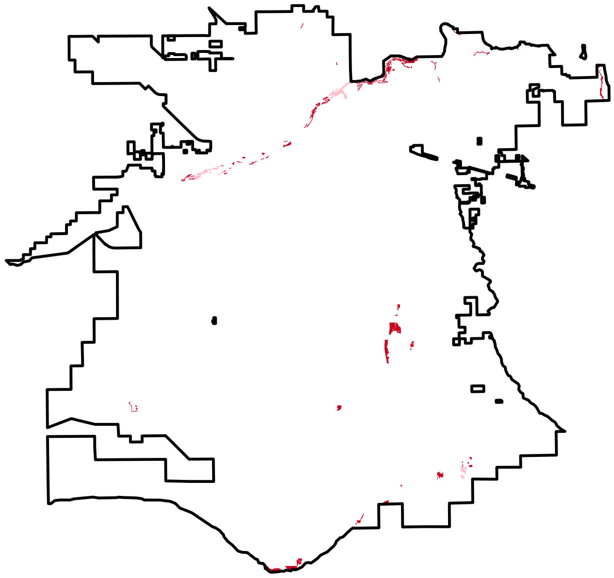

DISADVANTAGED COMMUNITIES

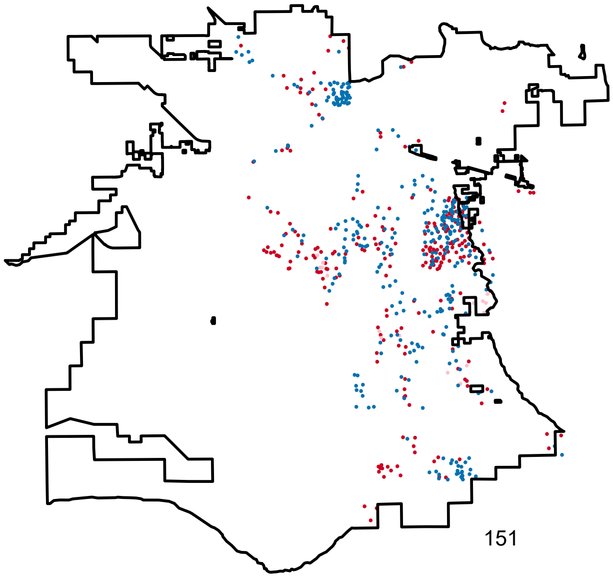

5−022.15 TRACY

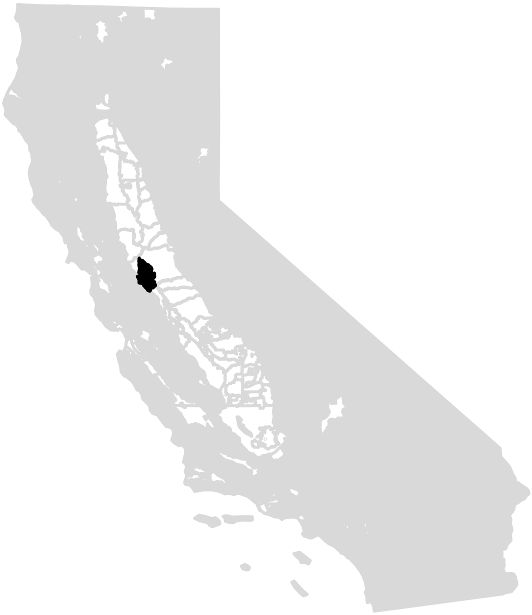

AGRICULTURE

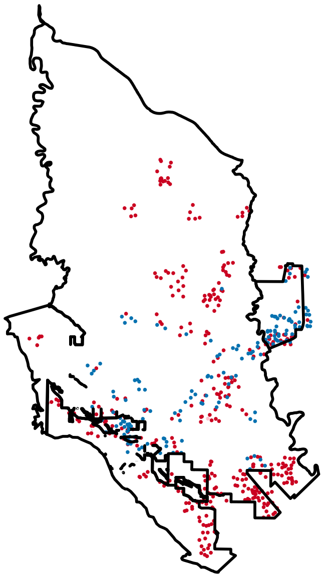

DOMESTIC

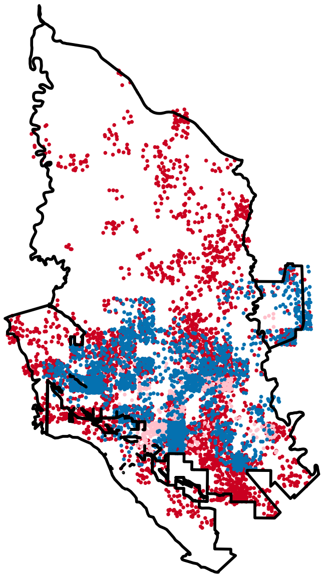

ENVIRONMENT

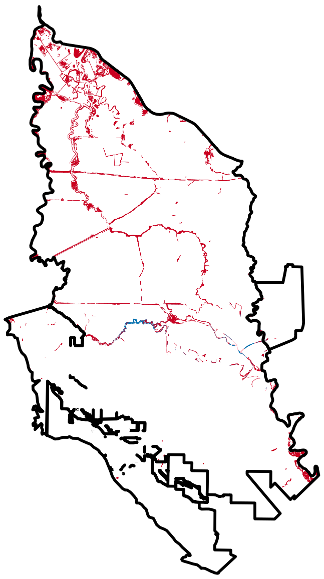

DISADVANTAGED COMMUNITIES

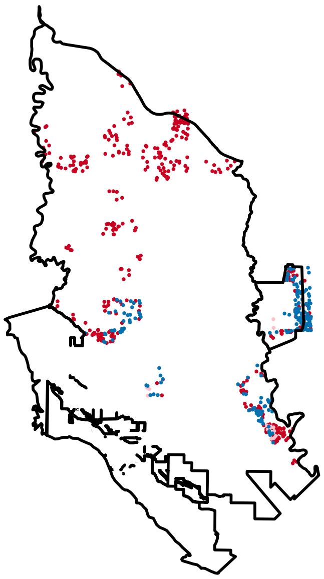

5−022.16 COSUMNES

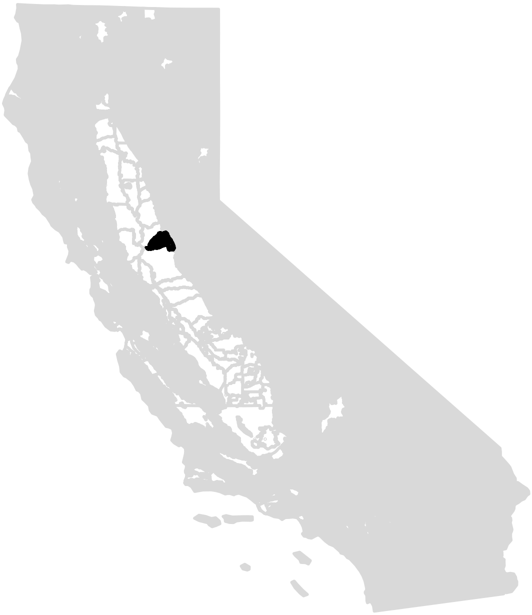

AGRICULTURE

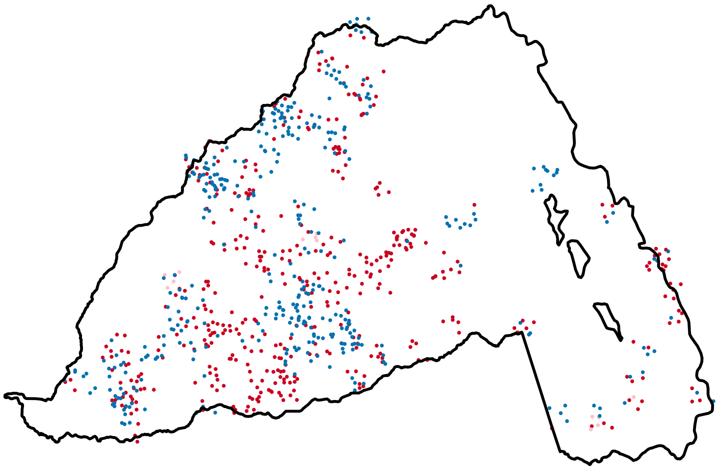

DOMESTIC

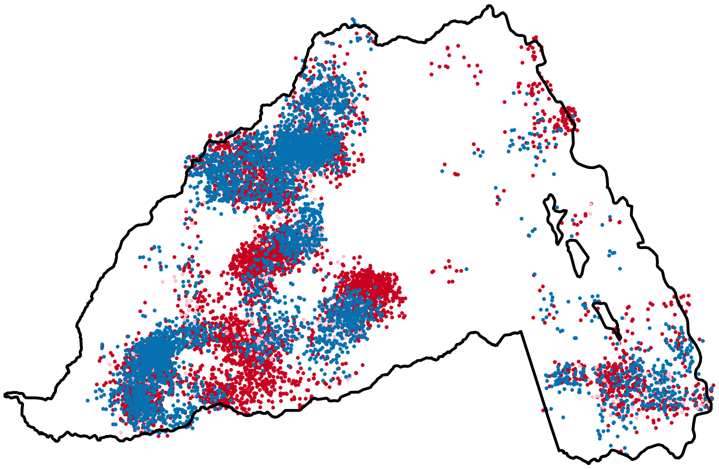

ENVIRONMENT

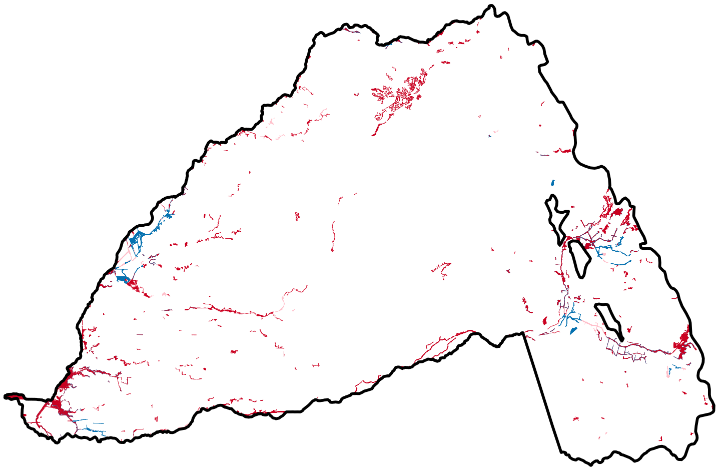

DISADVANTAGED COMMUNITIES

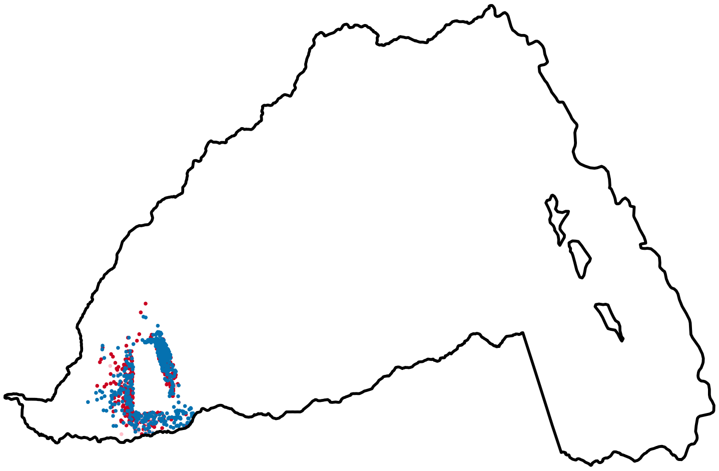

5−022.18 WHITE WOLF

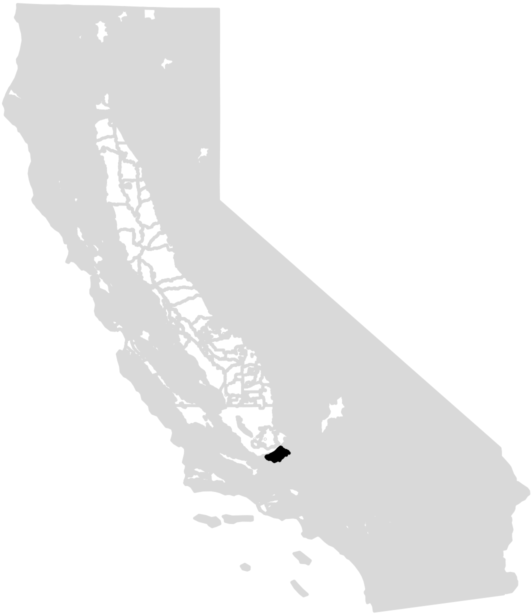

AGRICULTURE

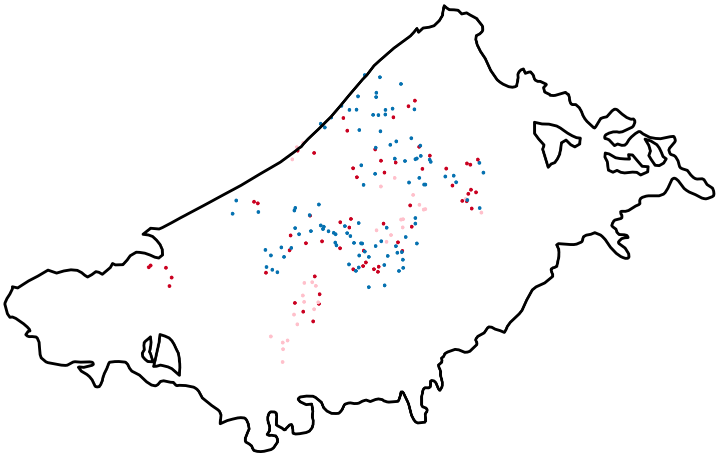

DOMESTIC

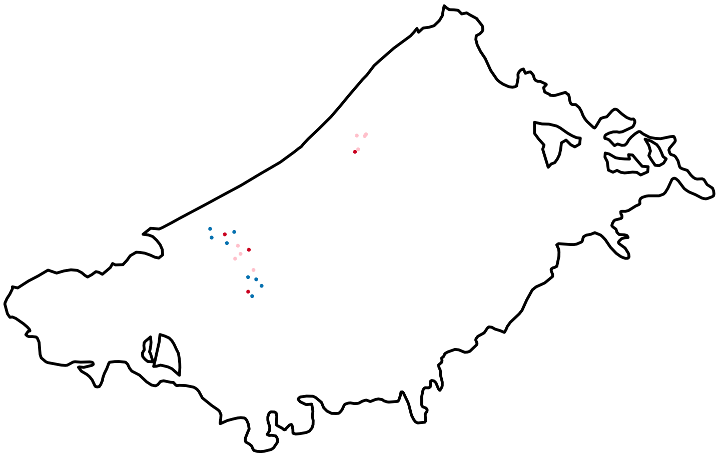

ENVIRONMENT

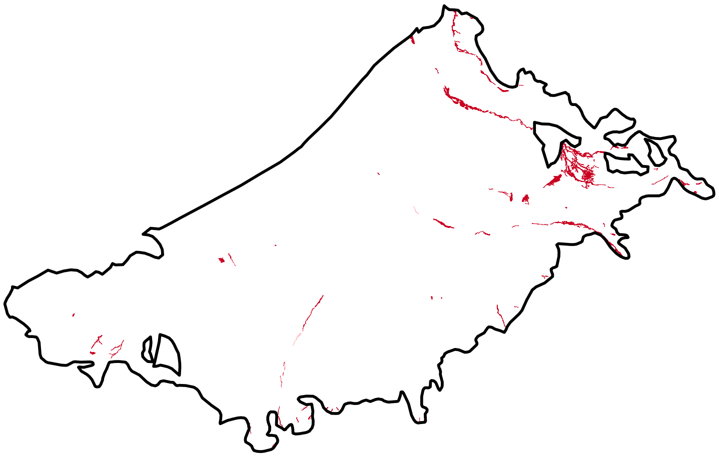

DISADVANTAGED COMMUNITIES

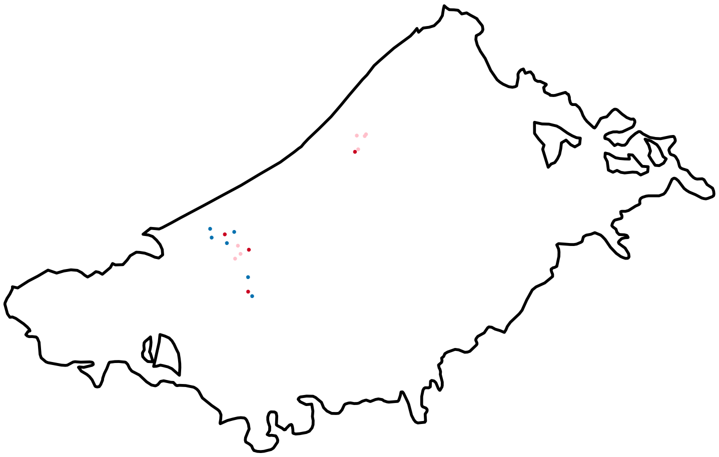

5−022.19 EAST CONTRA COSTA

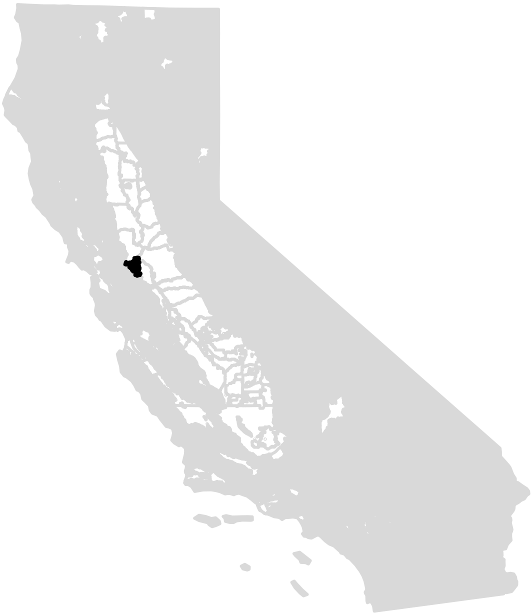

AGRICULTURE

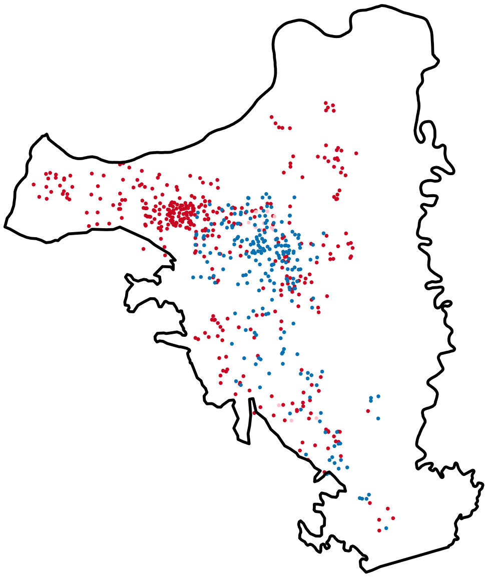

DOMESTIC

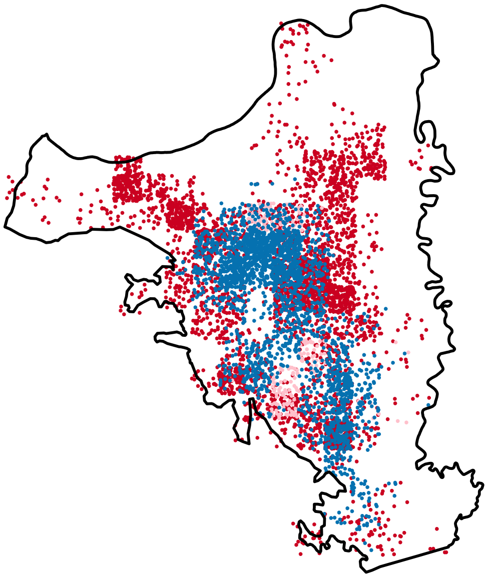

ENVIRONMENT

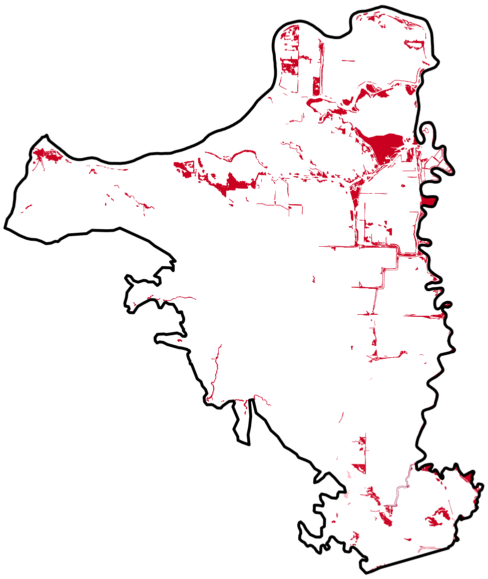

DISADVANTAGED COMMUNITIES

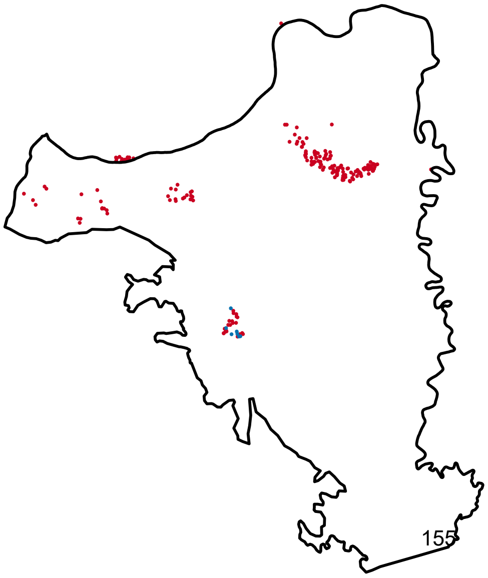

6−054 INDIAN WELLS VALLEY

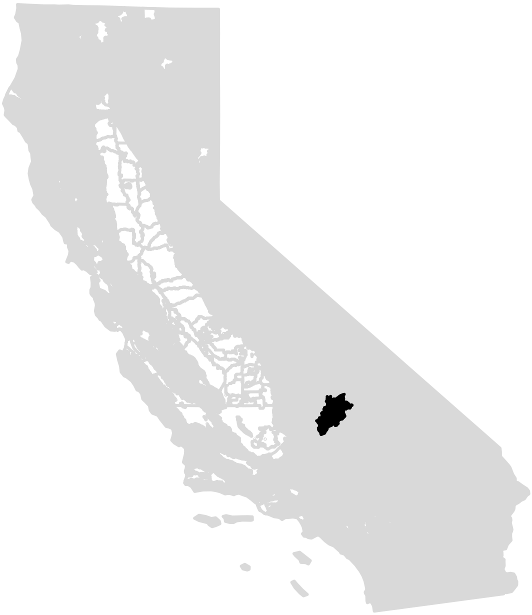

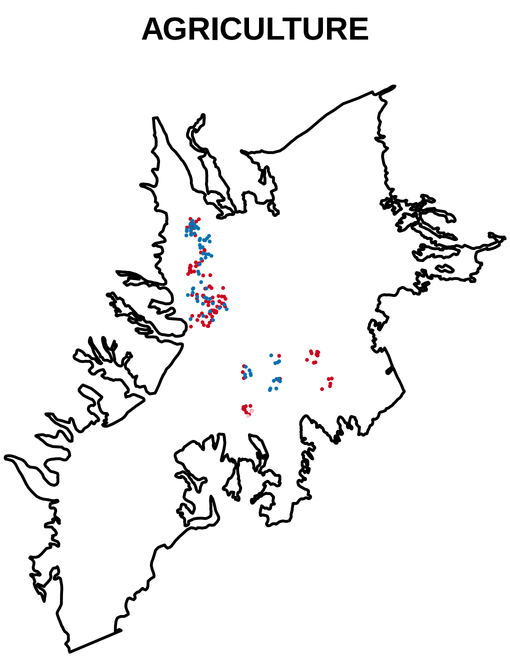

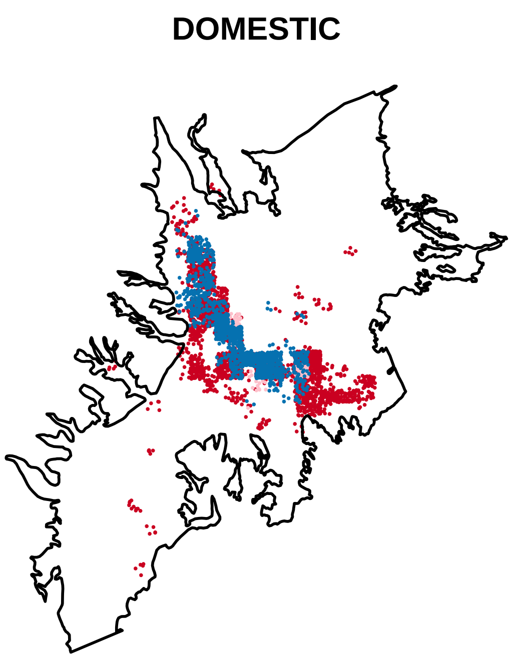

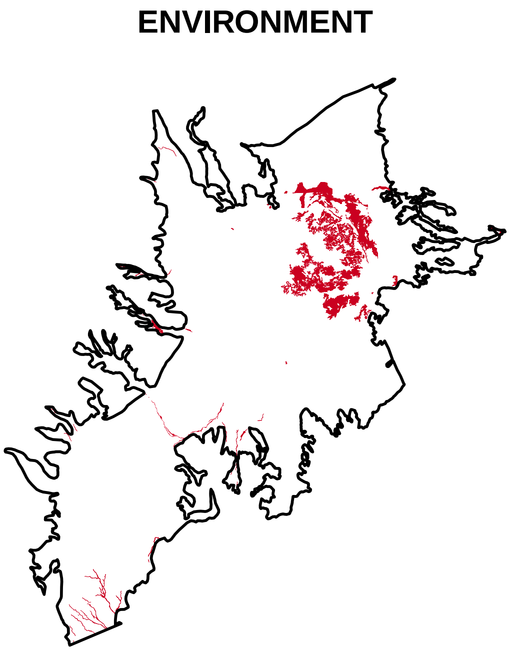

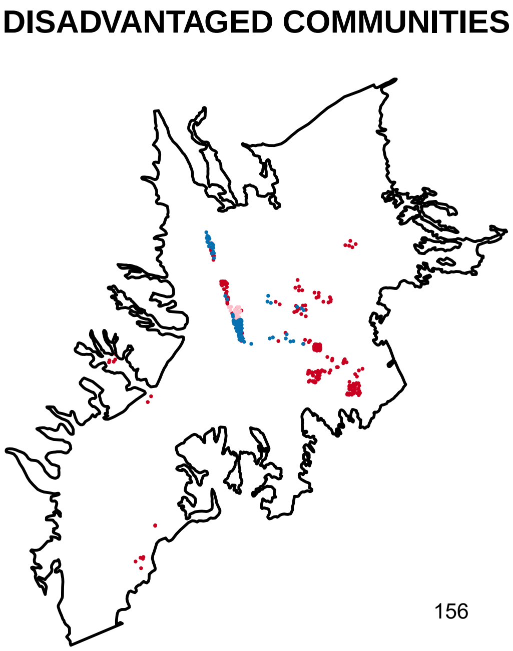

7−021.04 SAN GORGONIO PASS

AGRICULTURE

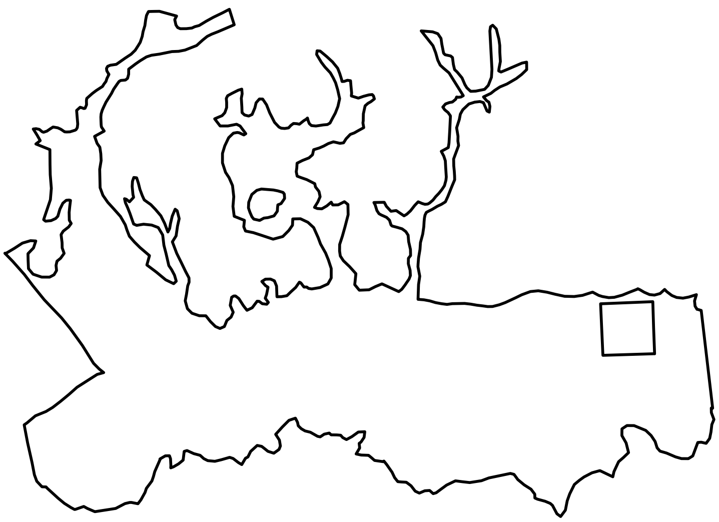

DOMESTIC

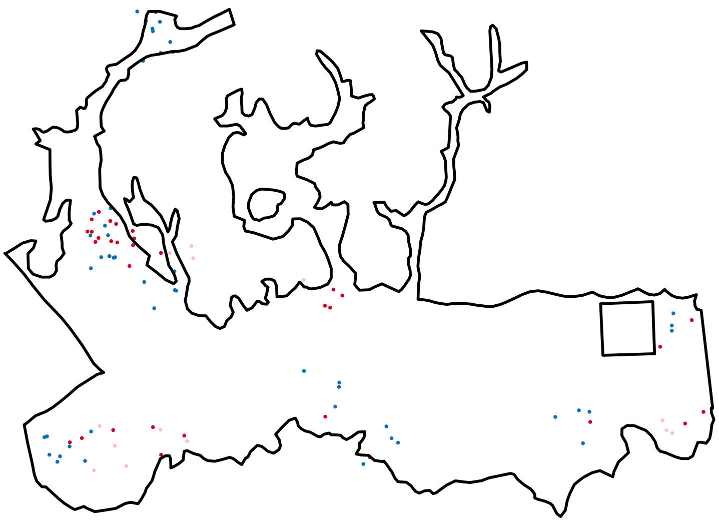

ENVIRONMENT

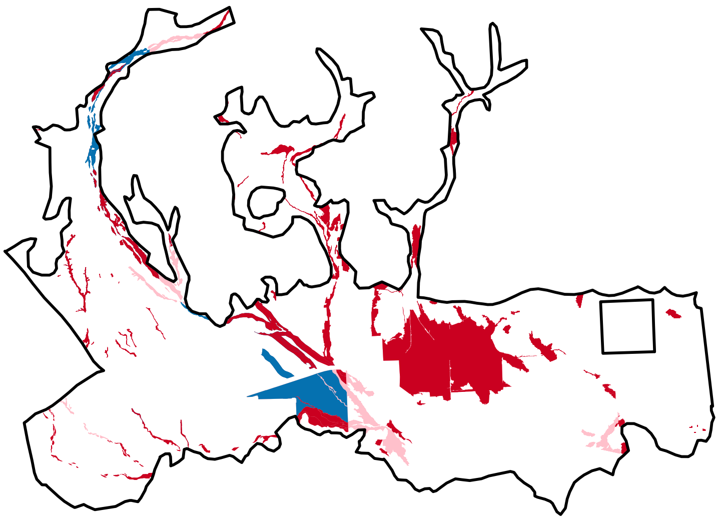

DISADVANTAGED COMMUNITIES

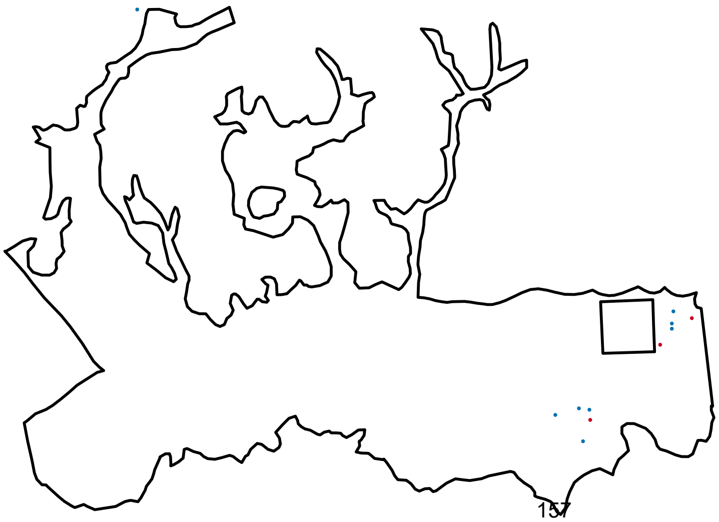

8−002.07 YUCAIPA

AGRICULTURE

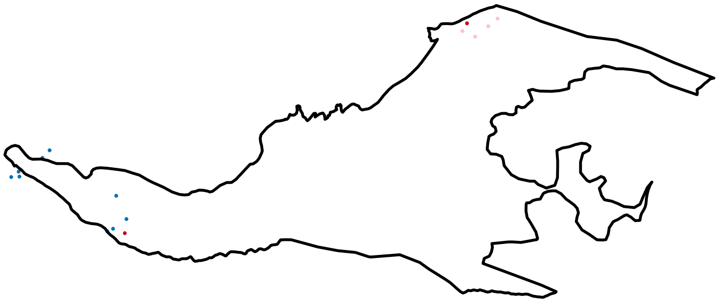

DOMESTIC

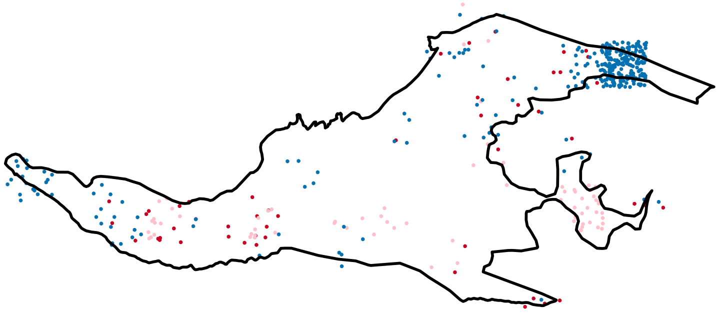

ENVIRONMENT

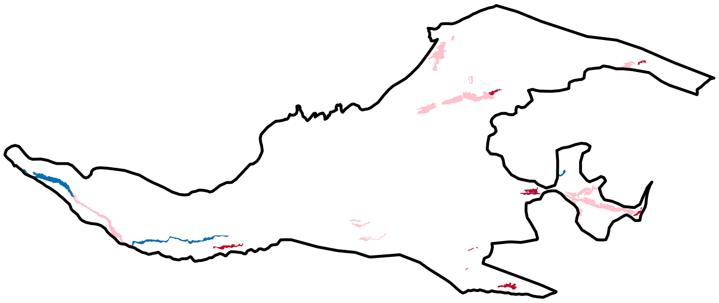

DISADVANTAGED COMMUNITIES

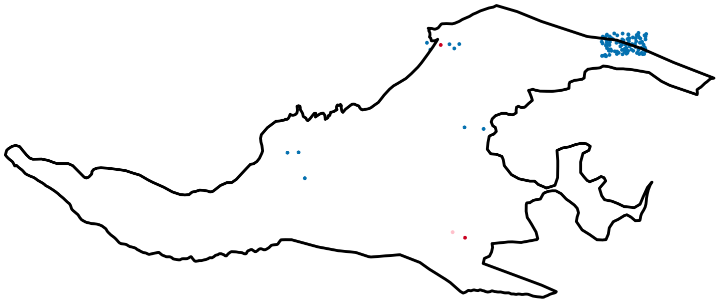

8−002.09 TEMESCAL

AGRICULTURE

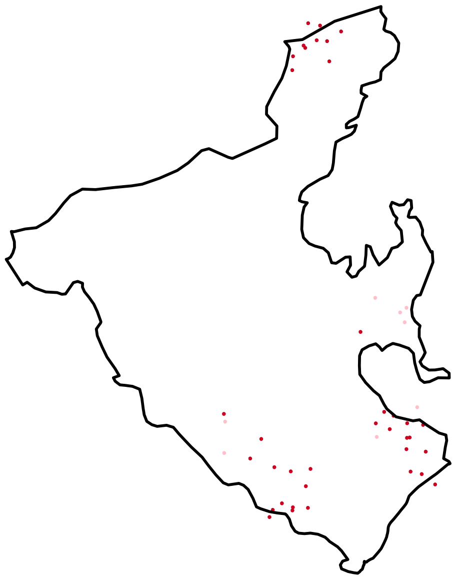

DOMESTIC

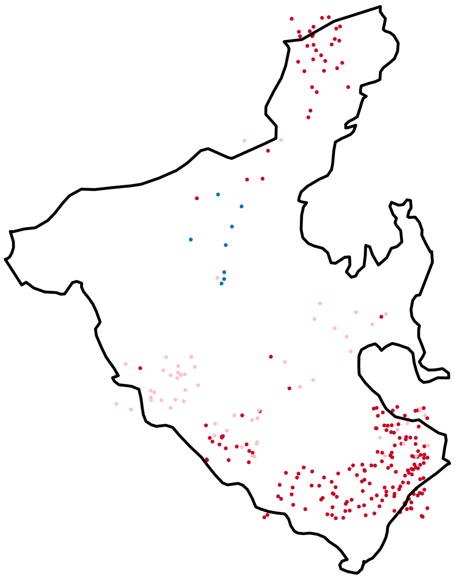

ENVIRONMENT

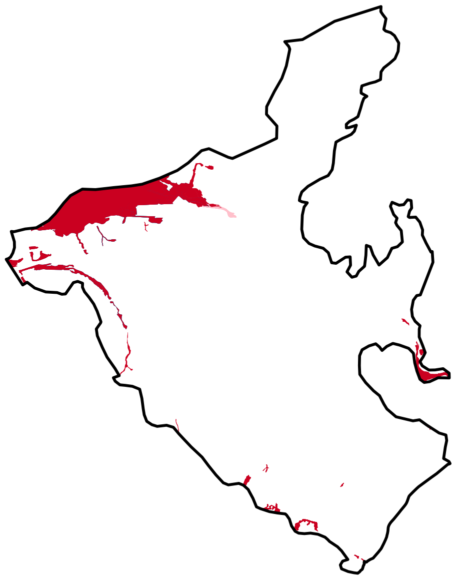

DISADVANTAGED COMMUNITIES

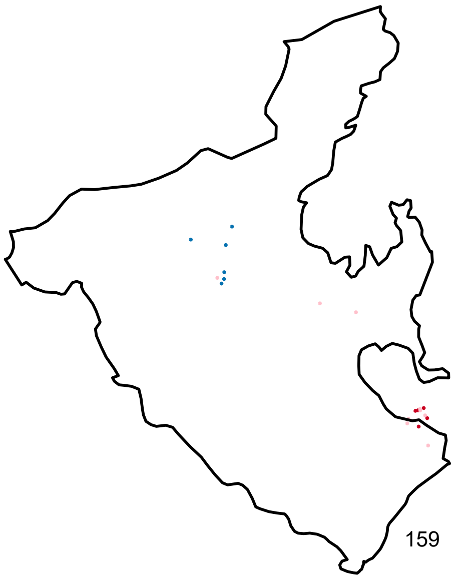

8−004.01 ELSINORE VALLEY

AGRICULTURE

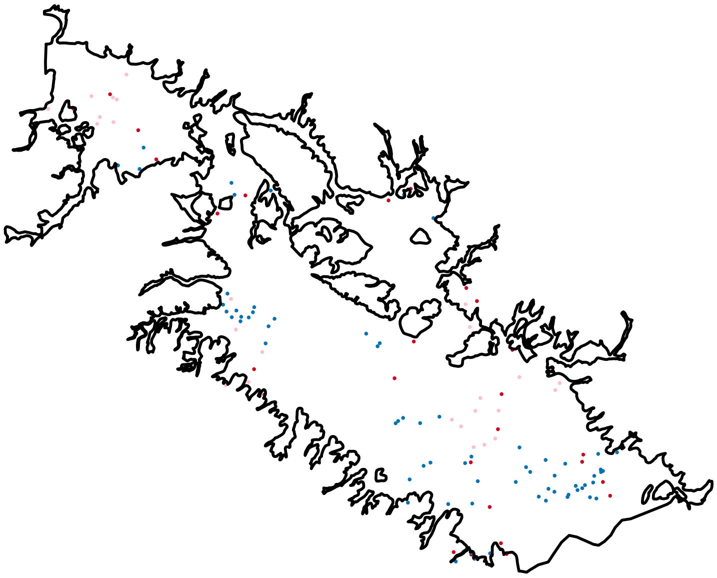

DOMESTIC

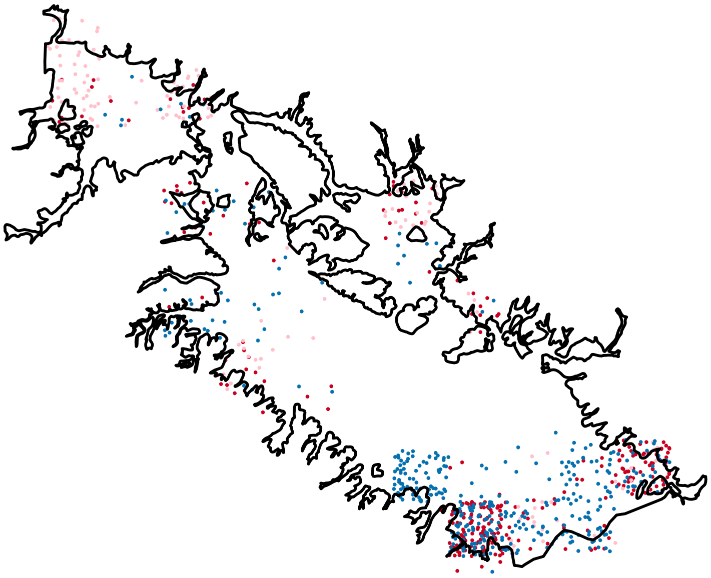

ENVIRONMENT

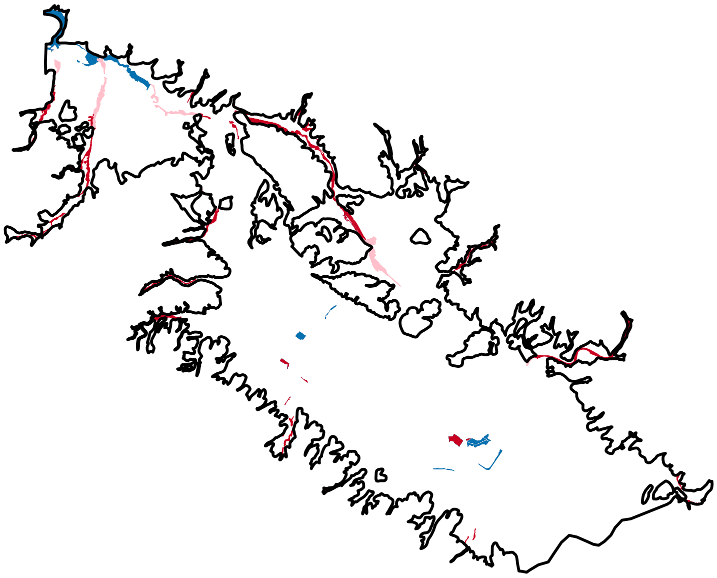

DISADVANTAGED COMMUNITIES

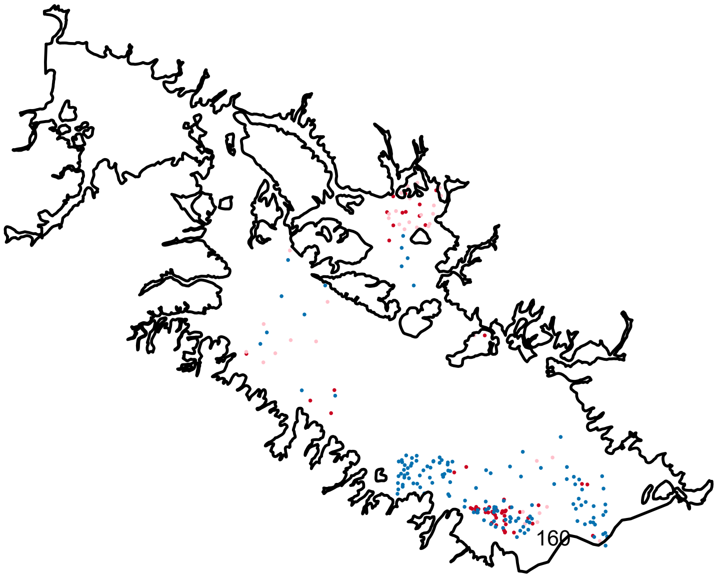

8−005 SAN JACINTO

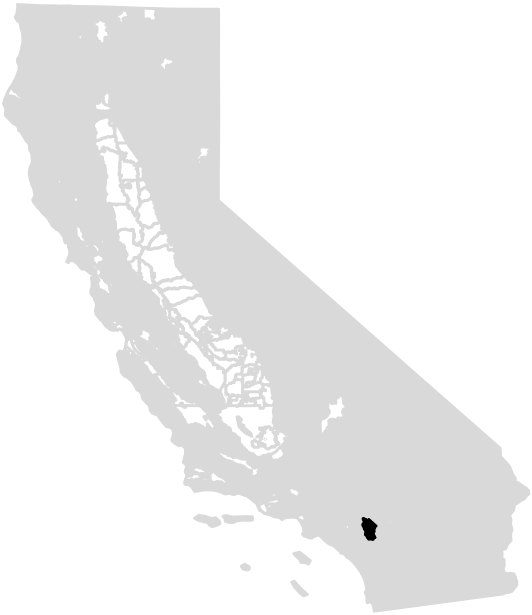

AGRICULTURE

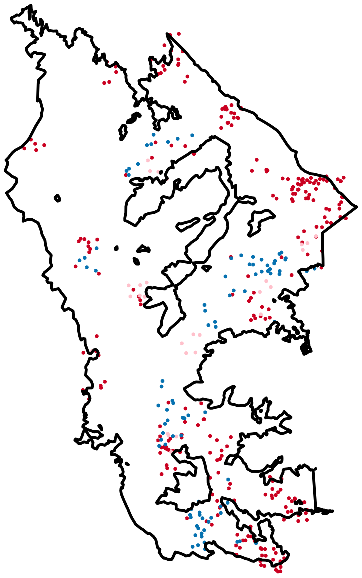

DOMESTIC

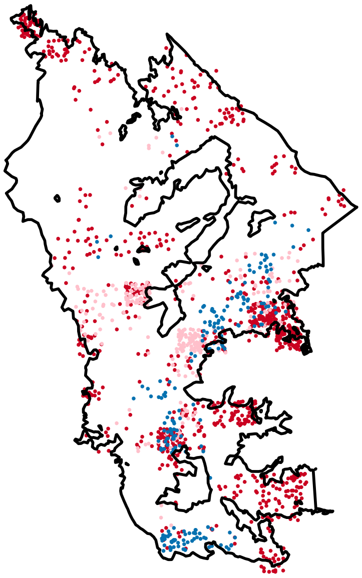

ENVIRONMENT

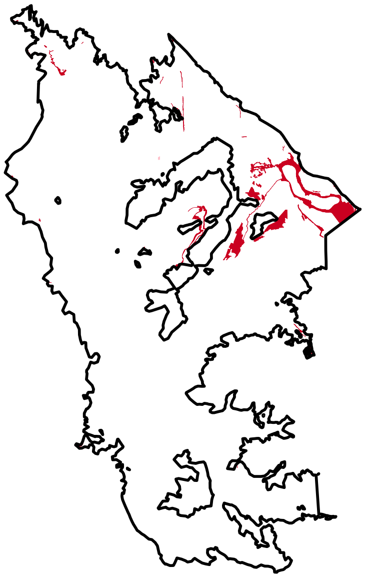

DISADVANTAGED COMMUNITIES

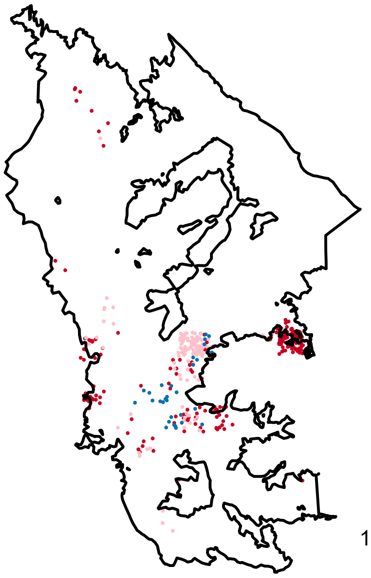

9−007.01 UPPER SAN LUIS REY VALLEY

AGRICULTURE

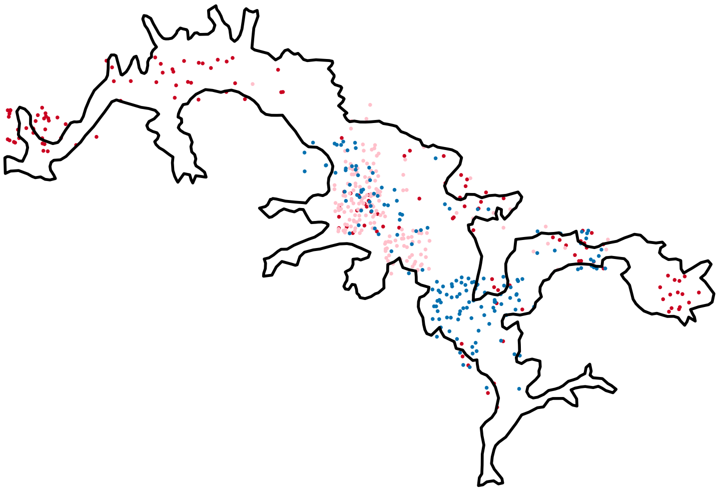

DOMESTIC

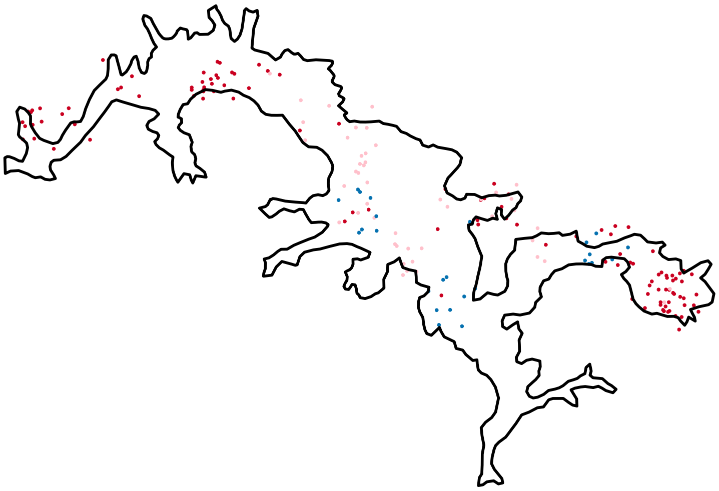

ENVIRONMENT

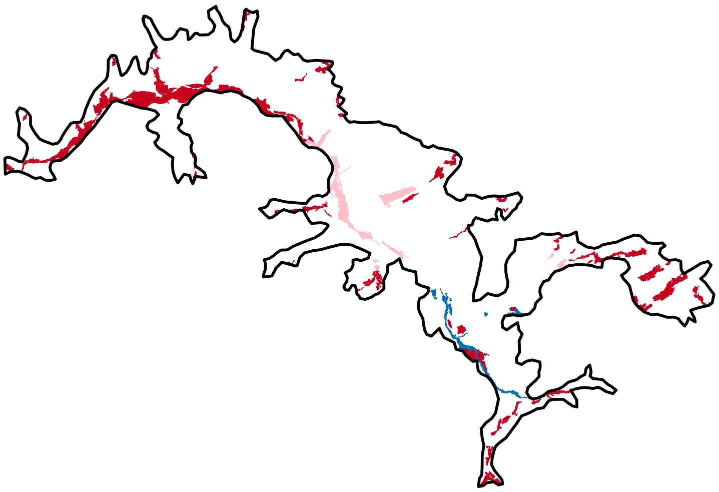

DISADVANTAGED COMMUNITIES

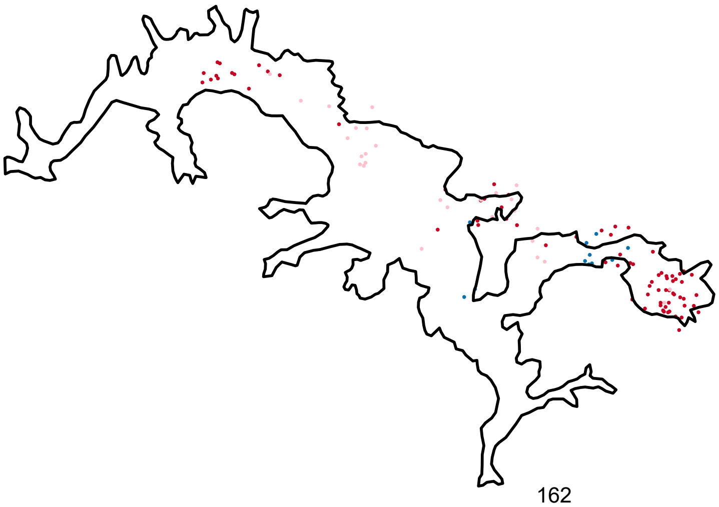

9−010 SAN PASQUAL VALLEY

AGRICULTURE

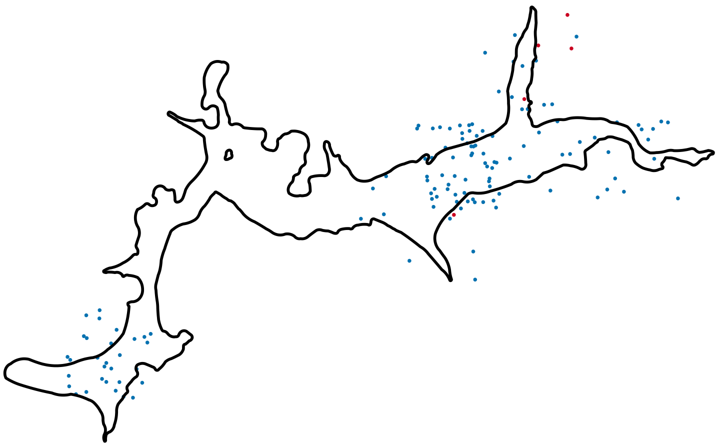

DOMESTIC

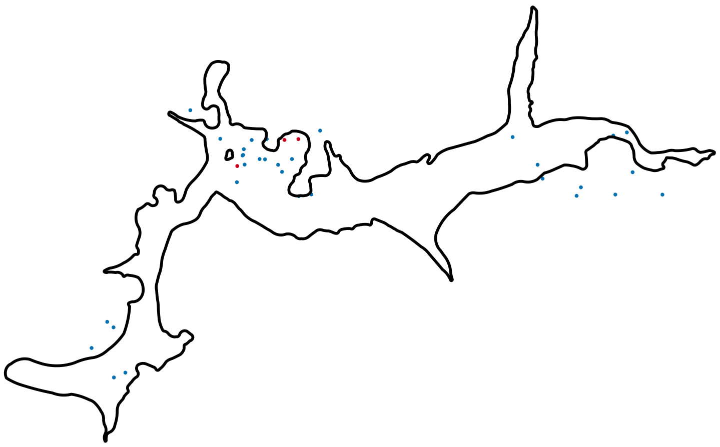

ENVIRONMENT

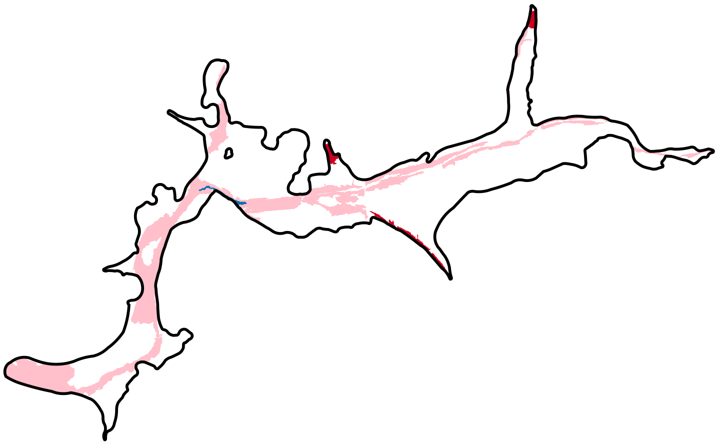

DISADVANTAGED COMMUNITIES

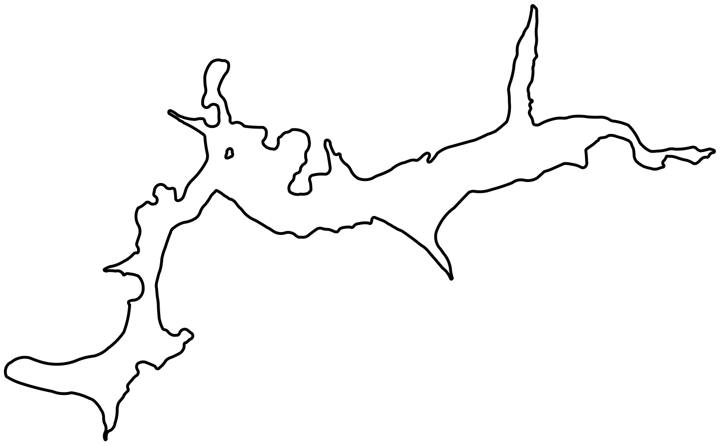

Supplementary Section 6. Integration Statistics

Supplementary Table 6.1. Comparison of mean protection score for lowest third of *aggregate* integration scores with the mean protection score for the highest third of *aggregate* integration scores by stakeholder group. These groups approximate the lowest and highest thirds, while keeping like integer scores together in groups. As the *aggregate* integration scores are integer values, an algorithm to split into thirds splits integer value groups randomly in order to create groups of equal size. To obtain these groups, we first created quartiles and then grouped all equal integer values into the same groups to approximate thirds. Each row is a one-sided t-test with the null hypothesis that the high *aggregate* integration score group has a higher mean protection score than the low *aggregate* integration score group.

Stakeholder group	Mean protection for low group	Mean protection for high group	t score	P value
Agriculture	40.81 (n=36)	48.23 (n=34)	1.28	0.10
Domestic	25.89 (n=39)	46.59 (n=33)	3.68	<0.001***
Environment	6.25 (n=39)	18.67 (n=43)	2.80	<0.01**

Supplementary Table 6.2. ANOVA with Tukey's post hoc between lowest tertile of agriculture, domestic, and environment *aggregate* integration scores.

Test	Group	DF	Sum Sq	Mean Sq	F Value	P Value
**ANOVA**	Stakeholder	2	22598	11299	27.84	<0.001***
	Residuals	111	45053	406	-	-
**Tukey's post hoc**	**Stakeholder 1 for pairwise comparison**	**Stakeholder 2 for pairwise comparison**	**Difference**	**Lower**	**Upper**	**Adjusted P Value**
	Domestic	Agriculture	-14.92	-25.98	-3.86	<0.01**
	Environment	Agriculture	-34.56	-45.62	-23.50	<0.001***
	Environment	Domestic	-19.64	-30.48	-8.80	<0.001***

Supplementary Table 6.3. ANOVA with Tukey's post hoc between highest tertile of agriculture, domestic, and environment *aggregate* integration scores.

**Test**	**Group**	**DF**	**Sum Sq**	**Mean Sq**	**F Value**	**P Value**
**ANOVA**	Stakeholder	2	21694	10847	17.19	<0.001***
	Residuals	107	67525	631	-	-
**Tukey's post hoc**	**Stakeholder 1 for pairwise comparison**	**Stakeholder 2 for pairwise comparison**	**Difference**	**Lower**	**Upper**	**Adjusted P Value**
	Domestic	Agriculture	-1.64	-16.23	12.95	>0.05
	Environment	Agriculture	-29.56	-43.26	-15.85	<0.001***
	Environment	Domestic	-27.92	-41.74	-14.10	<0.001***

Supplementary Table 6.4. Summary of simple ordinary least-squares linear model assessing the relationship between a stakeholder group's aggregate integration score and respective percent coverage and protection by the Sustainability Plan's management criteria. Each line in the Table represents a separate linear model.

Model	Independent Variable	Dependent Variable	Coefficient	Standard Error	P value	Model R squared
1	Agriculture *aggregate* integration score	Agriculture covered (%)	1.18	1.32	0.37	0.01
2	Agriculture *aggregate* integration score	Agriculture protected (%)	0.89	1.29	0.49	<0.01
3	Domestic *aggregate* integration score	Domestic covered (%)	2.83	1.22	0.02	0.05
4	Domestic *aggregate* integration score	Domestic protected (%)	4.34	1.11	< 0.01	0.13
5	Environment *aggregate* integration score	Environment covered (%)	5.50	1.80	<0.01	0.08
6	Environment *aggregate* integration score	Environment protected (%)	2.99	1.25	0.02	0.05

Supplementary Table 6.5. Ordinary least-squares linear model assessing the relationship between the four integration components for agricultural stakeholders and the percent coverage of agricultural wells by the Sustainability Plan's management criteria. Model R squared is 0.04. The coefficient represents the change in the dependent variable associated with a one point increase in the independent variable. Therefore, given that each component variable consists of a three point scale (0-2), we use a metric of two-times the coefficient to capture the two point change between a low score of 0 and a high score of 2.

**Independent Variables**	**Coefficient**	**Standard Error**	**P value**	**Low to High score Difference (2x Coefficient)**
engage agriculture	3.38	3.42	0.33	6.8
describe agriculture	1.35	3.56	0.70	2.7
analyze agriculture	3.97	3.85	0.31	7.9
act agriculture	-3.98	3.50	0.26	-8.0

Supplementary Table 6.6. Ordinary least-squares linear model assessing the relationship between the four integration components for agricultural stakeholders and the percent protection of agricultural wells by the Sustainability Plan's management criteria. Model R squared is <0.01. The coefficient represents the change in the dependent variable associated with a one point increase in the independent variable. Therefore, given that each component variable consists of a three point scale (0-2), we use a metric of two-times the coefficient to capture the two point change between a low score of 0 and a high score of 2.

Independent Variables	Coefficient	Standard Error	P value	Low to High score Difference (2x Coefficient)
engage agriculture	2.40	3.39	0.48	4.8
describe agriculture	-1.06	3.52	0.76	-2.1
analyze agriculture	0.91	3.81	0.81	1.8
act agriculture	1.80	3.46	0.61	3.6

Supplementary Table 6.7. Ordinary least-squares linear model assessing the relationship between the four integration components for domestic stakeholders and the percent coverage of domestic wells by the Sustainability Plan's management criteria. Model R squared is 0.06. The coefficient represents the change in the dependent variable associated with a one point increase in the independent variable. Therefore, given that each component variable consists of a three point scale (0-2), we use a metric of two-times the coefficient to capture the two point change between a low score of 0 and a high score of 2.

**Independent Variables**	**Coefficient**	**Standard Error**	**P value**	**Low to High score Difference**<br><br>**(2x Coefficient)**
engage domestic	1.16	5.13	0.82	2.3
describe domestic	0.63	3.50	0.86	1.3
analyze domestic	3.92	3.39	0.25	7.8
act domestic	4.65	3.69	0.21	9.3

Supplementary Table 6.8. Ordinary least-squares linear model assessing the relationship between the four integration components for domestic stakeholders and the percent protection of domestic wells by the Sustainability Plan's management criteria. Model R squared is 0.17. The coefficient represents the change in the dependent variable associated with a one point increase in the independent variable. Therefore, given that each component variable consists of a three point scale (0-2), we use a metric of two-times the coefficient to capture the two point change between a low score of 0 and a high score of 2.

**Independent Variables**	**Coefficient**	**Standard Error**	**P value**	**Low to High score Difference (2x Coefficient)**
engage domestic	2.47	4.58	0.59	4.9
describe domestic	-1.96	3.13	0.53	-3.9
analyze domestic	7.47	3.02	0.02	15
act domestic	8.19	3.30	0.01	16

Supplementary Table 6.9. Ordinary least-squares linear model assessing the relationship between the four integration components for environment stakeholders and the percent coverage of environment by the Sustainability Plan's management criteria. Model R squared is 0.12. The coefficient represents the change in the dependent variable associated with a one point increase in the independent variable. Therefore, given that each component variable consists of a three point scale (0-2), we use a metric of two-times the coefficient to capture the two point change between a low score of 0 and a high score of 2.

**Independent Variables**	**Coefficient**	**Standard Error**	**P value**	**Low to High score Difference (2x Coefficient)**
engage environment	12.97	4.60	0.01	26
describe environment	-0.76	8.27	0.93	-1.5
analyze environment	5.67	4.73	0.23	11
act environment	1.60	4.14	0.70	3.2

Supplementary Table 6.10. Ordinary least-squares linear model assessing the relationship between the four integration components for environment stakeholders and the percent protection of environment by the Sustainability Plan's management criteria. Model R squared is 0.10. The coefficient represents the change in the dependent variable associated with a one point increase in the independent variable. Therefore, given that each component variable consists of a three point scale (0-2), we use a metric of two-times the coefficient to capture the two point change between a low score of 0 and a high score of 2.

**Independent Variables**	**Coefficient**	**Standard Error**	**P value**	**Low to High score Difference (2x Coefficient)**
engage environment	6.30	3.17	0.05	13
describe environment	0.88	5.71	0.88	1.8
analyze environment	7.86	3.27	0.02	16
act environment	-2.66	2.86	0.35	-5.3

Supplementary Table 6.11. Ordinarily least squares models for disadvantage community coverage and protection by *aggregate* integration score.

Model	Independent Variable	Dependent Variable	Coefficient	Standard Error	P value	Model R squared
1	Disadvantaged communities *aggregate* integration score	Covered (%)	0.16	2.74	0.95	0.00
2	Disadvantaged communities *aggregate* integration score	Percent (%)	2.37	2.67	0.38	0.01

Supplementary Table 6.12. Ordinarily least squares model for disadvantage community well coverage by disadvantaged community integration components.

Independent Variables	Coefficient	Standard Error	P value
engage	-1.91	5.26	0.72
describe	0.27	5.15	0.96
analyze	2.77	9.00	0.76
act	1.24	4.99	0.80

Supplementary Table 6.13. Ordinarily least squares model for disadvantage community well protection by disadvantaged community integration components.

Independent Variables	Coefficient	Standard Error	P value
engage	-6.23	4.99	0.22
describe	5.99	4.89	0.22
analyze	8.80	8.53	0.31
act	3.97	4.73	0.40

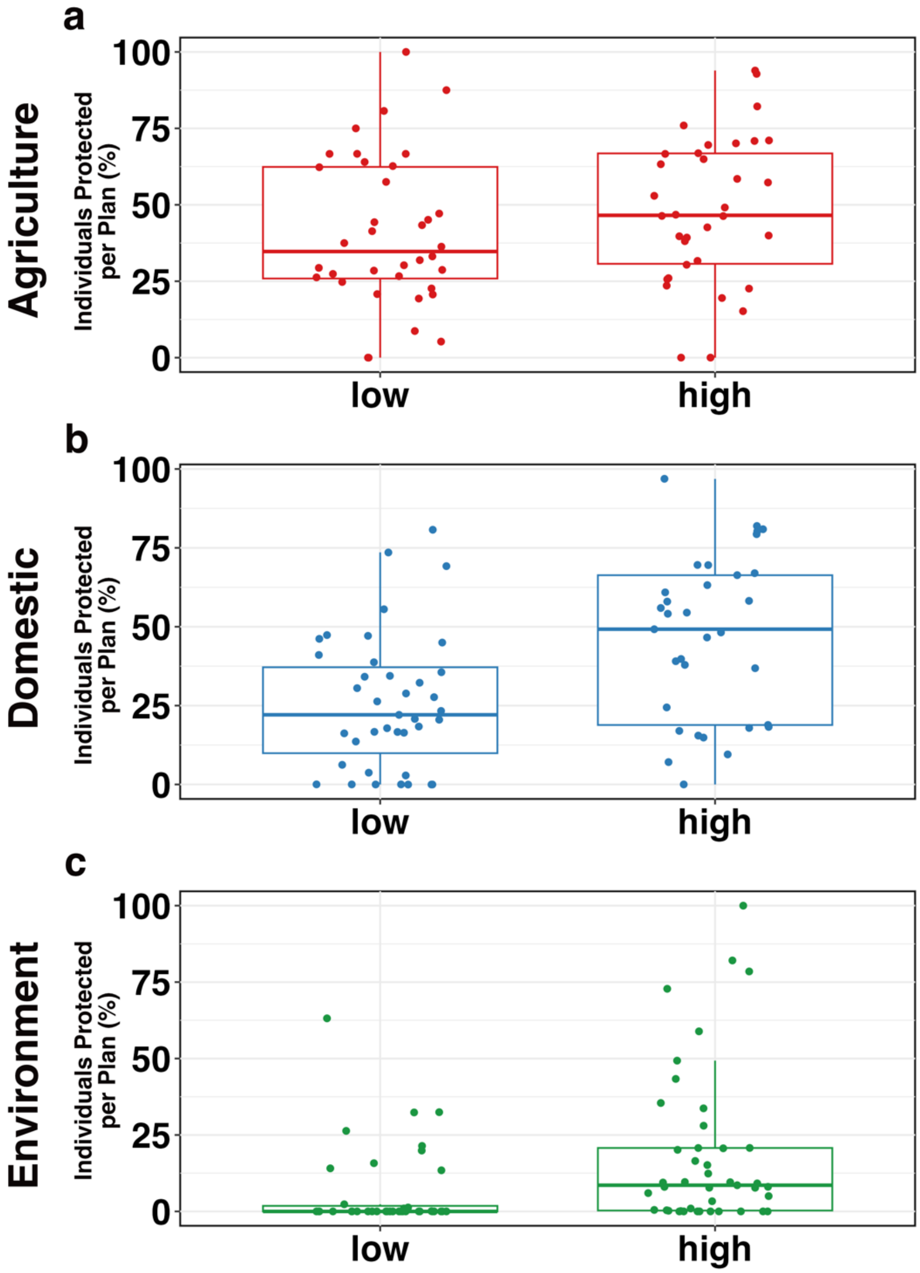

Supplementary Fig. 6.1. High and low tertile integration score boxplots for agriculture, domestic and environment in relation to the percentage of individuals protected with the Sustainability Plan.

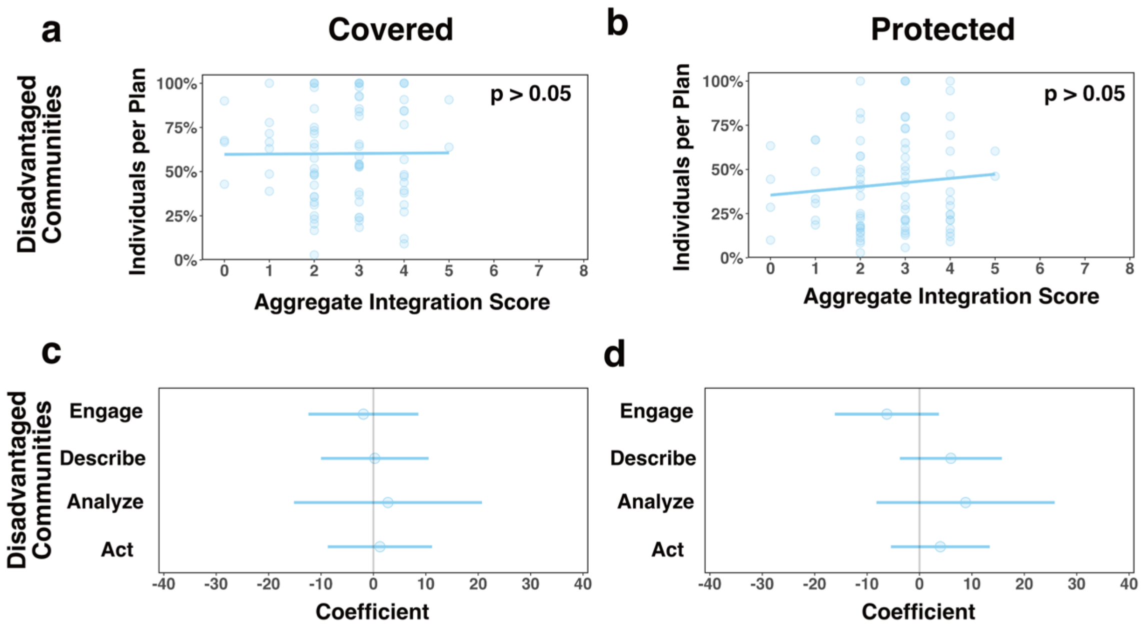

Supplementary Fig. 6.2. Disadvantaged communities integration and its influence on coverage and protection. **(a-b)** Scatter plots showing the relationship between integration *aggregate* scores and percentage covered and protected, including the ordinary least-squares linear fit. The a*ggregate* integration score for disadvantaged communities was not significantly associated with coverage and protection (p>0.05). **(c-d)** Coefficient dot-and-whisker plots showing which integration component(s) predicted whether disadvantaged communities were covered or protected. Dot and whiskers that entirely fall on either side of a coefficient of zero, are considered significant ($p \leq 0.05$). Disadvantaged communities coverage and protection were not significantly influenced by the *engage, describe, analyze,* or *act* scores.

Supplementary Section 7. Integration/Protection Sensitivity Analysis: Ag Engage

To further the sensitivity analysis for the agriculture stakeholder group, described previously in Supplementary Section 3, we ran our ordinary least squares models to assess the relationship between integration and coverage and protection with the alternative versions of *engage* and *aggregate* (defined in Supplementary Table 3.1).

Supplementary Table 7.1. Summary of simple ordinary least squares linear models for variations in the agriculture *aggregate* score. These models assess the relationship between agriculture's *aggregate* integration score and respective percent coverage and protection by the Sustainability Plan's management criteria. Each line in the Table represents a separate linear model.

Model	Independent Variable	Dependent Variable	Coefficient	Standard Error	P value	Model R squared
1	Agriculture *aggregate2* integration score	Covered (%)	-0.97	1.33	0.47	0.01
2	Agriculture *aggregate2* score	Protected (%)	0.20	1.30	0.88	0.00
3	Agriculture *aggregate3* score	Covered (%)	-0.25	1.31	0.85	0.00
4	Agriculture *aggregate3* score	Protected (%)	0.52	1.28	0.68	0.00

Supplementary Table 7.2. Integration component model with *engage2* variation for agriculture and agricultural coverage. Ordinary least squares Linear model assessing the relationship between the *engage2* and the three remaining standard integration components for agricultural stakeholders (i.e., *describe, analyze* and *act*) and the percent coverage of agricultural wells by the Sustainability Plan's management criteria. Model adjusted R squared is 0.13.

**Independent Variables**	**Coefficient**	**Standard Error**	**P value**
Agriculture *engage2*	-12.09	3.53	<0.001
Agriculture *describe*	1.74	3.38	0.61
Agriculture *analyze*	6.36	3.55	0.08
Agriculture *act*	-1.90	3.35	0.57

Supplementary Table 7.3. Integration component model with *engage3* variation for agriculture and agricultural coverage. Ordinary least squares linear model assessing the relationship between the *engage3* and the three remaining standard integration components for agricultural stakeholders (i.e., *describe, analyze* and *act*) and the percent coverage of agricultural wells by the Sustainability Plan's management criteria. Model R squared is 0.06.

**Independent Variables**	**Coefficient**	**Standard Error**	**P value**
Agriculture *engage3*	-5.58	3.29	0.09
Agriculture *describe*	0.93	3.51	0.79
Agriculture *analyze*	6.21	3.75	0.10
Agriculture *act*	-2.80	3.48	0.42

Supplementary Table 7.4. Integration component model with *engage2* variation for agriculture and agricultural protection. Ordinary least squares linear model assessing the relationship between the *engage2* and the three remaining standard integration components for agricultural stakeholders (i.e., *describe, analyze* and *act*) and the percent protection of agricultural wells by the Sustainability Plan's management criteria. Model R squared is <0.01.

**Independent Variables**	**Coefficient**	**Standard Error**	**P value**
Agriculture *engage2*	-2.49	3.68	0.50
Agriculture *describe*	-1.11	3.52	0.75
Agriculture *analyze*	1.91	3.70	0.61
Agriculture *act*	2.40	3.49	0.49

Supplementary Table 7.5. Integration component model with *engage3* variation for agriculture and agricultural protection. Ordinary least squares linear model assessing the relationship between the *engage3* and the three remaining standard integration components for agricultural stakeholders (i.e., *describe, analyze* and *act*) and the percent protection of agricultural wells by the Sustainability Plan's management criteria. Model R squared is < 0.01.

**Independent Variables**	**Coefficient**	**Standard Error**	**P value**
Agriculture *engage3*	0.00	3.30	1.00
Agriculture *describe*	-1.25	3.52	0.72
Agriculture *analyze*	1.62	3.76	0.67
Agriculture *act*	2.05	3.49	0.56

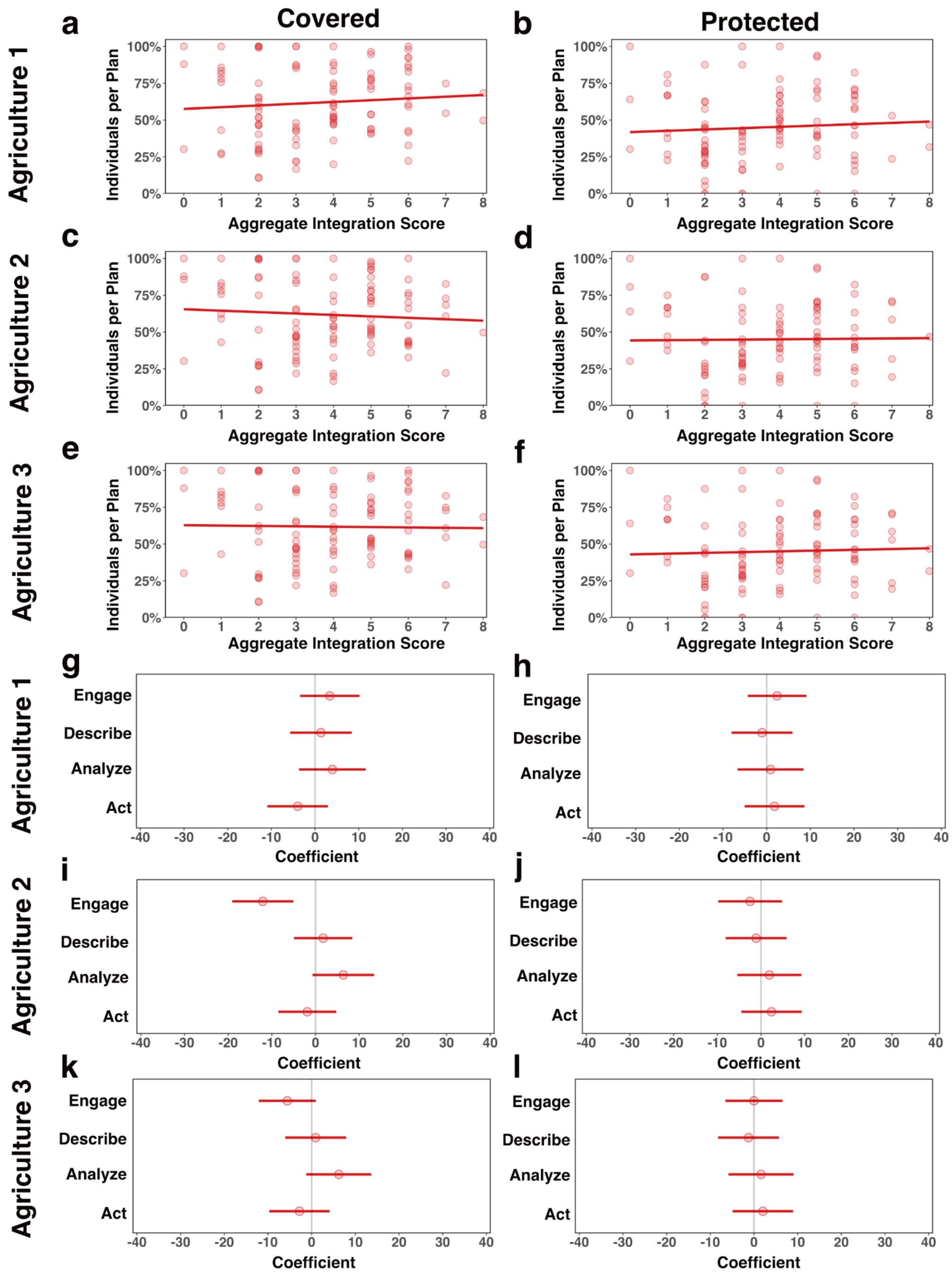

Supplementary Fig. 7.1. Agriculture *aggregate* integration score and *engage* score variants and their relationship to coverage and protection. **a.-f.** Scatter plots showing relationship between agriculture *aggregate* integration score variations *aggregate2* and *aggregate3* with percentage covered and protected by stakeholder group, including the ordinary least-squares linear fit. **g.-l.** Coefficient dot-and-whisker plots of agriculture integration components featuring variations for the *engage* scores: *engage2* and *engage3*. The plots show which integration component(s) predicted whether a particular stakeholder group was covered or predicted. Dot and whiskers that entirely fall on either side of a coefficient of zero, are considered significant ($p \leq 0.05$).

Supplementary Section 8. Post-hoc Exploratory Analyses

Supplementary Table 8.1. Variables used in post-hoc exploratory analyses.

Variable	Description	Variable Type	Range	Source
Sustainability Plan year	Year in which Sustainability Plan was submitted to the California Department of Water Resources. Groundwater Basins designated as critically overdrafted were required to submit their plans by January 31, of 2020, and all other designated high and medium priority basins were required to submit their plans by January 31, 2022.	Categorical	2020; 2022	https://sgma.water.ca.gov/portal/gsp/status
Number of agricultural supply wells	Sum of agricultural supply wells in each Sustainability Plan area within the California Department of Water Resources' Online System of Well Completion Reports database with a recorded construction date 1975-2022	Continuous	1 - 2572	https://water.ca.gov/Programs/Groundwater-Management/Wells/Well-Completion-Reports
Number of domestic supply wells	Sum of domestic supply wells in each Sustainability Plan area within the California Department of Water Resources' Online System of Well Completion Reports database with a recorded construction date 1975-2022	Continuous	2 - 7877	https://water.ca.gov/Programs/Groundwater-Management/Wells/Well-Completion-Reports
Total acreage of groundwater-dependent ecosystems	Sum of acreage of groundwater-dependent ecosystems within a Sustainability Plan area from the California Department of Water Resources' Natural Communities Commonly Associated with Groundwater dataset	Continuous	0.072 - 175.46	Klausmeyer, K. et al. Mapping indicators of groundwater dependent ecosystems in California: Methods report. San Franc. Calif. (2018).

Supplementary Table 8.2. T-tests of stakeholder protection and coverage by Sustainability Plan submission year.

Variable	2020 mean	2022 mean	t score	Pr(>|t|)
agriculture covered	54.82	67.29	-2.61	0.01
agriculture protected	41.06	48.14	-1.50	0.14
domestic covered	50.99	63.59	-2.56	0.01
domestic protected	29.43	41.84	-2.74	0.01
environment covered	43.70	57.95	-2.39	0.02
environment protected	5.13	17.75	-3.52	0.00

Supplementary Table 8.3. Ordinary least squares linear models of stakeholder protection and coverage by the total number of agriculture supply wells, number of domestic supply wells, or total acreage of groundwater-dependent ecosystems within a Sustainability Plan area.

Model	Estimate	Std. Error	t value	Pr(>|t|)	r_squared
agriculture coverage ~ agricultural well supply	-0.02	0.00	-4.12	<0.001***	0.14
agricultural protection ~ agricultural well supply	-0.01	0.00	-1.70	0.09	0.03
domestic coverage ~ domestic well supply	-0.01	0.00	-2.65	0.01*	0.06
domestic protection ~ domestic well supply	0.00	0.00	0.11	0.91	0.00
environment coverage ~ square kilometers of groundwater-dependent ecosystems	-0.32	0.13	-2.38	0.02*	0.05
environmental protection ~ square kilometers of groundwater-dependent ecosystems	-0.11	0.09	-1.19	0.24	0.01

Supplementary Table 8.4. T-tests of stakeholder *aggregate* integration scores by Sustainability Plan submission year.

Stakeholder	2020 mean	2022 mean	t score	Pr(>|t|)
agriculture *aggregate* integration score	2.87	4.19	-3.92	< 0.001***
domestic *aggregate* integration score	2.07	4.17	-6.07	<0.001***
environment *aggregate* integration score	3.11	4.94	-7.22	<0.001***

Supplementary Table 8.5. Ordinary least squares linear models of stakeholder *aggregate* integration scores by the total number of agriculture supply wells, number of domestic supply wells, or total acreage of groundwater-dependent ecosystems within a Sustainability Plan area.

stakeholder	Estimate	Std. Error	t value	Pr(>|t|)	r_squared
agriculture *aggregate* integration score ~ agricultural well supply	0.00	0.00	0.30	0.76	0.00
domestic *aggregate* integration score ~ domestic well supply	0.00	0.00	1.82	0.07	0.03
environment *aggregate* integration score ~ square kilometers of groundwater-dependent ecosystems	0.01	0.01	0.90	0.37	0.01

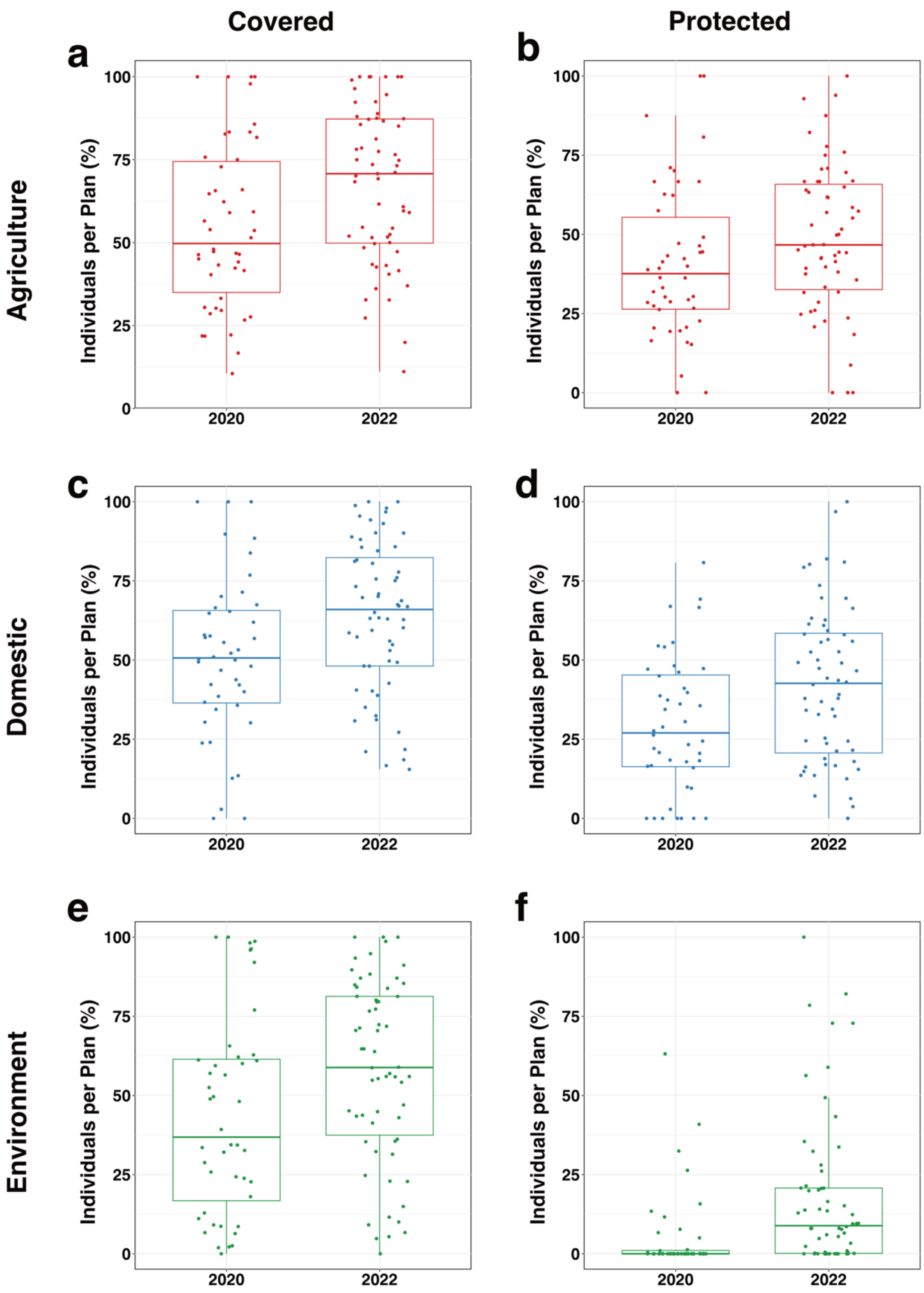

Supplementary Fig. 8.1. Stakeholder coverage and protection by Sustainability Plan submittal year.

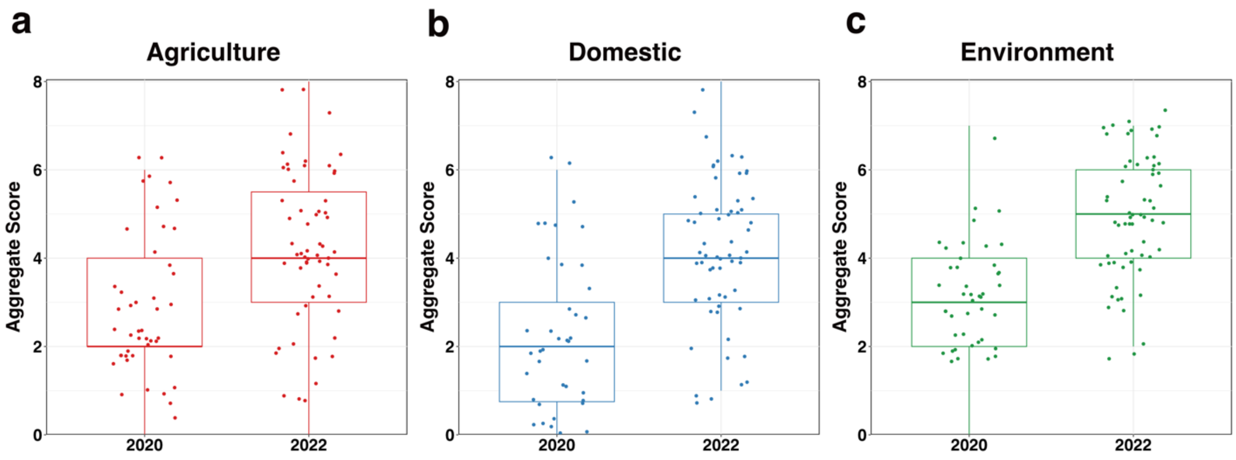

Supplementary Fig. 8.2. Stakeholder *aggregate* integration scores by Sustainability Plan submittal year.

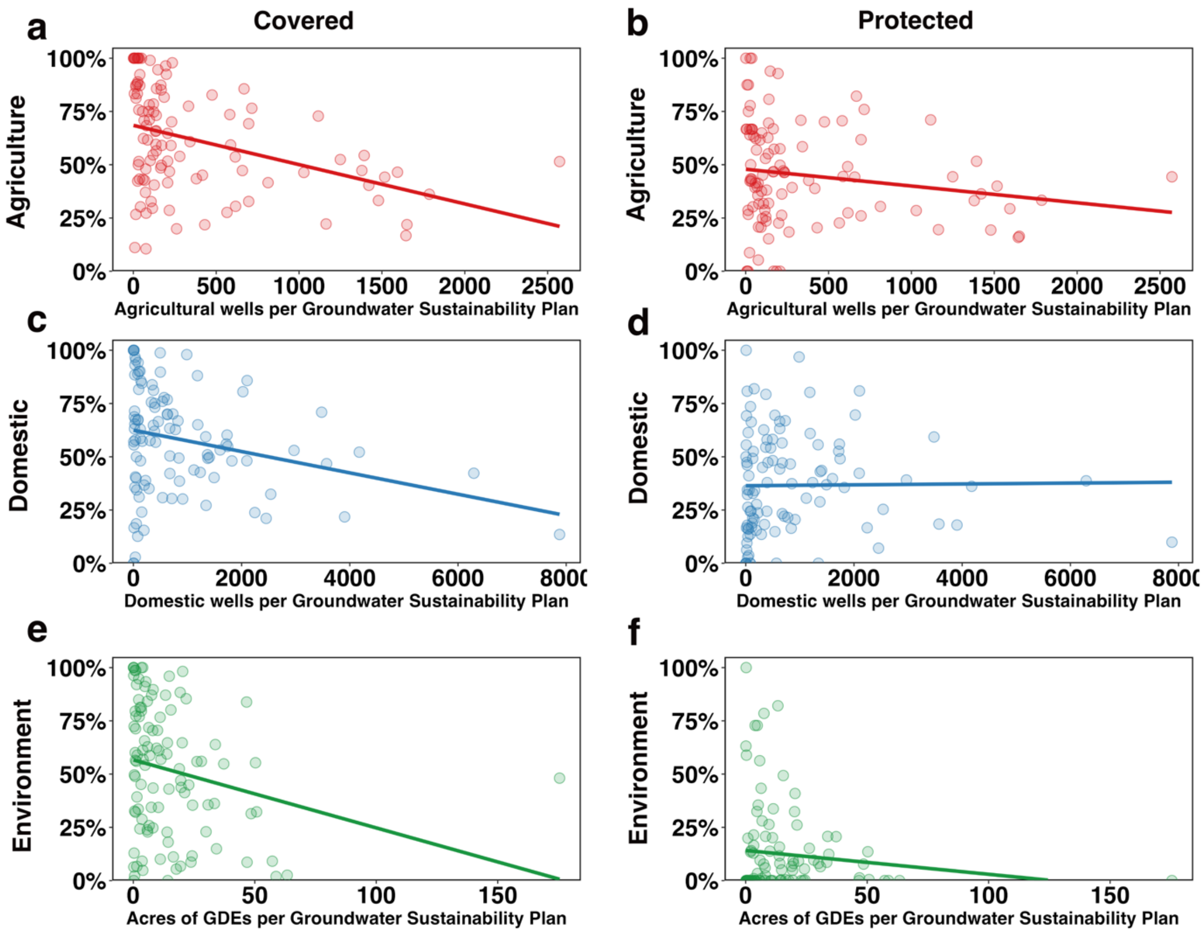

Supplementary Fig. 8.3. Stakeholder coverage and protection by the total number of agriculture supply wells, number of domestic supply wells, or total acreage of groundwater-dependent ecosystems within a Sustainability Plan area.

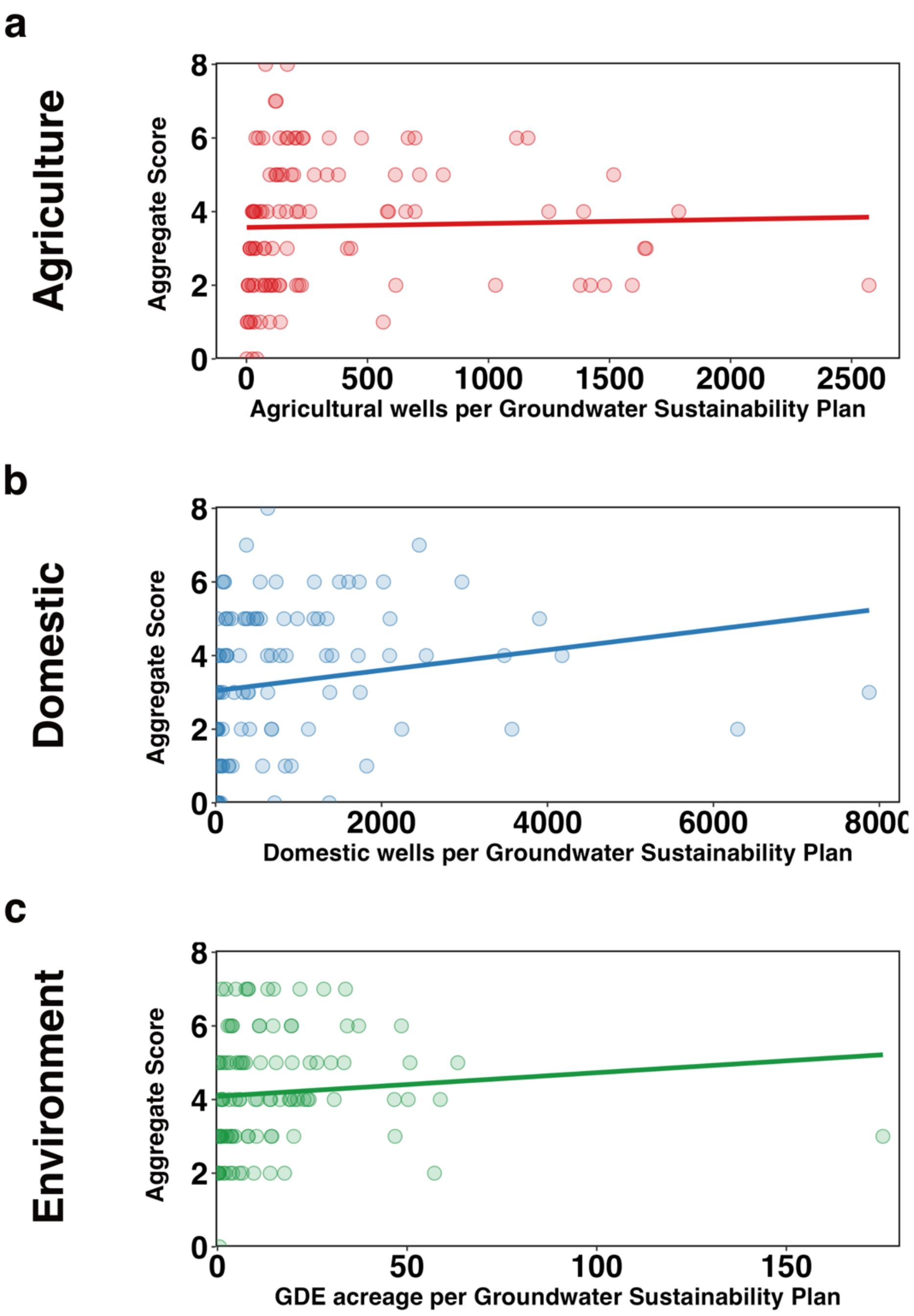

Supplementary Fig. 8.4. Stakeholder *aggregate* integration scores by the total number of agriculture supply wells, number of domestic supply wells, or total acreage of groundwater-dependent ecosystems within a Sustainability Plan area.

Supplementary Section 9. References

1. Thompson, B. H., Rohde, M. M., Howard, J. K. & Matsumoto, S. *Mind the Gaps: The case for truly comprehensive sustainable groundwater management*. https://stacks.stanford.edu/file/druid:hs475mt1364/Mind%20the%20Gaps%2C%20The%20Case%20for%20Truly%20Comprehensive%20Sustainable%20Groundwater%20Management.pdf (2021).

2. California Department of Water Resources Sustainable Groundwater Management Program. *Guidance Document for Groundwater Sustainability Plan: Stakeholder Communication and Engagement*. 20 https://water.ca.gov/Programs/Groundwater-Management/SGMA-Groundwater-Management/Best-Management-Practices-and-Guidance-Documents (2018).

3. Dyble, L. N. Aquifers and Democracy: Enforcing Voter Equal Protection to Save California's Imperiled Groundwater and Redeem Local Government. *Calif. Law Rev.* **105**, 1471–1512 (2017).

4. Nelson, R. L. Assessing local planning to control groundwater depletion: California as a microcosm of global issues. *Water Resour. Res.* **48**, (2012).

5. Blomquist, W. *Dividing the waters: governing groundwater in Southern California.* (ICS Press Institute for Contemporary Studies, 1992).

6. Dennis, E. M., Blomquist, W., Milman, A. & Moran, T. Path Dependence, Evolution of a Mandate and the Road to Statewide Sustainable Groundwater Management. *Soc. Nat. Resour.* **33**, 1542–1554 (2020).

7. Garner, E., McGlothlin, R., Szeptycki, L., Babbitt, C. & Kincaid, V. The Sustainable Groundwater Management Act and the Common Law of Groundwater Rights-Finding a Consistent Path Forward for Groundwater Allocation. *UCLA J Envtl Pol* **38**, 163 (2020).

8. Ulibarri, N., Garcia, N. E., Nelson, R. L., Cravens, A. E. & McCarty, R. J. Assessing the Feasibility of Managed Aquifer Recharge in California. *Water Resour. Res.* **57**, e2020WR029292 (2021).

9. Niles, M. T. & Hammond Wagner, C. R. The carrot or the stick? Drivers of California farmer support for varying groundwater management policies. *Environ. Res. Commun.* **1**, 045001 (2019).

10. Holley, C. & Sinclair, D. Compliance and enforcement of water licences in NSW: limitations in law, policy and institutions. *Australas. J. Nat. Resour. Law Policy* **15**, 149–189 (2012).

11. Ostrom, E. *Governing the commons: The evolution of institutions for collective action*. (Cambridge University Press Cambridge:, 1990).

12. Koontz, T. M. & Thomas, C. W. What Do We Know and Need to Know about the Environmental Outcomes of Collaborative Management? *Public Adm. Rev.* **66**, 111–121 (2006).

13. Ansell, C. & Gash, A. Collaborative Governance in Theory and Practice. *J. Public Adm. Res. Theory* **18**, 543–571 (2008).

14. Newig, J., Challies, E., Jager, N. W., Kochskaemper, E. & Adzersen, A. The Environmental Performance of Participatory and Collaborative Governance: A Framework of Causal Mechanisms. *Policy Stud. J.* **46**, 269–297 (2018).

15. Reed, M. S. *et al.* A theory of participation: what makes stakeholder and public engagement in environmental management work? *Restor. Ecol.* **26**, S7–S17 (2018).

16. Nelson, R. L. Allocations and legal trends in the 21st century. in *Water Resources Allocation and Agriculture: Transitioning from Open to Regulated Access* (eds. Rouillard, J., Babbitt, C., Challies, E. & Rinaudo, J.-D.) 25–36 (IWA Publishing, 2022).

17. Ostrom, E. *Understanding institutional diversity*. (Princeton university press, 2009).

18. Reed, M. S. Stakeholder participation for environmental management: A literature review. *Biol. Conserv.* **141**, 2417–2431 (2008).

19. Yoder, L., Wagner, C. H., Sullivan-Wiley, K. & Smith, G. The Promise of Collective Action for Large-Scale Commons Dilemmas: Reflections on Common-Pool-Resource Theory. *Int. J. Commons* **16**, (2022).

20. Scott, T. Does Collaboration Make Any Difference? Linking Collaborative Governance to Environmental Outcomes. *J. Policy Anal. Manage.* **34**, 537–566 (2015).

21. Bodin, Ö. Collaborative environmental governance: Achieving collective action in social-ecological systems. *Science* **357**, eaan1114 (2017).

22. Eaton, W. M. *et al.* A Conceptual Framework for Social, Behavioral, and Environmental Change through Stakeholder Engagement in Water Resource Management. *Soc. Nat. Resour.* **34**, 1111–1132 (2021).

23. Sterling, E. J. *et al.* Assessing the evidence for stakeholder engagement in biodiversity conservation. *Biol. Conserv.* **209**, 159–171 (2017).

24. Escobedo Garcia, N. & Ulibarri, N. Plan writing as a policy tool: instrumental, conceptual, and tactical uses of water management plans in California. *J. Environ. Stud. Sci.* 1–15 (2022).

25. Klausmeyer, K. *et al.* Mapping indicators of groundwater dependent ecosystems in California: Methods report. *San Franc. Calif.* (2018).

26. Gaventa, J. Finding the Spaces for Change: A Power Analysis. *IDS Bull.* **37**, 23–33 (2006).
